# Structure–Activity Studies on Bis-Sulfonamide SHIP1 Activators

**DOI:** 10.3390/molecules28248048

**Published:** 2023-12-12

**Authors:** Shea T. Meyer, Sandra Fernandes, Robert E. Anderson, Angela Pacherille, Bonnie Toms, William G. Kerr, John D. Chisholm

**Affiliations:** 1 Department of Chemistry, Syracuse University, Syracuse, NY 13244, USA; 2 Department of Microbiology & Immunology, SUNY Upstate Medical University, Syracuse, NY 13210, USA

**Keywords:** sulfonamide, phosphatase, SHIP1, SHIP2, activator

## Abstract

The SH2-containing inositol polyphosphate 5-phosphatase 1 (SHIP1) enzyme opposes the activity of PI3K and therefore is of interest in the treatment of inflammatory disorders. Recent results also indicate that SHIP1 promotes phagolysosomal degradation of lipids by microglia, suggesting that the enzyme may be a target for the treatment of Alzheimer’s disease. Therefore, small molecules that increase SHIP1 activity may have benefits in these areas. Recently we discovered a bis-sulfonamide that increases the enzymatic activity of SHIP1. A series of similar SHIP1 activators have been synthesized and evaluated to determine structure–activity relationships and improve in vivo stability. Some new analogs have now been found with improved potency. In addition, both the thiophene and the thiomorpholine in the parent structure can be replaced by groups without a low valent sulfur atom, which provides a way to access activators that are less prone to oxidative degradation.

## 1. Introduction

**Figure 1:**
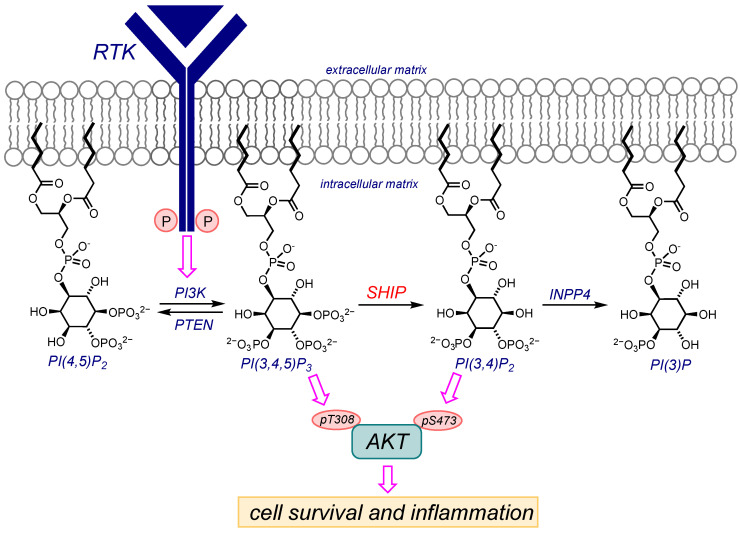
The PI3K Pathway and SHIP.

Inositol phospholipids are critical participants in the PI3K/AKT pathway, a key cell signaling axis in eukaryotic cells. The phosphorylation pattern on the inositol acts as a recognition element for intracellular protein kinases, which, after binding, initiate phosphorylation cascades that contribute to signaling between the cell membrane and the nucleus. Inositol phosphorylation is tightly controlled by inositol kinases and phosphatases, as this signaling influences cell survival [1,2,3,4,5] and inflammatory processes [6,7,8]. The major enzymes involved in metabolizing inositol phospholipids include PI3K, PTEN, SHIP1/2, and INPP4A/B (Figure 1). Modulation of these enzymes can be clinically useful. For example, the activity of phosphatidylinositol-3-kinases (PI3K) has been shown to have profound effects on cellular physiology [9,10]. Once activated, PI3K rapidly synthesizes PI(3,4,5)P_3_ from phosphatidylinositol-4,5-bisphosphate (PI(4,5)P_2_) (Figure 1). PI(3,4,5)P_3_ then recruits and activates the protein phosphatase AKT, which phosphorylates other protein kinases, transmitting signals from the cell membrane to the nucleus. Interest in PI3K has led to a number of small molecules that are clinically useful for the treatment of cancer [11]. The successes with PI3K inhibitors have led to the investigation of other enzymes that metabolize inositol phospholipids, and we have been active in evaluating small molecules that modulate the activity of SHIP [10]. In contrast to PTEN (phosphatase and tensin homolog), which directly reverses the PI3K reaction to generate PI(4,5)P_2_ from PI(3,4,5)P_3_, SHIP generates PI(3,4)P_2_, which can be further dephosphorylated by INPP4. Notably, the PH domain of AKT recognizes both PI(3,4,5)P_3_ and PI(3,4)P_2_, however, and both phosphoinositol species are needed for the full activation of AKT [12]. The activity of AKT may be influenced by modulation of either PI3K or SHIP, and therefore SHIP modulation may also provide a means to treat a number of these disease states [10].

Recently we have been investigating SHIP1 modulators and their therapeutic use in Alzheimer’s disease (AD) [13,14]. Several genome-wide association studies have identified single nucleotide polymorphisms in the INPP5D (SHIP1) gene linked to AD risk [15,16,17,18]. Both SHIP1 and SHIP2 are expressed in microglia [19], and thus both SHIP paralogs could potentially limit microglial homeostatic functions that control amyloidosis and remove dead or dying neurons [19]. SHIP1 expression is increased in the brains of late-onset AD patients and also in 5XFAD mice, which are a common AD model system [20,21,22].

While these results would seem to implicate SHIP1 inhibition as a potential treatment option for AD, other studies suggest a more complex situation [23]. Two groups have published analyses of SHIP1 mutations in 5XFAD mice but have arrived at differing conclusions. One study, which used germline SHIP1 haploinsufficient 5XFAD mice, found that plaque burden was reduced, and behavioral studies in these mice demonstrated a preserved cognitive function [24], supporting the use of SHIP inhibitors. A second study used a conditional knockout of SHIP1 restricted to microglia and macrophages, finding that SHIP1 is required to limit Aβ plaque burden [25], which would implicate the use of SHIP1 activators as a treatment option. One hypothesis for these differing results is that up- or downregulation of SHIP1 enzymatic activity could be beneficial depending on the disease stage. For example, in early stages, patients may benefit from enhanced microglial homeostatic function that is induced by activation, while in late stages, antagonism of SHIP1 may instead be beneficial to reduce damage from dysfunctional microglia. The role of SHIP1 in AD is therefore still under investigation, with potential benefits of up- or downregulation of the enzyme still being evaluated.

**Figure 2:**
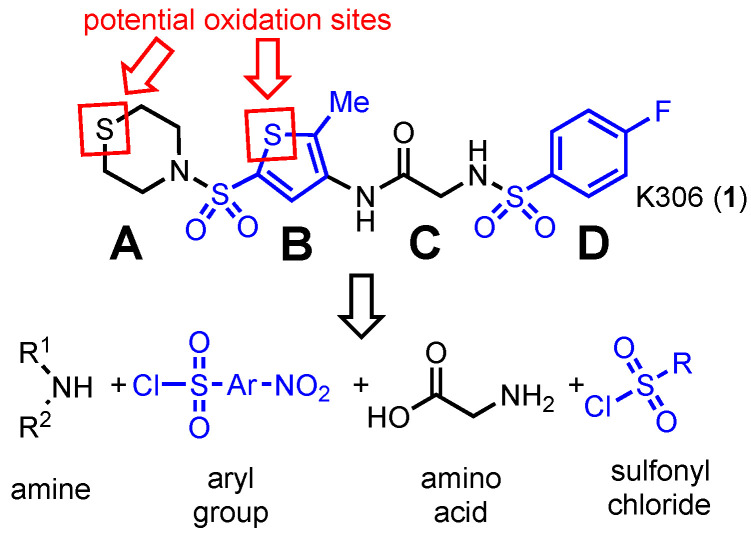
Analysis of K306 structure and potential CYP oxidation sites.

New small molecule SHIP1 activators are therefore needed to explore their effects in AD model systems to define the role of SHIP1 in AD. SHIP is known to be activated by the binding of its product, PI(3,4)P_2_, to an allosteric site on the enzyme near the C2 domain [26]. While some SHIP1 activators (often referred to as enzyme agonists [26,27,28,29]) have demonstrated activity as anti-inflammatories, most suffer from poor bioavailability or low efficacy [13,30,31,32]. Recently we discovered a bis-sulfonamide (K306, **1**, Figure 2) that has significant activity as a SHIP1 activator [14]. K306 enhanced phagolysosomal degradation of synaptosomes and dead neurons in microglial models, which occurred in a SHIP1-dependent manner, revealing a new function for SHIP1 in the brain that could be exploited to enhance microglial function. The K306 structure is related to a series of sulfonamide-based endothelial lipase inhibitors [33]. Sulfonamides have been used previously as phosphate mimics in the development of phosphatase inhibitors, however [34,35], so one of the sulfonamides in the K306 structure may mimic a phosphate on PI(3,4)P_2_, the endogenous ligand for allosteric activation of SHIP1 [36]. Interestingly, K306 was shown to bind to a new (as yet undefined) allosteric site on the protein, as other activators lost activity when the C2 domain of the protein was deleted [36], yet K306 maintained agonism with this C2-less SHIP1 construct [13,14].

Initial in vivo evaluation of K306 in a murine model demonstrated a short half-life, requiring a multiple-dosing regimen to achieve anti-inflammatory responses [14]. This may be due to rapid oxidation of the sulfides in K306 by cytochrome P450 (CYP) enzymes, which are prevalent in mice [37,38]. Evaluation of the K306 structure by SwissADME [39] showed a high likelihood of oxidation, particularly of the sulfide in the thiomorpholine (Figure 2). A lower possibility of oxidation on the thiophene was noted, as the aromaticity of the system and steric effects slow these reactions. The thiophene is still a concern, however, as this heterocycle is known to undergo oxidation by CYP enzymes and can lead to toxic metabolites [40,41].

Given that compound **1** provides a platform for a new class of SHIP1 activators with a potentially new binding mode and interesting biological effects in AD models, synthetic studies were initiated with two primary objectives: (1) determine key structure–activity relationships and (2) find molecules less prone to oxidative degradation. In considering the K306 structure, the molecule may be readily divided into four distinct fragments: the sulfonamide fragment A, the aromatic linker fragment B, the amino acid fragment C, and a second sulfonamide fragment D (Figure 2). Studies to modify each of these fragments were undertaken. Once the most active constituent of each fragment was determined, hybrid molecules that could have even greater potency may be explored by combining the best subunits.

## 2. Results and Discussion

Initially, the sulfonamide of fragment A was varied to determine the necessity for the thiomorpholine and to verify that oxidation of the thiomorpholine sulfide was leading to inactivation. The synthesis of these analogs closely followed the reported synthesis of K306 [33], beginning with the formation of a sulfonamide between the known sulfonyl chloride **2** [42] and different amines (Table 1). Reduction of the nitro group with iron powder in acetic acid then gave the corresponding aromatic amines **3**–**11**. Amide formation with Boc-glycine mediated by HATU provided the carbamates **12**–**20**. Removal of the Boc-protecting groups with TFA (which also produces amine **21**) followed by sulfonamide formation with 4-fluorobenzenesulfonyl chloride gave the bis-sulfonamides **22**–**29**.

While most of the analogs with variations in fragment A were easy to handle, the sulfone analog **23** proved difficult. During the final sulfonamide formation to access **23,** an insoluble solid was formed and filtered from the reaction mixture. This solid was postulated to be the sulfone **23** but was insoluble in all solvents (DMSO, acetone, methanol, chloroform, and water), so verification of the structure by NMR was not feasible. A combustion analysis did match the formula for the desired product, but given the insolubility of this substance, it was not evaluated further.

**Scheme 1:**
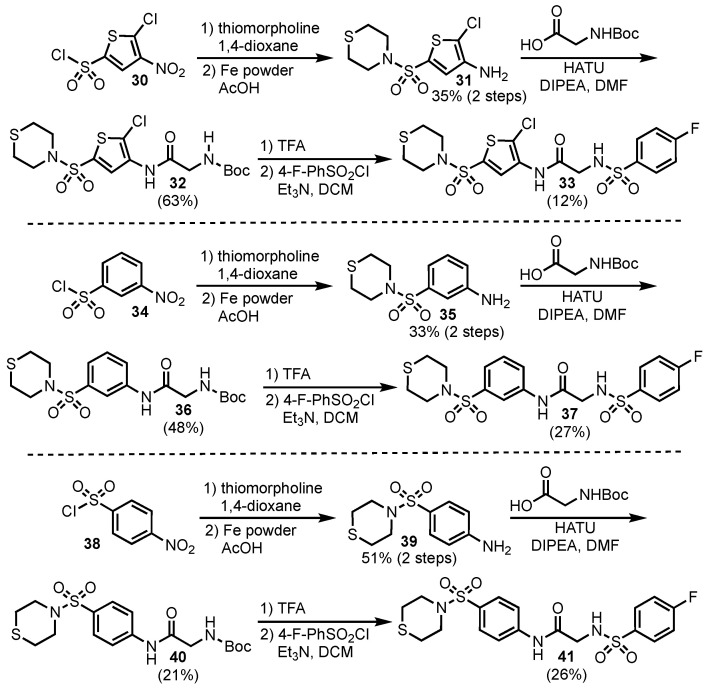
Synthesis of analogs varying fragment B of K306.

The structure of the aryl group in fragment B of the parent structure was also varied. The synthesis of these systems began with the requisite nitro-substituted sulfonyl chloride (**30**, **34**, and **38**, Scheme 1), which were then used in a sulfonamide formation with thiomorpholine. Attempts to directly couple a glycine *N*-sulfonamide to the aromatic amine were unsuccessful, so completion of the molecules was accomplished in a stepwise fashion. After addition of thiomorpholine to the sulfonyl chlorides, the nitro groups were reduced, and an amide coupling was performed with Boc-glycine. Deprotection of the Boc group and sulfonamide formation with 4-fluorobenzenesulfonyl chloride then gave analogs **33**, **37**, and **41**.

The amino acid fragment of the activator (fragment C) was then modified. This was accomplished by using different amino acids as coupling partners following the general pathway shown in Table 2 below. The amine **3** was coupled with a Boc-protected amino acid utilizing HATU as the coupling agent. Removal of the Boc-protecting group was then followed by sulfonamide formation with 4-fluorobenzenesulfonyl chloride. This led to the synthesis of compounds **48**–**53**.

Additionally, analogs of K306 with different groups in fragment D were also synthesized starting from amine **21** (Table 3). Modification of this region of the molecule was accomplished by adding different electrophiles to the amine **21**. A number of different groups on the sulfonamide were synthesized, leading to **64**–**71**. These additions were not restricted to sulfonyl chlorides as coupling partners, with acid chlorides and chloroformates also being investigated, leading to the synthesis of **72** and **73**.

**Figure 3:**
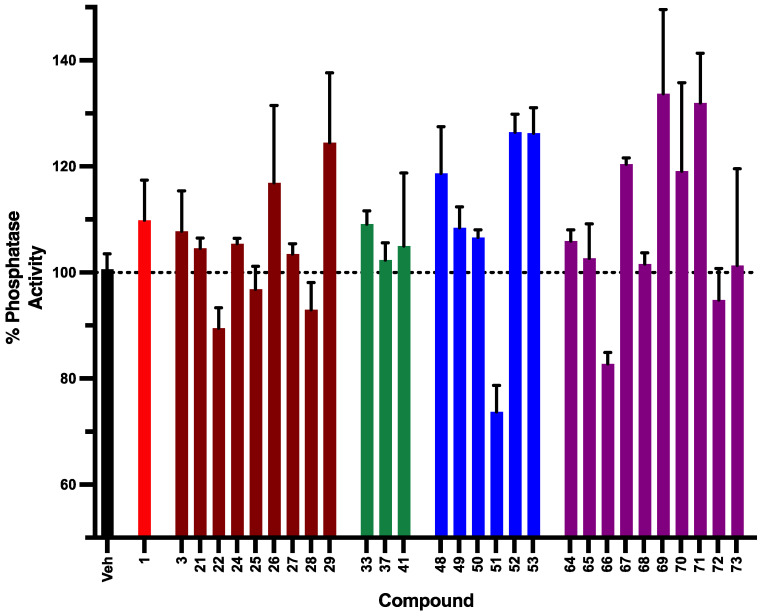
Evaluation of SHIP1 Activation in the Malachite Green Assay. Percentage of phosphatase activity for SHIP1 and SHIP2 enzyme as determined in the malachite green assay as compared to a vehicle control (veh, DMSO) used as 100% activity. The assay was performed at 250 µM concentration for the small molecule activator with 100 µM PI(3,4,5)P_3_-diC8 as the substrate. Results are the mean of at least three trials. Values >100% are due to activation, while values <100% show inhibition of the enzyme.

Evaluation of the effects of these analogs on SHIP1 utilizing the malachite green assay for phosphatase activity showed some interesting trends in reactivity (Figure 3). The malachite green assay is based on the quantification of the green complex formed between malachite green dye, molybdate, and free orthophosphate under acidic conditions [43]. The rapid color formation from this reaction can be conveniently measured and quantified at 620 nm on a plate reader. As the malachite reaction requires free phosphate, the quantity of free phosphate generated from a substrate over a short time (in this case 20 min) can be measured and directly related to phosphatase activity. For in vitro assays with SHIP, PI(3,4,5)P_3_-diC8 [44] is used as the substrate, as the endogenous PI(3,4,5)P_3_ aggregates without a lipid membrane, and is not recognized by SHIP. The malachite results showed that analogs that were truncated and missing fragment D sulfonamide or the amino acid and sulfonamide fragments (**3** and **21**) were less effective SHIP1 activators than K306. Sulfoxide **22**, where the thiomorpholine sulfide has been oxidized to a sulfoxide, also showed no SHIP1 activation, which is consistent with oxidation of the sulfur being a problem that needs to be addressed in this class of SHIP1 activators. Interestingly sulfoxide **22** showed inhibition of SHIP1. As the endogenous ligand of allosteric activation of SHIP1 is the product PI(3,4)P_2_, and this is similar in structure to PI(3,4,5)P_3_, the active site and the allosteric binding site may share recognition elements that allow small structural changes to switch the role of the molecule from activator to inhibitor. Replacement of the thiomorpholine with a morpholine or a pyrrolidine (**24** and **25**) also resulted in a loss of activity. The use of a more lipophilic piperidine did provide an active analog (**26**) that was more active than K306, however. Acyclic systems in fragment A were also explored with analogs **27**–**29**. A rough trend that favored the less polar derivatives was noted with these analogs, with the butylamine analog **29** being the most active. This may indicate that this fragment of the inhibitor is binding in a nonpolar region of the protein.

The analogs with different aromatic spacer groups (fragment B, compounds **33**, **37,** and **41**) were then evaluated. Replacing the 2-methylthiophene with a 2-chlorothiophene gave analog **33,** which was nearly equipotent as the parent K306 structure. The 2-chlorothiophene that was chosen as chlorine may slow oxidation of the thiophene sulfur due to steric and electronic effects. Attempts were also made to replace the thiophene ring with a more stable benzene ring. This led to the synthesis of benzenesulfonamides **37** and **41**. Some activity was maintained with a phenyl ring when the sulfonamide and the amide were in a 1,4-orientation (sulfonamide **41**); however, the 1,3-orientation (compound **40**) was significantly less active.

Evaluation of analogs with changes in amino acid fragment (fragment C) was then undertaken (compounds **48–53**, Figure 3). Analogs employing both enantiomers of alanine and phenylalanine were first investigated, with the results appearing to indicate that the presence of an (*S*) stereocenter led to compounds with better activator activity than the (*R*) enantiomers. Analogs bearing a phenylglycine and a proline in fragment C showed significantly improved activator activity compared to the parent compound.

Modification of the second sulfonamide fragment (fragment D) was then investigated. Deletion of the fluorine (as in **64**) or incorporation of a less polar group on the benzenesulfonamide (as in **65** and **66**) led to a loss of activator activity. Switching to the more polar 4-nitro-substituted benzenesulfonamide **67** gave an increase in the activity, so the 3-nitro and 2-nitro sulfonamides were also synthesized and evaluated. While the 3-nitrobenzenesulfonamide analog **68** was disappointing, the 2-nitrobenzenesulfonamide **69** was quite active. While the activity was promising, nitro groups can be problematic in the development of bioactive small molecules, as nitroreductases often will reduce the nitro group to an electrophilic nitroso derivative, which can have mutagenic effects and exhibit hepatotoxicity [45]. While the nitro group in **69** was relatively hindered, this was still a concern, so some other bioisosteres for a nitro group were then evaluated beginning with the trifluoromethyl group [46] in **70**, which had also showed good activator activity. An ester has also been used as a nitro bioisostere [47], but incorporation of the ester next to the sulfonamide led to the spontaneous cyclization of the sulfonamide nitrogen to form the benzoic sulfimide **71**. Sulfimide **71** displayed promising SHIP1 activator activity in the malachite green assay, nearly as potent as **69**, demonstrating that a polar group in this position is beneficial for bioactivity. Other functional groups in lieu of the sulfonamide in fragment D including the benzamide **72** and the carbamate **73** have little effect on SHIP activity.

## 3. Experimental

### 3.1. General Experimental Information

All anhydrous reactions were run under a positive pressure of argon. Dichloromethane (DCM) was dried by passage through an alumina column. 1,2-Dichloroethane (DCE), di-isopropylethylamine (DIPEA), triethylamine (TEA), and dimethylacetamide (DMA) were freshly distilled from calcium hydride before use. Tetrahydrofuran (THF) was freshly distilled from Na/benzophenone still before use. Ethyl acetate (EA) and hexanes were purchased from commercial sources and used as received. Silica gel column chromatography was performed using 60 Å silica gel (230–400 mesh). Copies of ^1^H NMR and ^13^C NMR spectra are available online in the supplementary materials.

### 3.2. General Procedure for the Synthesis of Sulfonamides ***3***–***11***

The amine (2 equiv) and 5-methyl-4-nitrothiophene-2-sulfonyl chloride (**3**) (1 equiv) were dissolved in 1,4-dioxane (1.0 M) and heated to 60 °C. The mixture was stirred for 1 h at 60 °C, after which the reaction was cooled to rt and 20 mL of water was added. The mixture was extracted with DCM (3 × 20 mL), and the organics were dried over MgSO_4_, filtered, and concentrated. The crude mixture was purified via silica gel chromatography, yielding the nitrothiophene intermediate. This nitrothiophene was dissolved in acetic acid (0.3 M) and iron power (5 equiv) was added. The mixture was heated to 60 °C and stirred for 1.5 h followed by removal of acetic acid in vacuo. The residue was taken up in EA and the iron powder was filtered away. The solution was washed with saturated NaHCO_3_ until a pH of 8 was reached. The organic layer was then dried over Na_2_SO_4_, filtered, and concentrated. The residue was purified via silica gel chromatography, yielding the desired amine.

*2-Methyl-5-thiomorpholine-4-sulfonyl)thiophen-3-amine* (**3**). Dark orange powder (0.230 g, 52%). mp = 140–146 °C; TLC R_f_ = 0.12 (30% EA/hexanes); IR (ATR) 3361 (NH_2_), 2913 (CH), 2852 (CH), 1565 (C=C), 1327 (SO_2_) cm^−1^; ^1^H NMR (CDCl_3_, 400 MHz) δ 7.54 (s, 1H, ArH), 3.72 (br s, 2H, NH_2_), 3.64 (t, *J* = 4.6 Hz, 4H, O_2_SN-CH_2_), 3.01 (t, *J* = 4.8, 4H, S-CH_2_-), 2.54 (s, 3H, CH_3_); ^13^C NMR (CDCl_3_, 100 MHz) δ 140.0, 130.5, 125.6, 122.0, 48.0 (O_2_SN-CH_2_), 27.3 (S-CH_2_-), 11.8 (CH_3_). Anal. Calcd for C_9_H_14_N_2_O_2_S_3_: C, 38.83; H, 5.09; N, 10.06. Found: C, 39.17; H, 4.73; N, 9.84.
*4-[(4-Amino-5-methylthiophen-2-yl)sulfonyl]-1-thiomorpholin-1-one* (**4**). Yellow solid (0.161 g, 99%). mp = 151–154 °C; TLC R_f_ = 0.54 (50% DCM/50% MeOH); IR (ATR) 3386 (NH), 3322 (NH), 2917 (CH), 2858 (CH), 1334 (SO_2_), 1017 (S=O) cm^−1^; ^1^H NMR (CD_3_OD, 400 MHz) δ 7.11 (s, 1H, ArH), 3.69–3.73 (m, 2H), 3.28–3.34 (m, 2H), 2.97–3.00 (m, 4H), 2.27 (s, 3H, CH_3_); ^13^C NMR (CD_3_OD, 100 MHz) δ 142.8, 128.7, 126.3, 120.7, 44.0 (CH_2_), 36.7 (CH_2_), 10.2 (CH_3_); Anal. Calcd for C_9_H_14_N_2_O_3_S_3_: C, 36.72; H, 4.79; N, 9.52. Found: C, 36.43; H, 4.73; N, 9.32.
*4-[(4-Amino-5-methylthiophen-2-yl)sulfonyl]-1-thiomorpholine-1,1-dione* (**5**). Yellow solid (0.237, 19%). mp = 194–199 °C; TLC R_f_ = 0.11 (50% EA/hexanes); IR (ATR) 3448 (NH), 3371 (NH), 3071(ArH), 2930 (CH), 2852 (CH), 1351 (SO_2_) cm^−1^; ^1^H NMR (CD_3_OD, 400 MHz) δ 7.13 (s, 1H, ArH), 3.59–3.61 (m, 4H), 3.22 (t, *J* = 5.4 Hz, 4H), 2.29 (s, 3H, CH_3_); ^13^C NMR (CD_3_OD, 100 MHz) δ 142.8, 129.3, 126.4, 121.0, 50.4 (CH_2_), 45.1 (CH_2_), 10.2 (CH_3_); Anal. Calcd for C_9_H_14_N_2_O_4_S_3_: C, 34.83; H, 4.55; N, 9.03. Found: C, 34.99; H, 4.19; N, 8.86.
*2-Methyl-5-(morpholine-4-sulfonyl)thiophen-3-amine* (**6**). Orange solid (0.80 g, 89%). TLC R_f_ = 0.22 (3% MeOH/CHCl_3_); ^1^H NMR (CDCl_3_, 400 MHz) δ 7.00 (s, 1H, ArH), 3.76 (t, *J =* 4.7 Hz 4H, CH_2_), 3.45 (bs, 2H, NH_2_), 3.04 (t, *J* = 4.8 Hz, 4H, CH_2_), 2.27 (s, 3H, CH_3_); ^13^C NMR (CDCl_3_, 100 MHz) δ 141.2, 128.5, 126.0, 121.1, 66.1 (CH_2_), 46.0 (CH_2_), 11.7 (CH_3_).
*2-Methyl-5-(pyrrolidine-1-sulfonyl)thiophen-3-amine* (**7**). Tan solid (0.41 g, 58%). mp = 152–155 °C; TLC R_f_ = 0.22 (50% EA/hexanes); IR (ATR) 3452 (NH), 3367 (NH), 2980 (CH), 2863 (CH), 1326 (SO_2_) cm^−1^; ^1^H NMR (CD_3_OD, 400 MHz) δ 7.09 (s, 3H, ArH), 3.24 (t, *J =* 6.6 Hz, 4H, N-CH_2_-), 2.27 (s, 3H, CH_3_), 1.77 (t, *J* = 6.8 Hz, 4H, CH_2_); ^13^C NMR (CDCl_3_, 100 MHz) δ 136.2, 131.4, 129.2, 126.0, 48.2 (CH_2_), 25.4 (CH_2_), 12.1 (CH_3_); Anal. Calcd for C_9_H_14_N_2_O_2_S_2_: C, 43.88; H, 5.73; N, 11.34. Found: C, 43.60; H, 5.36; N, 11.20.
*2-Methyl-5-(piperidine-1-sulfonyl)thiophen-3-amine* (**8**). Tan solid (0.63 g, 70%). mp = 96–101 °C; TLC R_f_ = 0.30 (50% EA/hexanes); IR (ATR) 3426 (NH), 3349 (NH), 2979 (CH), 2949 (CH), 1618 (C=C), 1334 (SO_2_) cm^−1^; ^1^H NMR (CDCl_3_, 400 MHz) δ 7.02 (s, 1H, ArH), 3.75 (bs, 2H, NH), 3.01 (t, *J =* 5.5 Hz, 4H, N-CH_2_-), 2.29 (s, 3H, CH_3_), 1.67 (p, *J* = 5.8 Hz, 4H, CH_2_), 1.45 (p, *J* = 6.0 Hz, 2H, CH_2_); ^13^C NMR (CDCl_3_, 100 MHz) δ 139.5, 130.3, 125.6, 121.9, 47.0, 25.1, 23.5, 11.8; Anal. Calcd for C_10_H_16_N_2_O_2_S_2_: C, 46.13; H, 6.19; N, 10.76. Found: C, 46.00; H, 6.45; N, 10.66.
*4-Amino-N,N,5-trimethylthiophene-2-sulfonamide* (**9**). Yellow solid (0.240 g, 39%). mp = 144–146 °C; TLC R_f_ = 0.46 (50% EA/hexanes); IR (ATR) 3454 (NH), 3372 (NH), 3063 (ArH), 2975 (CH), 2908 (CH), 1630 (C=C), 1324 (SO_2_) cm^−1^; ^1^H NMR (CD_3_OD, 400 MHz) δ 7.06 (s, 1H, ArH), 2.69 (s, 6H, N(CH_3_)_2_), 2.27 (s, 3H, CH_3_); ^13^C NMR (CD_3_OD, 100 MHz) δ 142.3, 127.8, 126.2, 120.1, 37.0 (N(CH_3_)_2_), 10.1 (CH_3_); Anal. Calcd for C_7_H_12_N_2_O_2_S_2_: C, 38.16; H, 5.49; N, 12.72. Found: C, 38.09; H, 5.76; N, 12.85.
*4-Amino-N,N-diethyl-5-methylthiophene-2-sulfonamide* (**10**). Orange solid (0.150 g, 30%). mp = 72–74 °C; TLC R_f_ = 0.26 (50% EA/hexanes); IR (ATR) 3434 (NH), 3356 (NH), 2982 (CH), 2937 (CH), 1320 (SO_2_) cm^−1^; ^1^H NMR (CDCl_3_, 400 MHz) δ 6.98 (s, 1H, ArH), 3.52 (bs, 2H, NH_2_), 3.16 (q, *J =* 7.2 Hz, 4H, N-CH_2_-), 2.20 (s, 3H, CH_3_), 1.13 (t, *J =* 7.2 Hz, 6H, CH_3_); ^13^C NMR (CDCl_3_, 100 MHz) δ 140.8, 133.7, 125.1, 119.5, 42.7 (N-CH_2_-), 14.3 (CH_3_), 11.6 (CH_3_); Anal. Calcd for C_9_H_16_N_2_O_2_S_2_: C, 43.53; H, 6.49; N, 11.28. Found: C, 43.18; H, 6.19; N, 11.48.
*4-Amino-N-butyl-5-methylthiophene-2-sulfonamide* (**11**). Off-white solid (0.384 g, 51%). mp = 65–67 °C; TLC R_f_ = 0.32 (50% EA/hexanes); IR (ATR) 3371 (NH), 3313 (NH), 3094 (ArH), 2967 (CH), 2869 (CH), 1316 (SO_2_) cm^−1^; ^1^H NMR (CDCl_3_, 400 MHz) δ 7.06 (s, 1H, ArH), 4.86 (t, *J =* 5.9 Hz, 1H, RNHSO2Ar), 3.65 (bs, 2H, NH_2_), 2.96 (q, *J* = 6.8 Hz, 2H, N-CH_2_-), 2.23 (s, 3H, CH_3_), 1.45 (p, *J =* 7.1 Hz, 2H, CH_2_-C*H*_2_-CH_2_), 1.29 (sextet, *J =* 7.2 Hz, 2H, CH_2_-C*H*_2_-CH_3_), 0.86 (t, *J =* 7.4 Hz, 3H, CH_3_); ^13^C NMR (CDCl_3_, 100 MHz) δ 140.8, 133.9, 125.6, 120.3, 43.2 (N-CH_2_-), 31.4 (CH_2_), 19.8 (CH_2_), 13.6 (CH_3_), 11.7 (CH_3_); Anal. Calcd for C_9_H_16_N_2_O_2_S_2_: C, 43.53; H, 6.49; N, 11.28. Found: C, 43.63; H, 6.79; N, 11.04.

### 3.3. General Procedure for the Synthesis of Amides ***12***–***20***

The desired Boc-protected amino acid (1.2 equiv), thiophenylamine (1 equiv), HATU (2 equiv), and DIPEA (2 equiv) were dissolved in dry DMF (0.08 M) under argon. The mixture was stirred for 24 h at rt. The reaction was diluted with EA and washed with NH_4_Cl (3 × 15 mL) and brine. The organic layer was then separated, dried over MgSO_4_, filtered, and concentrated. The residue was purified via silica gel chromatography, yielding the desired amide product.

*tert-Butyl N-(([2-methyl-5-(thiomorpholine-4-sulfonyl)thiophen-3-yl]carbamoyl)methyl) carbamate* (**12**). Yellow oil (0.250 g, 76%). TLC R_f_ = 0.31 (50% EA/hexanes); IR (ATR) 3254 (NH), 2980 (CH), 2971 (CH), 1665 (C=O), 1350 (SO_2_) cm^−1^; ^1^H NMR (CDCl_3_, 400 MHz) δ 8.55 (br s, 1H, ArNHCO2R), 7.78 (s, 1H, ArNHR), 5.60 (t, *J* = 5.7 Hz, 1H, NHBoc), 3.89 (d, *J* = 5.6 Hz, 2H, CH_2_), 3.32 (t, *J* = 4.0 Hz, 4H, NCH_2_), 2.68 (t, *J* = 5.2 Hz, 4H, S-CH_2_-), 2.32 (s, 3H, CH_3_), 1.43 (s, 9H, OtBu); ^13^C NMR (CDCl_3_, 100 MHz) δ 168.0 (C=O), 156.9 (C=O), 132.8, 132.2, 130.9, 128.6, 80.9 (OC(Me)_3_), 47.9 (CH_2_), 45.1 (CH_2_), 28.3 (C(*C*H_3_)_3_), 27.2 (CH_2_), 12.4 (CH_3_). This compound has been previously reported [14].
*tert-Butyl-N-[({2-methyl-5-[(1-oxo-1-thiomorpholin-4-yl)sulfonyl]thiophen-3-yl}carbamoyl)methyl] carbamate* (**13**). Clear oil (0.200 g, 73%). TLC R_f_ = 0.17 (100% EA); IR (ATR) 3283 (NH), 2979 (CH), 2929 (CH), 1683 (C=O), 1350 (SO_2_), 1150 (SO) cm^−1^; ^1^H NMR (CDCl_3_, 400 MHz) δ 8.55 (bs, 1H, NH), 8.04 (s, 1H, ArH), 5.25 (bs, 1H, NH), 3.89 (d, *J* = 4.5 Hz, 2H), 3.80 (d, *J* = 13.3 Hz, 2H), 3.45 (t, *J* = 11.5 Hz, 2H), 2.94 (d, *J* = 13.9 Hz, 2H), 2.79–2.86 (m, 2H), 2.38 (s, 3H, ArCH_3_), 1.49 (s, 9H, OtBu); ^13^C NMR (CD_3_OD, 100 MHz) δ 171.2 (C=O), 169.6 (C=O), 136.0, 132.5, 130.3, 130.1, 79.4 (OC(Me)_3_), 44.0, 43.3, 36.7, 27.3 (C(*C*H_3_)_3_), 11.2 (CH_3_); Anal. Calcd for C_16_H_25_N_3_O_6_S_3_: C, 42.56; H, 5.58; N, 9.34. Found: C, 42.28; H, 5.63 N, 9.70.
*tert-Butyl-N-[({5-[(1,1-dioxo-1-thiomorpholine-4-yl)sulfonyl]-2-methylthiophen-3-yl}carbamoyl) methyl] carbamate* (**14**). Light-yellow powder (0.140 g, 39%). mp = 187–190 °C; TLC R_f_ = 0.51 (30% EA/hexanes); IR (ATR) 3273 (NH), 2987 (CH), 2928 (CH), 1689 (C=O), 1670 (C=O), 1308 (SO_2_), 1150 (SO_2_) cm^−1^; ^1^H NMR (CD_3_OD, 400 MHz) δ 7.74 (s, 1H, ArH), 3.88 (s, 2H, CH_2_NBoc), 3.65 (bs, 4H, O_2_SCH_2_-), 3.23 (t, *J* = 5.4 Hz, 4H, N-CH_2_-), 2.42 (s, 3H, CH_3_), 1.47 (s, 9H, OtBu); ^13^C NMR (CD_3_OD, 100 MHz) δ 169.8 (C=O), 157.2 (C=O), 136.5, 132.5, 130.8, 130.4, 79.4 (OC(Me)_3_), 50.5, 45.1, 43.3, 27.3 (C(*C*H_3_)_3_), 11.2 (CH_3_); Anal. Calcd for C_16_H_25_N_3_O_7_S_3_: C, 41.10; H, 5.39; N, 8.99. Found: C, 39.89; H, 5.30; N, 9.34.
*tert-Butyl-N-[({2-methyl-5-(morpholine-4-sulfonyl)thiophen-3-yl}carbamoyl)methyl] carbamate* (**15**). Off-white solid (0.370 g, 86%). mp = 124–128 °C; TLC R_f_ = 0.21 (50% EA/hexanes); IR (ATR) 3623 (NH), 3313 (NH), 2976 (CH), 2859 CH), 1681 (C=O), 1348 (SO_2_), 1152 (SO_2_) cm^−1^; ^1^H NMR (CDCl_3_, 400 MHz) δ 8.48 (bs, 1H, NH), 7.92 (s, 1H, ArH), 5.27 (bs, 1H, NH), 3.91 (d, *J* = 4.2 Hz, 2H), 3.77 (t, *J =* 4.2 Hz, 4H, O-CH_2_-), 3.06 (t, *J* = 4.2 Hz, 4H, N-CH_2_-), 2.38 (s, 3H, CH_3_), 1.49 (s, 9H, OtBu); ^13^C NMR (CDCl_3_, 100 MHz) δ 167.9 (C=O), 156.8 (C=O), 132.7, 132.1, 130.8, 128.5, 80.8 (OC(Me)_3_), 47.8, 45.0, 28.2 (C(*C*H_3_)_3_), 27.1, 12.3 (CH_3_); Anal. Calcd for C_16_H_25_N_3_O_6_S_2_: C, 45.81; H, 6.01; N, 10.02. Found: C, 45.61; H, 6.04; N, 9.73.
*tert-Butyl-N-[({2-methyl-5-(pyrrolidine-1-sulfonyl)thiophen-3-yl}carbamoyl)methyl] carbamate* (**16**). Tan solid (0.280 g, 64%). mp = 63–67 °C; TLC R_f_ = 0.24 (50% EA/hexanes); IR (ATR) 3317 (NH), 2975 (CH), 2873 (CH), 1679 (C=O), 1344 (SO_2_), 1147 (SO_2_) cm^−1^; ^1^H NMR (CD_3_OD, 400 MHz) δ 7.67 (s, 1H, ArH), 3.87 (s, 2H, -CH_2_NBoc), 3.24–3.28 (m, 4H, N-CH_2_-), 2.39 (s, 3H, CH_3_), 1.77–1.80 (m, 4H, CH_2_), 1.47 (s, 9H, OtBu); ^13^C NMR (CDCl_3_, 100 MHz) δ 171.1 (C=O), 158.6 (C=O), 136.0, 135.8, 133.2, 130.3, 80.8 (OC(Me)_3_), 44.7, 44.0, 28.7 (C(*C*H_3_)_3_), 14.8, 12.5 (CH_3_); Anal. Calcd for C_16_H_25_N_3_O_5_S_2_: C, 47.63; H, 6.25; N, 10.41. Found: C, 47.30; H, 5.93; N, 10.14.
*tert-butyl-N-[({2-methyl-5-(piperidine-1-sulfonyl)thiophen-3-yl}carbamoyl) methyl]carbamate* (**17**). Yellow solid (0.220 g, 47%). mp = 156–160 °C; TLC R_f_ = 0.30 (50% EA/hexanes); IR (ATR) 3321 (NH), 2935 (CH), 2856 (CH), 1685 (C=O), 1676 (C=O), 1359 (SO_2_), 1142 (SO_2_) cm^−1^; ^1^H NMR (acetone-*d_6_*, 400 MHz) δ 9.01 (s, 1H, NH), 7.82 (s, 1H, ArH), 6.37 (s, 1H, NH), 3.90 (d, *J =* 5.8 Hz, 2H, -CH_2_NBoc), 3.00 (t, *J* = 5.4 Hz, 4H, N-CH_2_-), 2.40 (s, 3H, CH_3_), 1.65 (m, 4H), 1.44–1.49 (m, 11H); ^13^C NMR (acetone-*d_6_*, 100 MHz) δ 167.8 (C=O), 156.3 (C=O), 133.1, 132.0, 130.5, 128.8, 78.7 (OC(Me)_3_), 46.9, 44.1, 27.7, 25.0, 23.1, 11.6 (CH_3_); Anal. Calcd for C_17_H_27_N_3_O_5_S_2_: C, 48.90; H, 6.52; N, 10.06. Found: C, 48.63; H, 6.29; N, 10.03.
*tert-Butyl-N-[({5-(dimethylsulfamoyl)-2-methylthiophen-3-yl}carbamoyl)methyl] carbamate* (**18**). Orange powder (0.230 g, 67%). mp = 88–93 °C; TLC R_f_ = 0.20 (50% EA/hexanes); IR (ATR) 3314 (NH), 2974 (CH), 2928 (CH), 1677 (C=O), 1341 (SO_2_), 1142 (SO_2_) cm^−1^; ^1^H NMR (CD_3_OD, 400 MHz) δ 7.64 (s, 1H, ArH), 3.87 (bs, 2H, -CH_2_NBoc), 2.72 (s, 6H, N(CH_3_)_2_), 2.40 (s, 3H, CH_3_), 1.47 (s, 9H, OtBu); ^13^C NMR (CD_3_OD, 100 MHz) δ 169.7 (C=O), 165.3 (C=O), 135.6, 132.2, 129.8, 129.3, 78.1 (OC(Me)_3_), 43.3, 36.9, 27.3 (C(*C*H_3_)_3_), 11.2 (CH_3_); Anal. Calcd for C_14_H_23_N_3_O_5_S_2_: C, 44.55; H, 6.14; N, 11.13. Found: C, 44.67; H, 6.31; N, 10.88.
*tert-Butyl-N-[({5-(diethylsulfamoyl)-2-methylthiophen-3-yl}carbamoyl)methyl]carbamate* (**19**). The crude oil was purified via silica gel column chromatography using a 50% EA/hexanes eluent, yielding an orange solid (0.120 g, 49%). mp = 47–49 °C; TLC R_f_ = 0.31 (50% EA/hexanes); IR (ATR) 3309 (NH), 2978 (CH), 2935 (CH), 1679 (C=O), 1340 (SO_2_), 1115 (SO_2_) cm^−1^; ^1^H NMR (CD_3_OD, 400 MHz) δ 7.64 (s, 1H, ArH), 3.86 (bs, 2H, -CH_2_NBoc), 3.22 (q, *J =* 7.4 Hz, 4H, O_2_SNCH_2_-), 2.38 (s, 3H, ArCH_3_), 1.47 (s, 9H, OtBu), 1.17 (t, *J =* 7.2 Hz, 6H, CH_3_); ^13^C NMR (CD_3_OD, 100 MHz) δ 169.7 (C=O), 157.2 (C=O), 134.6, 134.4, 131.8, 128.9, 79.4 (OC(Me)_3_), 43.3, 42.6, 27.3 (C(*C*H_3_)_3_), 13.4 (ArCH_3_), 11.1 (CH_3_); Anal. Calcd for C_16_H_27_N_3_O_5_S_2_: C, 47.39; H, 6.71; N, 10.36. Found: C, 47.58; H, 6.42; N, 10.34.
*tert-Butyl-N-[({5-(butylsulfamoyl)-2-methylthiophen-3-yl}carbamoyl)methyl]carbamate* (**20**). Off-white foam (0.320 g, 70%). mp = 65–67 °C; TLC R_f_ = 0.33 (50% EA/hexanes); IR (ATR) 3657 (NH), 3217 (NH), 2980 (CH), 1676 (C=O), 1305 (SO_2_), 1146 (SO_2_) cm^−1^; ^1^H NMR (CDCl_3_, 400 MHz) δ 8.44 (bs, 1H, NH), 7.83 (bs, 1H, ArH), 5.44 (bs, 1H, NH), 4.97 (bs, 1H, NH), 3.91 (s, 2H, -CH_2_NBoc), 3.01 (t, *J =* 6.6 Hz, 2H, O_2_SNCH_2_-), 2.33 (s, 3H, ArH), 1.43–1.53 (m, 11H), 1.32 (sext, *J =* 7.0 Hz, 2H), 0.88 (t, *J =* 7.3 Hz, 3H, CH_3_); ^13^C NMR (CDCl_3_, 100 MHz) δ 167.9 (C=O), 156.9 (C=O), 135.2, 131.7, 128.0, 128.0, 81.0 (OC(Me)_3_), 45.3, 43.3, 31.5, 28.3 (C(*C*H_3_)_3_), 19.7, 13.6 (ArCH_3_), 12.4 (CH_3_); Anal. Calcd for C_9_H_27_N_3_O_5_S_2_: C, 47.39; H, 6.71; N, 10.36. Found: C, 47.43; H, 6.40; N, 10.49.
*2-Aminotrifluoroacetate-N-[2-methyl-5-(thiomorpholine-4-sulfonyl)thiophene-3-yl]acetamide* (**21**). Boc-protected amine **12** was dissolved in TFA and stirred at rt for 0.5 h. The solvent was then removed in vacuo to provide **21** as an off-white solid that was recovered (1.57 g, 96%). mp = 190 °C (dec); IR (ATR) 3255 (NH), 2980 (CH), 2915 (CH), 1665 (C=O), 1350 (SO_2_), 1153 (SO_2_) cm^−1^; ^1^H NMR (CD_3_OD, 400 MHz) δ 7.79 (s, 1H, ArH), 3.93 (s, 2H, -CH_2_N), 3.37 (t, *J* = 5.0 Hz, 4H, O_2_SNCH_2_-), 2.74 (t, *J* = 5.2, 4H, SCH_2_-), 2.45 (s, 3H, ArCH_3_); ^13^C NMR (CD_3_OD, 100 MHz) δ 164.5 (C=O), 134.7, 131.7, 131.4, 128.9, 40.4, 26.7, 11.1 (ArCH_3_). Anal. Calcd for C_13_H_18_F_3_N_3_O_5_S_3_: C, 34.74; H, 4.04; N, 9.35. Found: C, 34.97; H, 4.16; N, 8.96.
*2-(4-Fluorobenzenesulfonamido)-N-[2-methyl-5-(thiomorpholine-4-sulfonyl)thiophen-3-yl]acetamide K306* (**1**). Amine salt **21** (0.600 g, 1.384 mmol) was dissolved in dry DCM (2.15 mL) and dry TEA (0.425 mL, 3.045 mmol) and stirred at rt. 4-Fluorobenzenesulfonyl chloride (0.296 g, 1.523 mmol) was added and the mixture was stirred at rt for 24 h. The mixture was washed with water (3 × 5 mL) followed by a 5% HCl solution. The organics were collected, dried over MgSO_4_, filtered, and concentrated. The crude mixture was purified by precipitation from DCM, yielding **1** as a white powder (0.213 g, 31%). mp = 177–180 °C; TLC R_f_ = 0.37 (1% MeOH/DCM); IR (ATR) 3301 (NH), 3259 (NH), 2959 (CH), 2915 (CH), 1665 (C=O), 1352 (SO_2_), 1141 (SO_2_) cm^−1^; ^1^H NMR (DMSO, 400 MHz) δ 7.82 (dd, *J* = 8.5, 5.0 Hz, 2H, ArH), 7.60 (s, 1H, thiophene ArH), 7.36 (t, *J* = 8.7 Hz, 2H, ArH), 3.63 (s, 2H, O=C-CH_2_N), 3.19 (t, *J* = 4.4 Hz, 4H, O_2_SNCH_2_-), 2.68 (t, *J* = 4.4 Hz, 4H, SCH_2_-), 2.31 (s, 3H, ArCH_3_); ^13^C NMR (DMSO, 100 MHz) δ 167.7 (C=O), 163.8 (d, *J* = 247.9 Hz, ArC-F), 138.5, 133.4, 133.2, 130.1, 130.0, 129.9 (d, *J* = 9.2 Hz, Ar*C*-C-C-F), 116.4 (d, *J* = 22.5, Ar*C*-C-F), 48.3, 46.5, 26.8, 12.8 (CH_3_); Anal. Calcd for C_17_H_20_FN_3_O_5_S_4_: C, 41.37; H, 4.08; N, 8.51. Found: C, 41.66; H, 3.80; N, 8.87. HRMS calcd for C_17_H_20_FN_3_O_5_S_4_K (M+K^+^): 531.9901. Found: 531.9903.

### 3.4. General Procedure for the Synthesis of Sulfonamides ***22***–***29***

The Boc-protected amine was dissolved in TFA (0.5 M) and stirred at rt for 0.5 h. The solvent was then removed in vacuo to provide the amine TFA salt. This amine salt was dissolved in dry DCM (0.7 M) and dry TEA was added (2.2 equiv). The desired sulfonyl chloride or other electrophile (1.1 equiv) was then added and the mixture was stirred at rt for 24 h under argon. The reaction was then taken up in EA and washed with H_2_O and 5% HCl. The organic layer was then separated, dried over Na_2_SO_4_, filtered, and concentrated. The residue was then purified by trituration from DCM or by silica gel chromatography, yielding the pure product.

*2-(4-Fluorobenzenesulfonamido)-N-{2-methyl-5-[1-oxo-1-thiomorpholin-4-yl)sulfonyl]thiophen-3-yl} acetamide* (**22**). The crude oil was purified via trituration from DCM, yielding a white solid (0.021 g, 13%). mp = 210–212 °C; TLC R_f_ = 0.49 (95% DCM/5% MeOH); IR (ATR) 3360 (NH), 3267 (NH), 3106 (ArH), 2927 (CH), 2858 (CH), 1685 (C=O), 1670 (C=O), 1352, 1328 (SO_2_), 1154 (SO_2_) cm^−1^; ^1^H NMR (DMSO-*d_6_*, 400 MHz) δ 9.77 (s, 1H, NH), 8.17 (bs, 1H, NH), 7.89 (m, 2H, ArH), 7.65 (s, 1H, thiophene ArH), 7.44 (t, *J =* 8.4 Hz, 2H, ArH), 3.73 (d, *J =* 4.8 Hz, 2H), 3.60 (d, *J =* 12.4 Hz, 2H), 3.05–3.10 (m, 2H), 2.90–2.99 (m, 4H), 2.35 (s, 3H, CH_3_); ^13^C NMR (DMSO-*d_6_*, 100 MHz) δ 166.8 (C=O), 161.6 (d, *J* = 364 Hz, ArC-F), 137.2 (d, *J* = 3.0 Hz, Ar*C*-C-C-F), 134.5, 133.3, 130.1 (d, *J =* 9.7 Hz, Ar*C*-C-C-F), 129.9, 129.2, 116.7 (d, *J =*23.2 Hz, Ar*C*-C-F), 45.6, 43.9, 36.9, 12.9 (CH_3_); Anal. Calcd for C_17_H_20_FN_3_O_6_S_4_: C, 40.07; H, 3.96; N, 8.25. Found: C, 39.93; H, 4.24; N, 8.08.
*2-(4-Fluorobenzenesulfonamido)-N-[2-methyl-5-(morpholine-4-sulfonyl)thiophen-3-yl]acetamide* (**24**). White powder (0.104 g, 30%). mp = 173–177 °C; TLC R_f_ = 0.28 (50% EA/hexanes); IR (ATR) 3395 (NH), 3233 (NH), 2980 (CH), 2890 (Ch), 1673 (C=O), 1345 (SO_2_), 1153 (SO_2_) cm^−1^; ^1^H NMR (CD_3_OD, 400 MHz) δ 8.19 (s, 1H, NH), 7.91–7.94 (m, 2H, ArH), 7.77 (s, 1H, thiophene ArH), 7.23–7.28 (m, 2H, ArH), 5.45 (t, *J* = 5.9 Hz, 1H, NH), 3.72–3.78 (m, 6H), 3.02–3.06 (m, 4H, O_2_SNCH_2_-), 2.40 (s, 3H, CH_3_); ^13^C NMR (CD_3_OD, 100 MHz) δ 165.6 (d, *J* = 255.0 Hz, ArC-F), 165.5, 134.2, 133.9, 131.5, 130.1 (d, *J* = 9.9 Hz, Ar*C*-C-C-F), 129.8, 128.8, 116.8 (d, *J* = 23.1 Hz, Ar*C*-C-F), 66.0, 46.3, 46.0, 12.5 (CH_3_); Anal. Calcd for C_17_H_20_FN_3_O_6_S_3_: C, 42.76; H, 4.22; N, 8.80. Found: C, 42.82; H, 4.24; N, 8.52.
*2-(4-Fluorobenzenesulfonamido)-N-[2-methyl-5-(pyrrolidine-1-sulfonyl)thiophen-3-yl]acetamide* (**25**). White foam (0.020 g, 10%). mp = 159–163 °C; TLC R_f_ = 0.18 (50% EA/hexanes); IR (ATR) 3360 (NH), 3239 (NH), 3099 (CH), 2885 (CH), 1676 (C=O), 1328 (SO_2_), 1151 (SO_2_) cm^−1^; ^1^H NMR (CD_3_OD, 400 MHz) δ 7.94–7.97 (m, 2H, ArH), 7.55 (s, 1H thiophene ArH), 7.31 (t, *J* = 8.5 Hz, 2H, ArH), 3.77 (s, 2H), 3.25 (m, 3H), 2.35 (s, 3H, ArCH_3_), 1.79 (m, 4H); ^13^C NMR (CD_3_OD, 100 MHz) δ 167.8 (C=O), 165.2 (d, *J* = 251.6, ArC-F), 136.2 (d, *J* = 2.7 Hz, Ar*C*-C-C-F), 135.2, 131.7, 130.7, 129.9 (d, *J* = 9.5 Hz, Ar*C*-C-C-F), 129.3, 115.8 (d, *J* = 23.4 Hz, Ar*C*-C-F), 47.8, 45.1, 24.9, 11.1 (CH_3_); Anal. Calcd for C_17_H_20_FN_3_O_5_S_3_: C, 44.24; H, 4.37; N, 9.10. Found: C, 44.21; H, 4.44; N, 8.97.
*2-(4-Fluorobenzenesulfonamido)-N-[2-methyl-5-(piperidine-1-sulfonyl)thiophen-3-yl]acetamide* (**26**). The crude oil was purified via silica gel column chromatography using a 50% EA/hexanes eluent, yielding a white solid (0.092 g, 37%). mp = 79–84 °C; TLC R_f_ = 0.21 (50% EA/hexanes); IR (ATR) 3300 (NH), 2939 (CH), 2853 (CH), 1686 (C=O), 1589 (C=C), 1335 (SO_2_), 1154 (SO_2_), 1143 (SO_2_) cm^−1^; ^1^H NMR (CD_3_OD, 400 MHz) δ 7.95 (m, 2H, ArH), 7.48 (s, 1H, thiophene ArH), 7.31 (t, *J =* 8.7 Hz, 2H, ArH), 3.77 (s, 2H, OC-CH_2_-N), 3.00 (t, *J =* 5.2 Hz, 4H, N-CH_2_-), 2.35 (s, 3H, CH_3_), 1.62–1.69 (m, 4H), 1.47–1.50 (m, 2H); ^13^C NMR (CD_3_OD, 100 MHz) δ 166.7 (C=O), 164.6 (d, *J* = 250.4 Hz, ArC-F), 137.3 (d, *J* = 2.9 Hz, Ar*C*-C-C-F), 133.5, 133.1, 130.1 (d, *J* = 9.4 Hz, Ar*C*-C-C-F), 129.7, 129.4, 116.6 (d, *J* = 23.1 Hz, Ar*C*-C-F), 47.1, 45.6, 25.0, 23.2, 12.9 (CH_3_); Anal. Calcd for C_18_H_22_FN_3_O_5_S_3_: C, 45.46; H, 4.66; N, 8.84. Found: C, 45.47; H, 4.92; N, 8.99.
*N-[5-(Dimethylsulfamoyl)-2-methylthiophen-3-yl]-2-(4-fluorobenzenesulfonamido)acetamide* (**27**). White powder (0.120 g, 51%). mp = 123–128 °C; TLC R_f_ = 0.21 (50% EA/hexanes); IR (ATR) 3321 (NH), 3278 (NH), 3107 (ArCH), 2928 (CH), 1658 (C=O), 1590 (C=C), 1342 (SO_2_), 1155 (SO_2_), 1137 (SO_2_) cm^−1^; ^1^H NMR (CD_3_OD, 400 MHz) δ 7.93–7.97 (m, 2H, ArH), 7.52 (s, 1H thiophene ArH), 7.31 (t, *J* = 8.1 Hz, 2H, ArH), 3.77 (s, 2H, OC-CH_2_-N), 2.71 (s, 6H, N(CH_3_)_2_), 2.36 (s, 3H, CH_3_); ^13^C NMR (CD_3_OD, 100 MHz) δ 167.8 (C=O), 165.2 (d, *J* = 251.5), 136.2 (d, *J* = 3.1 Hz, Ar*C*-C-C-F), 135.6, 131.8, 129.9 (d, *J* = 9.4 Hz, Ar*C*-C-C-F), 129.6, 129.4, 115.8 (d, *J* = 24.3 Hz, Ar*C*-C-F), 45.1, 36.9, 11.1 (CH_3_); Anal. Calcd for C_15_H_18_FN_3_O_5_S_3_: C, 41.37; H, 4.17; N, 9.65. Found: C, 41.24; H, 4.28; N, 9.36.
*N-[5-(Diethylsulfamoyl)-2-methylthiophen-3-yl]-2-(4-fluorobenzenesulfonamido)acetamide* (**28**). Orange wax (0.108 g, 80%). mp = 44–48 °C; TLC R_f_ = 0.18 (50% EA/hexanes); IR (ATR) 3309 (NH), 2977 (CH), 2934 (CH), 1685 (C=O), 1590 (C=C), 1327 (SO_2_), 1141 (SO_2_) cm^−1^; ^1^H NMR (CD_3_OD, 400 MHz) δ 7.95 (m, 2H, ArH), 7.52 (s, 1H thiophene ArH), 7.30 (t, *J =* 8.6 Hz, 2H, ArH), 3.76 (s, 2H, OC-CH_2_-N), 3.20 (m, 4H, NCH_2_-), 2.33 (s, 3H), 1.16 (t, *J =* 7.2 Hz, 6H, CH_3_); ^13^C NMR (CD_3_OD, 100 MHz) δ 167.7, 165.0 (d, *J =* 250.1 Hz, ArC-F), 136.2 (d, *J* = 2.5 Hz, Ar*C*-C-C-F), 134.6, 134.5, 131.5, 129.9 (d, *J* = 9.4 Hz, Ar*C*-C-C-F), 128.6, 115.8 (d, *J* = 23.8 Hz, Ar*C*-C-F), 45.1, 42.6, 13.4 (CH_3_), 11.1 (CH_3_); Anal. Calcd for C_17_H_22_FN_3_O_5_S_3_: C, 44.05; H, 4.78; N, 9.06. Found: C, 44.12; H, 4.99; N, 8.79.
*N-[5-(Butylsulfamoyl)-2-methylthiophen-3-yl]-2-(4-fluorobenzenesulfonamido)acetamide* (**29**). White powder (0.185 g, 13%). mp = 161–164 °C; TLC R_f_ = 0.19 (50% EA/50% hexane); IR (ATR) 3247, 2964, 2934, 2876, 1652, 1325 (SO_2_), 1151 (SO_2_) cm^−1^; ^1^H NMR (CD_3_OD, 400 MHz) δ 7.94–8.00 (m, 2H, ArH), 7.56 (s, 1H, thiophene ArH), 7.30–7.35 (m, 2H, ArH), 3.78 (s, 2H, OC-CH_2_-N), 2.93 (t, *J =* 7.0 Hz, 2H, N-CH_2_), 2.36 (s, 3H, CH_3_), 1.48 (pent, *J =* 6.9 Hz, 2H), 1.35 (sext, *J =* 7.0 Hz, 2H), 0.91 (t, *J =* 7.2, 3H, CH_3_); ^13^C NMR (CD_3_OD, 100 MHz) δ 167.8, 165.2 (d, *J* = 251.4 Hz, ArC-F), 136.2 (d, *J* = 3.4 Hz, Ar*C*-C-C-F), 135.8, 134.6, 131.2, 129.8 (d, *J* = 9.3 Hz, Ar*C*-C-C-F), 128.6, 115.8 (d, *J* = 23.1 Hz, Ar*C*-C-F), 45.1, 42.6, 31.2, 19.4, 12.5 (CH_3_), 11.1 (CH_3_); Anal. Calcd for C_17_H_22_FN_3_O_5_S_3_: C, 44.05; H, 4.78; N, 9.06. Found: C, 44.10; H, 4.70; N, 9.05.
*2-Chloro-5-(1,4-thiazinan-4-ylsulfonyl)thien-3-ylamine* (**31**). Thiomorpholine (0.086 mL, 0.859 mmol) and DIPEA (0.149 mL, 0.859 mmol) were dissolved in dry DCM (11.8 mL, 0.08M) and cooled to 0 °C. 2-Chloro-3-nitrothiophene-5-sulfonyl chloride (0.250 g, 0.954 mmol) was added dropwise and the mixture was heated to rt and stirred for 1hr until TLC indicated reaction was completed. The mixture was diluted in DCM and washed with sat. NaHCO_3_ followed by brine and the organics were dried over Na_2_SO_4_, filtered, and concentrated. The crude mixture was purified via silica gel chromatography using a 20% EA/hexanes eluent to yield 4-[(5-chloro-4-nitrothiophen-2-yl)sulfonyl]thiomorpholine (0.157 g, 50%). mp = 139–143 °C; TLC R_f_ = 0.50 (20% EA/hexanes); IR (ATR) 3106 (NH), 2972 (CH), 2906 (CH), 1531 (C=C), 1358 (SO_2_), 1152 (SO_2_) cm^−1^; ^1^H NMR (acetone-*d_6_*, 400 MHz) δ 8.06 (s, 1H, ArH), 3.50 (t, *J* = 4.0 Hz, 4H, O_2_SNCH_2_-), 2.79 (t, *J* = 5.1 Hz, 4H, S-CH_2_-); ^13^C NMR (DMSO-*d_6_*, 100 MHz) δ 143.9, 137.4, 133.2, 128.2, 48.2 (O_2_SNCH_2_-), 26.8 (S-CH_2_-); Anal. Calcd for C_8_H_9_ClN_2_O_4_S_3_: C, 29.22; H, 2.76; N, 8.52. Found: C, 29.42; H, 2.40; N, 8.35. 4-[(5-chloro-4-nitrothiophen-2-yl)sulfonyl]thiomorpholine (0.67 g, 2.04 mmol) was dissolved in acetic acid (6.8 mL, 0.3M) and iron powder (0.57 g, 10.19 mmol) was added. The mixture was heated to 60 °C and stirred for 1 h, after which the acetic acid was removed in vacuo. The residue was dissolved in EA and washed with saturated aq. NaHCO_3_. The organic layer was then washed with brine, dried over MgSO_4_, filtered, and concentrated. The residue was purified via silica gel chromatography using a 50% EA/hexanes eluent, yielding **31** (0.420 g, 69%) as a yellow powder. (**31**) mp = 138–140 °C; TLC R_f_ = 0.58 (50% EA/hexanes); IR (ATR) 3448 (NH), 3360 (NH), 2903 (CH), 2852 (CH), 1611 (C=C), 1334 (SO_2_), 1149 (SO_2_) cm^−1^; ^1^H NMR (acetone-*d_6_*, 400 MHz) δ 7.16 (s, 1H, ArH), 5.00 (bs, 2H, NH2), 3.36 (t, *J* = 4.9 Hz, 4H, O_2_SNCH_2_-), 2.77 (t, *J* = 5.3 Hz, 4H, S-CH_2_-); ^13^C NMR (DMSO-*d_6_*, 100 MHz) δ 143.9, 131.6, 124.5, 106.7, 48.1 (O_2_SNCH_2_-), 26.8 (S-CH_2_-); Anal. Calcd for C_8_H_11_ClN_2_O_2_S_3_: C, 32.16; H, 3.71; N, 9.37. Found: C, 32.31; H, 3.40; N, 9.11.
*tert-Butyl-N-({[2-chloro-5-(thiomorpholine-4-sulfonyl)thiophen-3-yl]carbamoyl}methyl) carbamate* (**32**). Boc-Glycine (0.300 g, 1.69 mmol), aminothiophene **31** (0.420 g, 1.41 mmol), HATU (1.069 g, 2.81 mmol), and DIPEA (0.49 mL, 2.81) were dissolved in 7 mL of dry DMA under argon. The mixture was stirred for 24 h at rt. The reaction was then diluted with EA and washed with sat aq. NH_4_Cl (3×) and 5 % aq. LiCl (3 × 15 mL). The organic layer was dried over MgSO_4_, filtered, and concentrated. The residue was purified via silica gel chromatography using 50% EA/hexanes, yielding **32,** a yellow solid (0.400g, 63%). mp = 143–148 °C; TLC R_f_ = 0.36 (50% EA/hexanes); IR (ATR) 3297 (NH), 2982 (CH), 2915 (CH), 1693 (C=O), 1147 (SO_2_) cm^−1^; ^1^H NMR (DMSO-*d_6_*, 400 MHz) δ 10.01 (s, 1H, NH), 7.93 (s, 1H, ArH), 7.12 (t, *J* = 6.1 Hz, 1H, NH), 3.80 (d, *J* = 5.7 Hz, 2H, OC-CH_2_-N), 3.29 (t, *J* = 4.4 Hz, 4H, O_2_SNCH_2_-), 2.72 (t, *J* = 5.0, 4H, S-CH_2_-), 1.40 (s, 9H, C(CH_3_)_3_); ^13^C NMR (DMSO-*d_6_*, 100 MHz) δ 169.2 (C=O), 156.4 (C=O), 134.8, 131.9, 128.4, 120.8, 78.7 (OC(Me)_3_), 48.3, 43.7, 28.7 (C(*C*H_3_)_3_), 26.8. Anal. Calcd for C_15_H_22_ClN_3_O_5_S_3_: C, 39.51; H, 4.86; N, 9.22. Found: C, 39.73; H, 4.62; N, 9.07.
*N-[2-Chloro-5-(thiomorpholine-4-sulfonyl)thiophen-3-yl]-2-(4-fluorobenzenesulfonamido) acetamide* (**33**). Carbamate **32** (0.250 g, 0.532 mmol) was dissolved in 2 mL of TFA and stirred at rt for 0.5 h. The solvent was removed in vacuo and the resulting off-white solid was dissolved in dry DCM (0.829 mL, 0.6M). Dry TEA (0.163 mL, 1.170 mmol) was added followed by 4-fluorobenzenesulfonyl chloride (0.114 g, 0.585 mmol). After 24 h at rt, the reaction mixture was diluted with EA and washed with water (3 × 5 mL) and 5% aq. HCl solution. The organic layer was dried over MgSO_4_, filtered, and concentrated. The residue was purified by precipitation from DCM, yielding **33** as a white powder (0.032 g, 12%). mp = 173–178 °C; TLC R_f_ = 0.24 (50% EA/hexanes); IR (ATR) 3274 (NH), 2917 (CH), 1664 (C=O), 1576 (C=C), 1332 (SO_2_), 1141 (SO_2_) cm^−1^; ^1^H NMR (CD_3_OD, 400 MHz) δ 7.93–7.96 (m, 2H, ArH), 7.80 (s, 1H, thiophene ArH), 7.27–7.32 (m, 2H, ArH), 3.84 (s, 2H, OC-CH_2_-N), 3.36 (t, *J* = 4.9, 4H, O_2_SNCH_2_-), 2.73 (t, *J* = 4.9 Hz, 4H, S-CH_2_-); ^13^C NMR (CD_3_OD, 100 MHz) δ 167.4 (C=O), 165.2 (d, *J =*252.9 Hz, ArC-F), 136.3 (d, *J* = 3.1 Hz, Ar*C*-C-C-F), 133.3, 132.7, 129.9 (d, *J =* 10.7 Hz, Ar*C*-C-F), 127.3, 121.7, 115.8 (d, *J* = 21.4 Hz, Ar*C*-C-F), 45.0, 29.3, 26.7; Anal. Calcd for C_16_H_17_FN_3_O_5_S_4_: C, 37.39; H, 3.33; N, 8.18. Found: C, 37.51; H, 3.02; N, 8.12.
*3-(1,4-Thiazinan-4-ylsulfonyl)aniline* (**35**). Thiomorpholine (2.00 mL, 19.85 mmol) and 3-nitrobenzenesulfonyl chloride (2.00 g, 9.02 mmol) were dissolved in 8.75 mL of 1,4-dioxane and heated to 60 °C. The mixture was stirred for 1 h at 60 °C, after which the reaction was cooled to rt and 20 mL of water was added. The mixture was extracted with DCM (3 × 20 mL), and the organics were dried over MgSO_4_, filtered, and concentrated. The residue was purified via silica gel chromatography with 20% EA/hexanes, yielding 4-(3-nitrophenylsulfonyl)-1,4-thiazinane as an off-white solid (1.21g, 47%). mp = 151–155 °C; TLC R_f_ = 0.33 (20% EA/hexanes); IR (ATR) 3104 (NH), 3073 (CH), 2918 (CH), 2860 (CH), 1531 (C=C), 1356 (SO_2_), 1341 (SO_2_) cm^−1^; ^1^H NMR (CDCl_3_, 400 MHz) δ 8.58 (t, *J* = 1.8 Hz, 1H, ArH), 8.47 (ddd, *J* = 8.3, 2.0, 0.8 Hz, 1H, ArH), 8.07 (dt, *J* = 7.8, 0.8 Hz, 1H, ArH), 7.78 (t, *J* = 8.1 Hz, 1H, ArH), 3.42 (t, *J* = 4.5 Hz, 4H, O_2_SNCH_2_-), 2.74 (t, *J* = 4.8, 4H, S-CH_2_-); ^13^C NMR (CDCl_3_, 100 MHz) δ 148.5, 139.5, 132.8, 130.7, 127.4, 122.4, 47.9, 27.3. Anal. Calcd for C_10_H_12_N_2_O_4_S_2_: C, 41.66; H, 4.20; N, 9.72. Found: C, 41.68; H, 4.46; N, 9.54. The 4-(3-nitrophenylsulfonyl)-1,4-thiazinane (1.20 g, 4.16 mmol) was dissolved in acetic acid (13.9 mL, 0.3M) and iron powder (1.16 g, 20.81 mmol) was added. The mixture was heated to 60 °C and stirred for 1 h, after which the acetic acid was removed in vacuo. The residue was dissolved in EA and washed with saturated NaHCO_3_ until a pH of 8 was reached. The organics were washed with brine, dried over MgSO_4_, filtered, and concentrated. This gave 3-(1,4-thiazinan-4-ylsulfonyl)aniline (**35**) as an off-white solid (0.750 g, 69%). (**35**) mp = 150–152 °C; TLC R_f_ = 0.43 (20% EA/hexanes); IR (ATR) 3467 (NH), 3375 (NH), 2907 (CH), 2859 (CH), 1619 (C=C), 1313 (SO_2_), 1151 (SO_2_) cm^−1^; ^1^H NMR (DMSO-*d_6_*, 400 MHz) δ 7.25 (t, *J* = 7.9 Hz, 1H, ArH), 6.91 (t, *J* = 1.9 Hz, 1H, ArH), 6.82–6.84 (m, 1H, ArH), 6.78–6.81 (m, 1H, ArH), 5.65 (bs, 2H, NH_2_), 3.17 (t, *J* = 4.9 Hz, 4H, O_2_SNCH_2_-), 2.67 (t, *J* = 5.1, 4H, S-CH_2_-); ^13^C NMR (DMSO-*d_6_*, 100 MHz) δ 150.1, 136.9, 130.3, 118.4, 114.2, 111.8, 48.3, 26.9. Anal. Calcd for C_10_H_14_N_2_O_2_S_2_: C, 46.49; H, 5.46; N, 10.84. Found: C, 46.63; H, 5.17; N, 10.96.
*tert-Butyl-N-([{3-(thiomorpholine-4-sulfonyl)phenyl]carbamoyl}methyl)carbamate* (**36**). Boc-Glycine (0.244 g, 1.39 mmol), aniline **35** (0.300 g, 1.16 mmol), HATU (0.883 g, 2.32 mmol) and DIPEA (0.59 mL, 2.32) were dissolved in 7 mL of dry DMA under argon. The mixture was stirred for 24 h at rt. The reaction was diluted with EA and washed with sat aq. NH_4_Cl solution (3x) followed by washing with 5% aq. LiCl (3 × 15 mL). The organic layer was dried (MgSO_4_), filtered, and concentrated. The residue was purified via silica gel chromatography (50% EA/hexanes), yielding amide **36,** a white solid (0.230 g, 48%). mp = 169–173 °C; TLC R_f_ = 0.44 (50% EA/hexanes); IR (ATR) 3436 (NH), 3267 (NH), 2980 (CH), 1702 (C=O), 1673 (C=O), 1349 (SO_2_), 1163 (SO_2_) cm^−1^; ^1^H NMR (CD_3_OD, 400 MHz) δ 8.14 (bs, 1H, ArH), 7.77 (d, *J* = 8.0 Hz, 1H, ArH), 7.55 (t, *J* = 8.0 Hz, 1H, ArH), 7.48 (d, *J* = 7.9 Hz, 1H, ArH), 3.88 (s, 2H, OC-CH_2_-N), 3.32–3.35 (m, 4H, O_2_SNCH_2_-), 2.68 (t, *J* = 5.1 Hz, 4H, S-CH_2_-), 1.47 (s, 9H, OtBu); ^13^C NMR (CD_3_OD, 100 MHz) δ 169.4 (C=O), 156.4 (C=O), 140.3, 137.1, 130.6, 123.7, 122.1, 117.6, 78.6 (O*C*(Me)_3_), 48.3, 44.3, 28.7, 26.8. Anal. Calcd for C_17_H_25_N_3_O_5_S_2_: C, 49.14; H, 6.06; N, 10.11. Found: C, 49.06; H, 6.35; N, 9.76.
*2-(4-Fluorobenzenesulfonamido)-N-[3-(thiomorpholine-4-sulfonyl)phenyl]acetamide* (**37**). Carbamate **36** (0.20 g, 0.466 mmol) was dissolved in 2 mL of TFA and stirred at rt for 0.5 h. The solvent was then removed in vacuo and the resulting off-white solid was dissolved in dry DCM (0.700 mL, 0.6 M). Dry TEA (0.143 mL, 1.025 mmol) and 4-fluorobenzenesulfonyl chloride (0.100 g, 0.513 mmol) were added and the mixture was stirred at rt for 24 h. The reaction mixture was then diluted with EA and washed with water (3 × 5 mL) and 5% aq. HCl. The organic layer was dried over MgSO_4_, filtered, and concentrated. The residue was purified by precipitation from DCM, yielding **37** as a white powder (0.059 g, 27%). mp = 143–148 °C; TLC R_f_ = 0.44 (50% EA/hexanes); IR (ATR) 3318 (NH), 3221 (NH), 3137 (CH), 2918 (CH), 1697 (C=O), 1590 (C=C), 1314 (SO_2_), 1148 (SO_2_) cm^−1^; ^1^H NMR (acetone-*d_6_*, 400 MHz) δ 9.57 (s, 1H, NH), 8.11 (s, 1H, ArH), 7.97–8.01 (m, 2H, ArH), 7.83 (dd, *J* = 8.2, 0.9 Hz, 1H, ArH), 7.57 (t, *J* = 7.8 Hz, 1H, ArH), 7.47 (d, *J* = 7.8 Hz, 1H, ArH), 7.33–7.37 (m, 2H, ArH), 6.99 (bs, 1H, NH), 3.86 (d, *J* = 5.9 Hz, 2H, OC-CH_2_-N), 3.30 (t, *J =* 4.9 Hz, 4H, O_2_SNCH_2_-), 2.70 (t, *J* = 4.0, 4H, S-CH_2_-); ^13^C NMR (acetone-*d_6_*, 100 MHz) δ 166.7 (C=O), 165.0 (d, *J* = 250.0 Hz, ArC-F), 139.4, 137.7, 136.6 (d, *J* = 3.1 Hz, Ar*C*-C-C-F), 130.1 (d, *J =* 10.7 Hz, Ar*C*-C-F), 129.8, 123.4, 122.4, 118.0, 116.1 (d, *J* = 23.1 Hz, Ar*C*-C-F), 48.1, 46.3, 26.8 (S-CH_2_-). Anal. Calcd for C_18_H_20_FN_3_O_5_S_3_: C, 45.65; H, 4.26; N, 8.87. Found: C, 45.43; H, 4.30; N, 8.80.
*4-(1,4-Thiazinan-4-ylsulfonyl)aniline* (**39**). Thiomorpholine (2.00 mL, 19.85 mmol) and 4-nitrobenzenesulfonyl chloride (2.00 g, 9.02 mmol) were dissolved in 8.75 mL of 1,4-dioxane and heated to 60 °C. The mixture was stirred for 1 h at 60 °C, after which the reaction was cooled to rt and 20 mL of water was added. The mixture was extracted with DCM (3 × 20 mL), and the organics were dried over MgSO_4_, filtered, and concentrated. The crude mixture was purified via precipitation from DCM and yielded 4-(4-nitrophenylsulfonyl)-1,4-thiazinane, a white solid (1.72 g, 66%). mp = 146–150 °C; TLC R_f_ = 0.41 (20% EA/hexanes); IR (ATR) 3103 (CH), 2924 (CH), 2871 (CH), 1527 (C=C), 1348 (SO_2_), 1160 (SO_2_) cm^−1^; ^1^H NMR (CDCl_3_, 400 MHz) δ 8.39 (d, *J* = 8.6 Hz, 2H, ArH), 7.93 (d, *J* = 8.8 Hz, 2H, ArH), 3.31 (t, *J* = 4.6 Hz, 4H, O_2_SNCH_2_-), 2.72 (t, *J* = 4.6, 4H, S-CH_2_-); ^13^C NMR (CDCl_3_, 100 MHz) δ 150.2, 143.1, 128.5, 124.5, 47.9 (O_2_SNCH_2_-), 27.3 (S-CH_2_-). Anal. Calcd for C_10_H_12_N_2_O_4_S_2_: C, 41.66; H, 4.20; N, 9.72. Found: C, 41.92; H, 4.01; N, 9.86. The 4-(4-nitrophenylsulfonyl)-1,4-thiazinane (1.70 g, 5.90 mmol) was dissolved in acetic acid (19.7 mL, 0.3 M) and iron powder (1.65 g, 29.48 mmol) was added. The mixture was heated to 60 °C and stirred for 1 h, after which the acetic acid was removed via vacuo. The residue was dissolved in EA and washed with saturated NaHCO_3_ until a pH of 8 was reached. The organics were washed with brine, dried over MgSO_4_, filtered, and concentrated. This gave aniline **39** as an off-white solid that was used without further purification (1.17g, 77%). (**39**) mp = 176–179 °C; TLC R_f_ = 0.53 (20% EA/hexanes); IR (ATR) 3445 (NH), 3358 (NH), 3144 (CH), 2844 (CH), 1594 (C=C), 1313 (SO_2_), 1148 (SO_2_) cm^−1^; ^1^H NMR (acetone-*d_6_*, 400 MHz) δ 7.74 (d, *J* = 8.8 Hz, 2H, ArH), 6.79 (d, *J* = 8.8 Hz, 2H, ArH), 5.55 (bs, 2H, NH_2_), 3.21 (t, *J* = 5.0 Hz, 4H, O_2_SNCH_2_-), 2.68 (t, *J* = 5.0, 4H, S-CH_2_-); ^13^C NMR (acetone-*d_6_*, 100 MHz) δ 152.9, 129.5, 122.8, 113.2, 48.1 (O_2_SNCH_2_-), 26.9 (S-CH_2_-). Anal. Calcd for C_10_H_14_N_2_O_2_S_2_: C, 46.49; H, 5.46; N, 10.84. Found: C, 46.73; H, 5.64; N, 10.68.
*tert*-*Butyl*-*N-([{4-(thiomorpholine-4-sulfonyl)phenyl]carbamoyl}methyl)carbamate* (**40**). Boc-Glycine (0.408 g, 2.32 mmol), aniline **39** (0.500 g, 1.94 mmol), HATU (1.472 g, 3.87 mmol) and DIPEA (0.99 mL, 3.87) were dissolved in 9.7 mL of dry DMA under argon. The mixture was stirred for 24 h at rt. The reaction mixture was then diluted with EA and washed with sat aq. NH_4_Cl solution (3×) and 5% aq. LiCl (3 × 15 mL). The organic layer was then dried over MgSO_4_, filtered, and concentrated. The residue was purified via silica gel chromatography using 50% EA/hexanes, yielding carbamate **40** as a white powder (0.170 g, 21%). mp = 180–182 °C; TLC R_f_ = 0.31 (50% EA/hexanes); IR (ATR) 3369 (NH), 3280 (NH), 3114 (CH), 2977 (CH), 2853 (CH), 1675 (C=O), 1305 (SO_2_), 1158 (SO_2_) cm^−1^; ^1^H NMR (CD_3_OD, 400 MHz) δ 7.84 (d, *J* = 8.5 Hz, 2H, ArH), 7.73 (d, *J* = 8.5 Hz, 2H, ArH), 3.91 (s, 2H, OC-CH_2-_NBoc), 3.29–3.33 (m, 4H, O_2_SNCH_2_-), 2.70 (t, *J* = 5.0, 4H, S-CH_2_-), 1.49 (s, 9H, OtBu); ^13^C NMR (DMSO-*d_6_*, 100 MHz) δ 169.6 (C=O), 156.4 (C=O), 143.6, 130.1, 129.0, 119.4, 78.6 (OC(Me)_3_), 48.2, 44.4, 28.7, 26.8. Anal. Calcd for C_17_H_25_N_3_O_5_S_2_: C, 49.14; H, 6.06; N, 10.11. Found: C, 49.38; H, 6.32; N, 10.48.
*2-(4-Fluorobenzenesulfonamido)-N-[4-(thiomorpholine-4-sulfonyl)phenyl]acetamide* (**41**). Carbamate **40** (0.170g, 0.396 mmol) was dissolved in 2 mL of TFA and stirred at rt for 0.5 h. The solvent was then removed in vacuo and the resulting off-white solid was dissolved in dry DCM (0.595 mL, 0.6M). Dry TEA (0.121 mL, 0.871 mmol) and 4-fluorobenzenesulfonyl chloride (0.085 g, 0.436 mmol) were added and the mixture was stirred at rt for 24 h. The reaction was then diluted with EA and washed with water (3 × 5 mL) and 5% aq. HCl. The organic layer was dried over MgSO_4_, filtered, and concentrated. The residue was purified by precipitation from DCM, yielding **41** as an off-white solid (0.068 g, 36%). mp = 168–173 °C; TLC R_f_ = 0.39 (50% EA/hexanes); IR (ATR) 3326 (NH), 3288 (NH), 3109 (CH), 2980 (CH), 2888 (CH), 1700 (C=O), 1540 (C=C), 1317 (SO_2_), 1090 (SO_2_) cm^−1^; ^1^H NMR (acetone-*d_6_*, 400 MHz) δ 9.61 (s, 1H, NH), 7.97–8.00 (m, 2H, ArH), 7.83 (d, *J* = 8.6 Hz, 2H, ArH), 7.70 (d, *J* = 8.6 Hz, 2H, ArH), 7.32–7.37 (m, 2H, ArH), 6.99 (bs, 1H, NH), 3.88 (d, *J* = 5.6 Hz, 2H, OC-CH_2-_N), 3.28 (t, *J =* 4.7 Hz, 4H, O_2_SNCH_2_-), 2.69 (t, *J* = 5.0, 4H, S-CH_2_-); ^13^C NMR (acetone-*d_6_*, 100 MHz) δ 166.8 (C=O), 165.0 (d, *J* = 254.8 Hz, ArC-F), 142.6, 136.7 (d, *J* = 3.4 Hz), 131.5, 130.1 (d, *J =* 10.0 Hz, Ar*C*-C-C-F), 128.6, 119.2, 116.1 (d, *J* = 23.1 Hz, Ar*C*-C-C-F), 48.1, 46.3, 26.8 (S-CH_2_-). Anal. Calcd for C_18_H_20_FN_3_O_5_S_3_: C, 45.65; H, 4.26; N, 8.87. Found: C, 45.34; H, 4.23; N, 8.66.

### 3.5. General Procedure for the Synthesis of Sulfonamides ***48***–***53***

Aminothiophene **3** (1 equiv), the Boc-protected amino acid (1.2 equiv), HATU (2 equiv), and DIPEA (2 equiv, 0.59 mL, 2.32) were dissolved in dry DMA (0.2 M) under argon. The mixture was stirred for 24 h at rt. The reaction was diluted with EA and washed with sat aq. NH_4_Cl solution (3×) followed by washing with 5% aq. LiCl (3 × 15 mL). The organic layer was then dried over MgSO_4_, filtered, and concentrated. The residue was purified via silica gel chromatography. The Boc-protected amine was then dissolved in TFA and stirred at rt for 0.5 h. The solvent was then removed in vacuo to provide the amine TFA salt. This amine salt was suspended in dry DCM (0.3 M) and dry TEA was added (2.2 equiv). 4-Fluorobenzenesulfonyl chloride(1.1 equiv) was then added and the mixture was stirred at rt for 24 h under argon. The reaction was then taken up in EA and washed with H_2_O and 5% HCl. The organic layer was then dried over Na_2_SO_4_, filtered, and concentrated. The residue was purified by trituration from DCM or by silica gel chromatography, yielding the pure product.

*(2S)-2-(4-Fluorobenzenesulfonamido)-N-[2-methyl-5-(thiomorpholine-4-sulfonyl)thiophen-3-yl] propanamide* (**48**). White powder (0.118 g, 21% over 3 steps); mp = 178–181 °C; TLC R_f_ = 0.35 (50% EA/hexanes); IR (ATR) 3296 (NH), 3247 (NH), 3102 (CH), 3066 (CH), 2912 (CH), 1654 (C=O), 1336 (SO_2_) cm^−1^; ^1^H NMR (400 MHz, CD_3_OD) δ 7.93–7.98 (m, 2H, ArH), 7.43 (s, 1H, thiophene ArH), 7.30 (t, *J* = 8.7 Hz, 2H, ArH), 4.03 (q, *J* = 7.0 Hz, 1H, OC-CH_-_N), 3.35–3.31 (m, 4H, O_2_SNCH_2_-), 2.74 (t, *J* = 5.4 Hz, 4H, S-CH_2_-), 2.36 (s, 3H, ArCH_3_), 1.35 (d, *J* = 7.0 Hz, 3H, CH_3_); ^13^C NMR (CD_3_OD, 100 MHz) δ 170.4 (C=O), 164.5 (d, *J* = 249.4 Hz, ArC-F), 137.7 (d, *J* = 3.0 Hz, Ar*C*-C-C-F), 134.2, 133.0, 130.2, 130.0 (d, *J* = 12.8 Hz, Ar*C*-C-F), 129.6, 116.5 (d, *J* = 22.6 Hz, Ar*C*-C-F), 52.2, 48.2, 26.8, 19.6 (CH_3_), 12.8 (CH_3_); Anal calcd for C_18_H_22_FN_3_O_5_S_4_: C, 42.59; H, 4.37; N, 8.28. Found: C, 42.55; H, 4.08; N, 8.20.
*(2R)-2-(4-Fluorobenzenesulfonamido)-N-[2-methyl-5-(thiomorpholine-4-sulfonyl)thiophen-3-yl] propanamide* (**49**). White foam (0.124 g, 21% over 3 steps); mp = 178–181 °C; TLC R_f_ = 0.35 (50% EA/hexanes); IR (ATR) 3297 (NH), 3247 (NH), 3101 (CH), 3066 (CH), 2958 (CH), 2952 (CH), 1654 (C=O), 1289 (SO_2_) cm^−1^; ^1^H NMR (400 MHz, CD_3_OD) δ 7.93–7.98 (m, 2H, ArH), 7.43 (s, 1H, thiophene ArH), 7.29 (t, *J* = 8.6 Hz, 2H, ArH), 4.04 (q, *J* = 7.2 Hz, 1H, OC-CH_-_N), 3.34 (t, *J* = 5.0 Hz, 4H, O_2_SNCH_2_-), 2.74 (t, *J* = 5.3 Hz, 4H, S-CH_2_-), 2.36 (s, 3H, ArCH_3_), 1.35 (d, *J* = 7.1 Hz, 3H, CH_3_); ^13^C NMR (100 MHz, CD_3_OD) δ 170.4 (C=O), 164.5 (d, *J* = 249.4 Hz, ArC-F), 137.7 (d, *J* = 3.0 Hz, Ar*C*-C-C-F), 134.2, 133.0, 130.2, 130.0 (d, *J* = 12.8 Hz, Ar*C*-C-F), 129.6, 116.5 (d, *J* = 22.6 Hz, Ar*C*-C-F), 52.2, 48.2, 26.8, 19.6 (CH_3_), 12.8 (CH_3_); Anal Calcd for C_18_H_22_FN_3_O_5_S_4_, C, 42.59, H, 3.37, N, 8.28. Found: C, 42.88, H, 4.14, N, 8.38.
*(2S)-2-(4-Fluorobenzenesulfonamido)-N-[2-methyl-5-(thiomorpholine-4-sulfonyl)thiophen-3-yl]-3-phenylpropanamide* (**50**). White powder (62 mg, 15% over 3 steps); mp = 197–202 °C; TLC R_f_ = 0.38 (40% EtOAc/hexanes); IR (ATR) 3246 (NH), 2922 (CH), 2854 (CH), 1652 (C=O), 1336 (SO_2_), 1157 (SO_2_), 1139 (SO_2_) cm^−1^; ^1^H NMR (400 MHz, acetone-d_6_) δ 9.02 (bs, 1H, NH), 7.82–7.81 (m, 2H, ArH), 7.55 (s, 1H, thiophene ArH), 7.23–7.18 (m, 7H, ArH), 7.14 (bs, 1H, NH), 4.31 (t, *J* = 6.7 Hz, 1H, OC-CH_-_N), 3.33–3.30 (m, 4H, O_2_SNCH_2_-), 3.13–3.08 (m, 1H, ArCH2), 2.99–2.94 (m, 1H, ArCH2), 2.77–2.75 (m, 4H, S-CH_2_-), 2.23 (s, 3H, ArCH_3_); ^13^C NMR (100 MHz, acetone-*d_6_*) δ 169.6 (C=O), 165.6 (d, *J* = 253.1 Hz, ArC-F), 138.0 (d, *J* = 3.9 Hz, Ar*C*-C-C-F), 137.4, 134.3, 133.2, 131.8, 130.8 (d, *J* = 9.2 Hz, Ar*C*-C-F), 130.3, 129.8, 129.2, 127.6, 116.8 (d, *J* = 23.2 Hz, Ar*C*-C-F), 59.2, 49.0, 39.9, 27.7, 12.4 (CH_3_); Anal. Calcd for C_24_H_26_FN_3_O_5_S_4_: C, 49.38; H, 4.49; N, 7.20. Found: C, 49.47; H, 4.31; N, 7.03.
*(2R)-2-(4-Fluorobenzenesulfonamido)-N-[2-methyl-5-(thiomorpholine-4-sulfonyl)thiophen-3-yl]-3-phenylpropanamide* (**51**). White powder (86 mg, 18% over 3 steps); mp = 199–204 °C; TLC R_f_ = 0.33 (40% EA/hexanes); IR (ATR) 3262 (NH), 2913 (CH), 2850 (CH), 1676 (C=O), 1333 (SO_2_), 1150 (SO_2_) cm^−1^; ^1^H NMR (400 MHz, CD_3_CN) δ 8.14 (s, 1H, NH), 7.77–7.73 (m, 2H, ArH), 7.36 (s, 1H, thiophene ArH), 7.27–7.12 (m, 7H, ArH), 6.29 (d, *J* = 9.2 Hz, 1H, NH), 4.19–4.13 (m, 1H, OC-CH_-_N), 3.29–3.26 (m, 4H, O_2_SNCH_2_-), 3.06–3.01 (m, 1H, ArCH2), 2.92–2.86 (m, 1H, ArCH2), 2.73–2.70 (m, 4H, S-CH_2_-), 2.17 (s, 3H, ArCH_3_); ^13^C NMR (100 MHz, acetone-*d_6_*) δ 169.6, 165.7 (d, *J* = 252.2 Hz, ArC-F), 138.1 (d, *J* = 3.3 Hz, Ar*C*-C-C-F), 137.4, 134.3, 133.3, 131.8, 130.8 (d, *J* = 9.4 Hz, Ar*C*-C-F), 130.3, 129.8, 129.2, 127.6, 116.8 (d, *J* = 22.9 Hz, Ar*C*-C-F), 59.3, 49.0, 40.0, 27.7, 12.4 (CH_3_); Anal. Calcd for C_24_H_26_FN_3_O_5_S_4_: C, 49.38; H, 4.49; N, 7.20. Found: C, 49.47; H, 4.35; N, 7.23.
*(2S)-2-(4-Fluorobenzenesulfonamido)-N-[2-methyl-5-(thiomorpholine-4-sulfonyl)thiophen-3-yl]-2-phenylacetamide* (**52**). White foam (0.135 g, 43% over 3 steps); mp = 80–84 °C; TLC R_f_ = 0.53 (50% EA/hexanes); IR (ATR) 3265 (NH), 2980 (CH), 2888 (CH), 1676 (C=O), 1336 (SO_2_) cm^−1^; ^1^H NMR (400 MHz, DMSO-*d_6_*) δ 10.08 (s, 1H, NH), 8.87 (d, *J* = 9.8 Hz, 1H, NH), 7.81 (q, *J* = 7.8 Hz, 2H, ArH), 7.38–7.42 (m, 3H, ArH), 7.23–7.33 (m, 5H, ArH), 5.29 (d, *J* = 9.7 Hz, 1H, OC-CH_-_N), 3.19 (t, *J* = 4.5 Hz, 4H, O_2_SNCH_2_-), 2.70 (t, *J* = 4.9 Hz, 4H, S-CH_2_-), 2.24 (s, 3H, CH_3_); ^13^C NMR (DMSO-*d_6_*, 100 MHz) δ 168.0 (C=O), 164.5 (d, *J* = 249.2 Hz, ArC-F), 137.6 (d, *J* = 2.9 Hz, Ar*C*-C-C-F), 137.2, 134.3, 132.7, 130.2, 130.2, 130.1, 128.9, 128.8 (d, *J* = 83.8 Hz, Ar*C*-C-F), 127.5, 116.3 (d, J = 22.5 Hz, Ar*C*-C-F), 55.4, 48.2, 26.8, 12.8 (CH_3_); Anal calcd for C_23_H_24_FN_3_O_5_S_4_: C, 48.49; H, 4.25; N, 7.38. Found: C, 48.12; H, 4.06; N, 7.74.
*(2S)-1-(4-Fluorobenzenesulfonyl)-N-[2-methyl-5-thiomorpholine-4-sulfonyl)thiophen-3-yl]pyrrolidine-2-carboxamide* (**53**). White power (0.160 g, 54% over 3 steps); mp = 119–123 °C; TLC R_f_ = 0.34 (50% EA/hexanes); IR (ATR) 3342 (NH), 2980 (CH), 1684 (C=O), 1338 (SO_2_) cm^−1^; ^1^H NMR (400 MHz, DMSO-*d_6_*) δ 9.81 (s, 1H, NH), 7.96 (m, 2H, ArH), 7.63 (s, 1H, thiophene ArH), 7.50 (m, 2H, ArH), 4.25 (t, *J* = 6.7 Hz, 1H, OC-CH_-_N), 3.46–3.53 (m, 1H), 3.24 (t, *J* = 4.9 Hz, 4H, O_2_SNCH_2_-), 2.72 (t, *J* = 4.9 Hz, 4H, S-CH_2_-), 2.40 (s, 3H, ArCH_3_), 1.87–1.96 (m, 3H), 1.56–1.65 (m, 1H), 1.26–1.20 (m, 1H); ^13^C NMR (100 MHz, DMSO-*d_6_*) δ 170.5 (C=O), 165.2 (d, *J* = 250.4 Hz, ArC-F), 135.3, 133.9, 133.3, 130.8, 130.5 (d, *J* = 90.9 Hz, Ar*C*-C-F), 130.2, 117.1 (d, *J* = 22.5 Hz, Ar*C*-C-F), 61.7, 49.6, 48.3, 31.5, 26.8, 24.7, 13.0 (CH_3_); Anal calcd for C_20_H_24_FN_3_O_5_S_4_: C, 45.01; H, 4.53; N, 7.87. Found: C, 45.38; H, 4.73; N, 7.53.

### 3.6. General Procedure for the Synthesis of ***64***–***73***

The amine salt **21** was suspended in dry DCM (0.7 M) and dry TEA (2.2 equiv) was added. The electrophile (sulfonyl chloride, benzyl chloride, or chloroformate, (1.1 equiv)) was added and the mixture was stirred at rt for 24 h under argon. The reaction was then taken up in EA and washed with H_2_O and 5% aq. HCl. The organic layer was dried over Na_2_SO_4_, filtered, and concentrated. The residue was then purified by trituration from DCM or by silica gel chromatography, yielding the pure product.

*2-Benzenesulfonamido-N-[2-methyl-5-(thiomorpholine-4-sulfonyl)thiophen-3-yl]acetamide* (**64**). White powder (0.074 g, 27%); mp = 172–176 °C; TLC R_f_ = 0.26 (50% EA/hexanes); IR (ATR) 3352 (NH), 3160 (CH), 2980 (CH), 2889 (CH), 1682 (C=O), 1587 (C=C), 1329 (SO_2_), 1156 (SO_2_) cm^−1^; ^1^H NMR (DMSO-*d_6_*, 400 MHz) δ 9.72 (s, 1H, NH), 8.12 (t, *J* = 6.4 Hz, 1H, ArH), 7.82 (d, *J =* 7.5 Hz, 2H, ArH), 7.55–7.65 (m, 4H, ArH), 3.70 (d, *J* = 6.2 Hz, 2H, OC-CH_2-_N), 3.20 (t, *J* = 4.8 Hz, 4H, O_2_SNCH_2_-), 2.71 (t, *J* = 4.5 Hz, 4H, S-CH_2_-), 2.32 (s, 3H, CH_3_); ^13^C NMR (DMSO-*d_6_*, 100 MHz) δ 166.9 (C=O), 140.8, 134.0, 133.2, 133.0, 130.0, 129.6, 129.6, 127.1, 48.3, 45.7 (O_2_SNCH_2_-), 26.8 (S-CH_2_-), 12.9 (CH_3_); Anal. Calcd for C_17_H_21_N_3_O_5_S_4_: C, 42.93; H, 4.45; N, 8.84. Found: C, 42.65; H, 4.06; N, 8.50.
*N-[2-Methyl-5-(thiomorpholine-4-sulfonyl)thiophen-3-yl]-2-{[(4-methylbenzenesulfonyl) carbamoyl] amino}acetamide* (**65**). White powder (0.037g, 12%); mp = 192–195 °C; TLC R_f_ = 0.39 (10% MeOH/DCM); IR (ATR) 3329 (NH), 3107 (CH), 2980 (CH), 2889 (CH), 1708 (C=O), 1655 (C=C), 1333 (SO_2_), 1148 (SO_2_) cm^−1^; ^1^H NMR (acetone-*d_6_*, 400 MHz) δ 9.08 (s, 1H, NH), 7.92 (d, *J* = 8.3 Hz, 2H, ArH), 7.83 (s, 1H, thiophene ArH), 7.41 (d, *J* = 8.4 Hz, 2H, ArH), 6.97 (bs, 1H, NH), 3.78 (d, *J* = 5.0 Hz, 2H, OC-CH_2-_N), 3.33 (t, *J* = 5.0 Hz, 4H, O_2_SNCH_2_-), 2.75 (t, *J* = 5.0 Hz, 4H, S-CH_2_-), 2.43 (s, 3H, CH_3_), 2.37 (s, 3H, CH_3_); ^13^C NMR (acetone-*d_6_*, 100 MHz) δ 167.1 (C=O), 151.4, 144.3, 137.7, 132.9, 132.8, 130.9, 129.5, 129.0, 127.5, 48.2, 43.2, 26.8, 20.6 (CH_3_), 11.6 (CH_3_); Anal. Calcd for C_19_H_24_N_4_O_6_S_4_: C, 48.60; H, 4.94; N, 8.95. Found: C, 48.85; H, 4.64; N, 9.07.
*2-(4-Iodobenzenesulfonamido)-N-[2-methyl-5-(thiomorpholine-4-sulfonyl)thiophen-3-yl] acetamide* (**66**). White powder (0.094 g, 27%); mp = 198–202 °C; TLC R_f_ = 0.51 (50% EA/hexane); IR (ATR) 3381 (NH), 3156 (CH), 2979 (CH), 2907 (CH), 2865 (CH), 1655 (C=O), 1335 (SO_2_), 1327 (SO_2_), 1164 (SO_2_), 1148 (SO_2_) cm^−1^; ^1^H NMR (DMSO-*d_6_*, 400 MHz) δ 9.73 (s, 1H, NH), 8.20 (t, *J =* 5.9 Hz, 1H, NH), 7.97 (d, *J* = 8.1 Hz, 2H, ArH), 7.59 (d, *J* = 5.0 Hz, 2H, ArH), 7.57 (s, 1H, thiophene ArH), 3.71 (d, *J* = 6.2 Hz, 2H, OC-CH_2_-N), 3.22 (t, *J* = 4.6 Hz, 4H, O_2_SNCH_2_-), 2.71 (t, *J* = 4.6 Hz, 4H, S-CH_2_-), 2.32 (s, 3H , CH_3_); ^13^C NMR (DMSO-*d_6_*, 100 MHz) δ 166.8 (C=O), 140.5, 138.5, 134.1, 133.2, 130.1, 129.6, 128.8, 101.0, 48.3, 45.6 (O_2_SNCH_2_-), 26.8 (S-CH_2_-), 12.9 (CH_3_); Anal. Calcd for C_17_H_20_IN_3_O_5_S_4_: C, 33.95; H, 3.35; N, 6.99. Found: C, 33.93; H, 3.42; N, 6.65.
*N-[2-Methyl-5-(thiomorpholine-4-sulfonyl)thiophen-3-yl]-2-(4-nitrobenzenesulfonamido) acetamide* (**67**). White powder (0.059 g, 20%); mp = 209–212 °C; TLC R_f_ = 0.19 (50% EA/hexanes); IR (ATR) 3380 (NH), 3247 (NH), 3105 (CH), 3063 (CH), 2980 (CH), 2914 (CH), 2853 (CH), 1668 (C=O), 1531 (C=C), 1349 (SO_2_), 1332 (SO_2_), 1150 (SO_2_) cm^−1^; ^1^H NMR (acetone-*d_6_*, 400 MHz) δ 9.73 (s, 1H, NH), 8.21 (t, *J =* 5.6 Hz, 1H, NH), 7.98 (d, *J* = 7.5 Hz, 2H, ArH), 7.60 (d, *J* = 5.7 Hz, 2H, ArH), 7.57 (s, 1H, thiophene ArH), 3.72 (d, *J* = 5.3 Hz, 2H, OC-CH_2_-N), 3.22 (t, *J* = 4.1 Hz, 4H, O_2_SNCH_2_-), 2.71 (t, *J* = 4.7 Hz, 4H, S-CH_2_-), 2.32 (s, 3H, CH_3_); ^13^C NMR (acetone-*d_6_*, 100 MHz) δ 166.8 (C=O), 140.5, 138.5, 134.1, 133.2, 130.1, 129.5, 128.8, 101.0, 48.3, 45.6 (O_2_SNCH_2_-), 26.8 (S-CH_2_-), 12.9 (CH_3_); Anal. Calcd for C_17_H_20_N_4_O_7_S_4_: C, 39.22; H, 3.87; N, 10.76. Found: C, 39.14; H, 3.84; N, 11.14.
*N-[2-Methyl-5-(thiomorpholine-4-sulfonyl)thiophen-3-yl]-2-(3-nitrobenzenesulfonamido) acetamide* (**68**). White powder (0.16 g, 53%); mp = 141–144 °C; TLC R_f_ = 0.19 (50% EA/hexanes); IR (ATR) 3365 (NH), 3104 (CH), 2927 (CH), 2851 (CH), 1677 (C=O), 1528 (C=C), 1349 (SO_2_), 1337 (SO_2_), 1161 (SO_2_) cm^−1^; ^1^H NMR (acetone-*d_6_*, 400 MHz) δ 9.13 (s, 1H, NH), 8.68 (t, *J* = 1.8 Hz, 1H, ArH), 8.50 (dd, *J* = 1.3, 8.3 Hz, 1H, ArH), 8.32 (d, *J* = 7.8 Hz, 1H, ArH), 7.93 (t, *J* = 7.9 Hz, 1H, ArH), 7.63 (s, 1H, thiophene ArH), 7.37 (bs, 1H, ArH), 4.00 (d, *J* = 5.5 Hz, 2H, OC-CH_2_-N), 3.29 (t, *J* = 4.9 Hz, 4H, O_2_SNCH_2_-), 2.75 (t, *J* = 4.9 Hz, 4H, S-CH_2_-), 2.37 (s, 3H, CH_3_); ^13^C NMR (acetone-*d*_6_, 100 MHz) δ 165.8 (C=O), 148.3, 142.4, 133.1, 133.0, 132.6, 131.0, 130.9, 128.8, 127.1, 122.1, 48.1, 45.6, 26.8 (S-CH_2_-), 11.6 (CH_3_); Anal. Calcd for C_17_H_20_N_4_O_7_S_4_: C, 39.22; H, 3.87; N, 10.76. Found: C, 39.23; H, 3.91; N, 10.94.
*N-[2-Methyl-5-(thiomorpholine-4-sulfonyl)thiophen-3-yl]-2-(2-nitrobenzenesulfonamido) acetamide* (**69**). Orange powder (0.140 g, 47%); mp = 101–104 °C; TLC R_f_ = 0.19 (50% EA/hexanes); IR (ATR) 3325 (NH), 3097 (CH), 2917 (CH), 1686 (C=O), 1336 (SO_2_), 1149 (SO_2_) cm^−1^; ^1^H NMR (CD_3_OD, 400 MHz) δ 8.11–8.15 (m, 1H, ArH), 7.90–7.93 (m, 1H, ArH), 7.79 (s, 1H, thiophene ArH), 4.03 (s, 2H, OC-CH_2_-N), 3.31–3.33 (m, 4H, O_2_SNCH_2_-), 2.72 (t, *J* = 5.2 Hz, 4H, S-CH_2_-), 2.36 (s, 3H, CH_3_); ^13^C NMR (CD_3_OD, 100 MHz) δ 167.8 (C=O), 148.0, 135.4, 133.8, 133.4, 132.4, 131.9, 131.0, 130.3, 129.3, 124.8, 48.0, 45.4, 26.7 (S-CH_2_-), 11.2 (CH_3_); Anal. Calcd for C_17_H_20_N_4_O_7_S_4_: C, 39.22; H, 3.87; N, 10.76. Found: C, 39.60; H, 4.11; N, 10.48.
*N-[2-Methyl-5-(thiomorpholine-4-sulfonyl)thiophen-3-yl]-2-(2-trifluoromethylbenzene sulfonamido)acetamide* (**70**). Off-white foam (0.030 g, 24%); mp = 89–93 °C; TLC R_f_ = 0.32 (50% EA/50% hexanes); IR (ATR) 3329 (NH), 2980 (CH), 1685 (C=O), 1581 (C=C), 1307 (SO_2_), 1143 (SO_2_), 1116 (SO_2_) cm^−1^; ^1^H NMR (acetone-*d_6_*, 400 MHz) δ 8.93 (s, 1H, NH), 8.13 (dd, *J* = 3.7, 5.0 Hz, 1H, ArH), 7.87 (dd, *J* = 3.7, 5.5 Hz, 1H, ArH), 7.74 (dd, *J* = 6.0, 3.7 Hz, 2H, ArH), 7.51 (s, 1H, thiophene ArH), 6.75 (bs, 1H, NH), 3.85 (d, *J =* 6.0 Hz, 2H, OC-CH_2_-N), 3.17 (t, *J* = 4.6 Hz, 4H, O_2_SNCH_2_-), 2.61 (t, *J* = 5.0 Hz, 4H, S-CH_2_-), 2.23 (s, 3H, CH_3_); ^13^C NMR (acetone-*d_6_*, 100 MHz) δ 166.0 (C=O), 138.9 (q, *J* =1.1 Hz, *C-*C-CF_3_), 133.2, 133.1, 132.8, 131.2, 130.0, 128.9, 128.5 (q, *J* = 6.3 Hz, *C-*C-CF_3_), 127.3, 127.2 (q, *J* = 32.8 Hz, *C*-CF3), 123.3 (q, *J* = 271.6 Hz, CF_3_), 48.1, 45.7, 26.8 (S-CH_2_-), 11.7 (CH_3_); Anal. Calcd for C_18_H_20_F_3_N_3_O_5_S_4_: C, 39.77; H, 3.71; N, 7.73. Found: C, 39.74; H, 3.95; N, 7.42.
*N-[2-Methyl-5-(thiomorpholine-4-sulfonyl)thiophen-3-yl]-2-(1,1,3-trioxo-2,3-dihydro-1-benzothiazol-2-yl)acetamide* (**71**). White powder (0.041g, 36%); mp = 234–237 °C; TLC R_f_ = 0.28 (50% EA/hexanes); IR (ATR) 3264 (NH), 2980 (CH), 2918 (CH), 1743 (C=O), 1671 (C=O), 1335 (SO_2_), 1150 (SO_2_) cm^−1^; ^1^H NMR (acetone-*d_6_*, 400 MHz) δ 9.32 (s, 1H, NH), 8.22 (d, *J* = 7.7 Hz, 1H, ArH), 8.05–8.17 (m, 3H, ArH), 7.80 (s, 1H, thiophene ArH), 4.64 (s, 2H, OC-CH_2_-N), 3.31 (bs, 4H, O_2_SNCH_2_-), 2.74 (t, *J* = 5.0 Hz, 4H, S-CH_2_-), 2.44 (s, 3H, CH_3_); ^13^C NMR (acetone-*d_6_*, 100 MHz) δ 163.4 (C=O), 158.9 (C=O), 137.9, 135.7, 135.0, 133.8, 132.6, 131.1, 129.0, 127.2, 125.1, 121.3, 48.2, 40.4, 26.8 (S-CH_2_-), 11.7 (CH_3_); Anal. Calcd for C_18_H_19_N_3_O_6_S_4_: C, 43.10; H, 3.82; N, 8.38. Found: C, 43.35; H, 3.98; N, 8.17.
*N-[2-Methyl-5-(thiomorpholine-4-sulfonyl)thiophen-3-yl]-2-(phenylformamido)acetamide* (**72**). White powder (0.079 g, 31%); mp = 189–193 °C; TLC R_f_ = 0.53 (5% MeOH/DCM); IR (ATR) 3309 (NH), 3098 (CH), 3066 (CH), 2907 (CH), 2860 (CH), 1675 (C=O), 1636 (C=C), 1337 (SO_2_), 1172 (SO_2_) cm^−1^; ^1^H NMR (acetone-*d_6_*, 400 MHz) δ 9.70 (s, 1H, NH), 8.65 (s, 1H, NH), 8.41 (d, *J =* 7.5 Hz, 2H, ArH), 8.30 (s, 1H, thiophene ArH), 8.01 (t, *J* = 7.5 Hz, 1H, ArH), 7.94 (t, *J* = 7.1 Hz, 2H, ArH), 4.68 (d, *J* = 5.2 Hz, 2H, OC-CH_2_-N), 3.77 (t, *J* = 4.9 Hz, 4H, O_2_SNCH_2_-), 3.19 (t, *J* = 4.9 Hz, 4H, S-CH_2_-), 2.87 (s, 3H, CH_3_); ^13^C NMR (acetone-*d_6_*, 100 MHz) δ 167.5 (C=O), 167.3 (C=O), 134.2, 133.2, 132.7, 131.5, 130.9, 129.0, 128.4, 127.3, 48.2, 43.6, 26.8 (S-CH_2_-), 11.7 (CH_3_); Anal. Calcd for C_18_H_21_N_3_O_4_S_3_: C, 49.18; H, 4.82; N, 9.56. Found: C, 49.00; H, 5.02; N, 9.22.
*Benzyl N-({[2-methyl-5-(thiomorpholine-4-sulfonyl)thiophen-3-yl]carbamoyl}methyl)carbamate* (**73**). White powder (0.079 g, 31%); mp = 69–74 °C; TLC R_f_ = 0.21 (50% EA/hexanes); IR (ATR) 3309 (NH), 3098 (CH), 3066 (CH), 2962 (CH), 2907 (CH), 2860 (CH), 2466 (CH), 1676 (C=O), 1636 (C=C), 1350 (SO_2_), 1333 (SO_2_), 1137 (SO_2_) cm^−1^; ^1^H NMR (DMSO-*d_6_*, 400 MHz) δ 9.78 (s, 1H, NH), 7.74 (s, 1H, NH), 7.58 (t, *J =* 5.9 Hz, 1H, ArH), 7.31–7.38 (m, 5H, ArH), 5.06 (s, 2H, OCH_2_Ph), 3.85 (d, *J* = 5.8 Hz, 2H, OC-CH_2_-N), 3.23 (t, *J* = 4.6 Hz, 4H, O_2_SNCH_2_-), 2.71 (t, *J* = 4.6 Hz, 4H, S-CH_2_-), 2.39 (s, 3H, CH_3_); ^13^C NMR (DMSO-*d_6_*, 100 MHz) δ 168.5 (C=O), 157.1 (C=O), 137.5, 133.7, 133.5, 129.9, 129.7, 128.8, 128.3, 128.2, 66.0 (OCH_2_Ph), 48.3, 44.0, 26.8 (S-CH_2_-), 13.0 (CH_3_); Anal. Calcd for C_18_H_21_N_3_O_4_S_3_: C, 49.18; H, 4.82; N, 9.56. Found: C, 49.00; H, 5.02; N, 9.22.

### 3.7. Evaluation of SHIP1 Activity

Cloning and expression of tSHIP1. Cloning and expression of truncated human SHIP1 protein (tSHIP1) from human whole blood mRNA has been previously described [48]. Briefly, tSHIP1 (SHIP1, residues 397–864) was amplified from pET24TEV-tSHIP1. Plasmids were extracted with Midi Prep (Qiagen, Germantown, MD, USA) and sequences were verified by sequencing of the entire expression regions. The plasmids were then transformed into *E. coli* BL21 (New England Biolabs, Ipswich, MA, USA). Fresh colonies were amplified in 1 L LB at 37 °C, shaking at 250rpm until OD 600nm reached 0.6. Protein expression was induced with 200 mM IPTG and incubation was continued at 16 °C, shaking at 250 rpm for 16 h. Following centrifugation, bacteria pellets were stored overnight at −20 °C. Protein was extracted from bacterial cell pellets using BugBuster HT (Millipore, Burlington, MA, USA). The His-tagged protein was purified by FPLC using a HisTrapHP 5 mL column (Cytiva Life Sciences, Marlborough, MA, USA) at a 5 mL/min flow rate and 10 mM-1M imidazole gradient in Buffer A (20 mM Tris pH 8.0, 300 mM NaCl, 10 mM imidazole, 5 mM β-mercaptoethanol). Fractions containing active protein by Malachite Green Phosphatase Release Assay (Echelon, see below) were pooled, concentrated, and buffer exchanged to reduce imidazole concentration by centrifugation using Pierce Protein Concentrators 30KMWCO PES, (ThermoFisher Scientific, Waltham, MA, USA). Protein was aliquoted and stored at −80 °C in a mixture of Buffer A (without β-ME) and 50% glycerol.

Malachite Green Phosphatase Release Assays. Malachite Green Phosphatase Release Assays (Echelon Biosciences, Salt Lake City, UT, USA) were performed with recombinant human truncated SHIP1 (tSHIP1) and SHIP2 (Echelon Biosciences). Briefly, serial dilutions of the compounds dissolved in DMSO were added to the recombinant enzymes diluted in reaction buffer Rx (50 mM Hepes pH 7.4, 150 mM NaCl, 1 mM MgCl_2_, 0.25 mM EDTA) in triplicate reactions in 96-well plates. Reactions were incubated for 2 min at room temperature. An amount of 2.5 mL of 1 mM Phosphatidylinositol 3,4,5-trisphosphate diC8 (PI(3,4,5)P_3_diC8) (Echelon Biosciences, Salt Lake City, UT, USA) was added to each reaction to a final concentration of 100 µM in a final volume of 25 mL/well. Following 20 min incubation at 37 °C, 100 µL of Malachite Green Solution (Echelon Biosciences, Salt Lake City, UT, USA) was added to each well, and plates were incubated at room temperature in the dark for 15 min. Plates were then read at 620nm on a plate reader (Synergy 2, BioTek, Shoreline, WA, USA).

## 4. Conclusions

The K306 bis-sulfonamide structure was modified to investigate structure–activity relationships. The thiomorpholine subunit is shown to be a potential liability, as its oxidation leads to inactive or insoluble analogs. Replacement of the thiomorpholine with a piperidine or a butyl sulfonamide provides structures that also act as SHIP1 activators without this problem. Substitutions in the aromatic fragment of K306 (fragment B) show that there is some tolerance for change in this area, with a 2-chlorothiophene and a 1,4-disubstituted benzene ring able to take the place of the thiophene. Substitution of the glycine for a proline or phenylglycine in the amino acid fragment of the molecule (fragment C) appears to provide more potent activators. The 4-fluorobenzenesulfonamide in fragment D can also be modified, with the incorporation of electron-withdrawing groups on this benzensulfonamide being beneficial to the activator activity. The results of this study are now being applied to the development of new SHIP activators with improved potency and bioavailability. In the future, we hope to use these molecules to define the allosteric binding site where the binding of small molecules to SHIP1 leads to increased enzyme activity. In addition, we plan to evaluate the anti-inflammatory properties of these molecules in model systems of a number of disease states, including Alzheimer’s disease.

## Figures and Tables

**Table 1:** Synthesis of K306 Analogs Varying Fragment A.

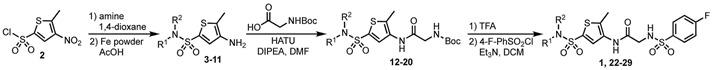			
Entry	Aminothiophene	Amide	Bis-Sulfonamide Analog
1	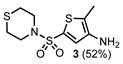	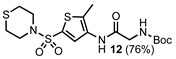	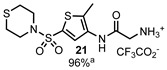
2	−	−	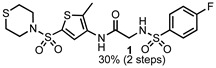
2	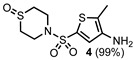	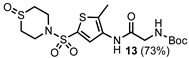	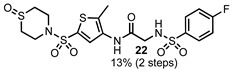
3	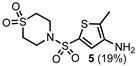	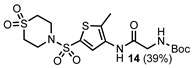	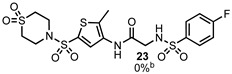
4	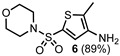	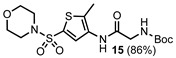	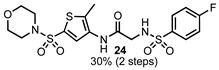
5	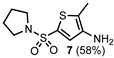	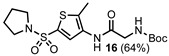	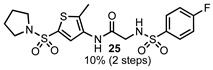
6	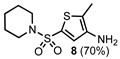	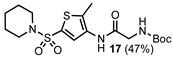	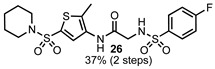
7	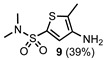	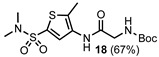	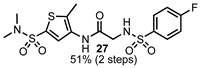
8	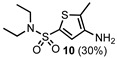	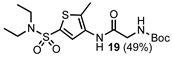	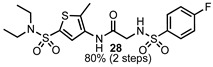
9	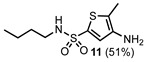	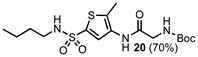	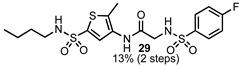

^a^ Amine **21** is the product of the TFA deprotection and was isolated before formation of the sulfonamide. ^b^ The sulfone **23** was insoluble (see text).

**Table 2:** Synthesis of K306 Analogs Varying Fragment C.

	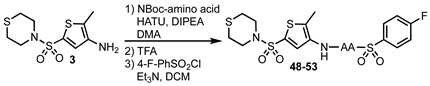		
Entry	Amino Acid	Product	Yield ^a^
1	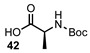	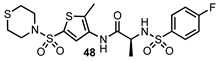	21%
2	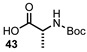	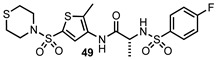	21%
3	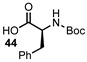	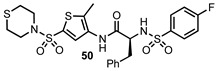	15%
4	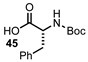	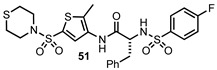	18%
5	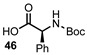	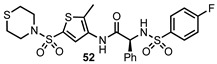	43%
6	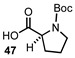	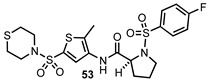	54%

^a^ Isolated yield over 3 steps.

**Table 3:** Synthesis of K306 Analogs Varying Fragment D.

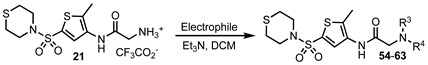			
Entry	Electrophile	Product	Yield
1	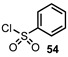	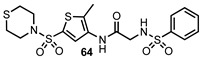	27%
2	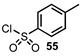	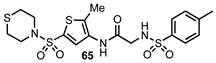	12%
3	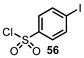	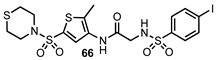	27%
4	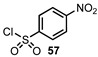	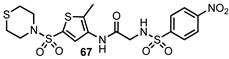	20%
5	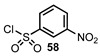	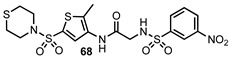	53%
6	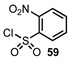	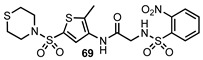	47%
7	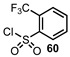	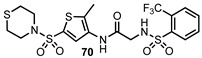	24%
8	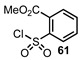	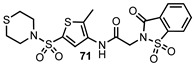	36%
9	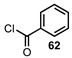	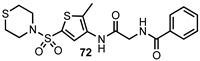	31%
10	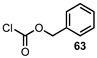	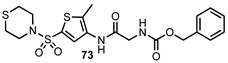	31%

## Data Availability

Data are contained within the article and supplementary materials.

## Supplementary Materials

The following supporting information can be downloaded at https://www.mdpi.com/article/10.3390/molecules28248048/s1: ^1^H NMR and ^13^C NMR spectra of compounds **1**, **3**–**22**, **24**–**29**, **31**–**33**, **35**–**37**, **39**–**41**, **48**–**53**, **64**–**73**.
