# Supplementary material for: Structure–Activity Studies on Bis-Sulfonamide SHIP1 Activators
Source: Molecules. 2023 Dec 12;28(24):8048. doi: 10.3390/molecules28248048 (PMC10745928; doi:10.3390/molecules28248048)
Supplement: Supplementary file 1 [file molecules-28-08048-s001.zip › molecules-2716349-supplementary.pdf]

Supporting Information

Structure–Activity Studies on Bis-Sulfonamide SHIP1 Activators

Shea T. Meyer,[1] Sandra Fernandes,[2] Robert E. Anderson,[1] Angela M. Pacherille,[1] Bonnie Toms,[2] William G. Kerr,[2] and John D. Chisholm*[1]*

[1]Department of Chemistry, Syracuse University, Syracuse, NY, USA and [2]Department of Microbiology & Immunology, SUNY Upstate Medical University, Syracuse, NY, USA

jdchisho@syr.edu

Contents

Table of Contents	S1
NMR Spectra	S2-S53

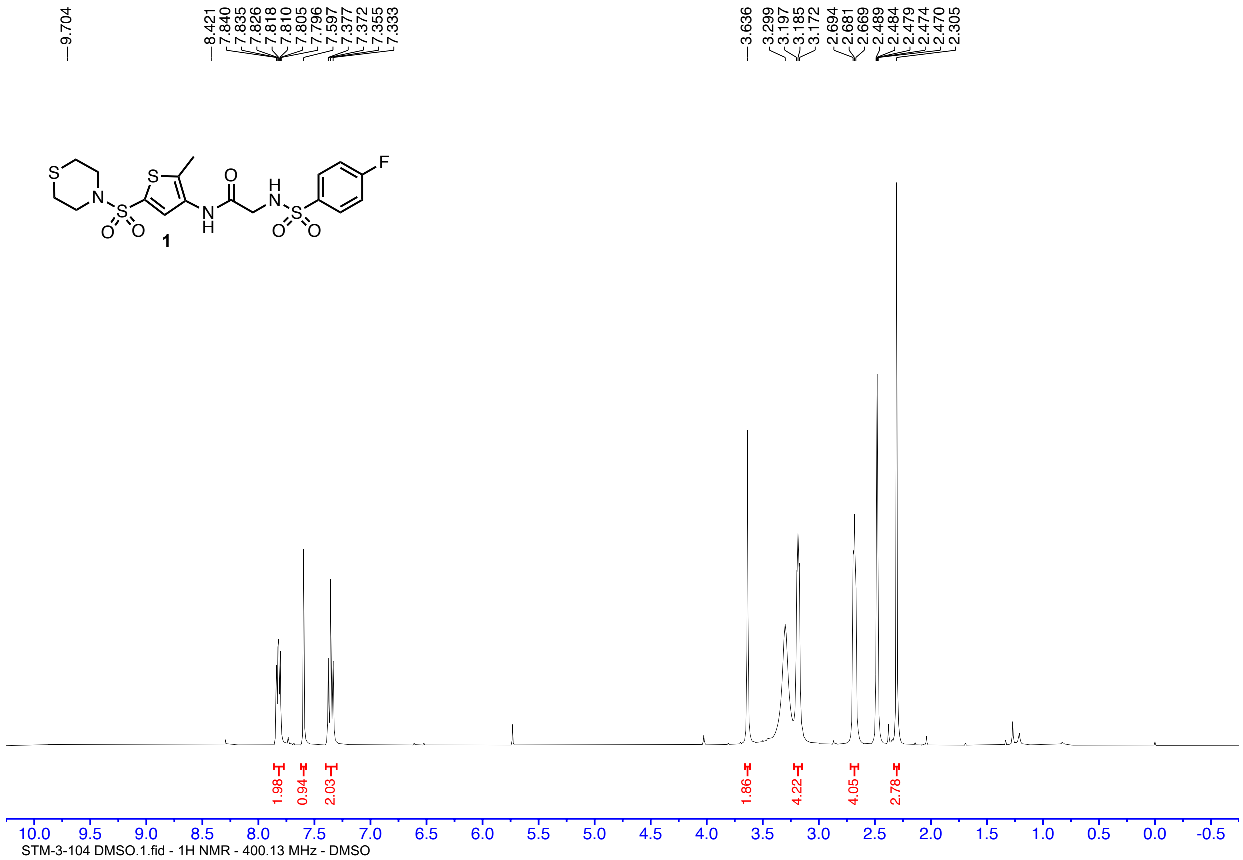

STM-3-104 DMSO.1.fid - 1H NMR - 400.13 MHz - DMSO

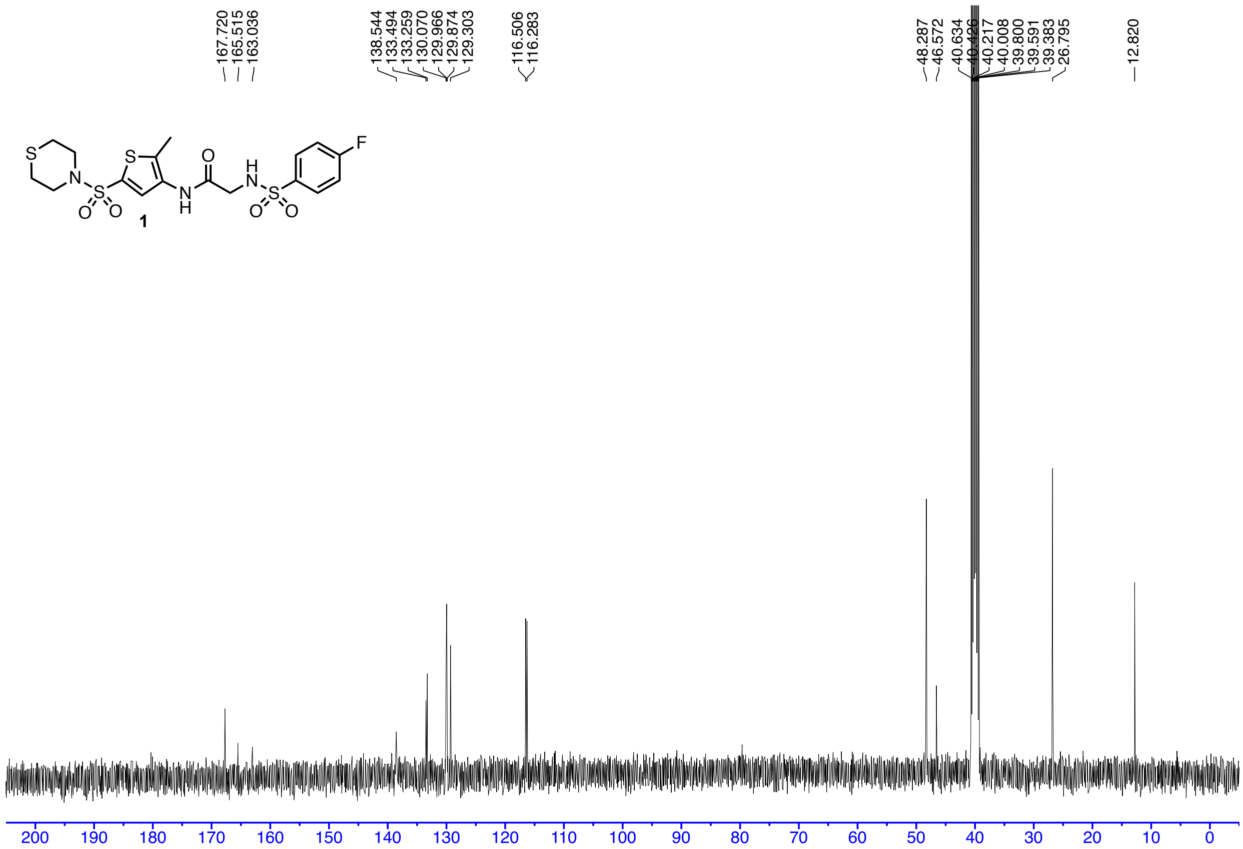

STM-3-104 DMSO CNMR.1.fid - 13C NMR - 100.62 MHz - DMSO

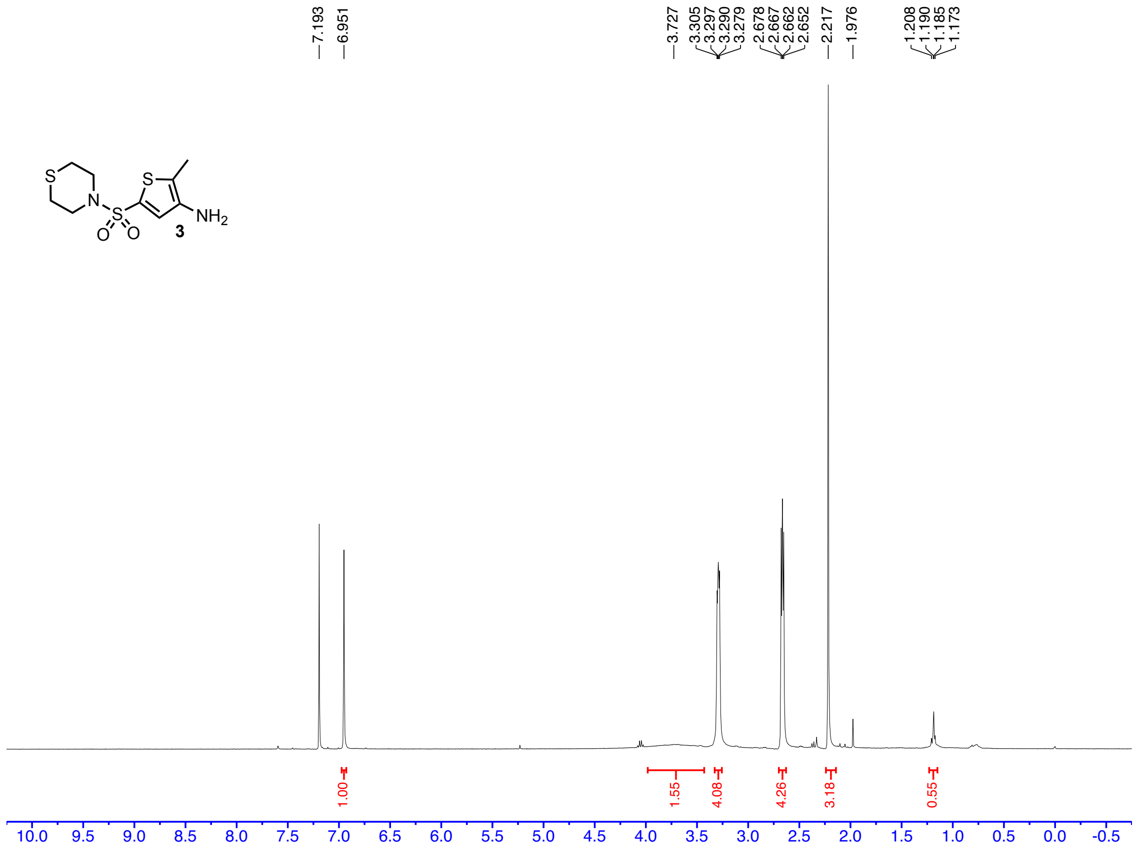

STM-3-63 - 1H NMR - 400.13 MHz - CDCl3

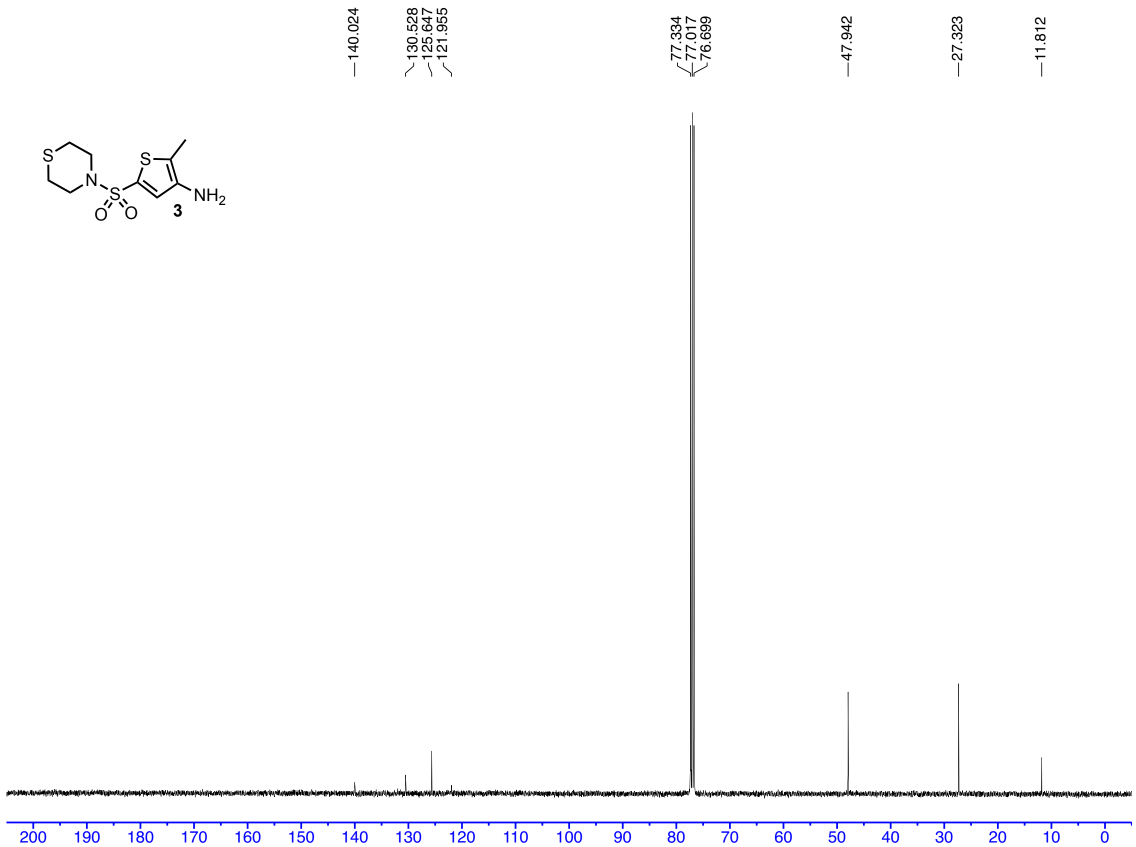

STM-3-63 CNMR - 13C NMR - 100.62 MHz - CDCl3

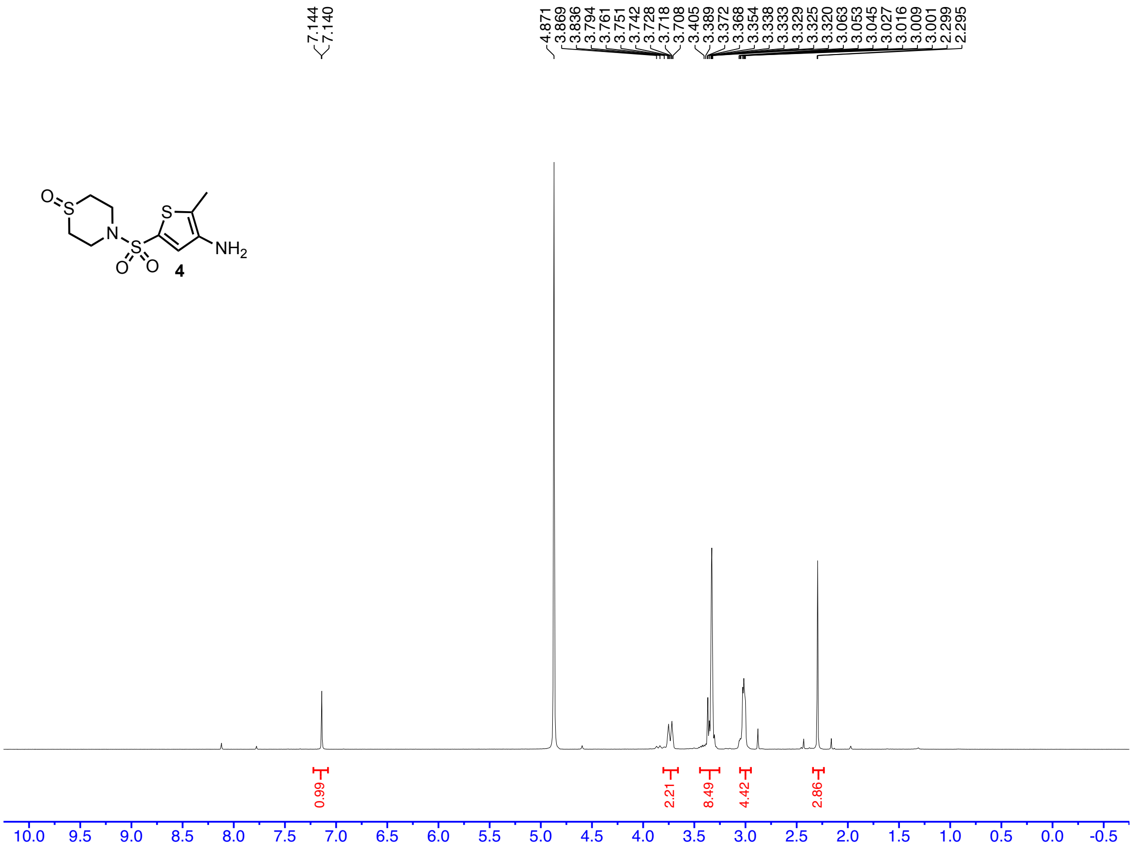

STM-4-54 MeOD - 1H NMR - 400.13 MHz - MeOD

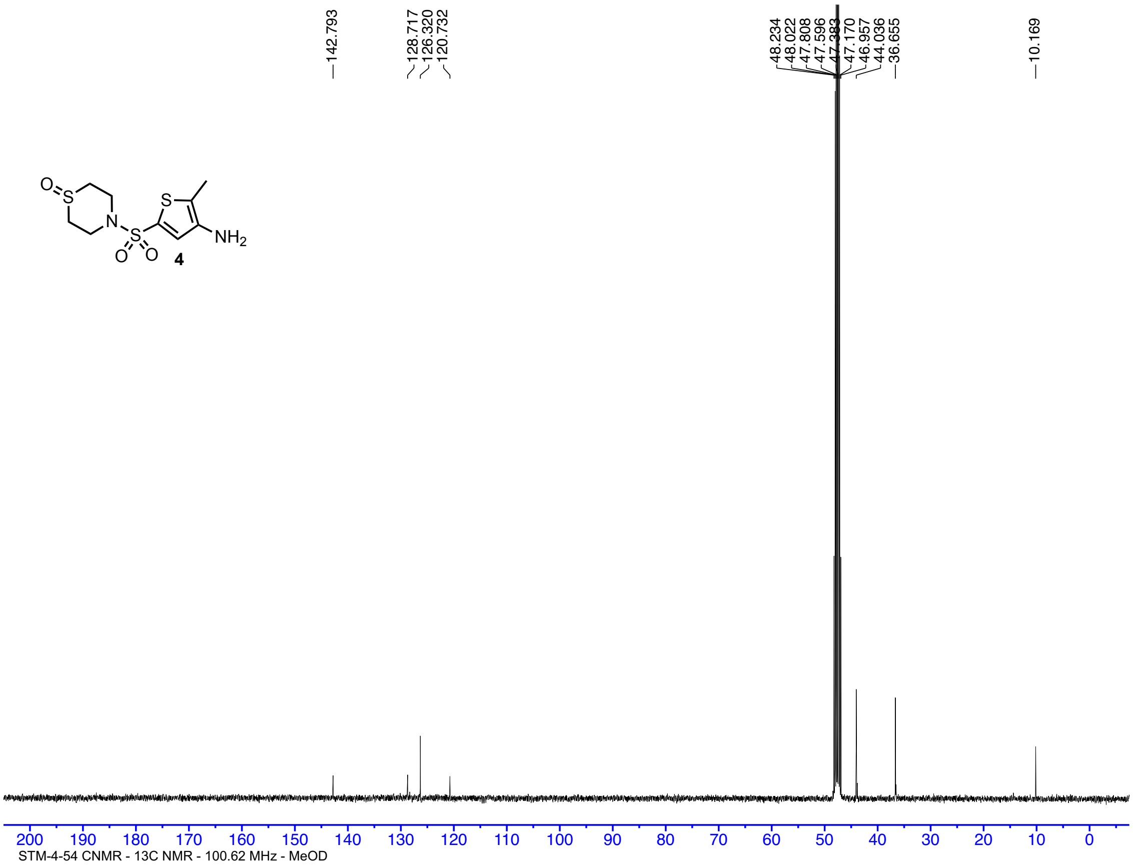

STM-4-54 CNMR - 13C NMR - 100.62 MHz - MeOD

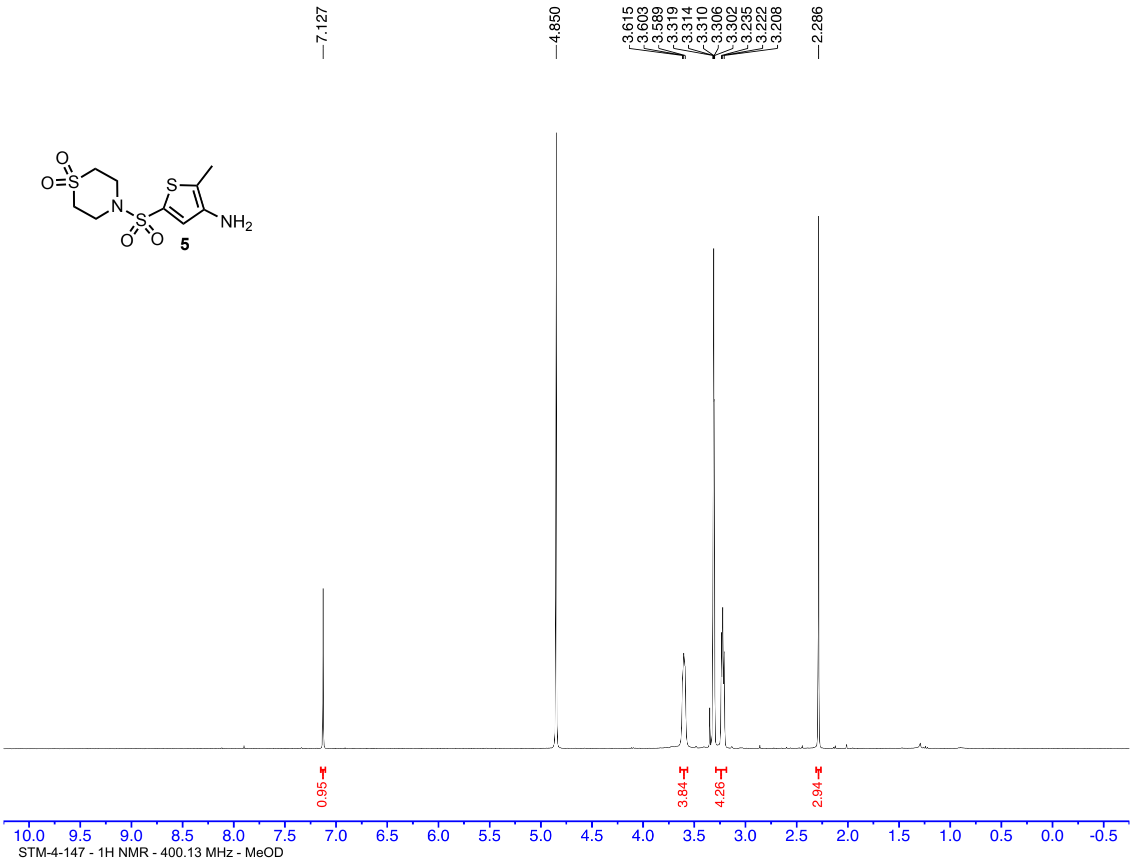

7.127
4.850
3.615
3.603
3.589
3.319
3.314
3.310
3.306
3.302
3.235
3.222
3.208
2.286
NH2
5
0.95
3.84
4.26
2.94
STM-4-147 - 1H NMR - 400.13 MHz - MeOD

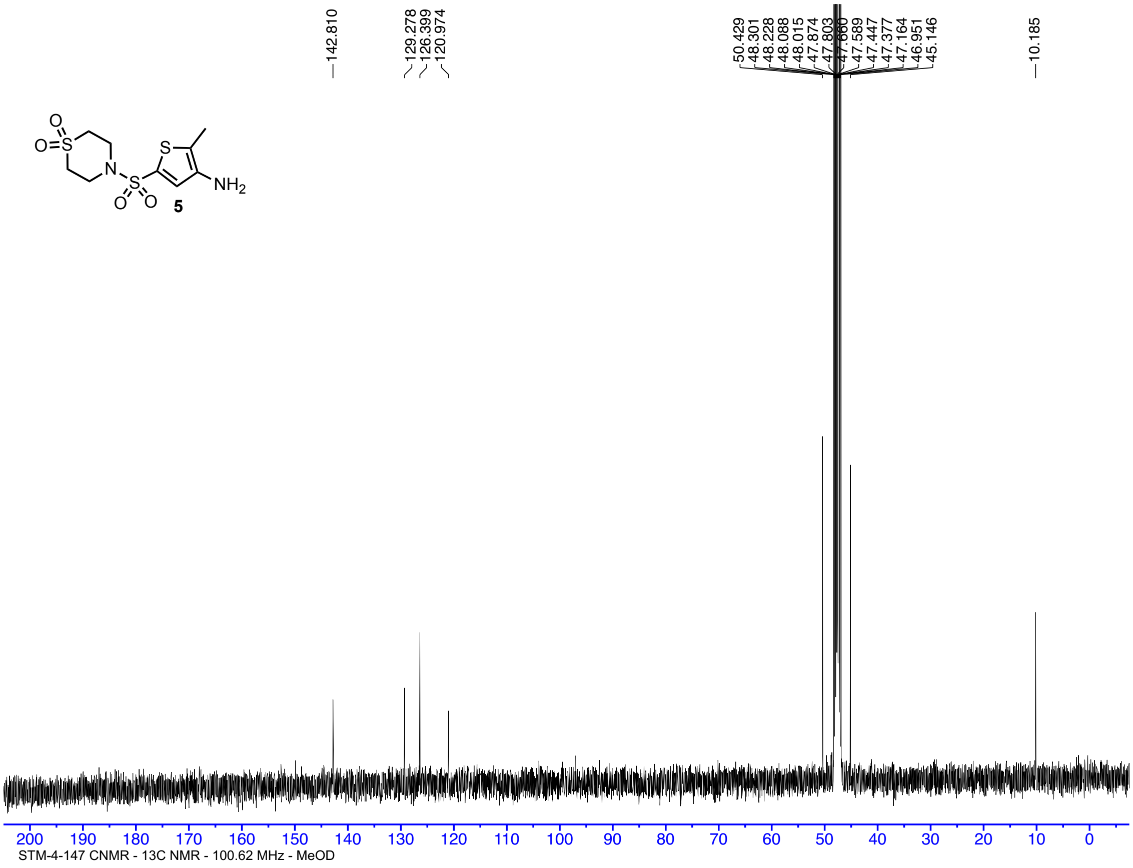

142.810
129.278
126.399
120.974
50.429
48.301
48.228
48.088
48.015
47.874
47.803
47.589
47.447
47.377
47.164
46.951
45.146
10.185
NH2
5
STM-4-147 CNMR - 13C NMR - 100.62 MHz - MeOD

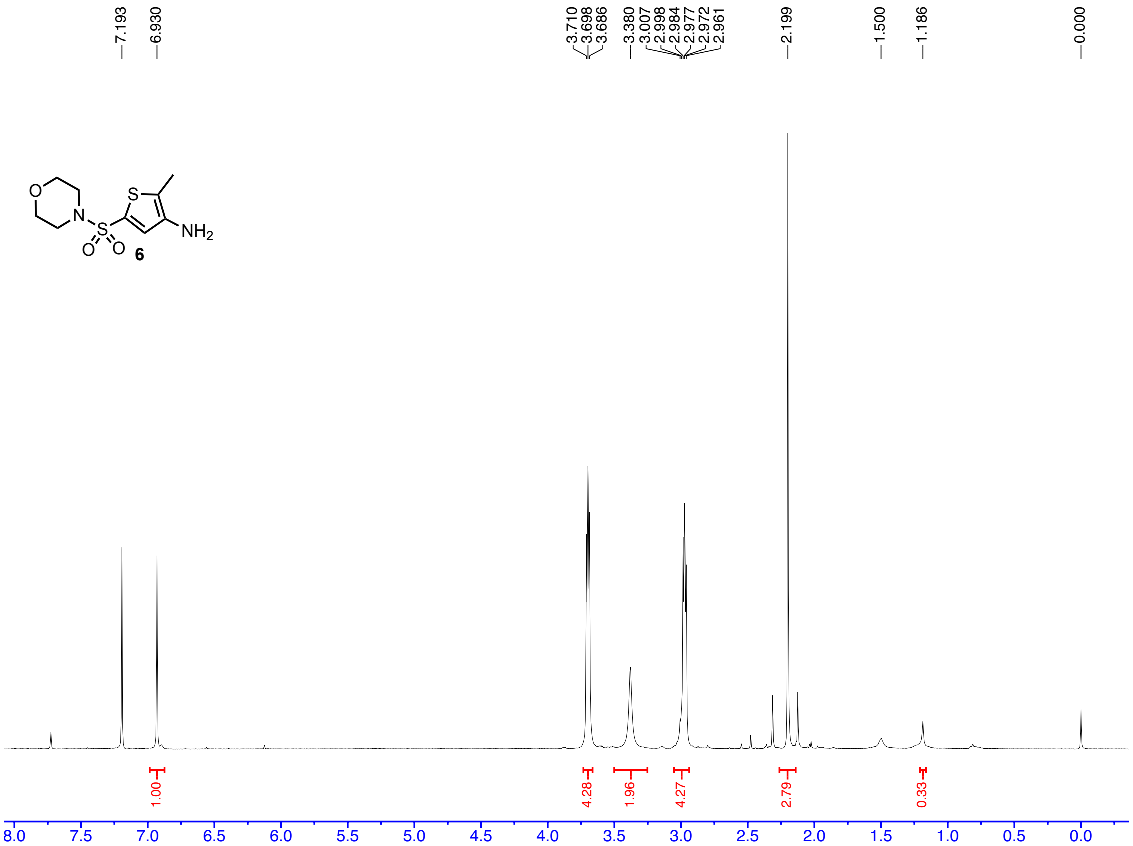

STM-3-111 - 1H NMR - 400.13 MHz - CDCl3

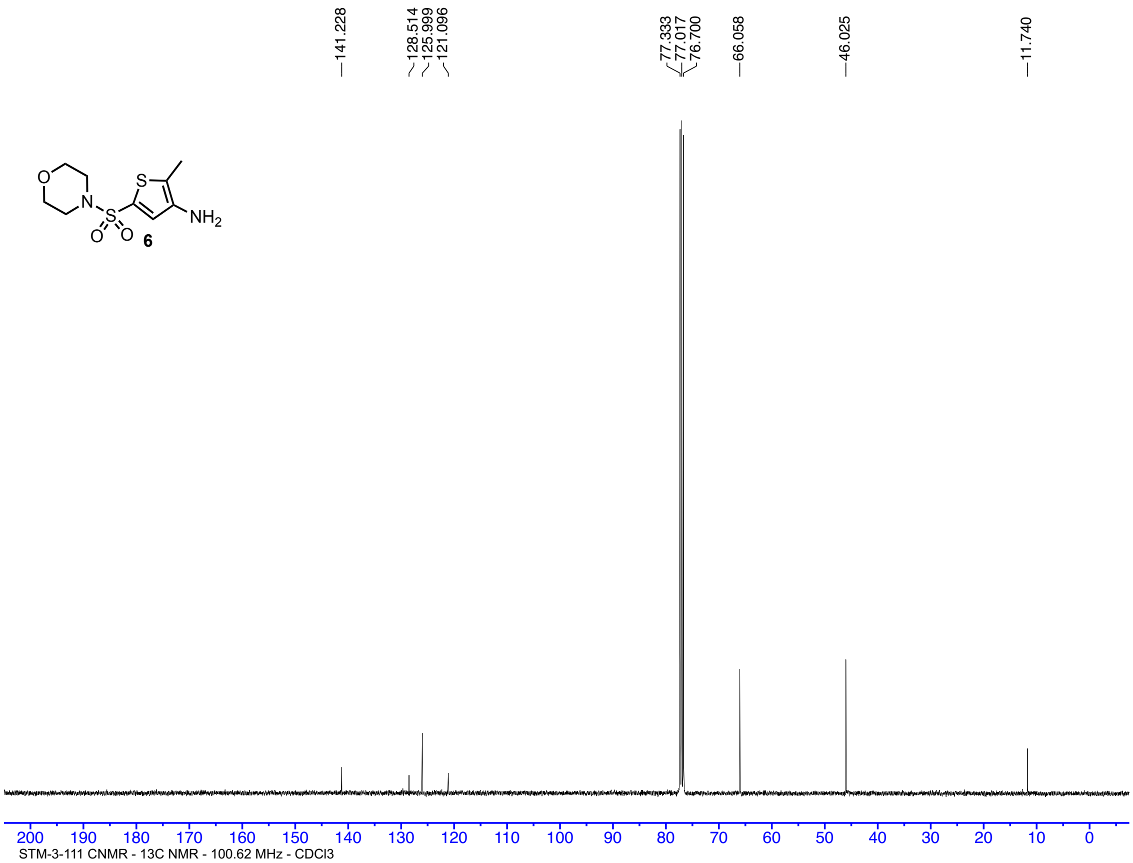

STM-3-111 CNMR - 13C NMR - 100.62 MHz - CDCl3

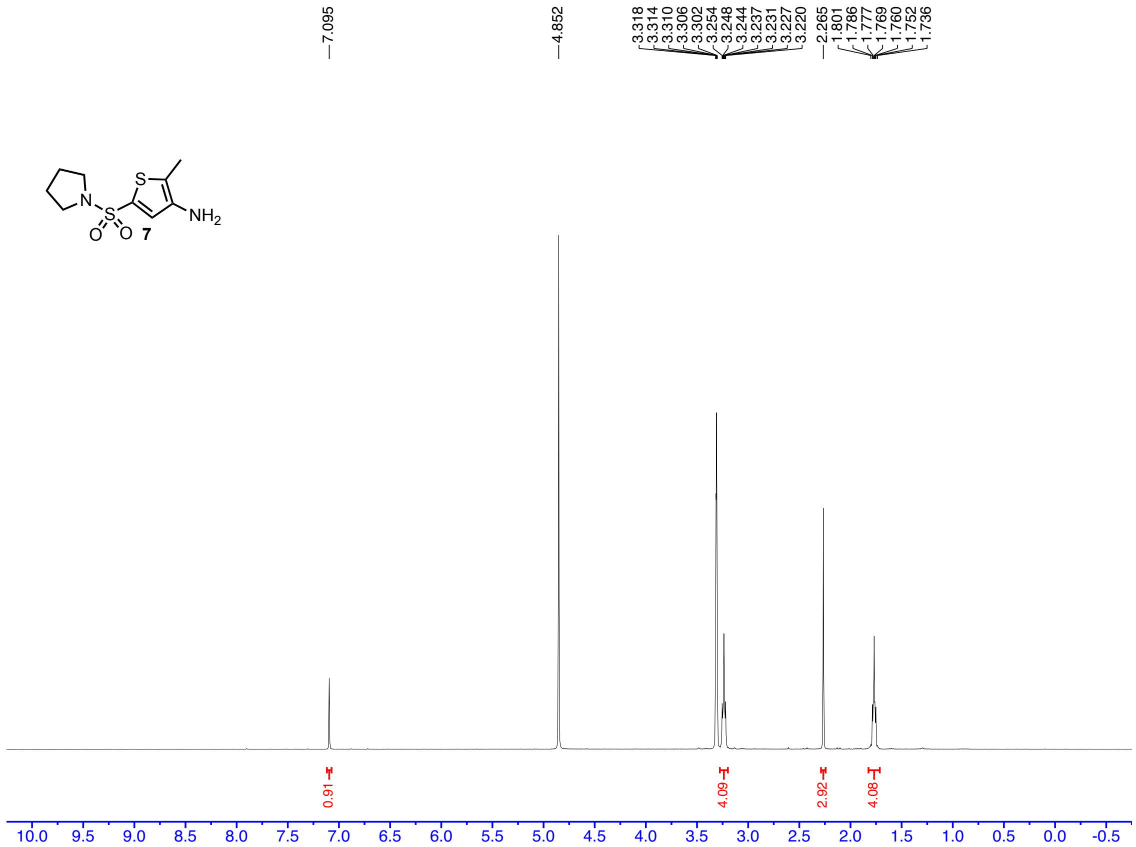

STM-5-1 MeOD - 1H NMR - 400.13 MHz - MeOD

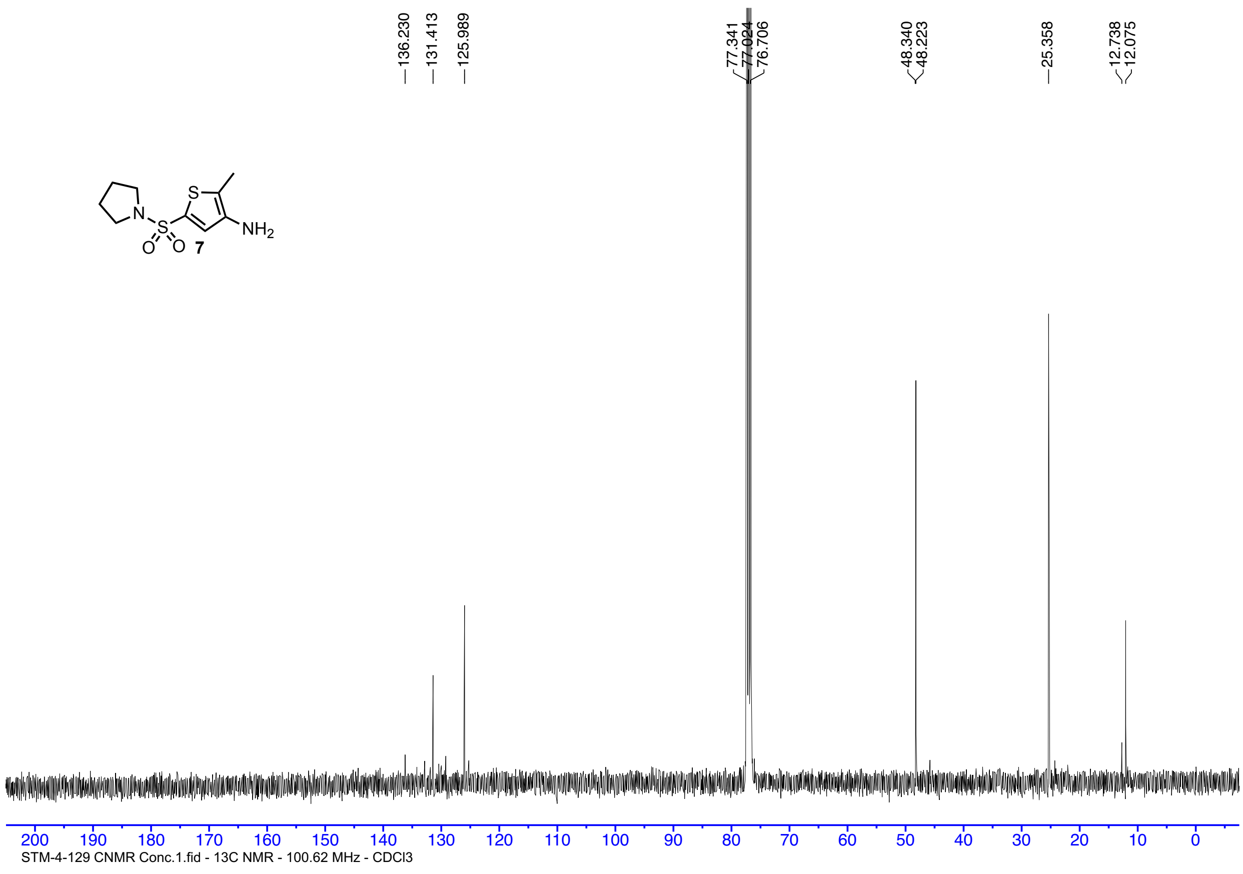

STM-4-129 CNMR Conc.1.fid - 13C NMR - 100.62 MHz - CDCl3

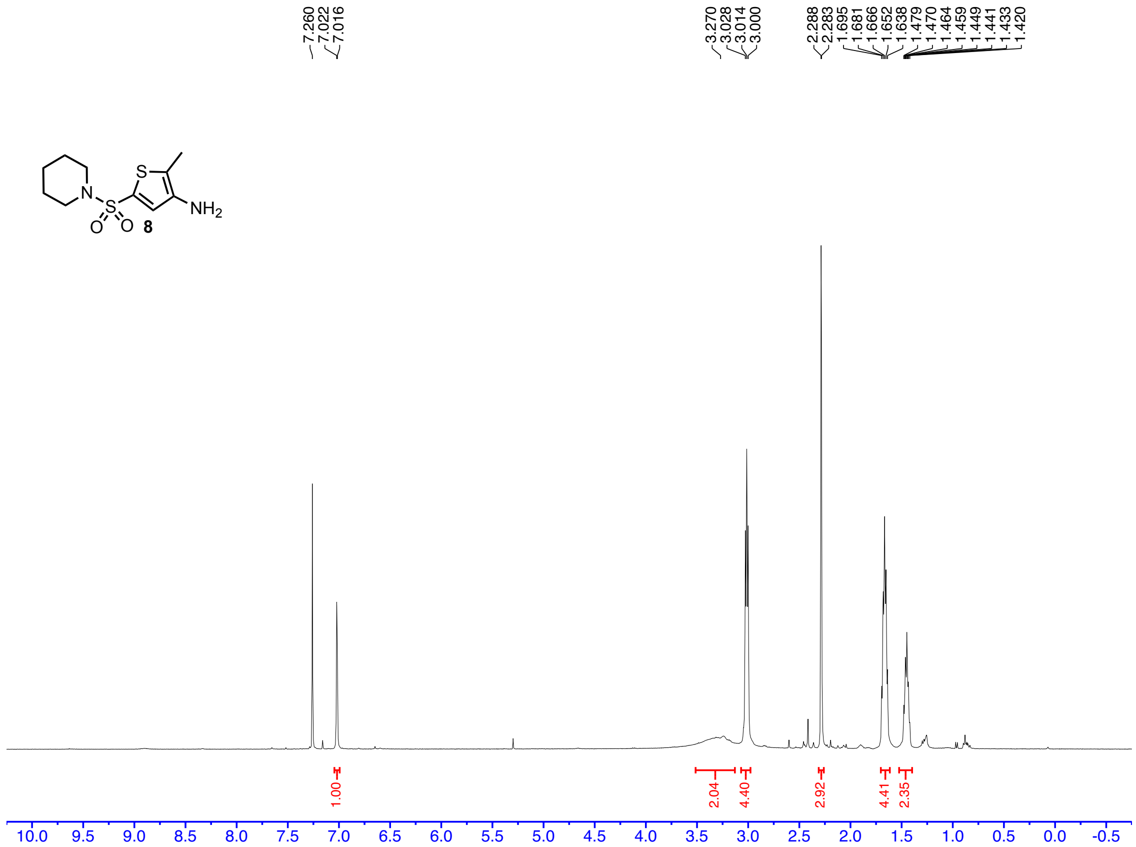

STM-4-9 Pure - 1H NMR - 400.13 MHz - CDCl3

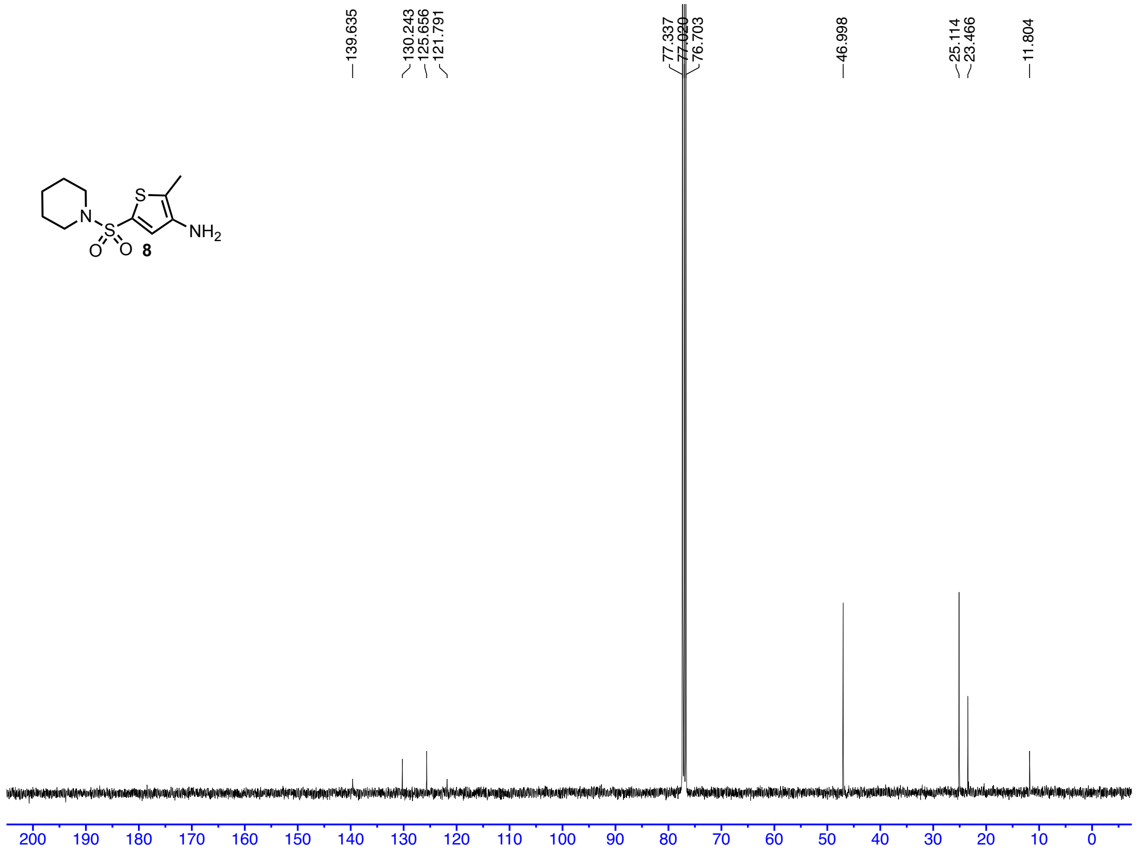

STM-4-9 CNMR - 13C NMR - 100.62 MHz - CDCl3

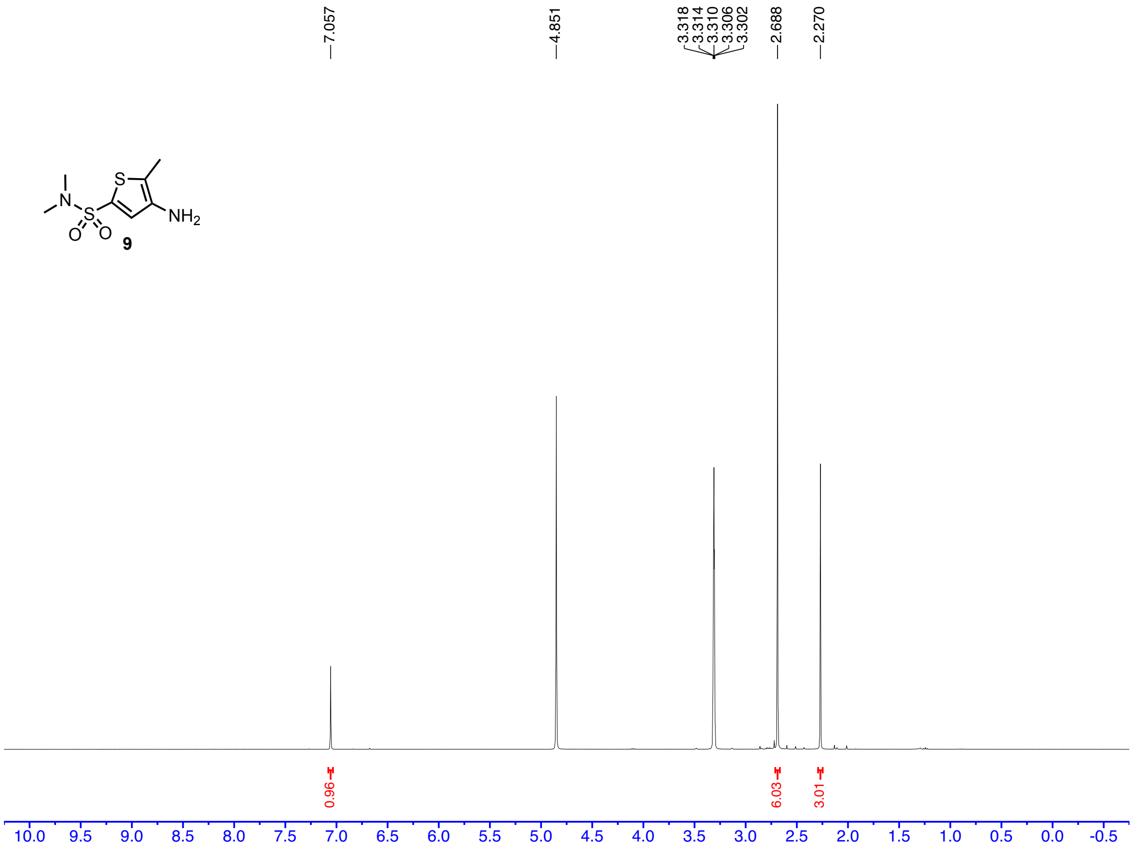

STM-4-131 (2-28-22) - 1H NMR - 400.13 MHz - MeOD

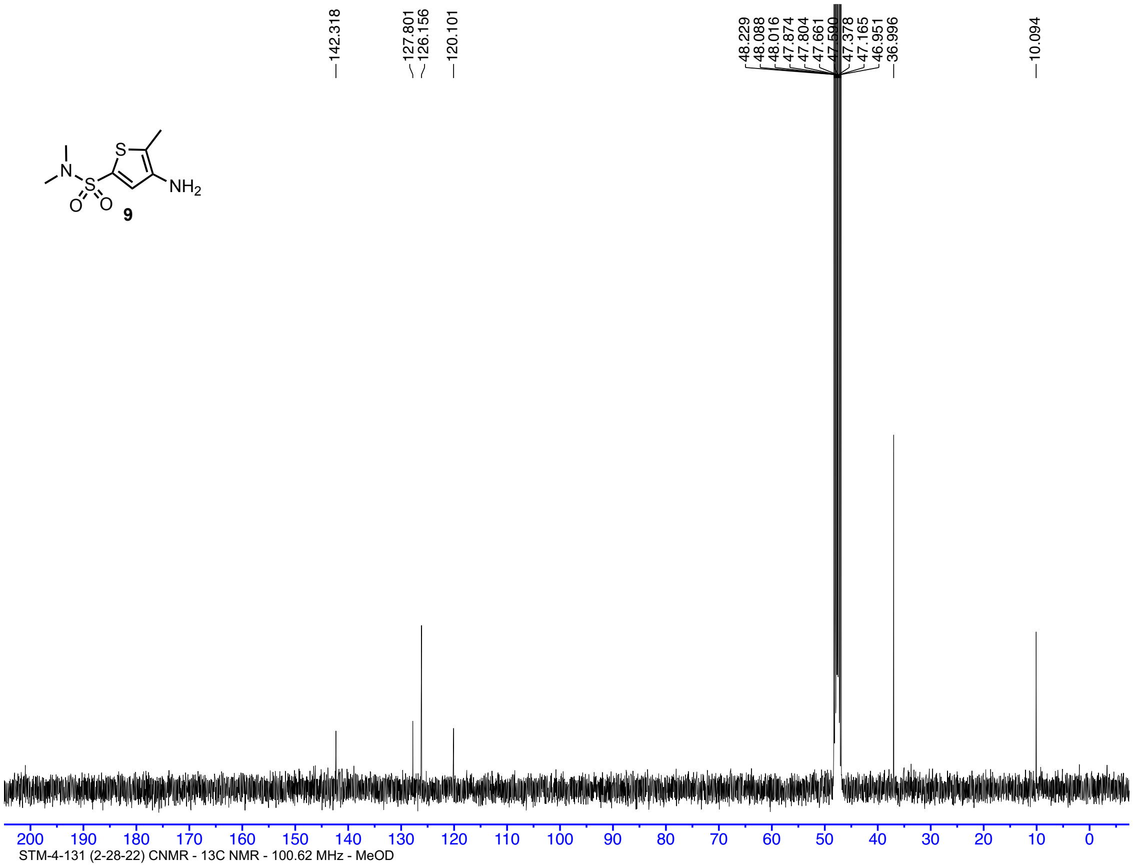

STM-4-131 (2-28-22) CNMR - 13C NMR - 100.62 MHz - MeOD

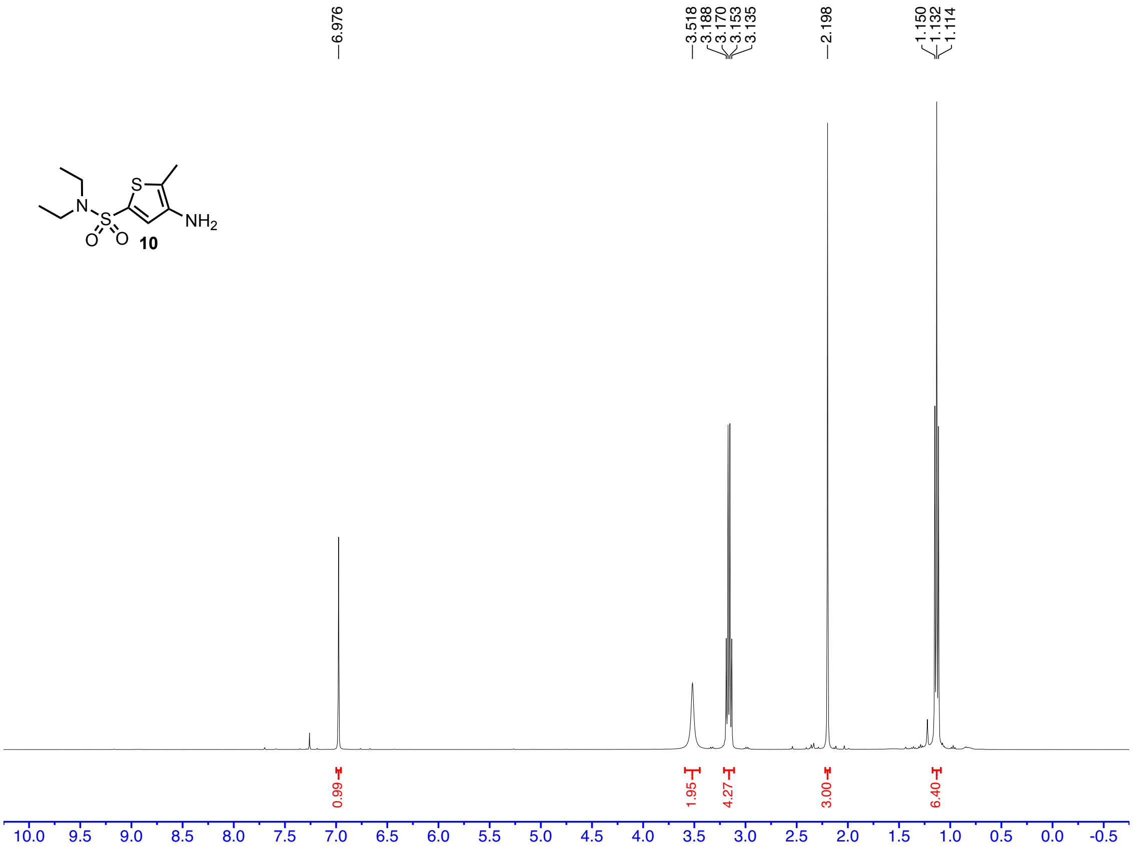

STM-4-123 - 1H NMR - 400.13 MHz - CDCl3

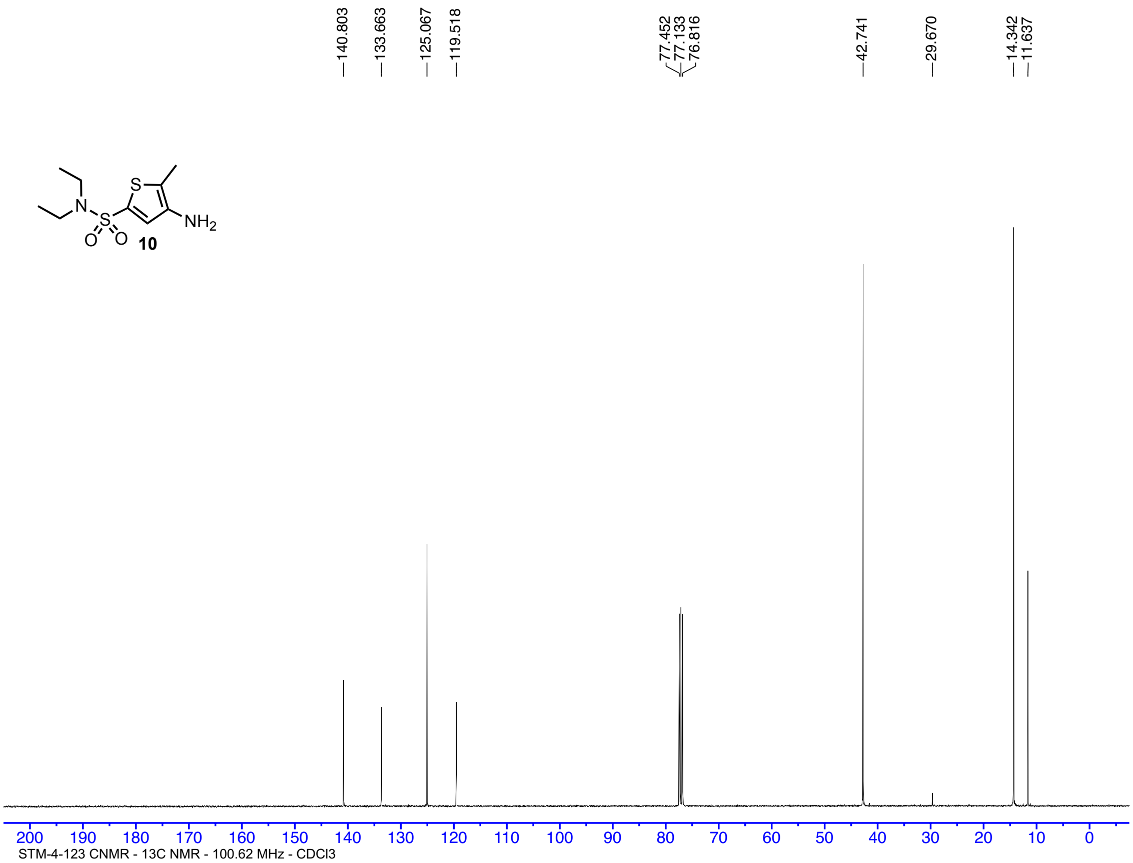

STM-4-123 CNMR - 13C NMR - 100.62 MHz - CDCl3

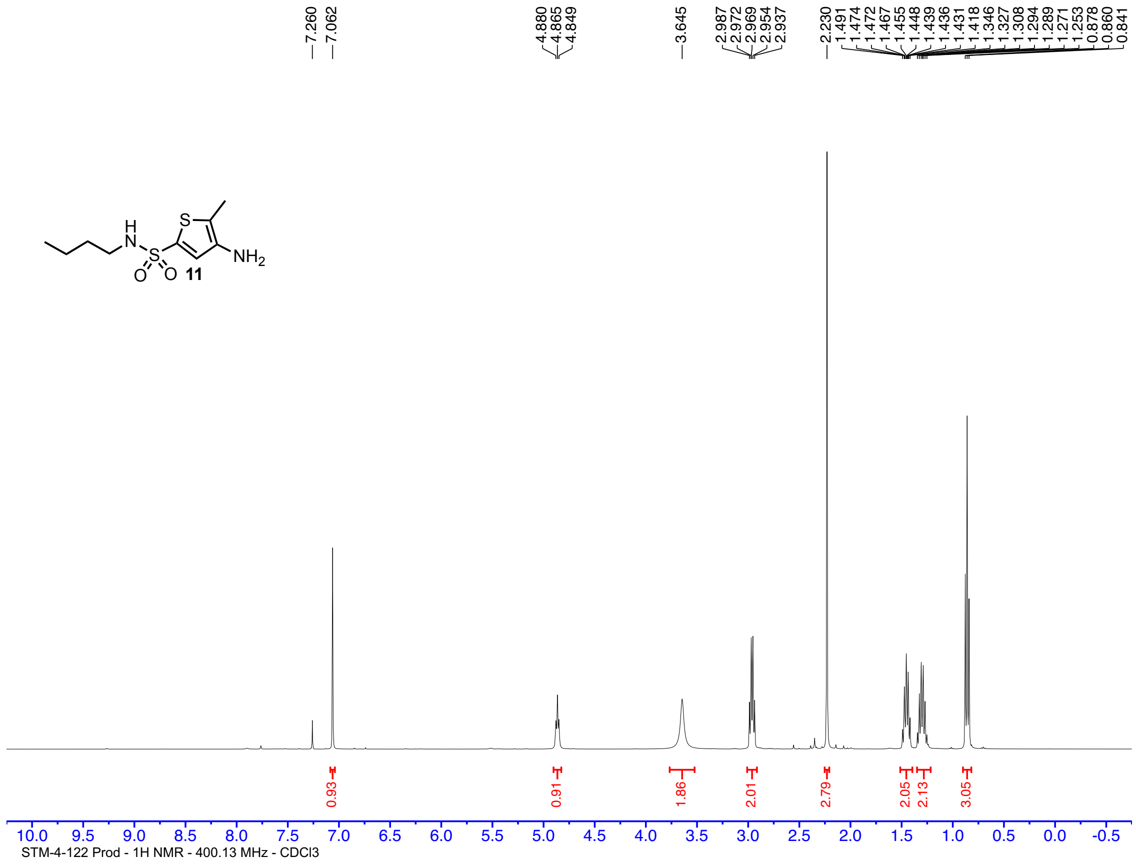

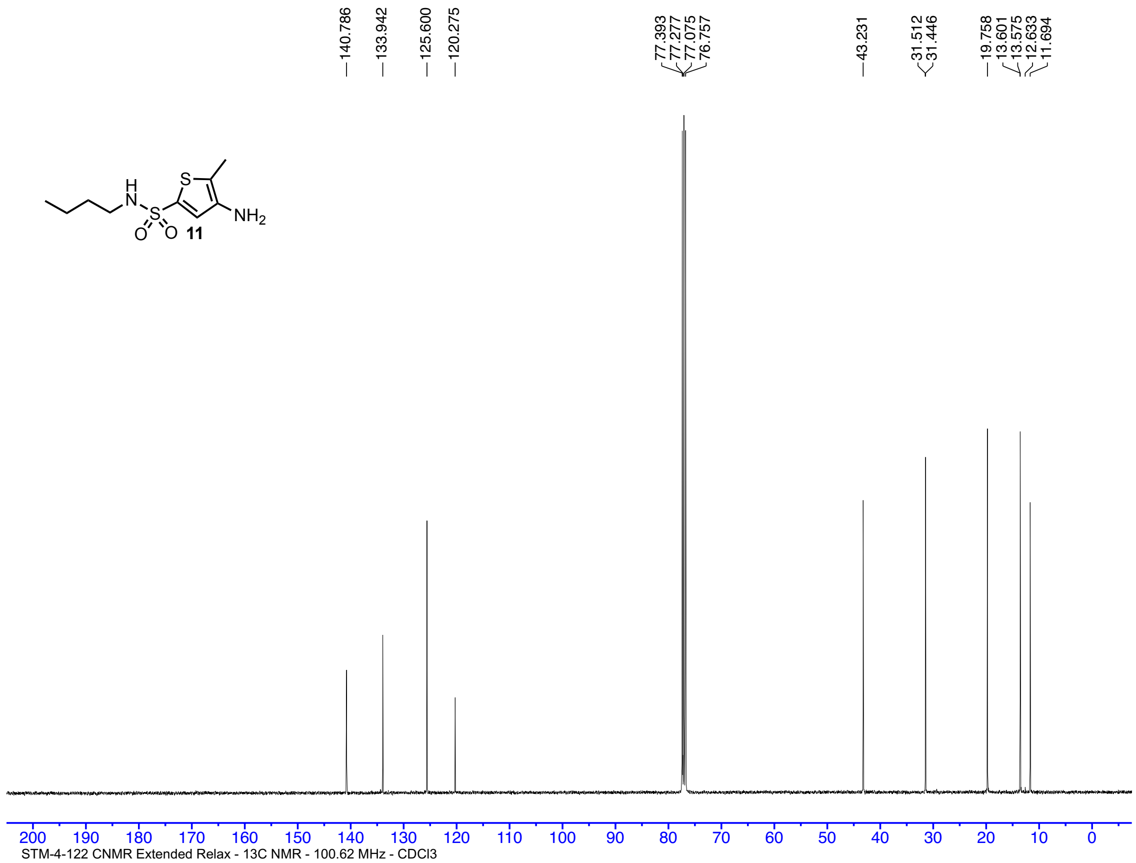

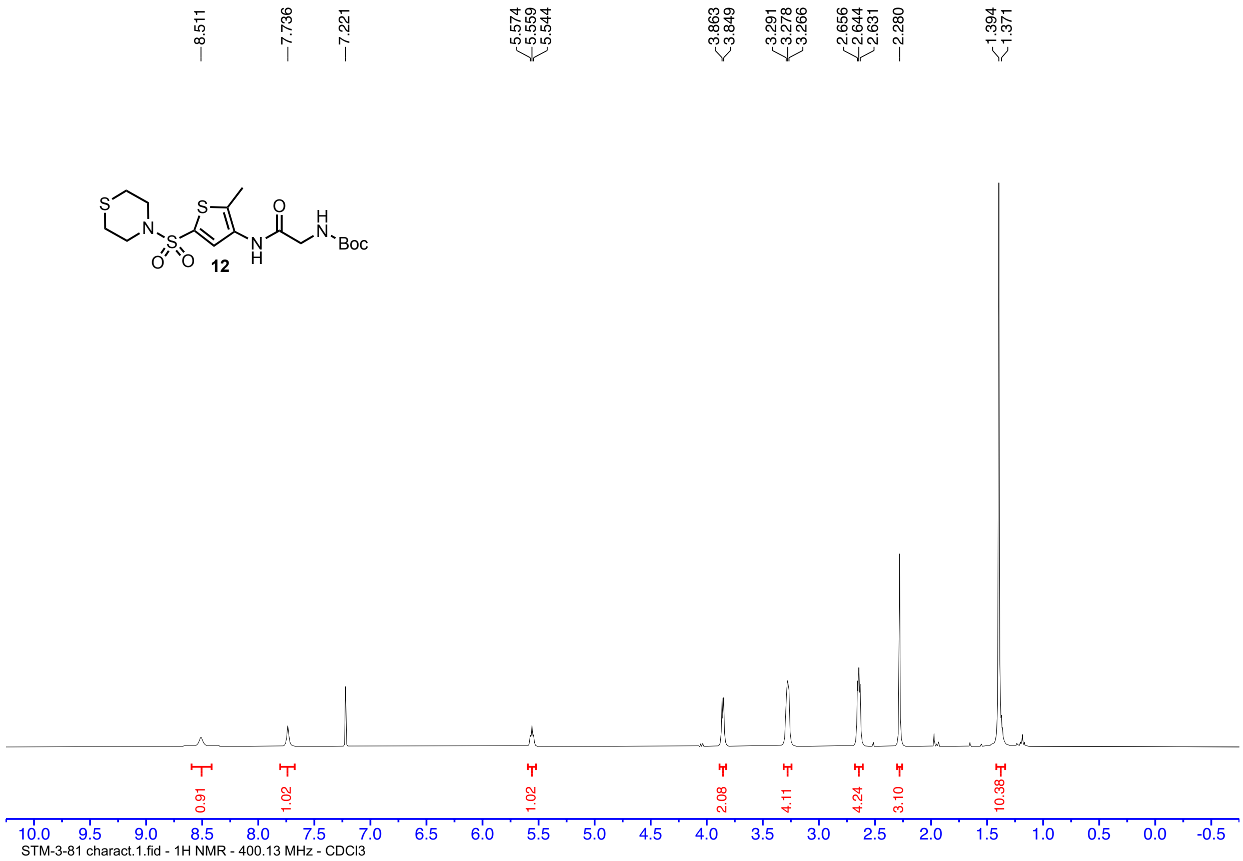
8.511
7.736
7.221
5.574
5.559
5.544
3.863
3.849
3.291
3.278
3.266
2.656
2.644
2.631
2.280
1.394
1.371
S
N
S
O
O
S
O
N
H
H
N
Boc
12
0.91
1.02
1.02
2.08
4.11
4.24
3.10
10.38
10.0 9.5 9.0 8.5 8.0 7.5 7.0 6.5 6.0 5.5 5.0 4.5 4.0 3.5 3.0 2.5 2.0 1.5 1.0 0.5 0.0 -0.5
STM-3-81 charact.1.fid - 1H NMR - 400.13 MHz - CDCl3

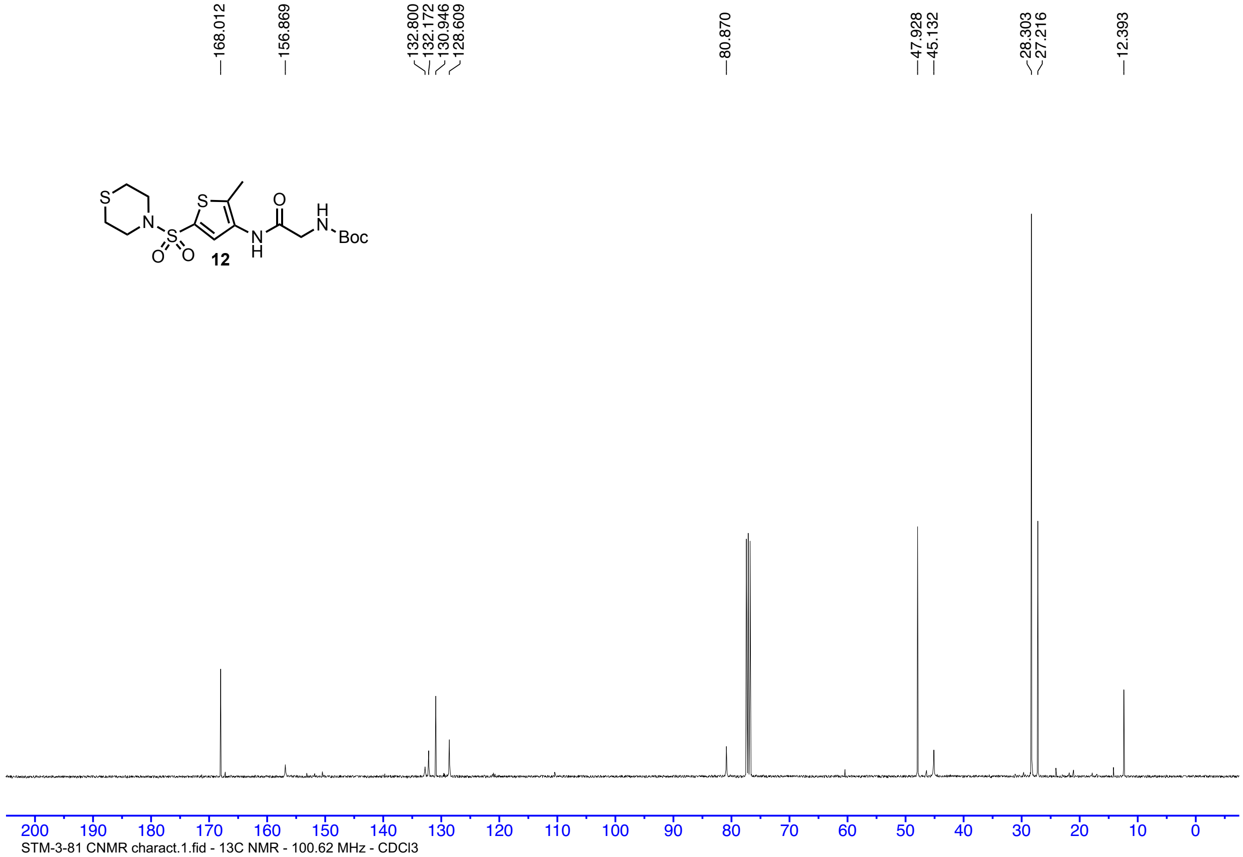
168.012
156.869
132.800
132.172
130.946
128.609
80.870
47.928
45.132
28.303
27.216
12.393
S
N
S
O
O
S
O
N
H
H
N
Boc
12
200 190 180 170 160 150 140 130 120 110 100 90 80 70 60 50 40 30 20 10 0
STM-3-81 CNMR charact.1.fid - 13C NMR - 100.62 MHz - CDCl3

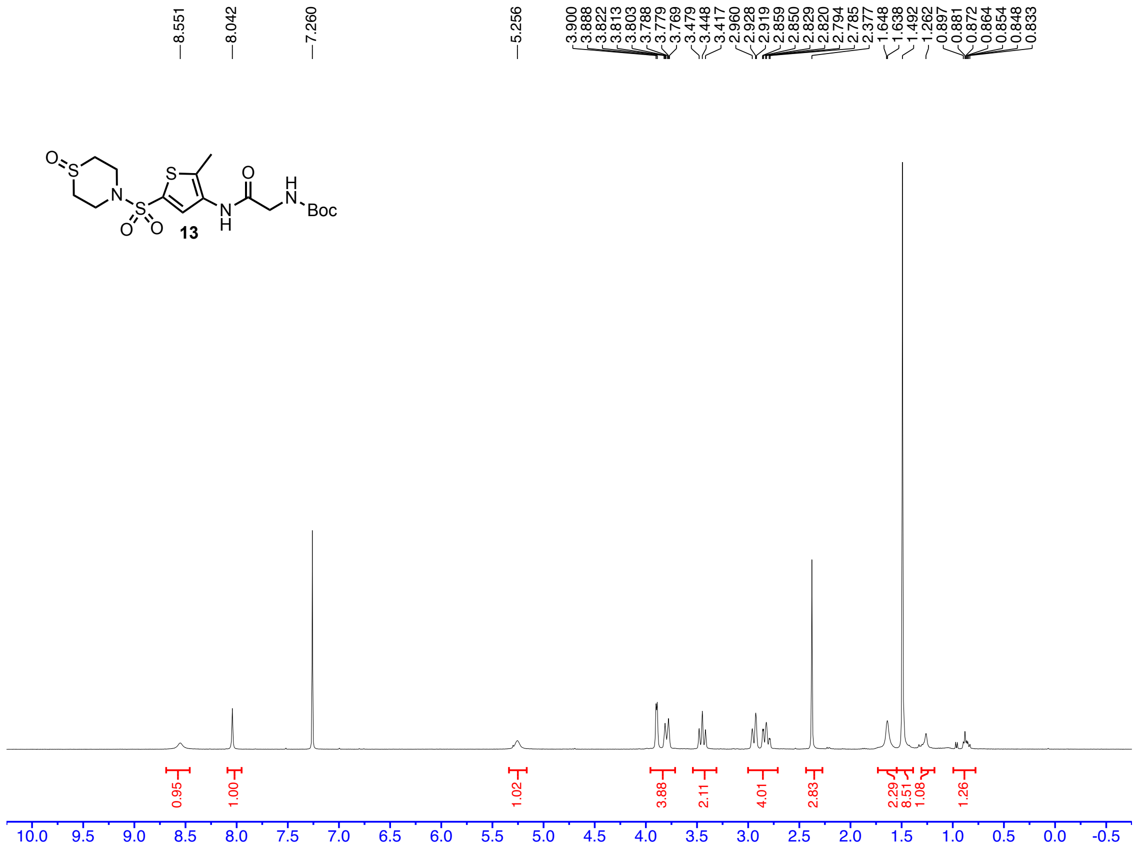

STM-4-60 Pure - 1H NMR - 400.13 MHz - CDCl3

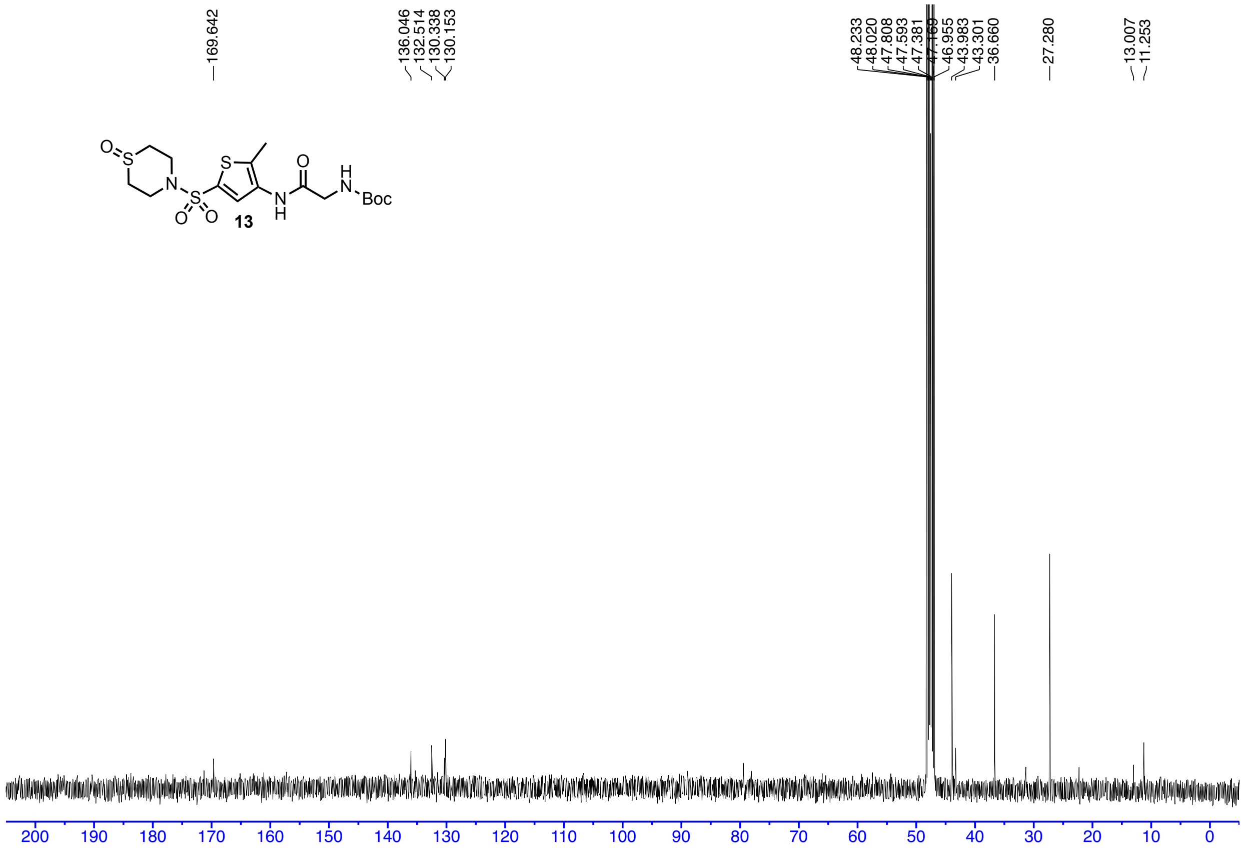

STM-4-60 MeOD CNMR.1.fid - 13C NMR - 100.62 MHz - MeOD

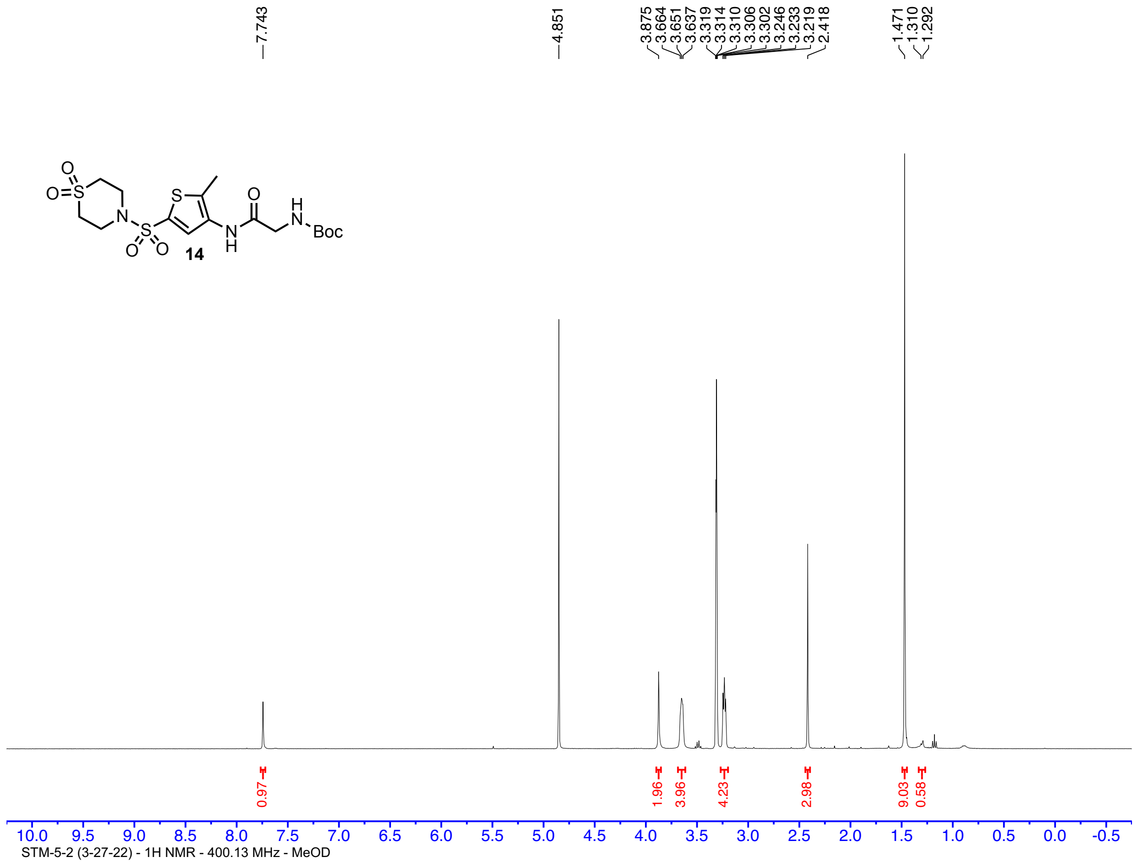
7.743
4.851
3.875
3.664
3.651
3.637
3.319
3.314
3.310
3.306
3.302
3.246
3.233
3.219
2.418
1.471
1.310
1.292
14
Boc
0.97
1.96
3.96
4.23
2.98
9.03
0.58
10.0 9.5 9.0 8.5 8.0 7.5 7.0 6.5 6.0 5.5 5.0 4.5 4.0 3.5 3.0 2.5 2.0 1.5 1.0 0.5 0.0 -0.5
STM-5-2 (3-27-22) - 1H NMR - 400.13 MHz - MeOD

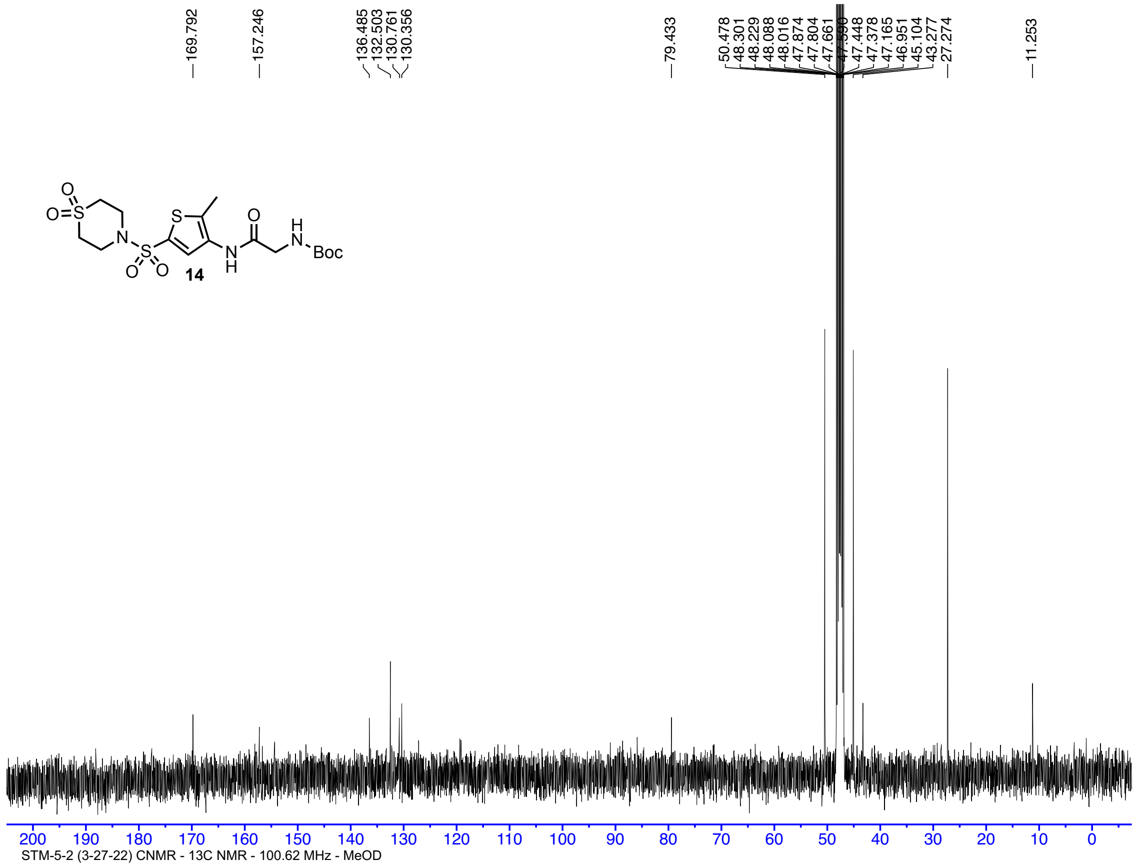
169.792
157.246
136.485
132.503
130.761
130.356
79.433
50.478
48.301
48.229
48.088
48.016
47.874
47.804
47.661
47.448
47.378
47.165
46.951
45.104
43.277
27.274
11.253
14
Boc
200 190 180 170 160 150 140 130 120 110 100 90 80 70 60 50 40 30 20 10 0
STM-5-2 (3-27-22) CNMR - 13C NMR - 100.62 MHz - MeOD

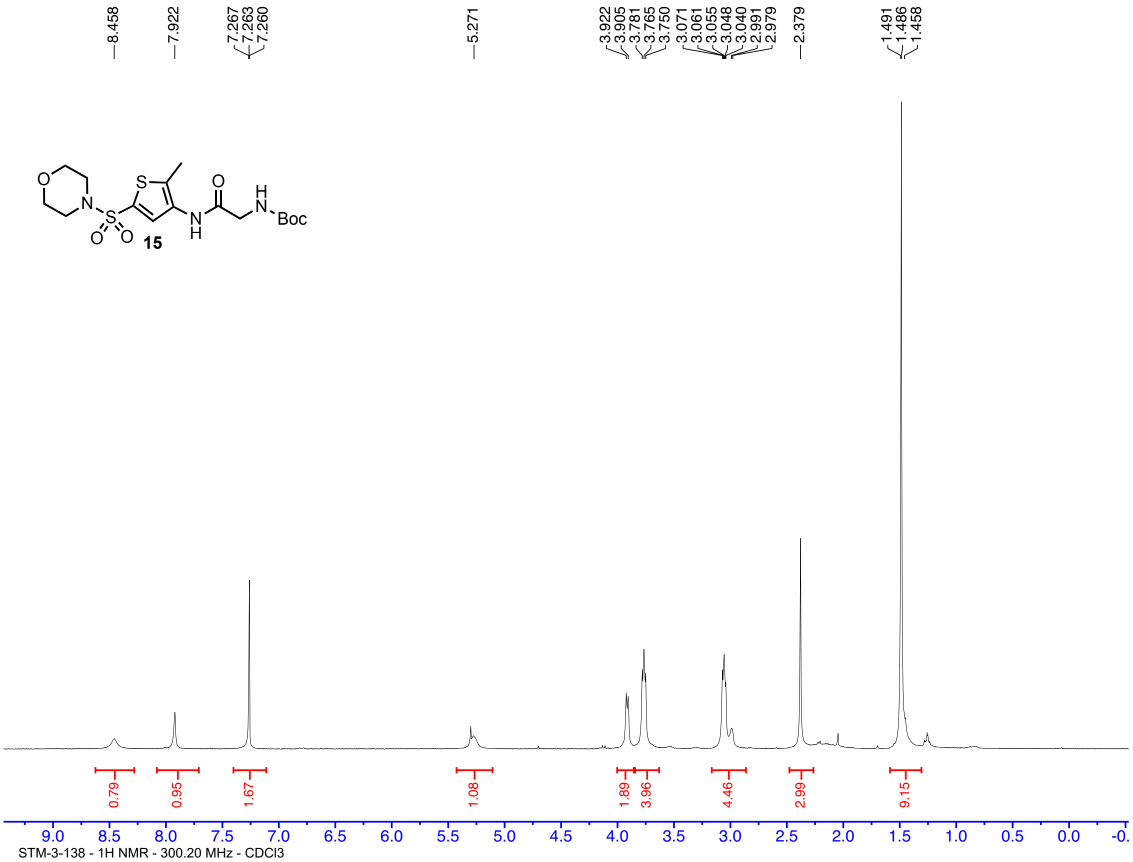

8.458
7.922
7.267
7.263
7.260
5.271
3.922
3.905
3.781
3.765
3.750
3.071
3.061
3.055
3.048
3.040
2.991
2.979
2.379
1.491
1.486
1.458
O
N
S
O
O
N
H
O
H
N
Boc
15
0.79
0.95
1.67
1.08
1.89
3.96
4.46
2.99
9.15
9.0 8.5 8.0 7.5 7.0 6.5 6.0 5.5 5.0 4.5 4.0 3.5 3.0 2.5 2.0 1.5 1.0 0.5 0.0 -0.
STM-3-138 - 1H NMR - 300.20 MHz - CDCl3

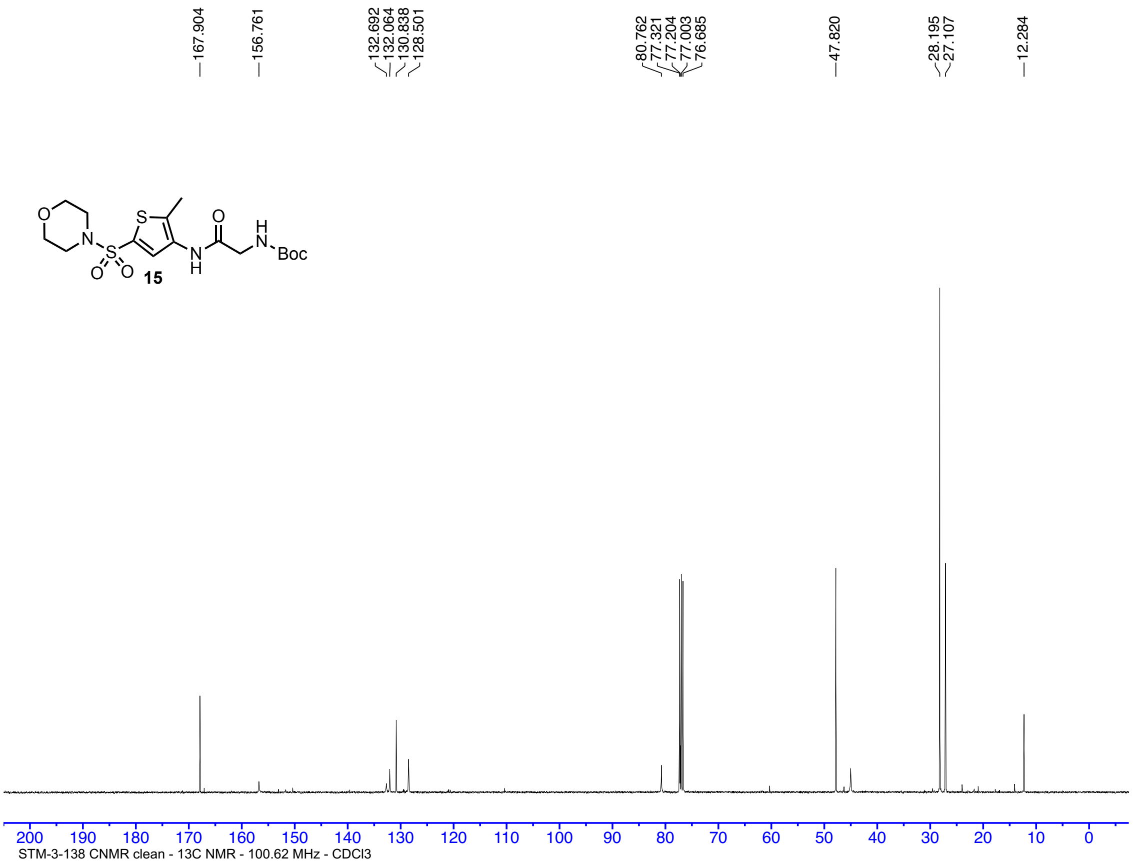

167.904
156.761
132.692
132.064
130.838
128.501
80.762
77.321
77.204
77.003
76.685
47.820
28.195
27.107
12.284
O
N
S
O
O
N
H
O
H
N
Boc
15
200 190 180 170 160 150 140 130 120 110 100 90 80 70 60 50 40 30 20 10 0
STM-3-138 CNMR clean - 13C NMR - 100.62 MHz - CDCl3

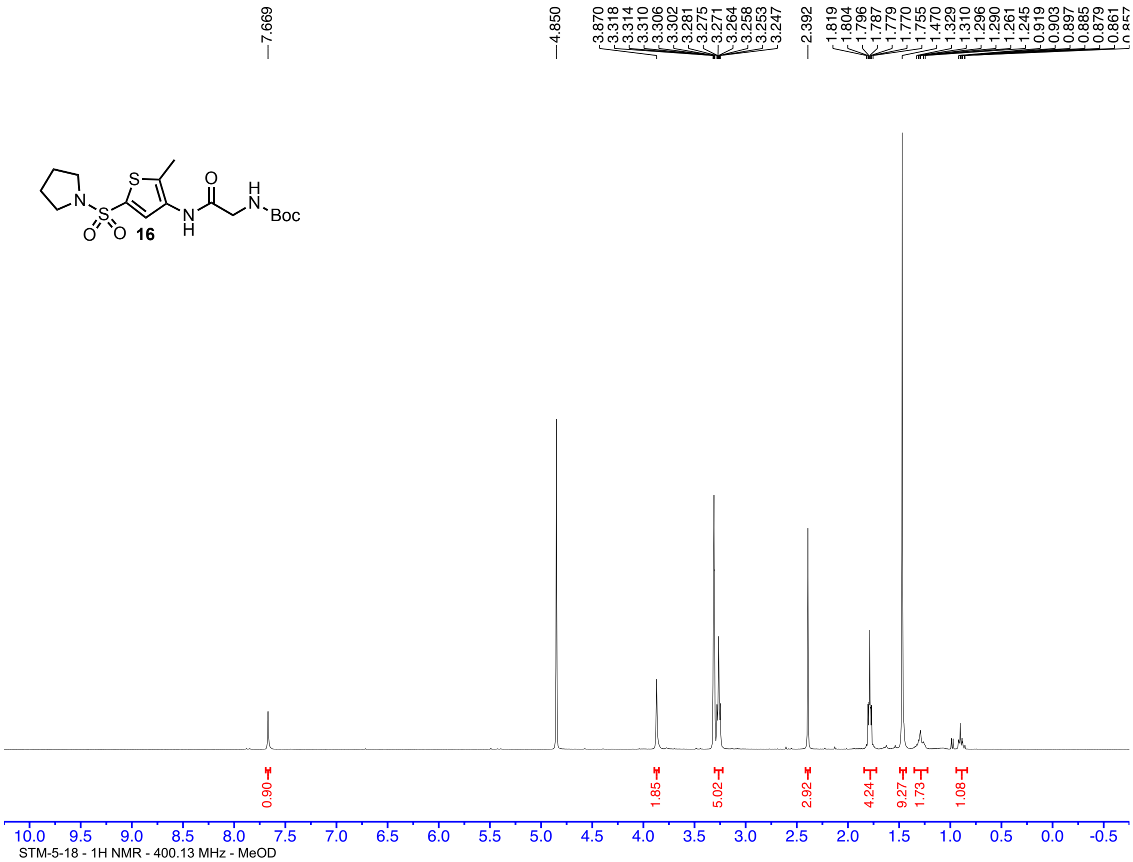
7.669
4.850
3.870
3.318
3.314
3.310
3.306
3.302
3.281
3.275
3.271
3.264
3.258
3.253
3.247
2.392
1.819
1.804
1.796
1.787
1.779
1.770
1.755
1.470
1.329
1.310
1.296
1.290
1.261
1.245
0.919
0.903
0.897
0.885
0.879
0.861
16
Boc
0.90
1.85
5.02
2.92
4.24
9.27
1.73
1.08
10.0 9.5 9.0 8.5 8.0 7.5 7.0 6.5 6.0 5.5 5.0 4.5 4.0 3.5 3.0 2.5 2.0 1.5 1.0 0.5 0.0 -0.5
STM-5-18 - 1H NMR - 400.13 MHz - MeOD

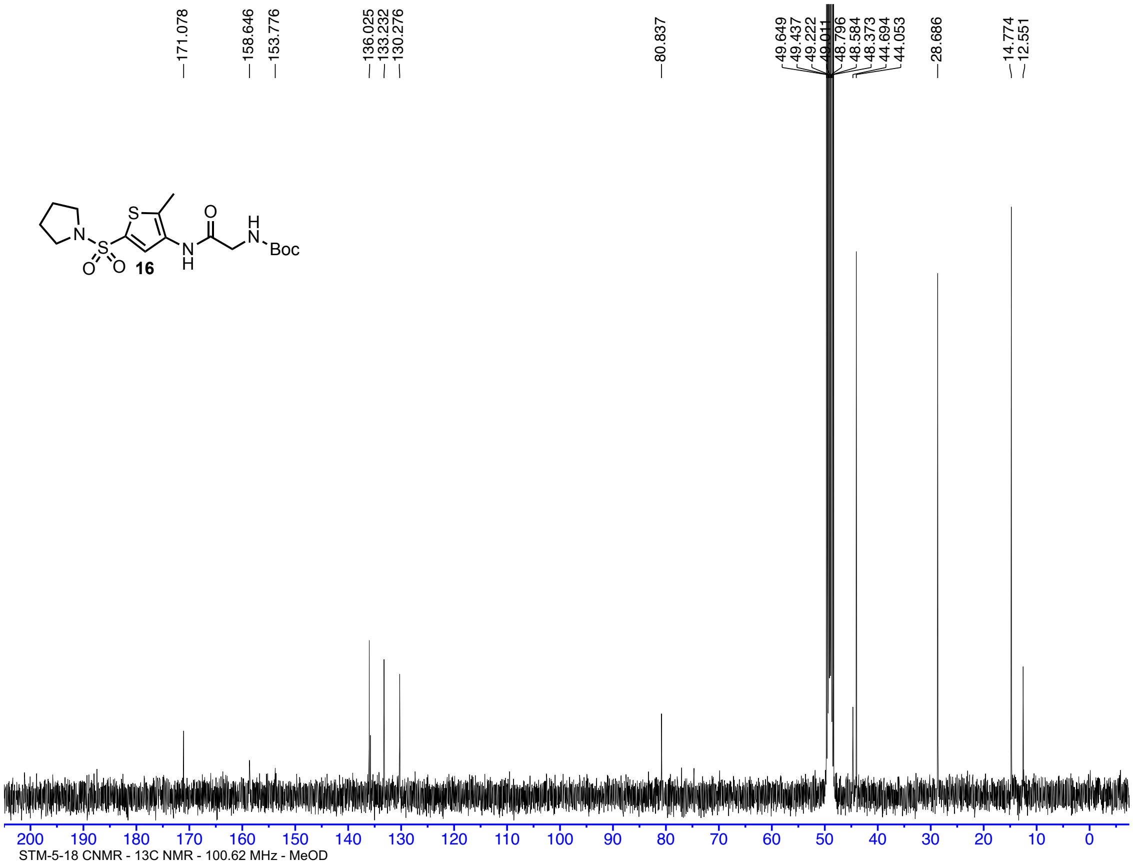
171.078
158.646
153.776
136.025
133.232
130.276
80.837
49.649
49.437
49.222
49.011
48.796
48.584
48.373
44.694
44.053
28.686
14.774
12.551
16
Boc
200 190 180 170 160 150 140 130 120 110 100 90 80 70 60 50 40 30 20 10 0
STM-5-18 CNMR - 13C NMR - 100.62 MHz - MeOD

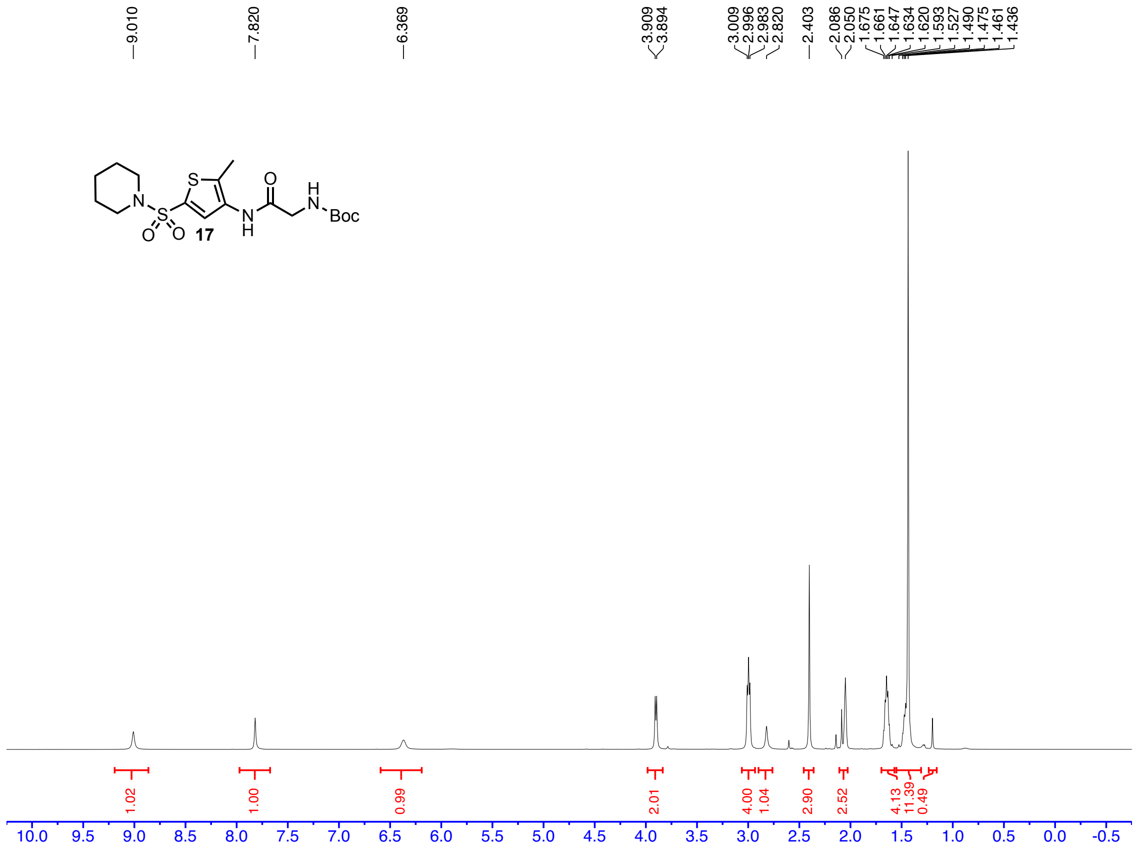

STM-5-26 (7-19-22) - 1H NMR - 400.13 MHz - Acetone

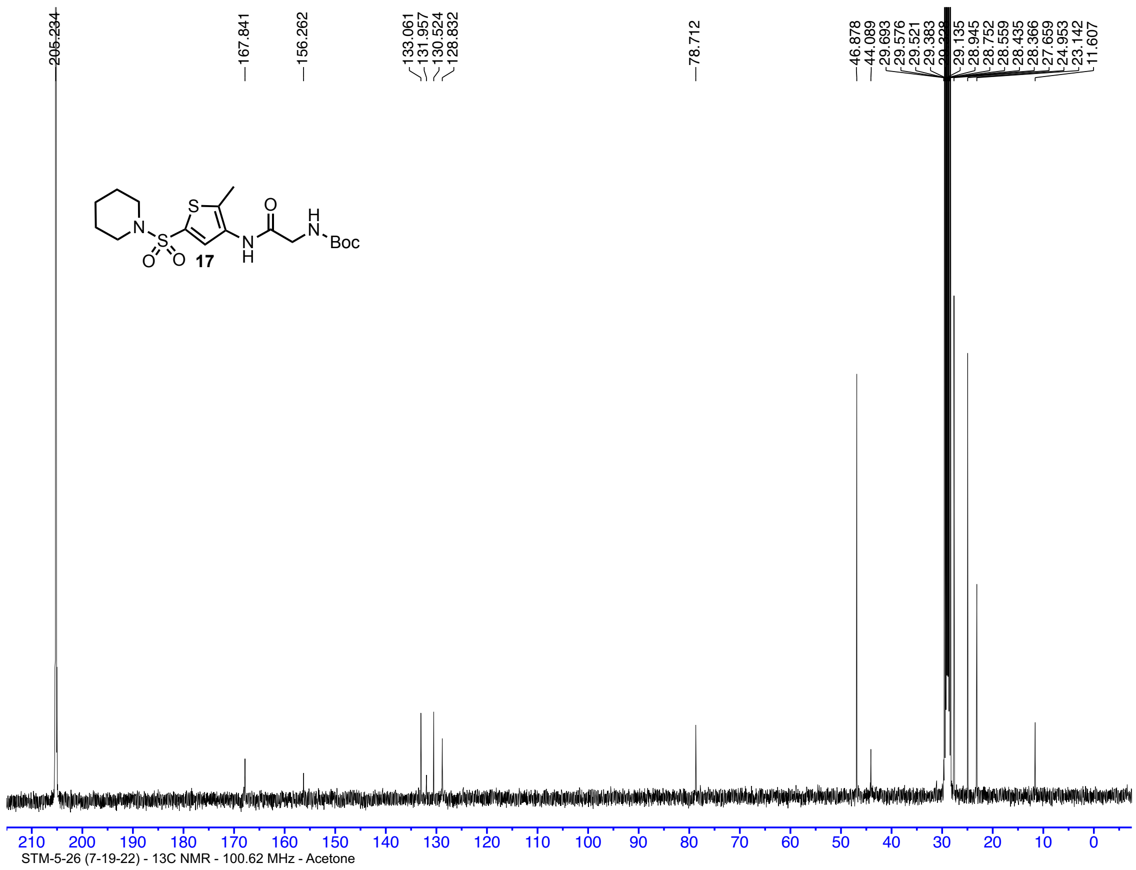

STM-5-26 (7-19-22) - 13C NMR - 100.62 MHz - Acetone

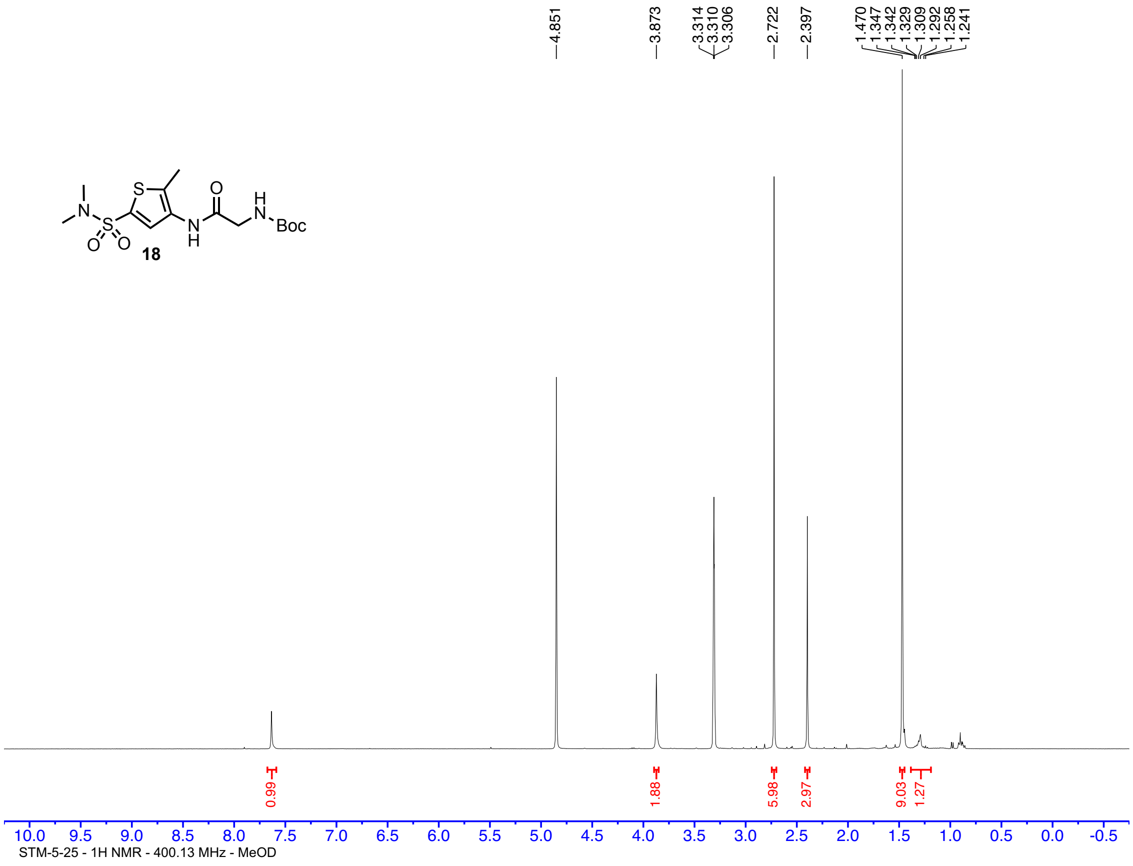
4.851
3.873
3.314
3.310
3.306
2.722
2.397
1.470
1.347
1.342
1.329
1.309
1.292
1.258
1.241
18
Boc
0.99
1.88
5.98
2.97
9.03
1.27
10.0 9.5 9.0 8.5 8.0 7.5 7.0 6.5 6.0 5.5 5.0 4.5 4.0 3.5 3.0 2.5 2.0 1.5 1.0 0.5 0.0 -0.5
STM-5-25 - 1H NMR - 400.13 MHz - MeOD

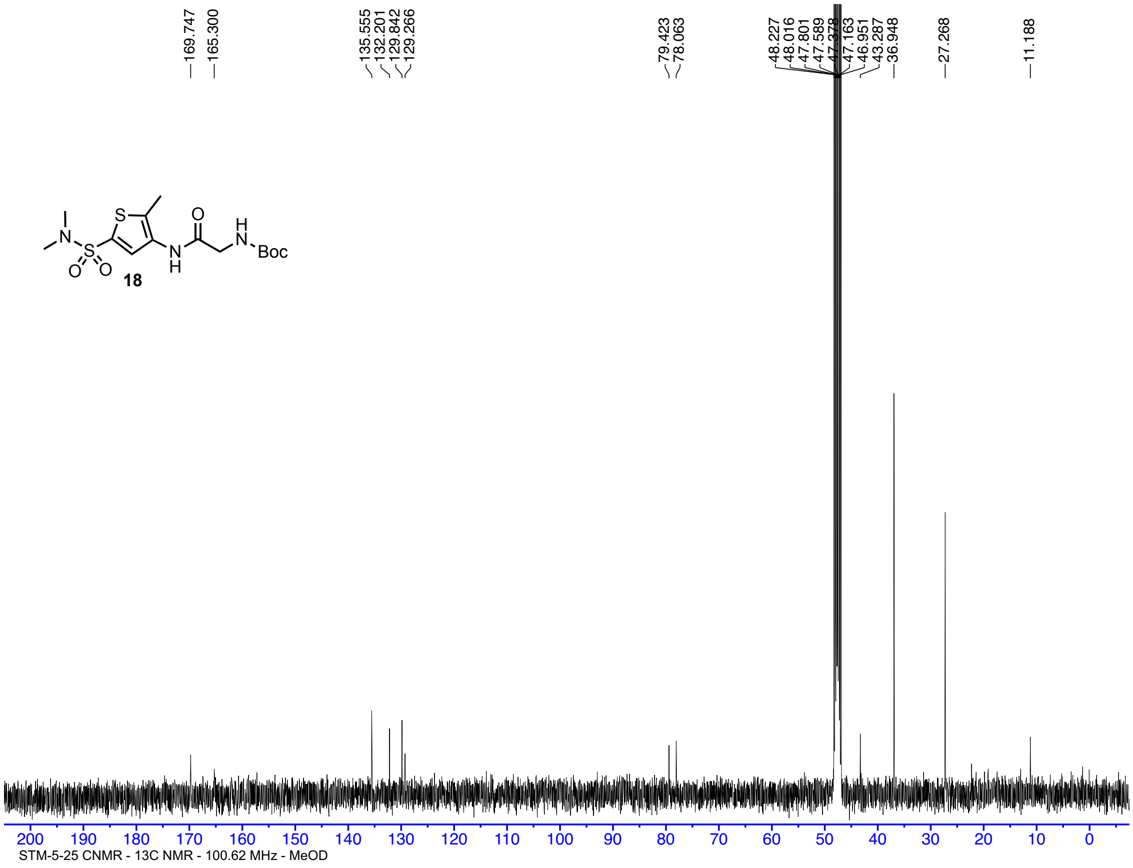
169.747
165.300
135.555
132.201
129.842
129.266
79.423
78.063
48.227
48.016
47.801
47.589
47.378
47.163
46.951
43.287
36.948
27.268
11.188
18
Boc
200 190 180 170 160 150 140 130 120 110 100 90 80 70 60 50 40 30 20 10 0
STM-5-25 CNMR - 13C NMR - 100.62 MHz - MeOD

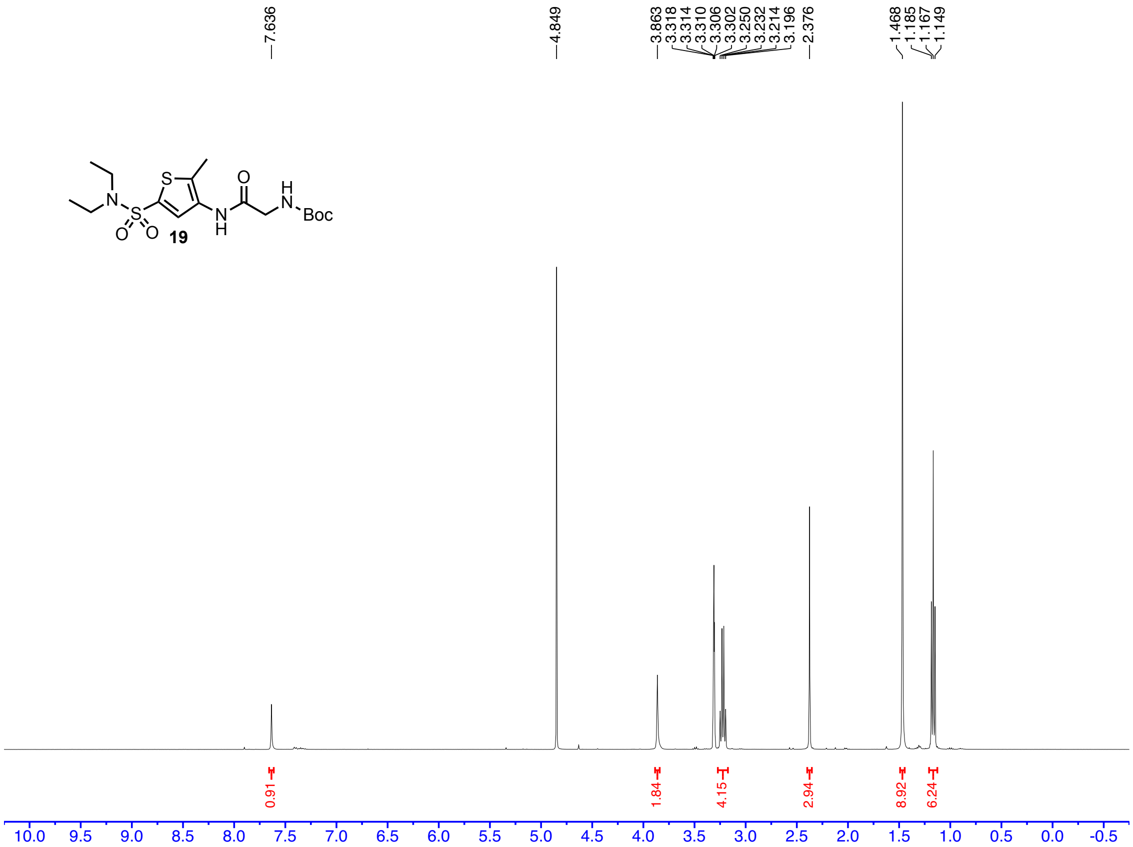
STM-4-132 - 1H NMR - 400.13 MHz - MeOD

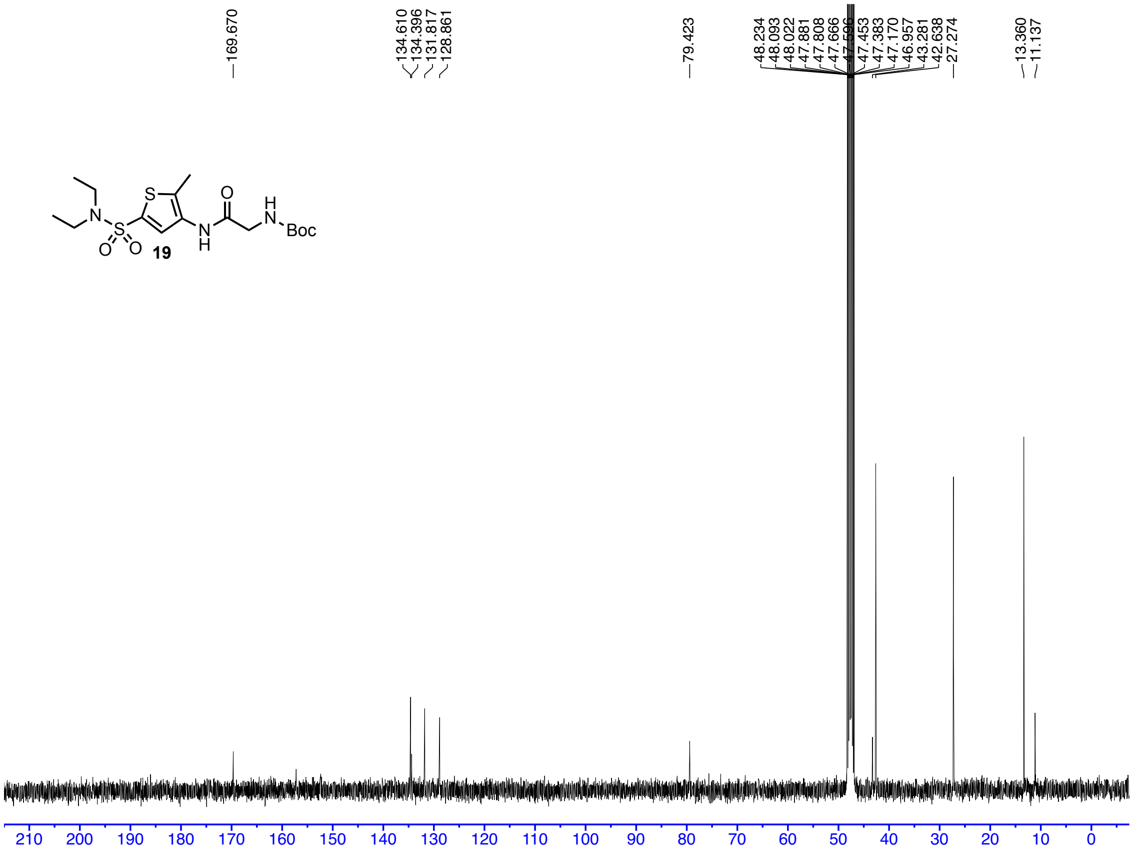
STM-4-132 MeOD CNMR - 13C NMR - 100.62 MHz - MeOD

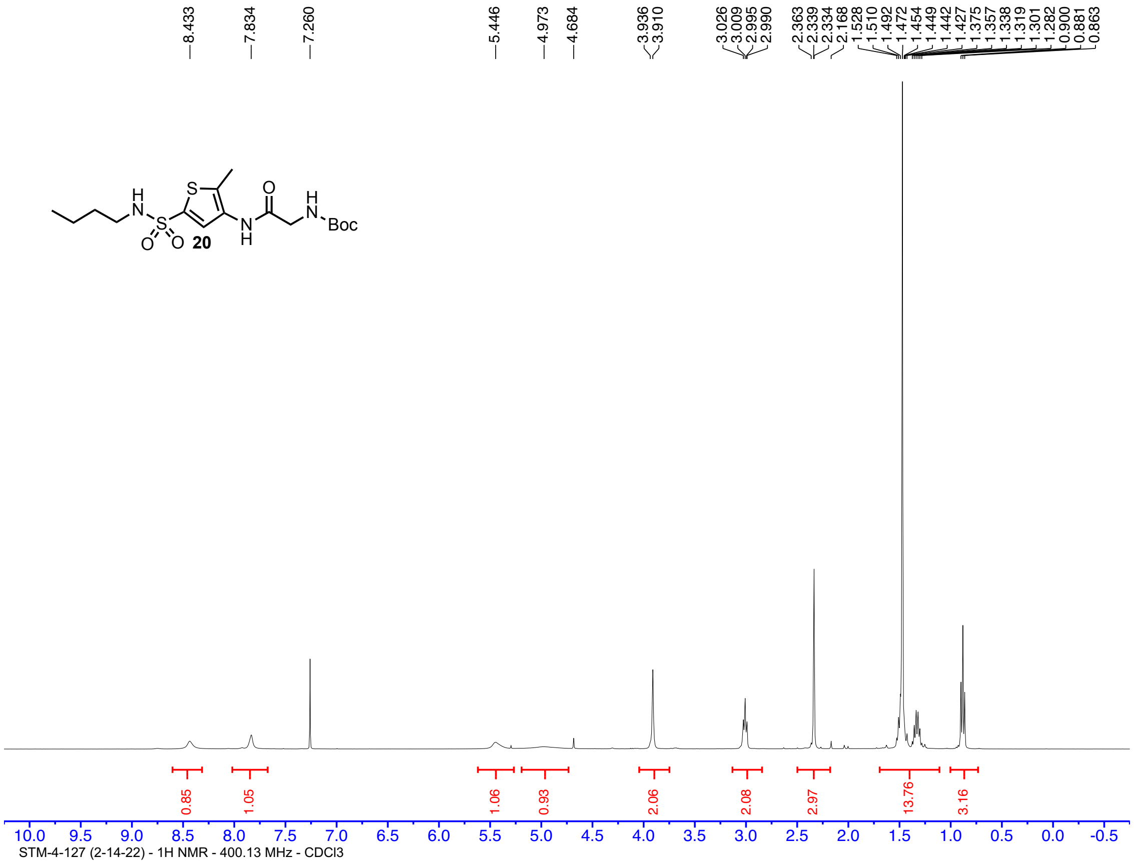
8.433
7.834
7.260
5.446
4.973
4.684
3.936
3.910
3.026
3.009
2.995
2.990
2.363
2.339
2.334
2.168
1.528
1.510
1.492
1.472
1.454
1.449
1.442
1.427
1.375
1.357
1.338
1.319
1.301
1.282
0.900
0.881
0.863
20
Boc
0.85
1.05
1.06
0.93
2.06
2.08
2.97
13.76
3.16
10.0 9.5 9.0 8.5 8.0 7.5 7.0 6.5 6.0 5.5 5.0 4.5 4.0 3.5 3.0 2.5 2.0 1.5 1.0 0.5 0.0 -0.5
STM-4-127 (2-14-22) - 1H NMR - 400.13 MHz - CDCl3

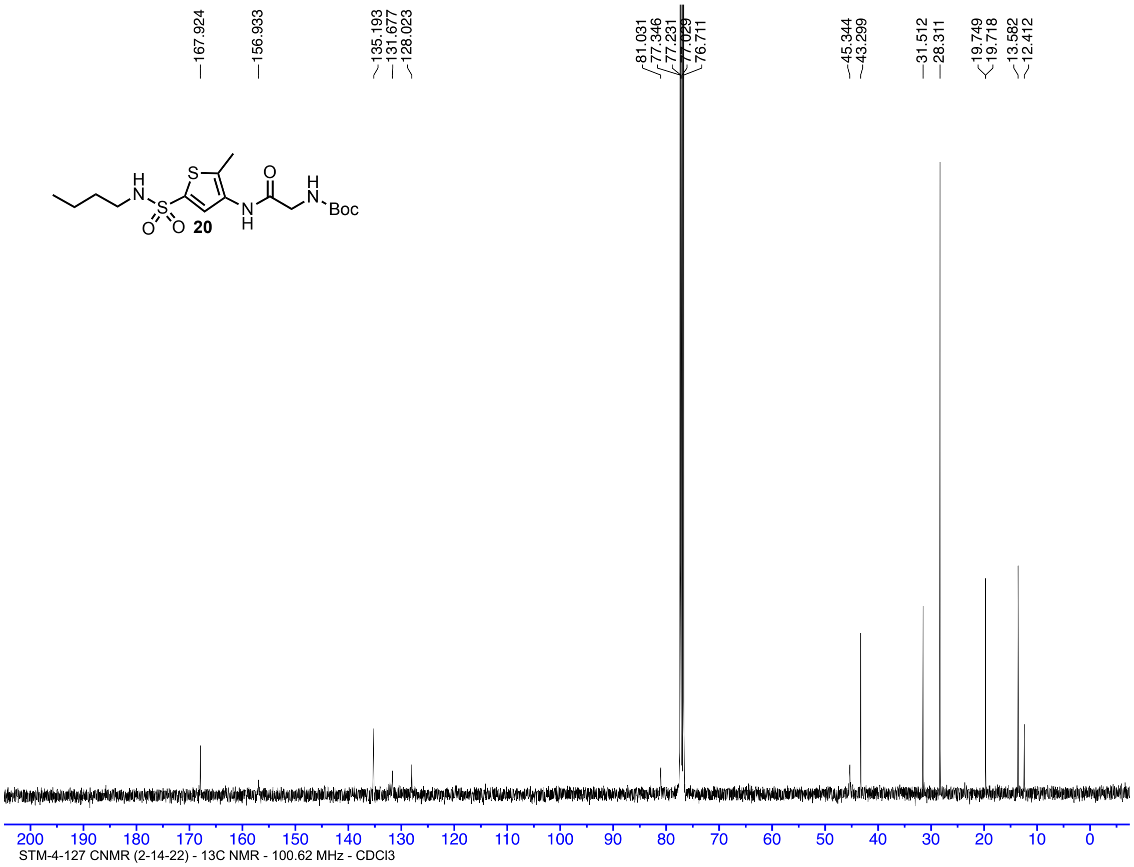
167.924
156.933
135.193
131.677
128.023
81.031
77.346
77.231
76.711
45.344
43.299
31.512
28.311
19.749
19.718
13.582
12.412
20
Boc
200 190 180 170 160 150 140 130 120 110 100 90 80 70 60 50 40 30 20 10 0
STM-4-127 CNMR (2-14-22) - 13C NMR - 100.62 MHz - CDCl3

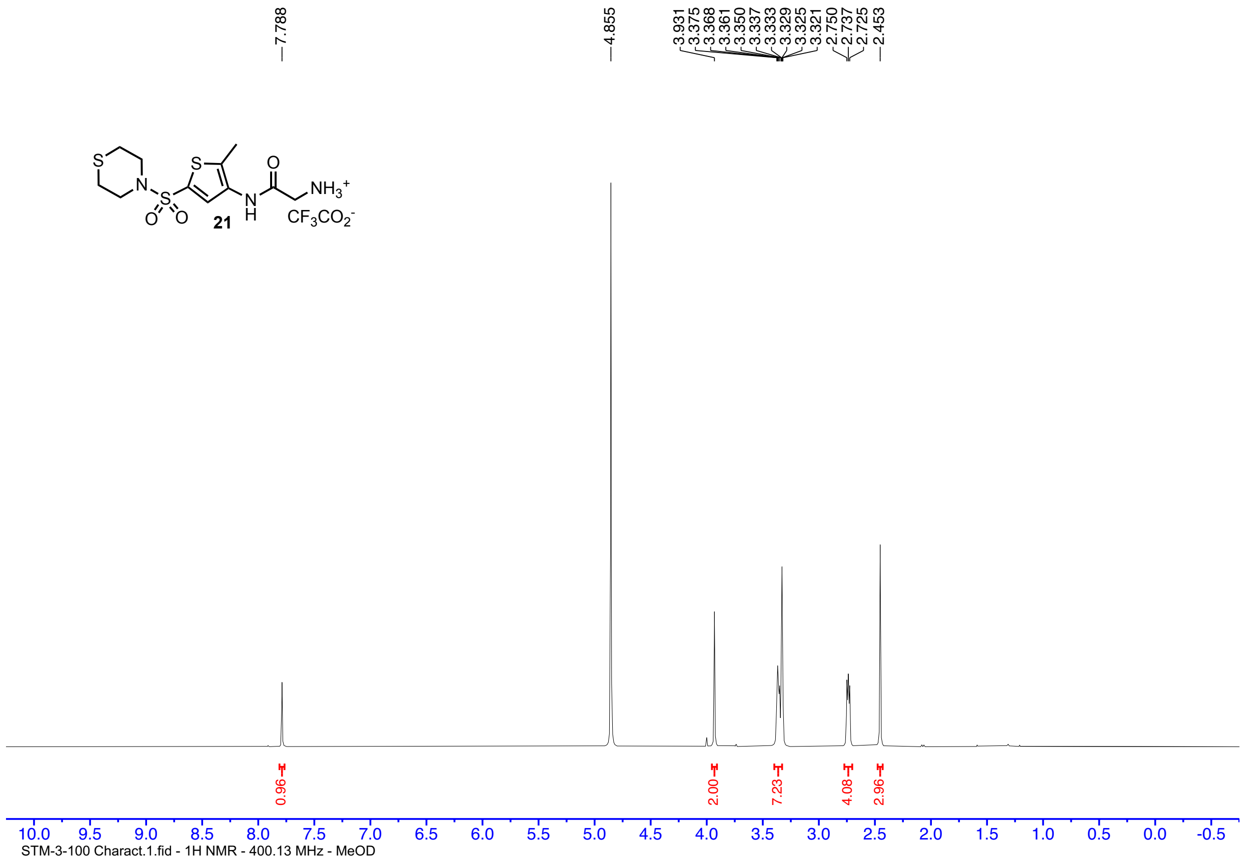
7.788
4.855
3.931
3.375
3.368
3.361
3.350
3.337
3.333
3.329
3.325
3.321
2.750
2.737
2.725
2.453
21
0.96
2.00
7.23
4.08
2.96
STM-3-100 Charact.1.fid - 1H NMR - 400.13 MHz - MeOD

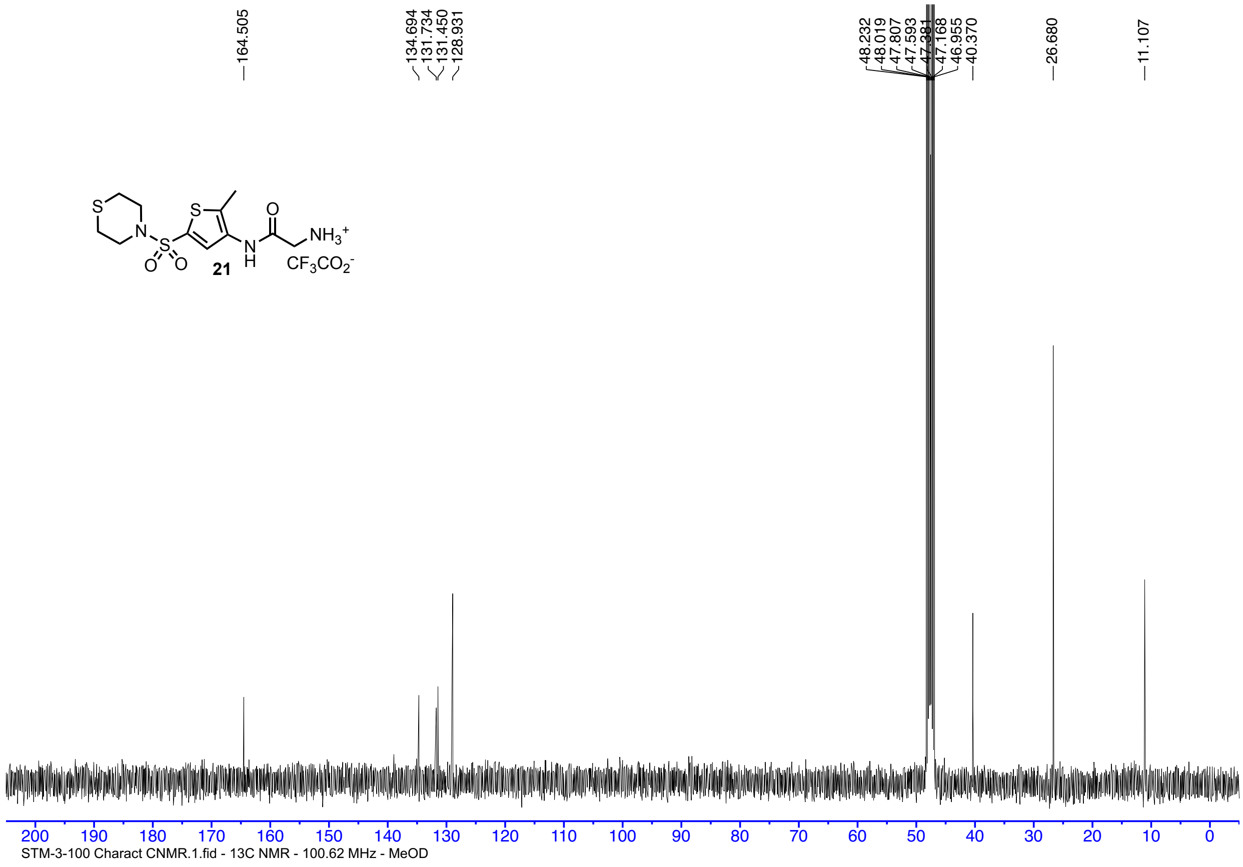
164.505
134.694
131.734
131.450
128.931
48.232
48.019
47.807
47.593
47.381
47.168
46.955
40.370
26.680
11.107
21
STM-3-100 Charact CNMR.1.fid - 13C NMR - 100.62 MHz - MeOD

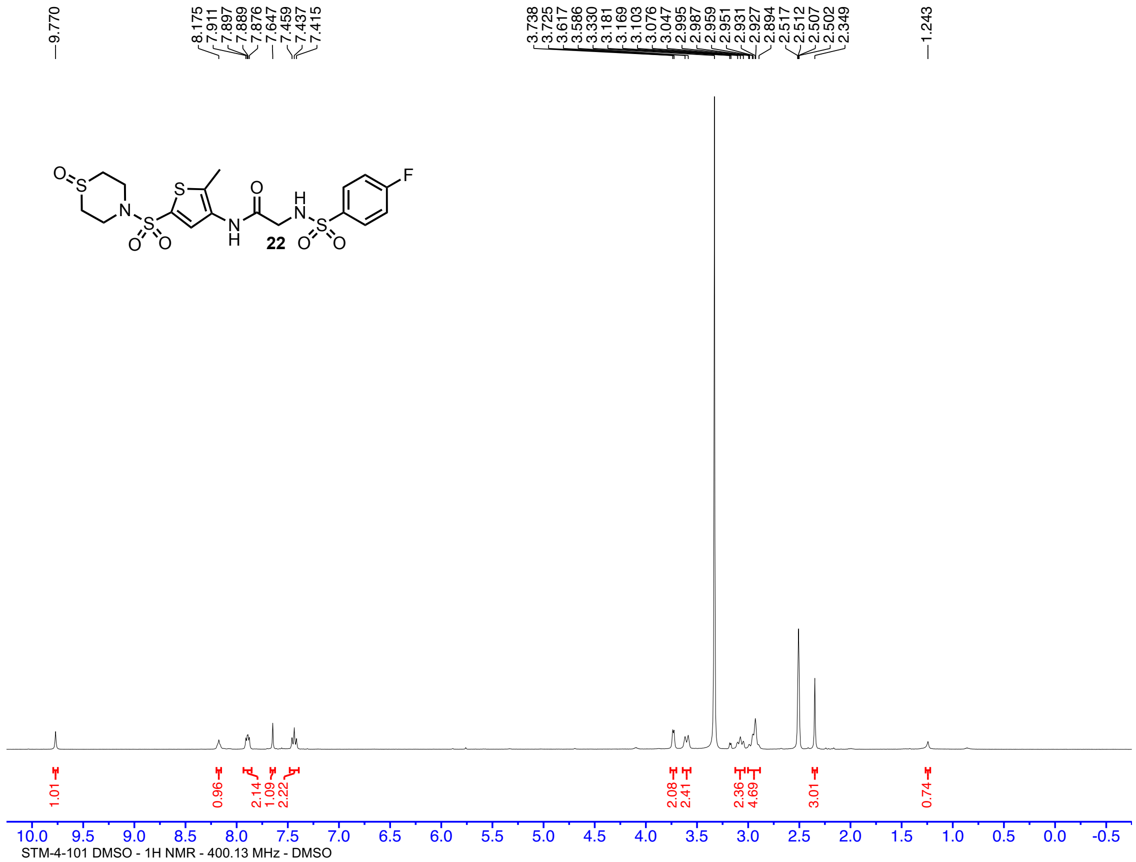

9.770
8.175
7.911
7.897
7.889
7.876
7.647
7.459
7.437
7.415
3.738
3.725
3.617
3.586
3.330
3.181
3.169
3.103
3.076
3.047
2.995
2.987
2.959
2.951
2.931
2.927
2.894
2.517
2.512
2.507
2.502
2.349
1.243
22
1.01
0.96
2.14
1.09
2.22
2.08
2.41
2.36
4.69
3.01
0.74
STM-4-101 DMSO - 1H NMR - 400.13 MHz - DMSO

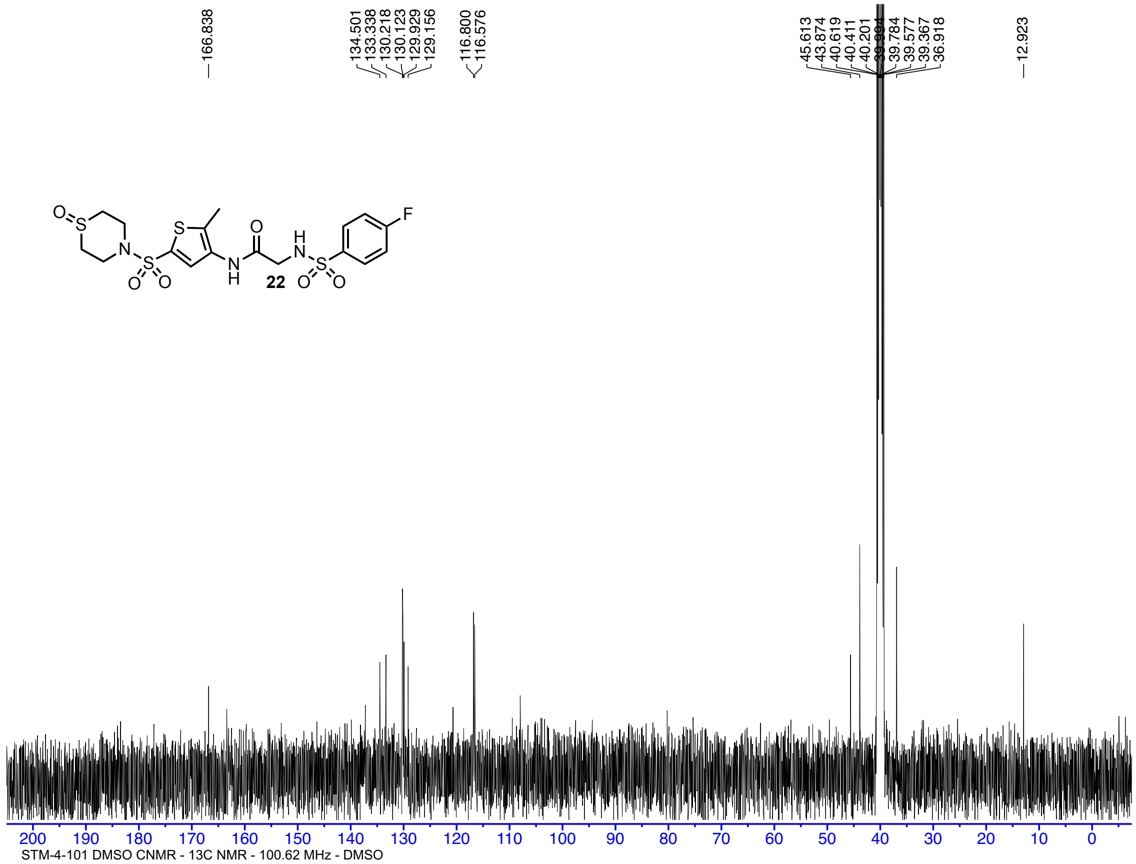

166.838
134.501
133.338
130.218
130.123
129.929
129.156
116.800
116.576
45.613
43.874
40.619
40.411
40.201
39.784
39.577
39.367
36.918
12.923
22
STM-4-101 DMSO CNMR - 13C NMR - 100.62 MHz - DMSO

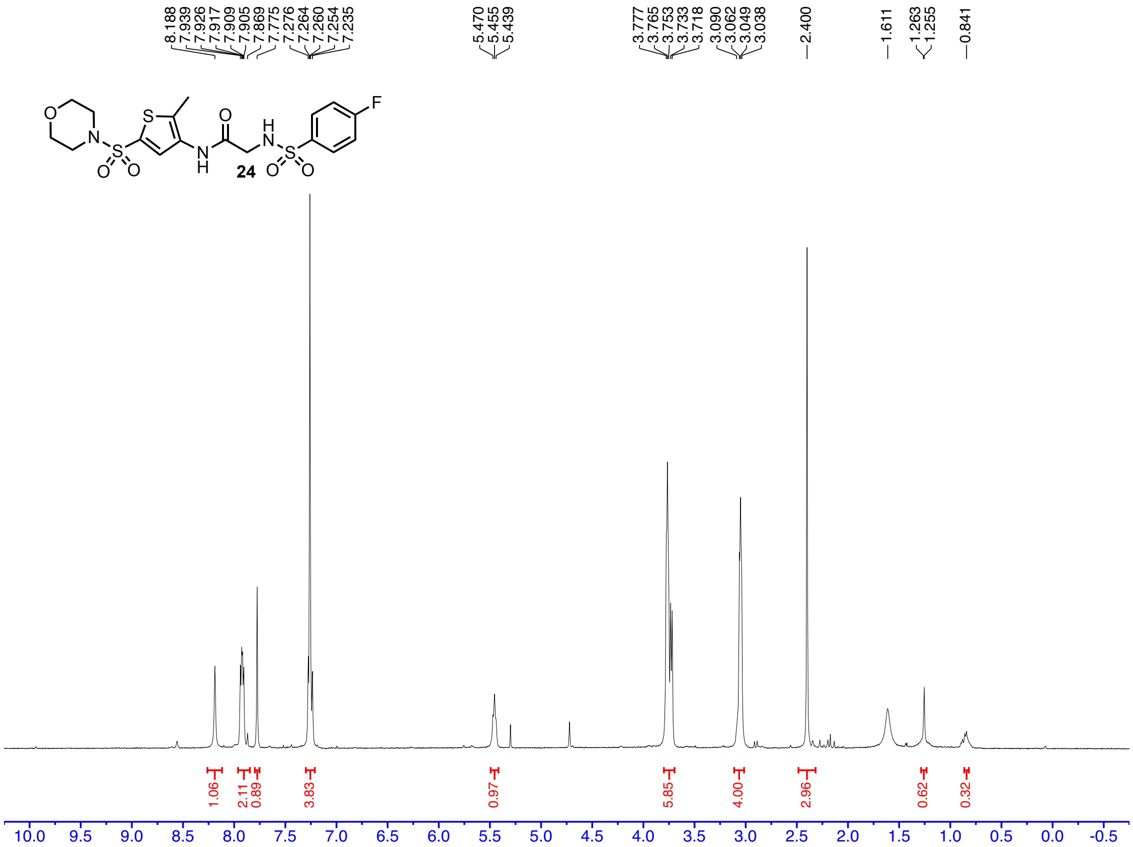

STM-3-144 Pumped - 1H NMR - 400.13 MHz - CDCl3

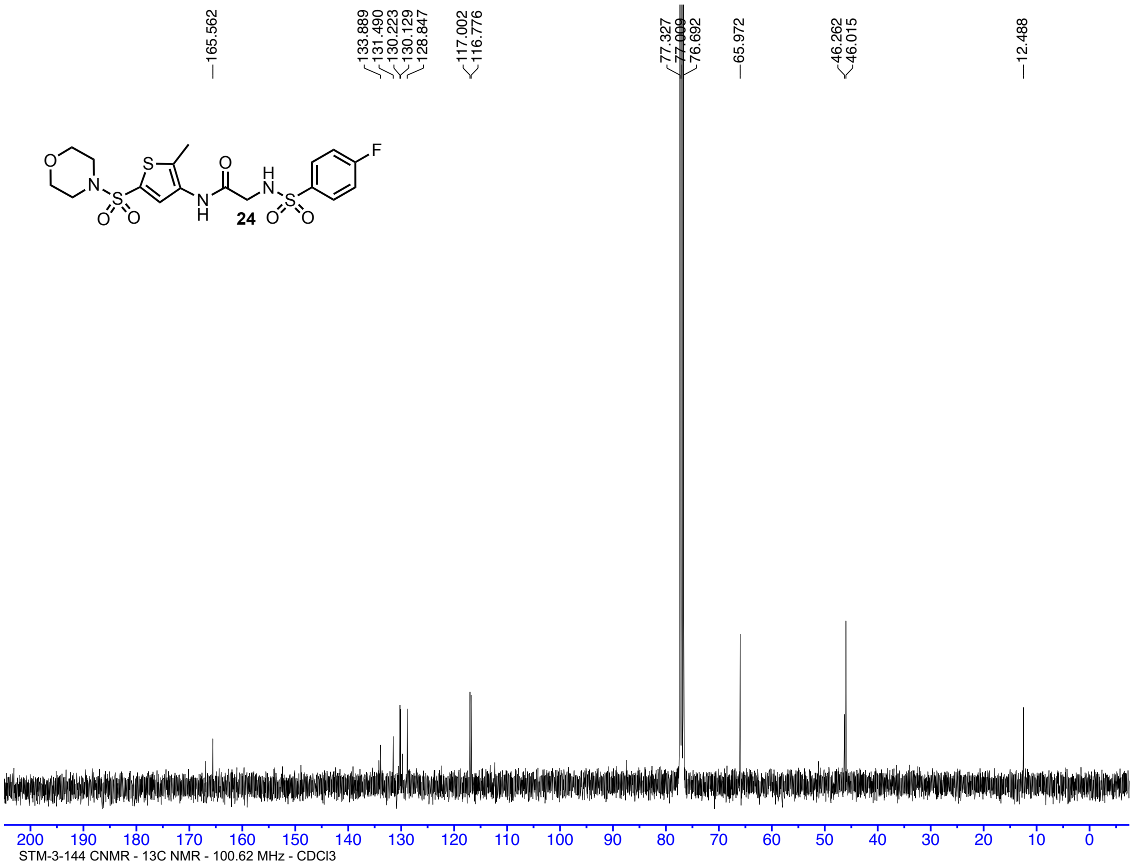

STM-3-144 CNMR - 13C NMR - 100.62 MHz - CDCl3

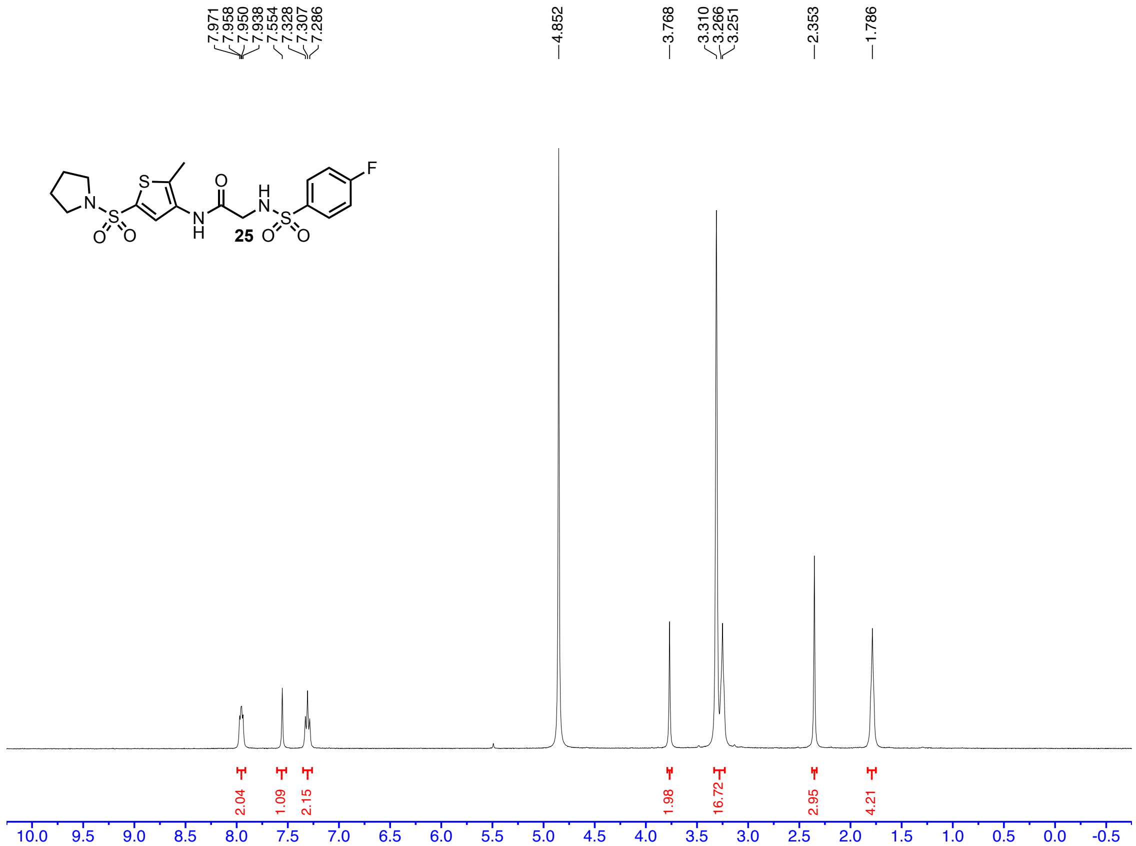

STM-5-29 (4-15-22) - 1H NMR - 400.13 MHz - MeOD

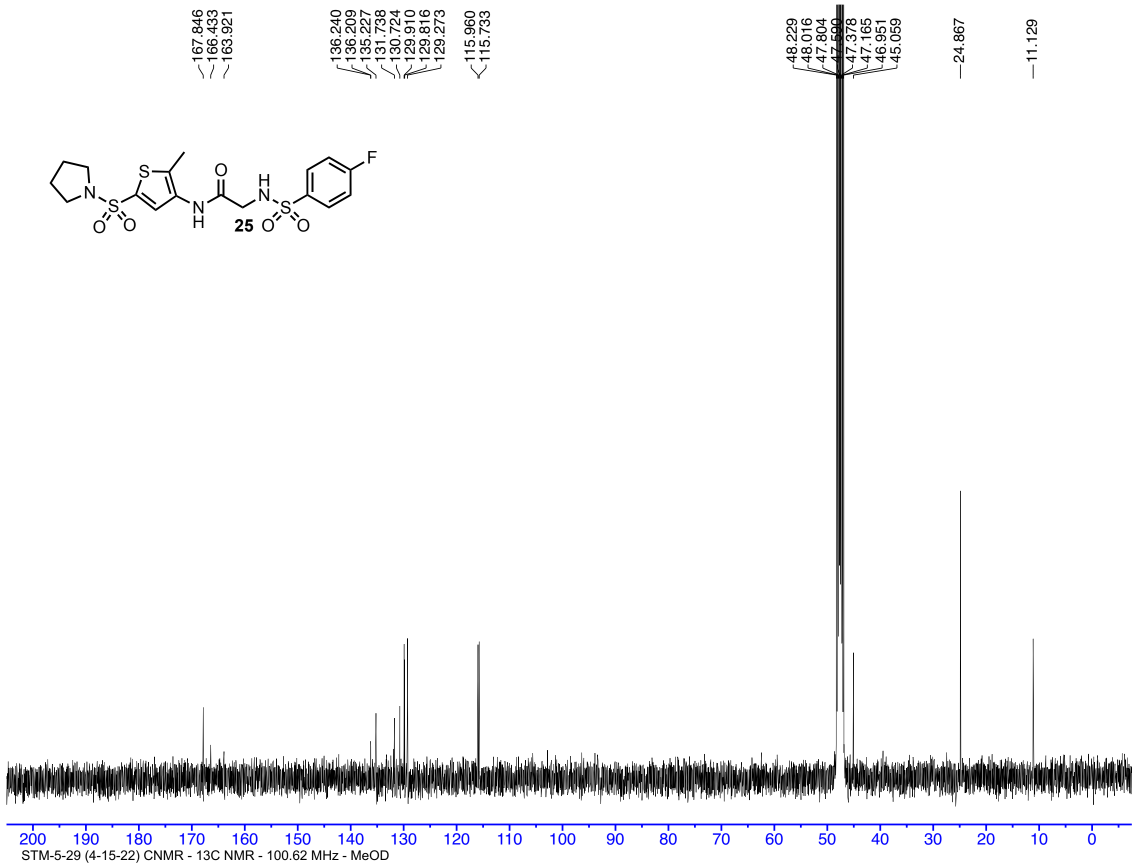

STM-5-29 (4-15-22) CNMR - 13C NMR - 100.62 MHz - MeOD

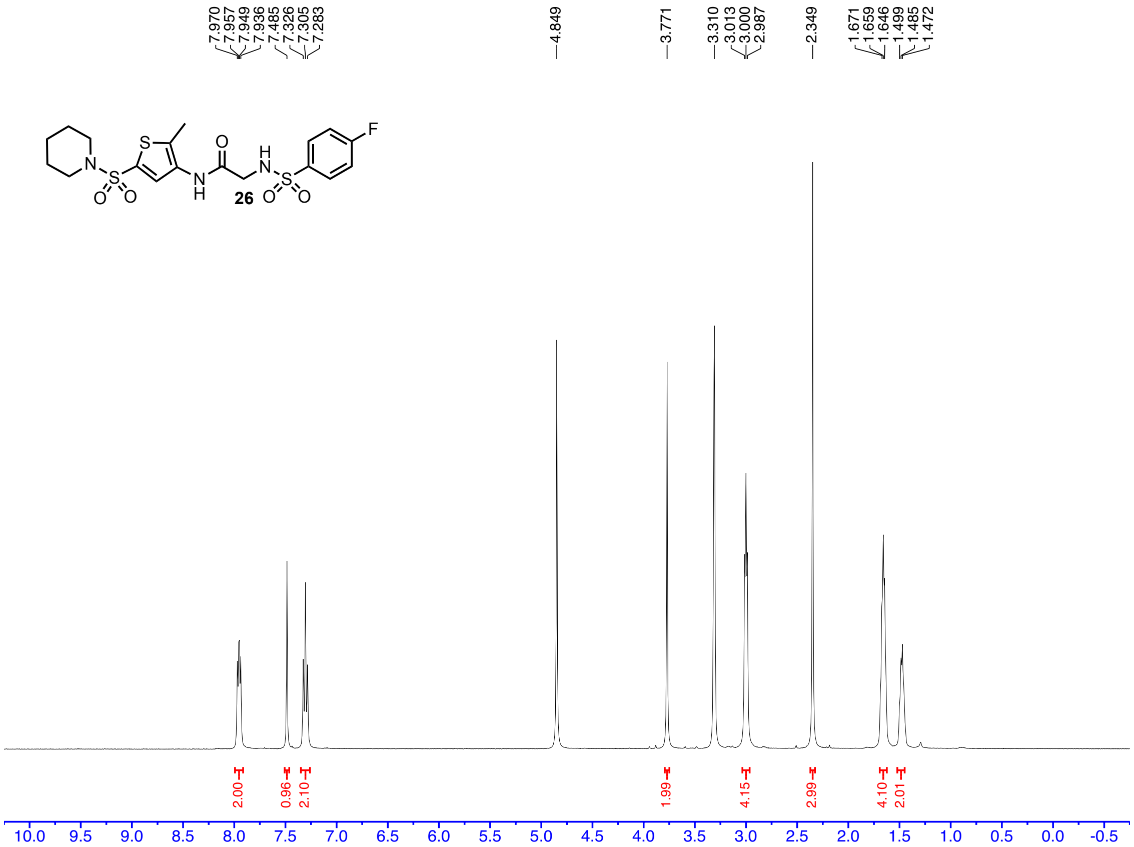

STM-4-23 recrystallized - 1H NMR - 400.13 MHz - MeOD

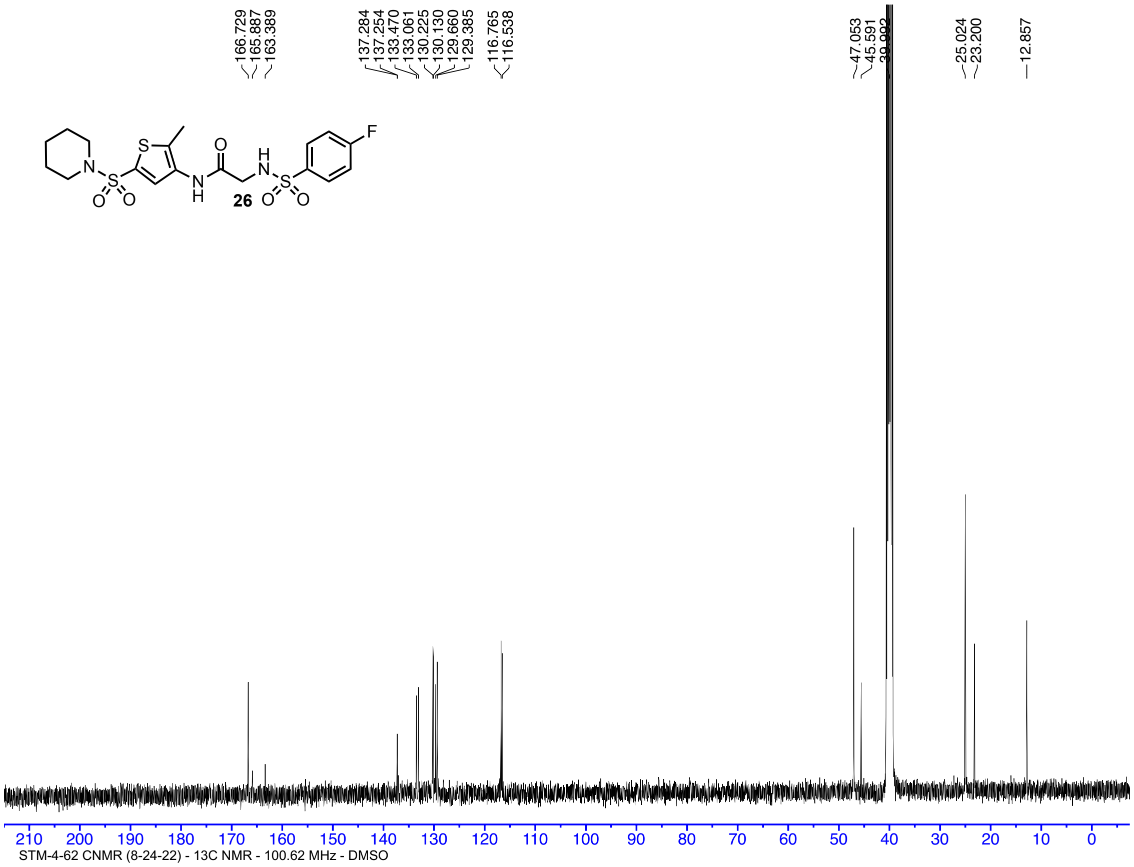

STM-4-62 CNMR (8-24-22) - 13C NMR - 100.62 MHz - DMSO

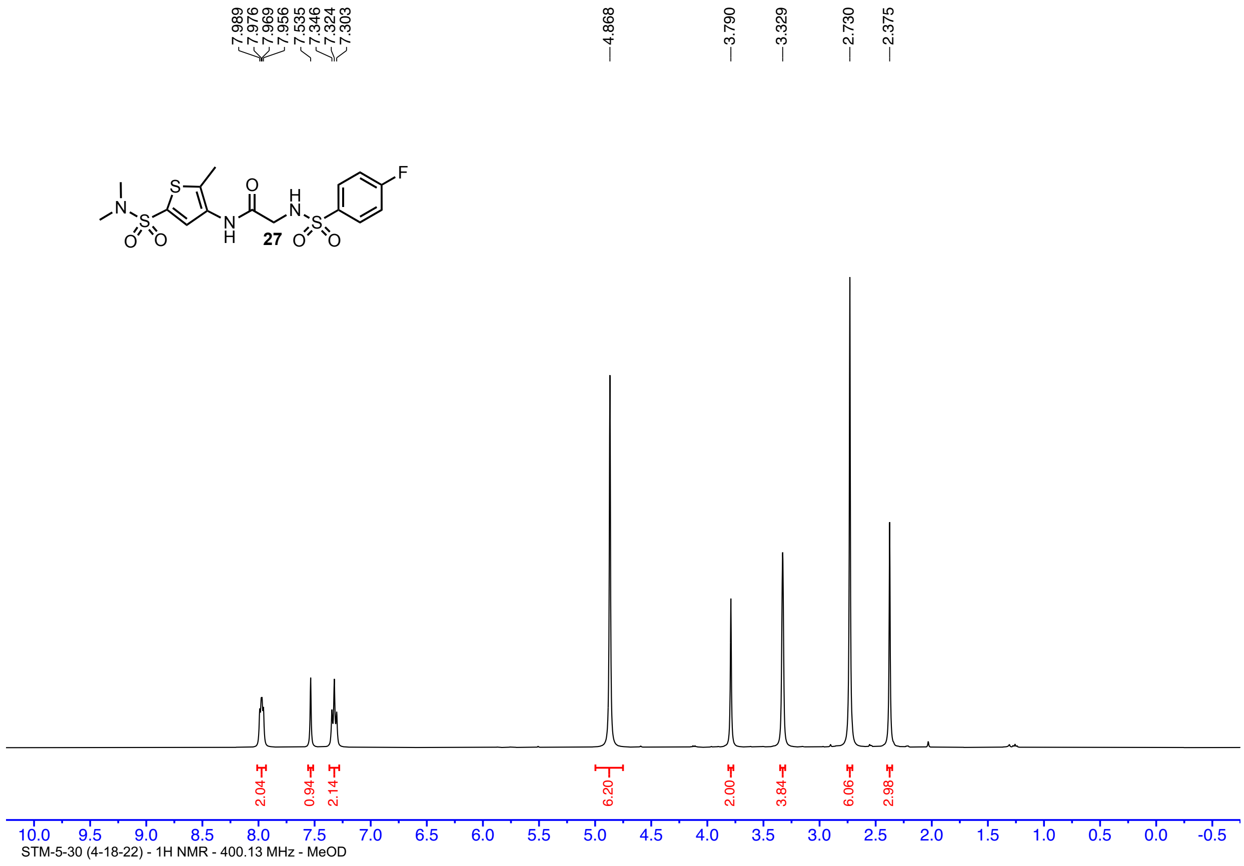
7.989
7.976
7.969
7.956
7.535
7.346
7.324
7.303
4.868
3.790
3.329
2.730
2.375
27
2.04
0.94
2.14
6.20
2.00
3.84
6.06
2.98
STM-5-30 (4-18-22) - 1H NMR - 400.13 MHz - MeOD

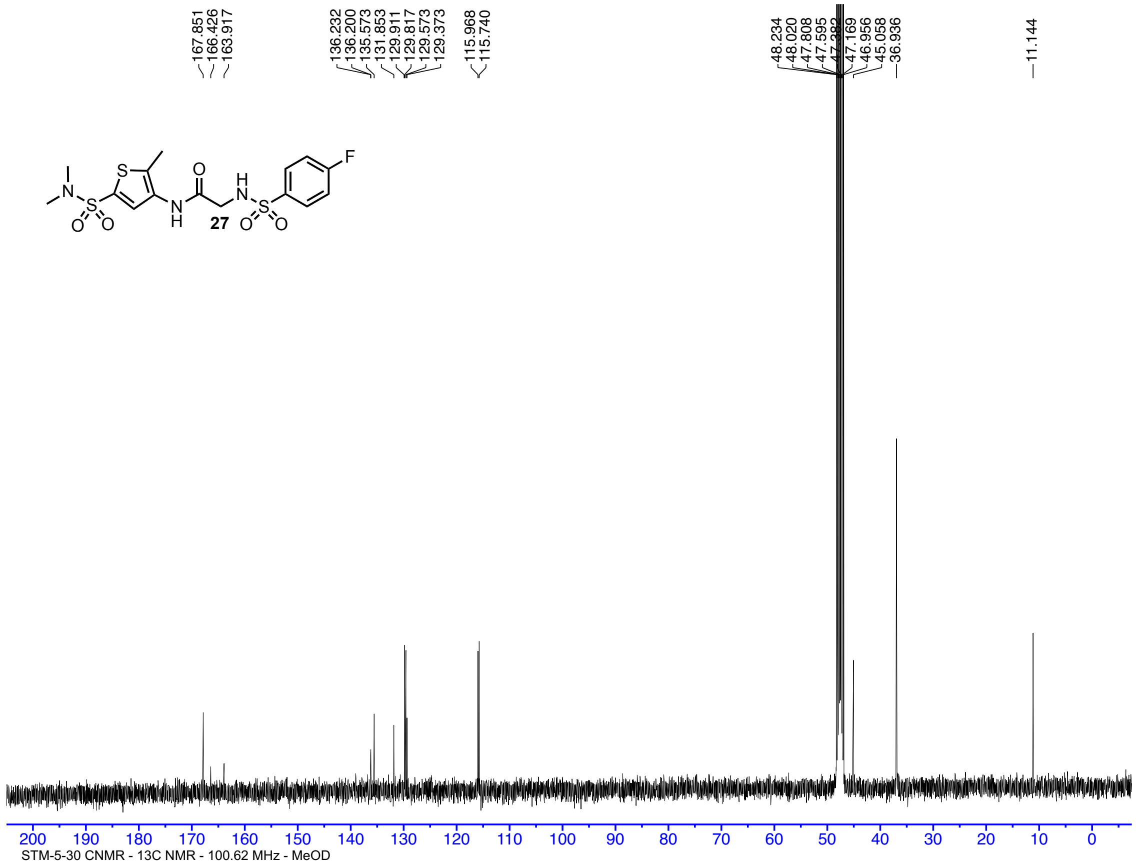
167.851
166.426
163.917
136.232
136.200
135.573
131.853
129.911
129.817
129.573
129.373
115.968
115.740
48.234
48.020
47.808
47.595
47.169
46.956
45.058
36.936
11.144
27
STM-5-30 CNMR - 13C NMR - 100.62 MHz - MeOD

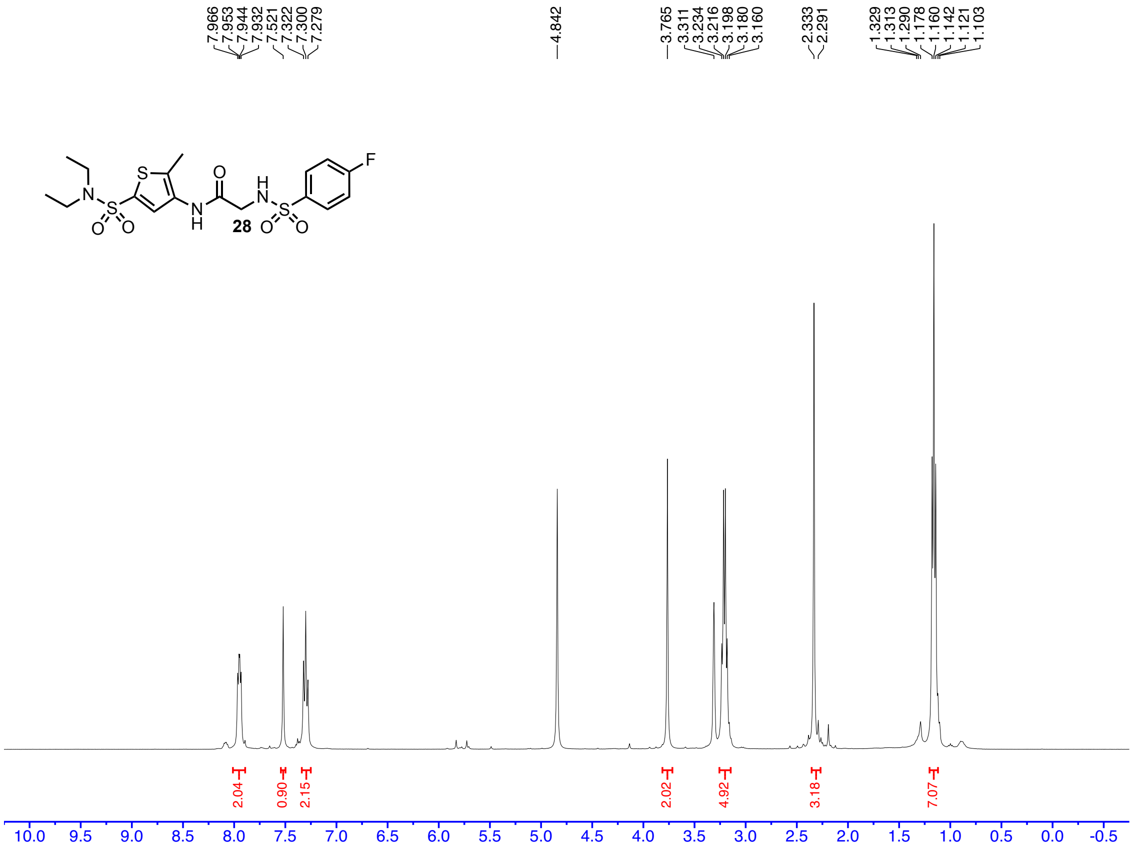

STM-4-150 (3-18-22) - 1H NMR - 400.13 MHz - MeOD

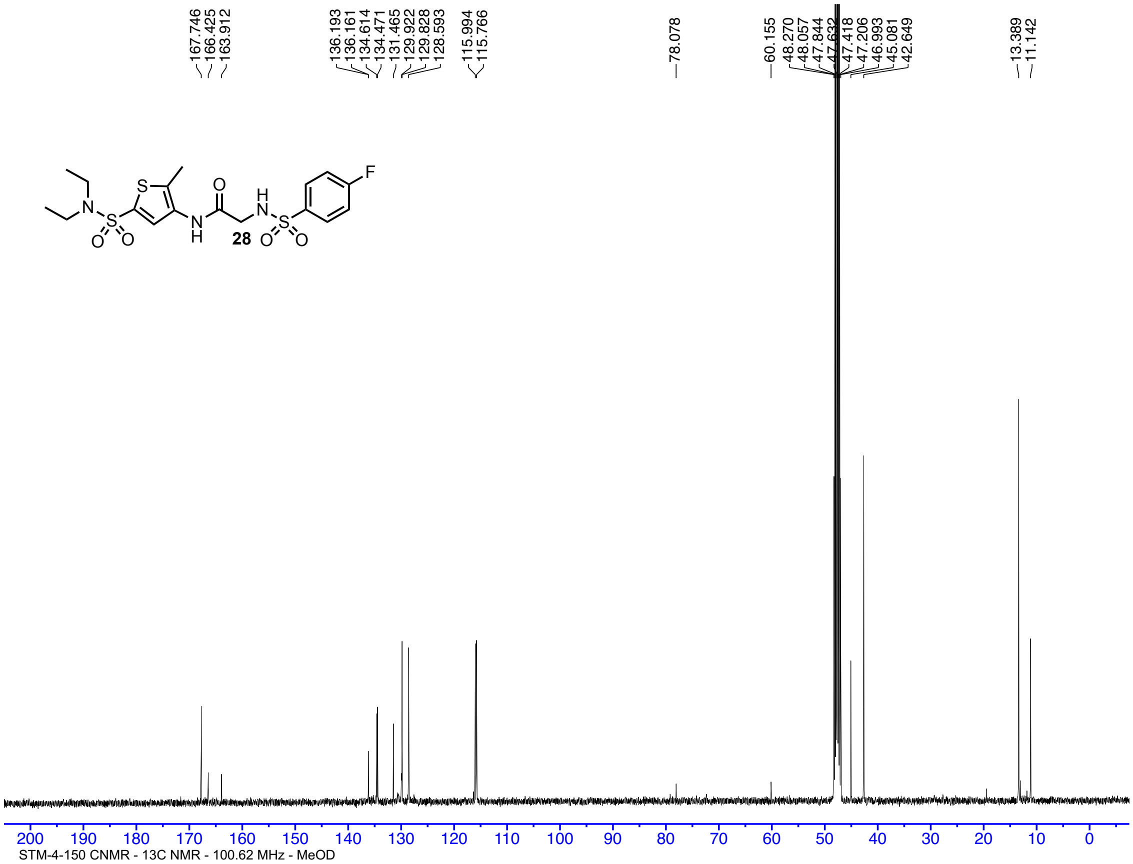

STM-4-150 CNMR - 13C NMR - 100.62 MHz - MeOD

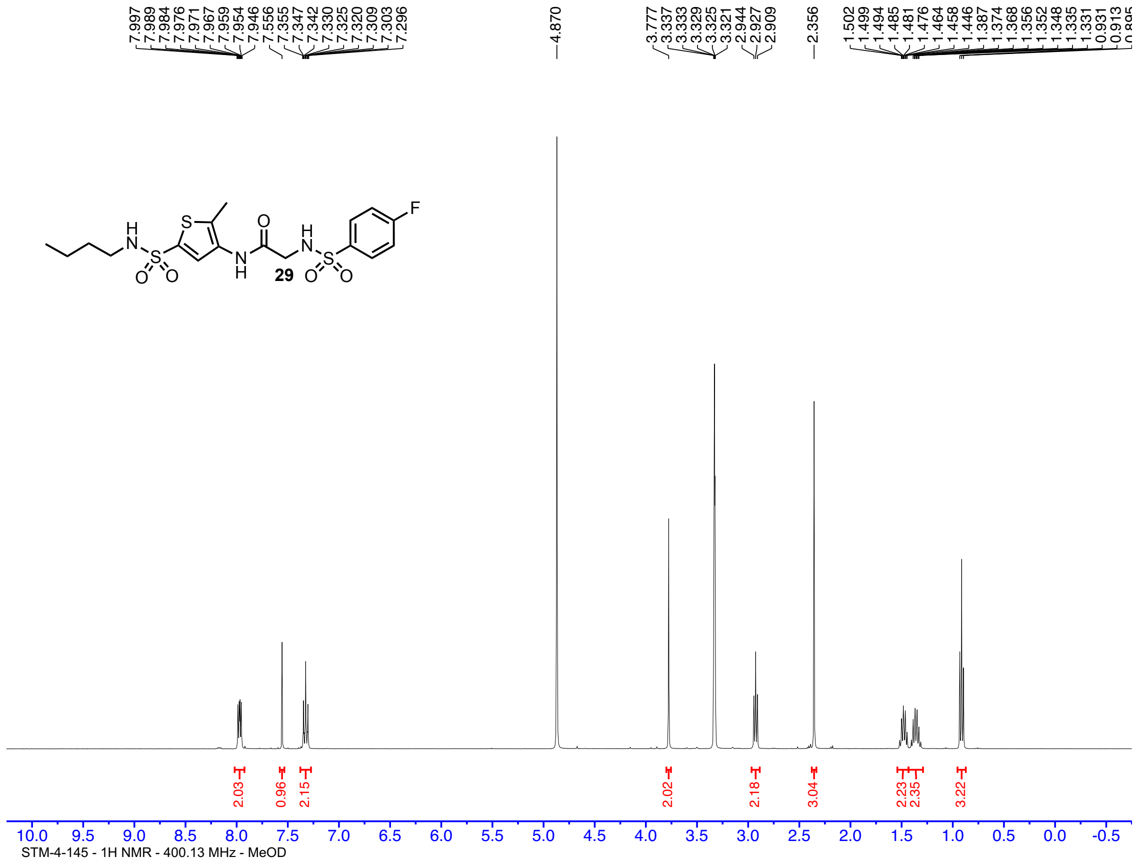
29
STM-4-145 - 1H NMR - 400.13 MHz - MeOD

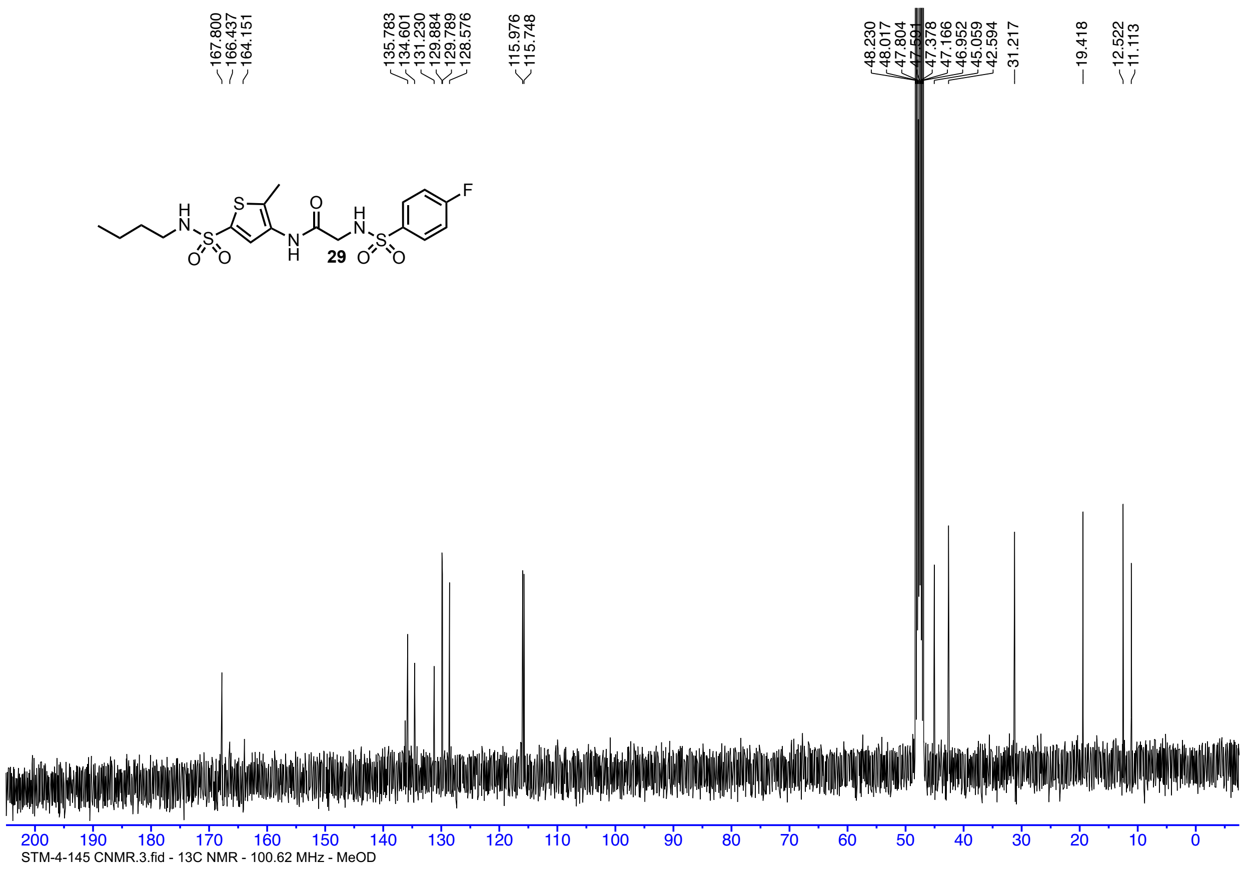
29
STM-4-145 CNMR.3.fid - 13C NMR - 100.62 MHz - MeOD

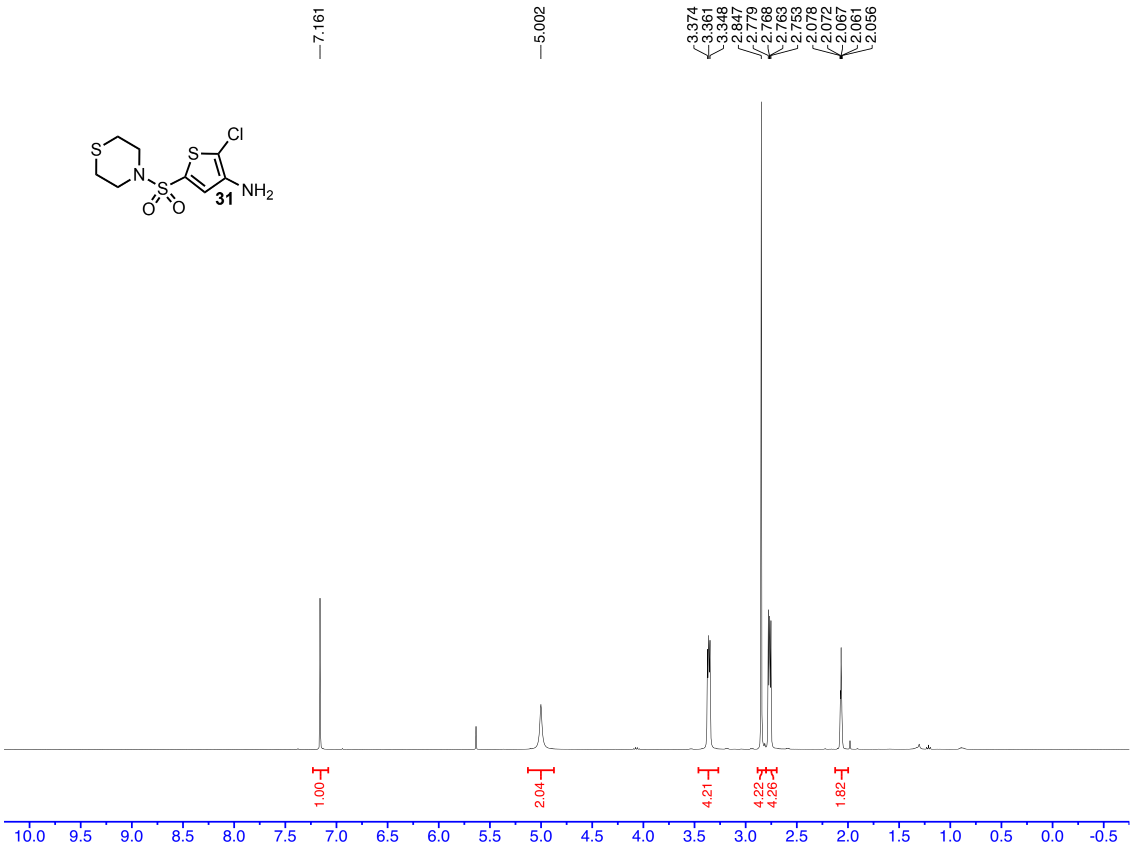

STM-5-115 (7-19-22) - 1H NMR - 400.13 MHz - Acetone

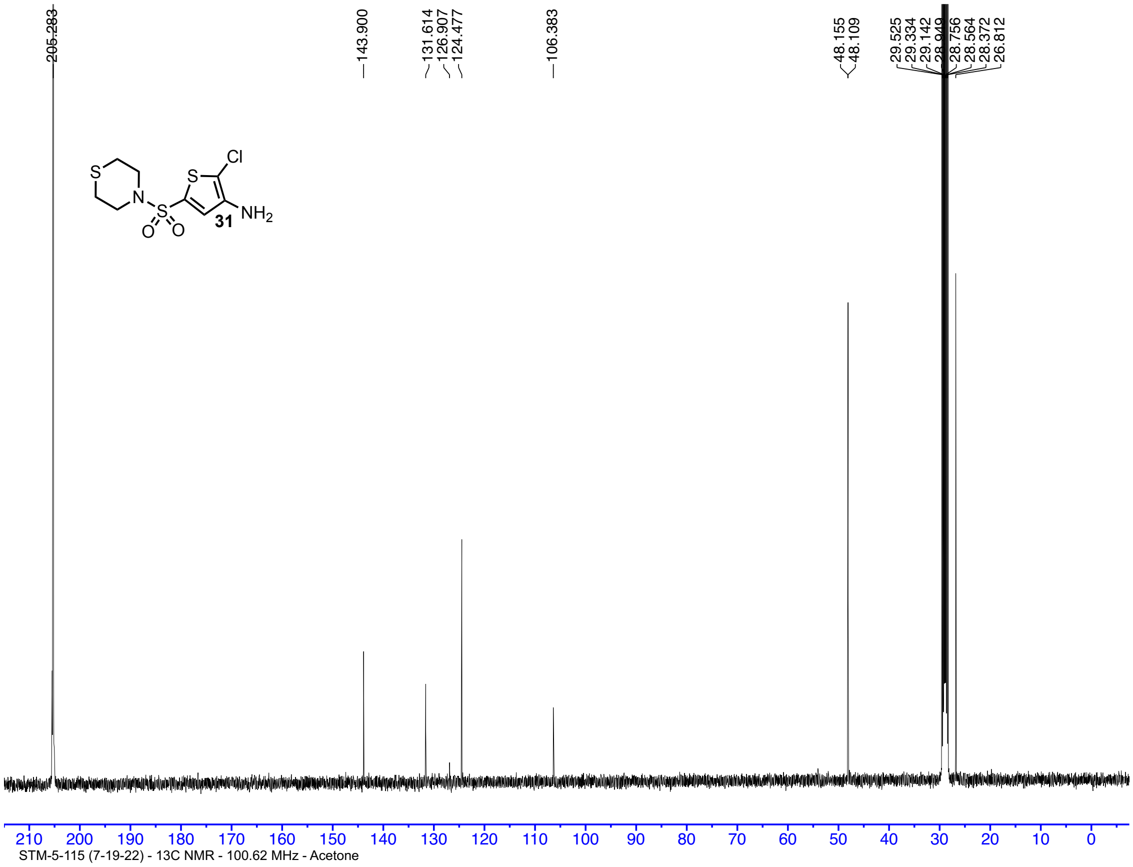

STM-5-115 (7-19-22) - 13C NMR - 100.62 MHz - Acetone

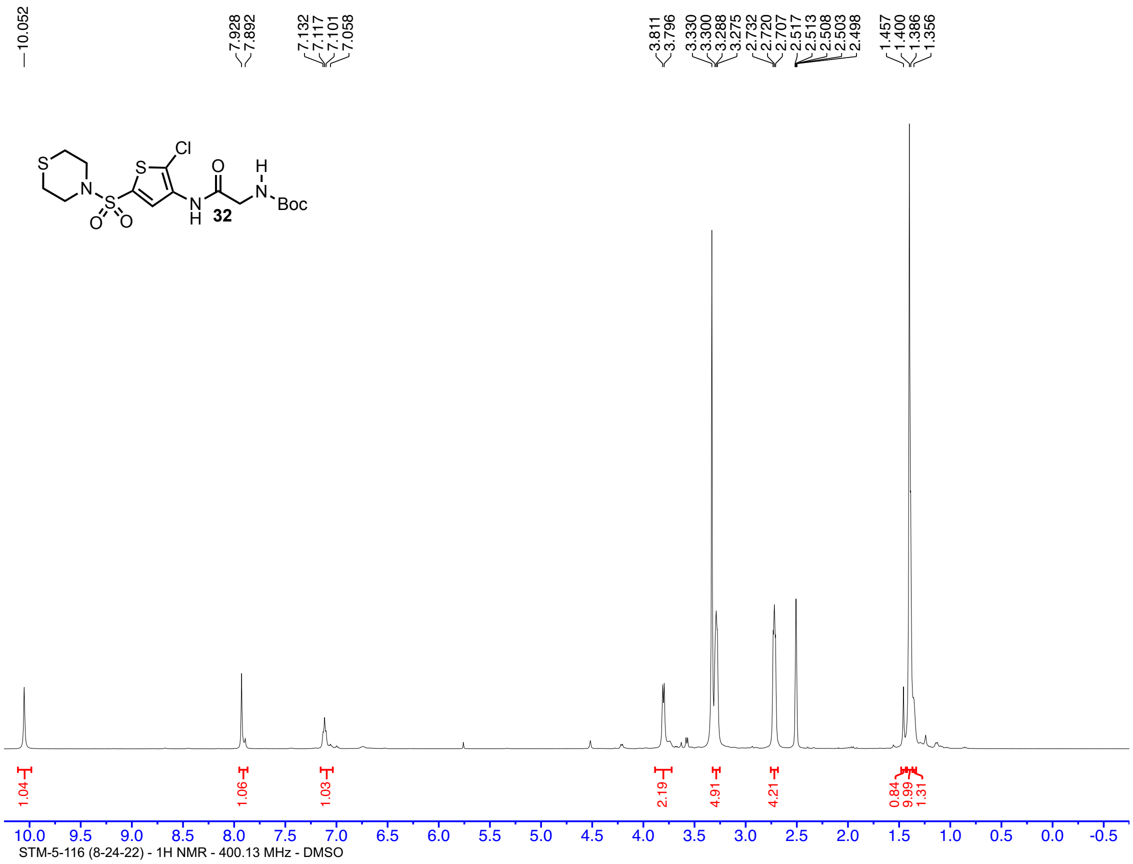
10.052
7.928
7.892
7.132
7.117
7.101
7.058
3.811
3.796
3.330
3.300
3.288
3.275
2.732
2.720
2.707
2.517
2.513
2.508
2.503
2.498
1.457
1.400
1.386
1.356
Cl
S
N
O
H
Boc
32
1.04
1.06
1.03
2.19
4.91
4.21
0.84
9.99
1.31
STM-5-116 (8-24-22) - 1H NMR - 400.13 MHz - DMSO

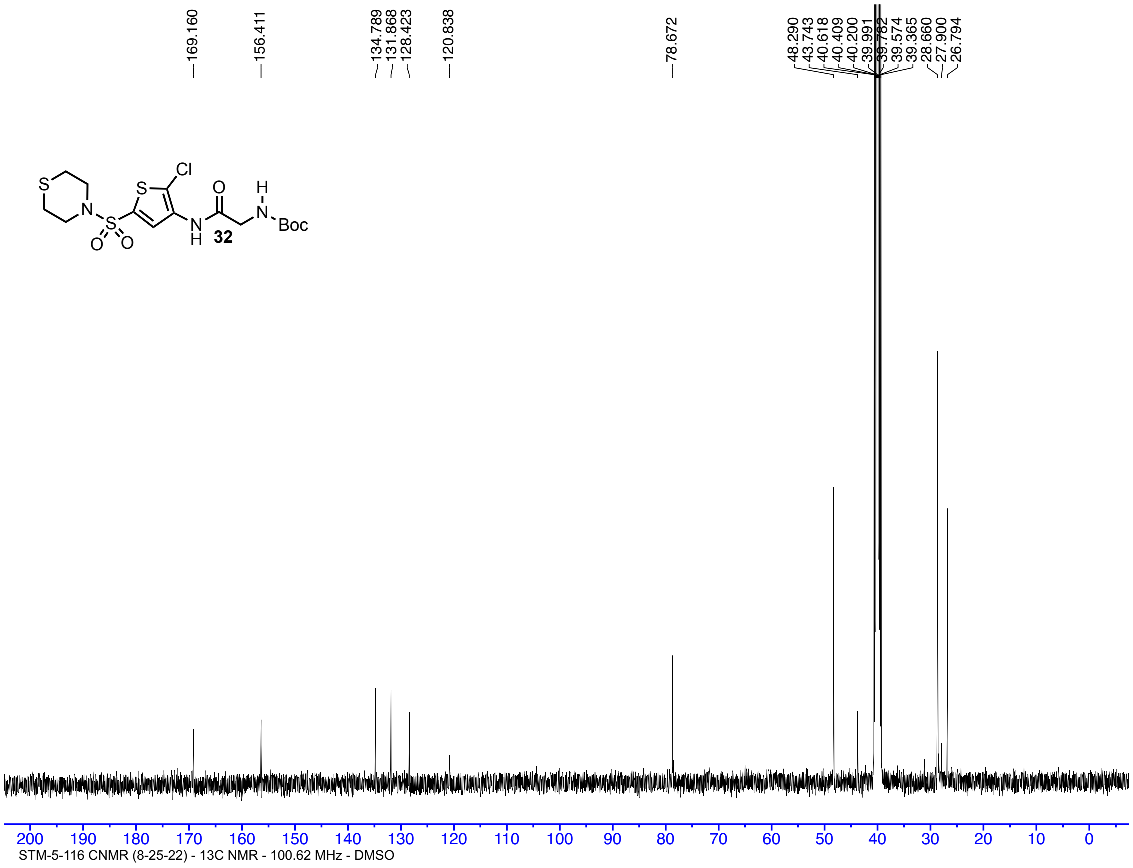
169.160
156.411
134.789
131.868
128.423
120.838
78.672
48.290
43.743
40.618
40.409
40.200
39.991
39.782
39.574
39.365
28.660
27.900
26.794
Cl
S
N
O
H
Boc
32
STM-5-116 CNMR (8-25-22) - 13C NMR - 100.62 MHz - DMSO

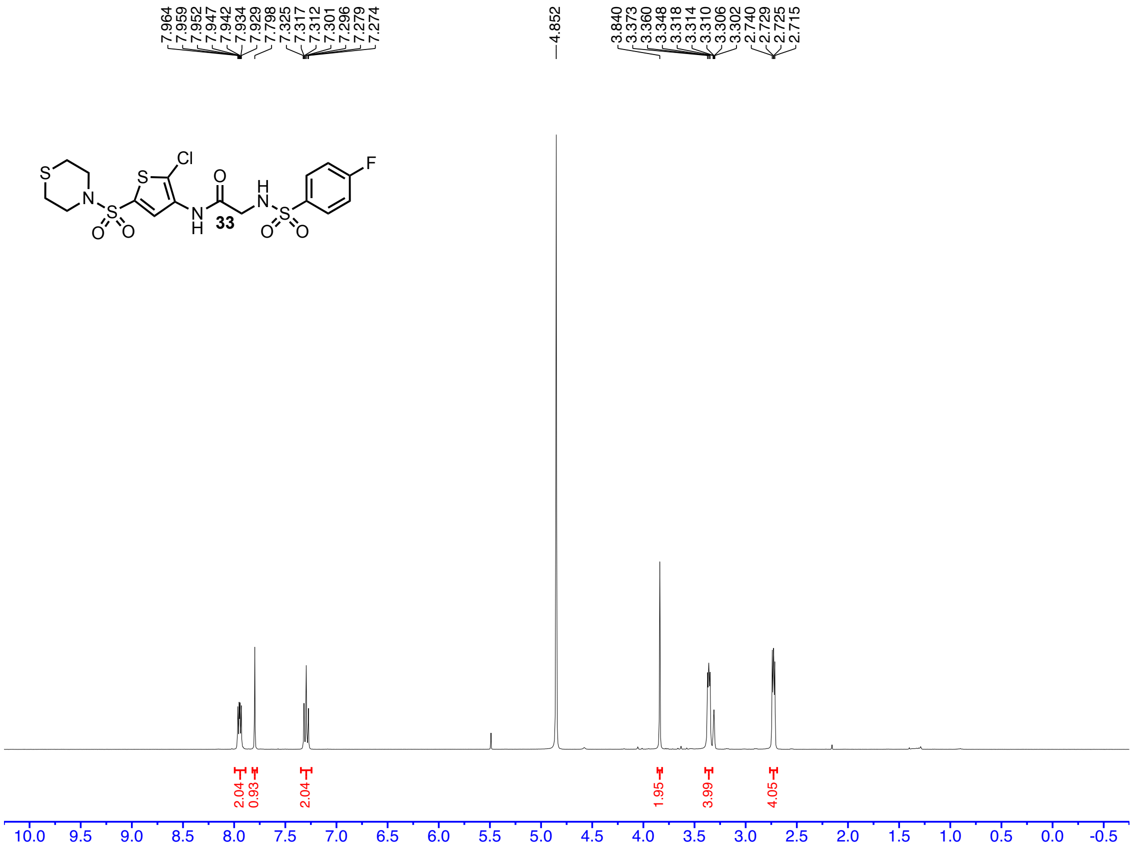

STM-5-119 - 1H NMR - 400.13 MHz - MeOD

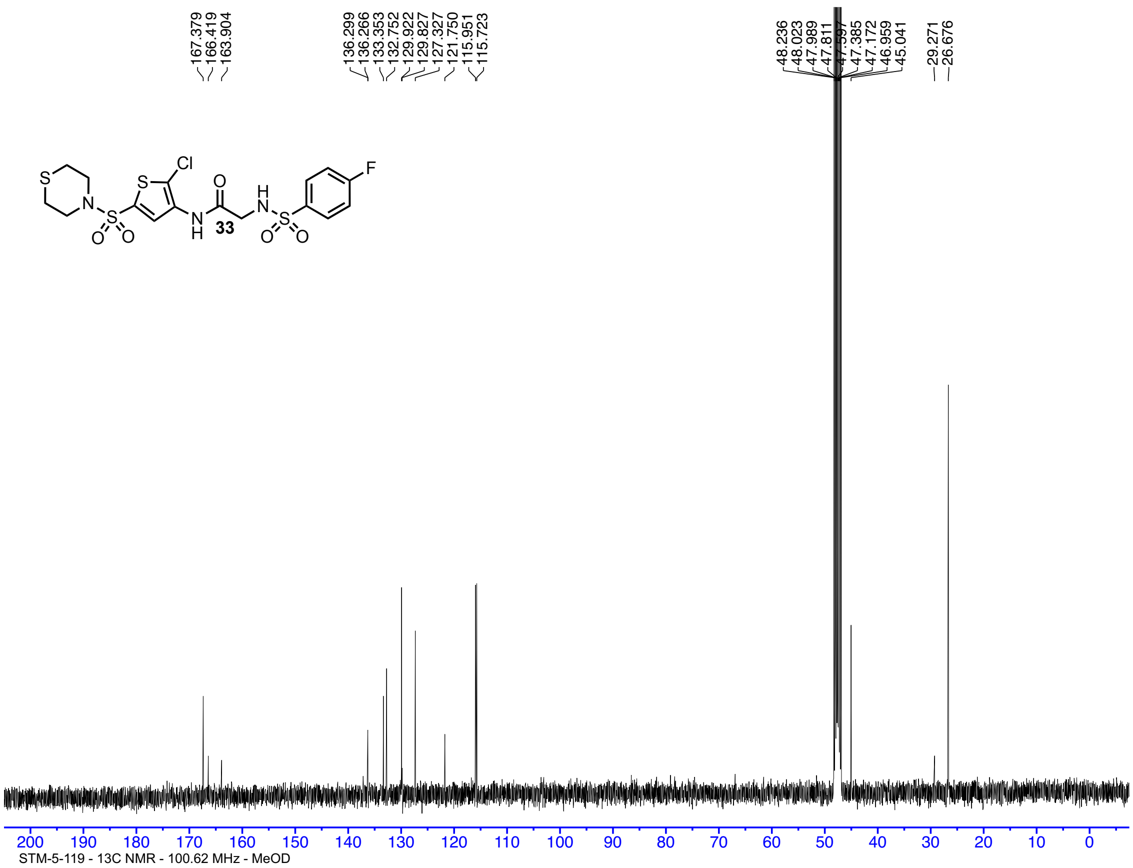

STM-5-119 - 13C NMR - 100.62 MHz - MeOD

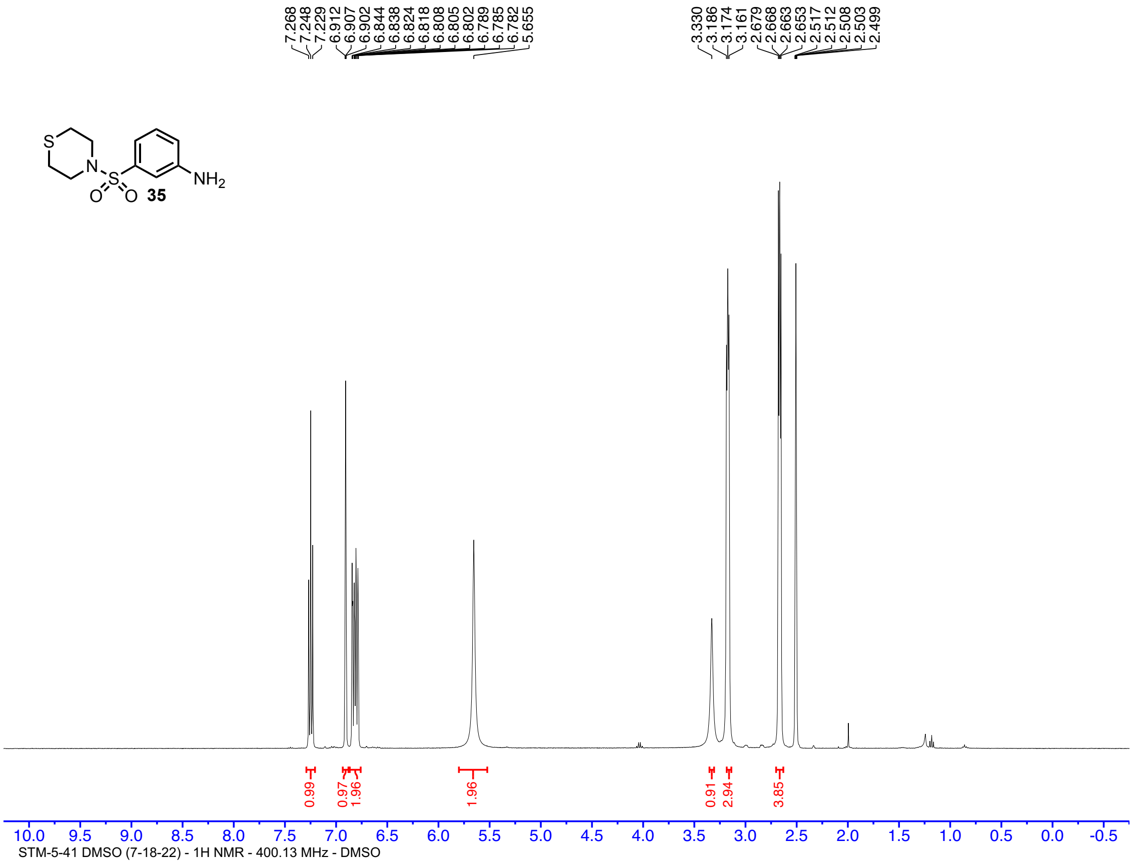

7.268
7.248
7.229
6.912
6.907
6.902
6.844
6.838
6.824
6.818
6.808
6.805
6.802
6.789
6.785
6.782
5.655
3.330
3.186
3.174
3.161
2.679
2.668
2.663
2.653
2.517
2.512
2.508
2.503
2.499
S
N
S
O
O
NH2
35
0.99
0.97
1.96
1.96
0.91
2.94
3.85
10.0 9.5 9.0 8.5 8.0 7.5 7.0 6.5 6.0 5.5 5.0 4.5 4.0 3.5 3.0 2.5 2.0 1.5 1.0 0.5 0.0 -0.5
STM-5-41 DMSO (7-18-22) - 1H NMR - 400.13 MHz - DMSO

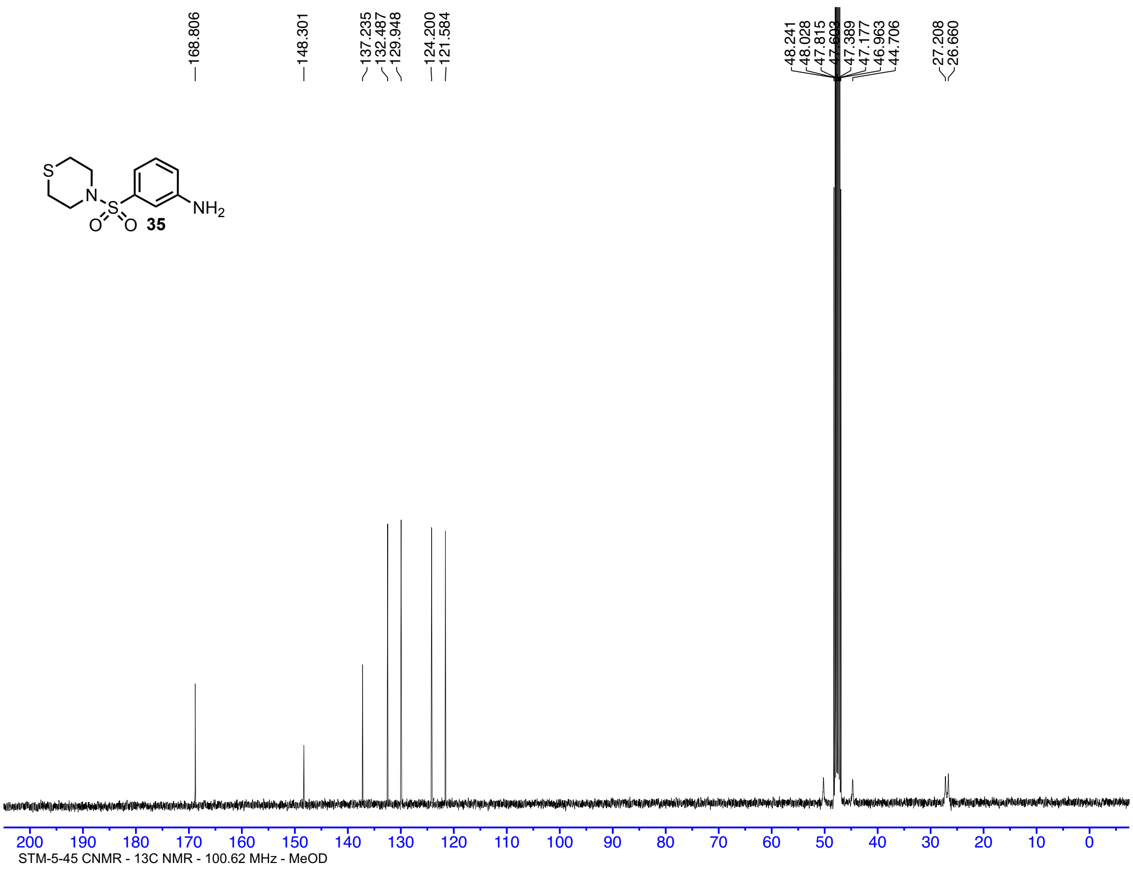

168.806
148.301
137.235
132.487
129.948
124.200
121.584
48.241
48.028
47.815
47.389
47.177
46.963
44.706
27.208
26.660
S
N
S
O
O
NH2
35
200 190 180 170 160 150 140 130 120 110 100 90 80 70 60 50 40 30 20 10 0
STM-5-45 CNMR - 13C NMR - 100.62 MHz - MeOD

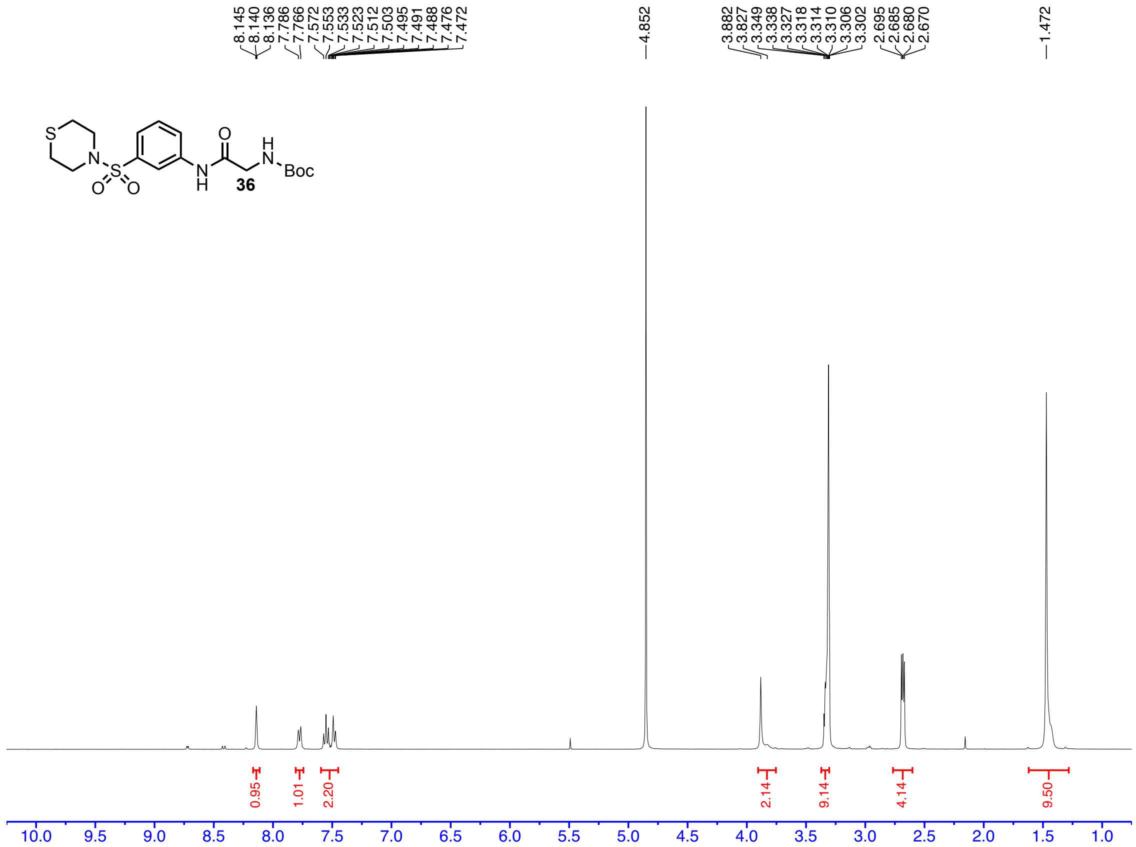

STM-5-47 (5-3-22) - 1H NMR - 400.13 MHz - MeOD

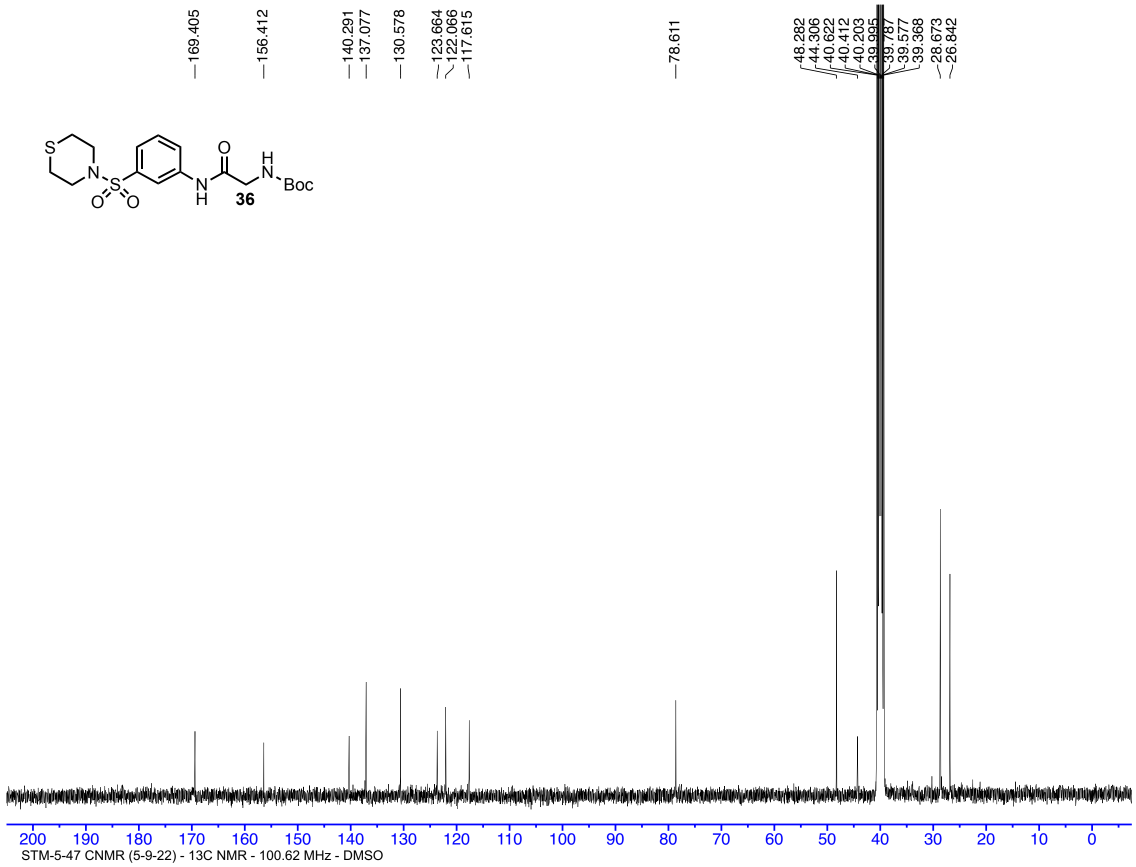

STM-5-47 CNMR (5-9-22) - 13C NMR - 100.62 MHz - DMSO

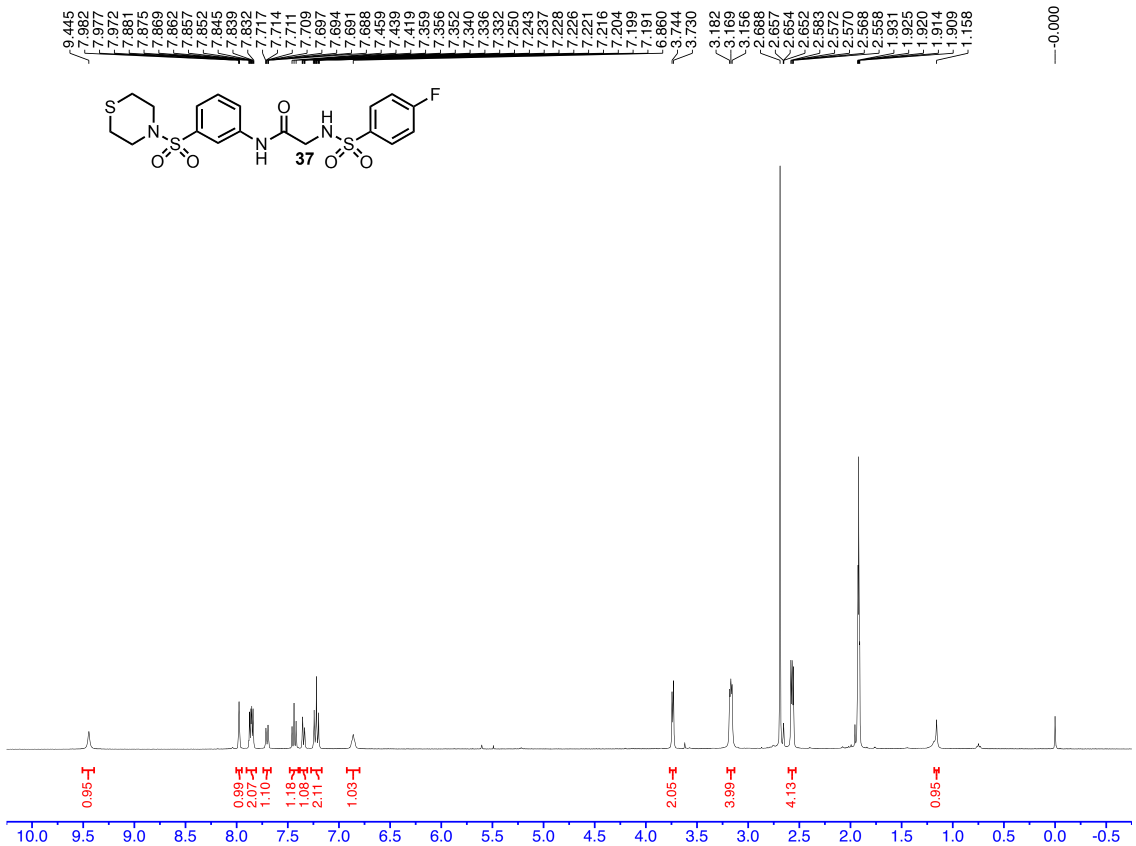

STM-5-51 (5-22-22) - 1H NMR - 400.13 MHz - Acetone

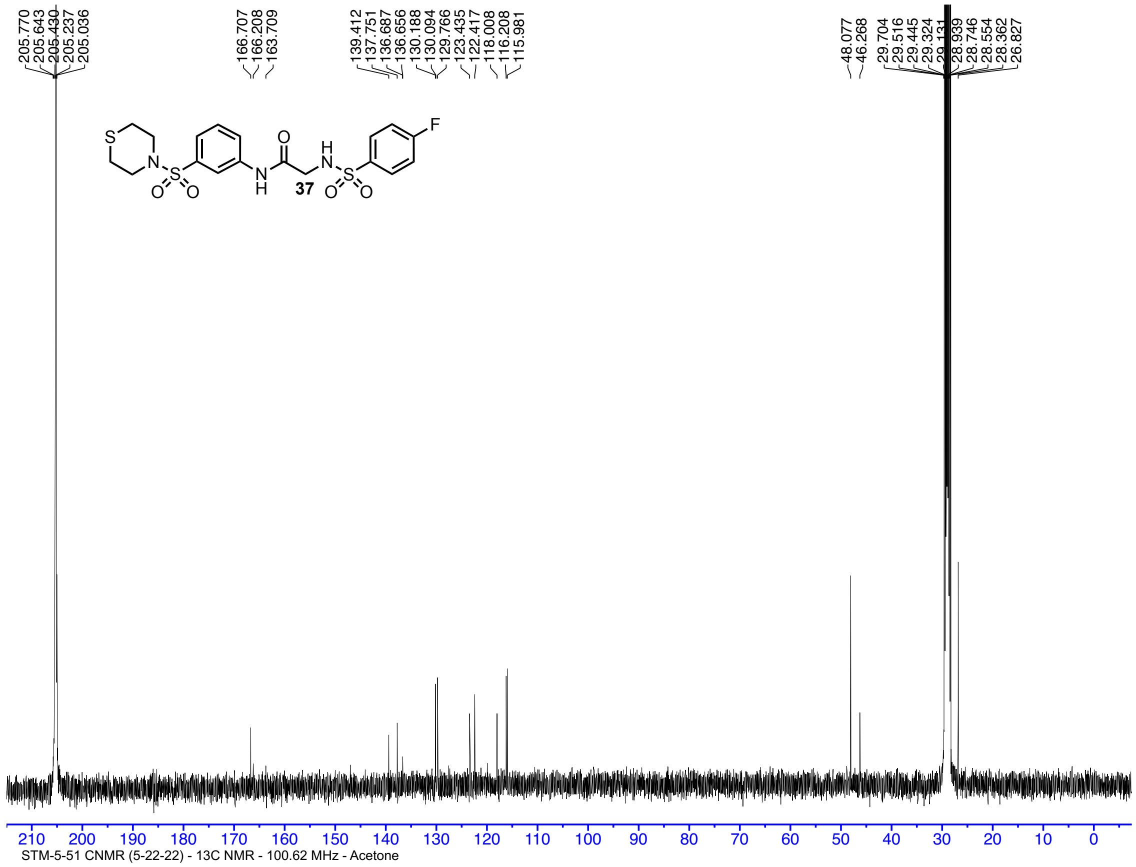

STM-5-51 CNMR (5-22-22) - 13C NMR - 100.62 MHz - Acetone

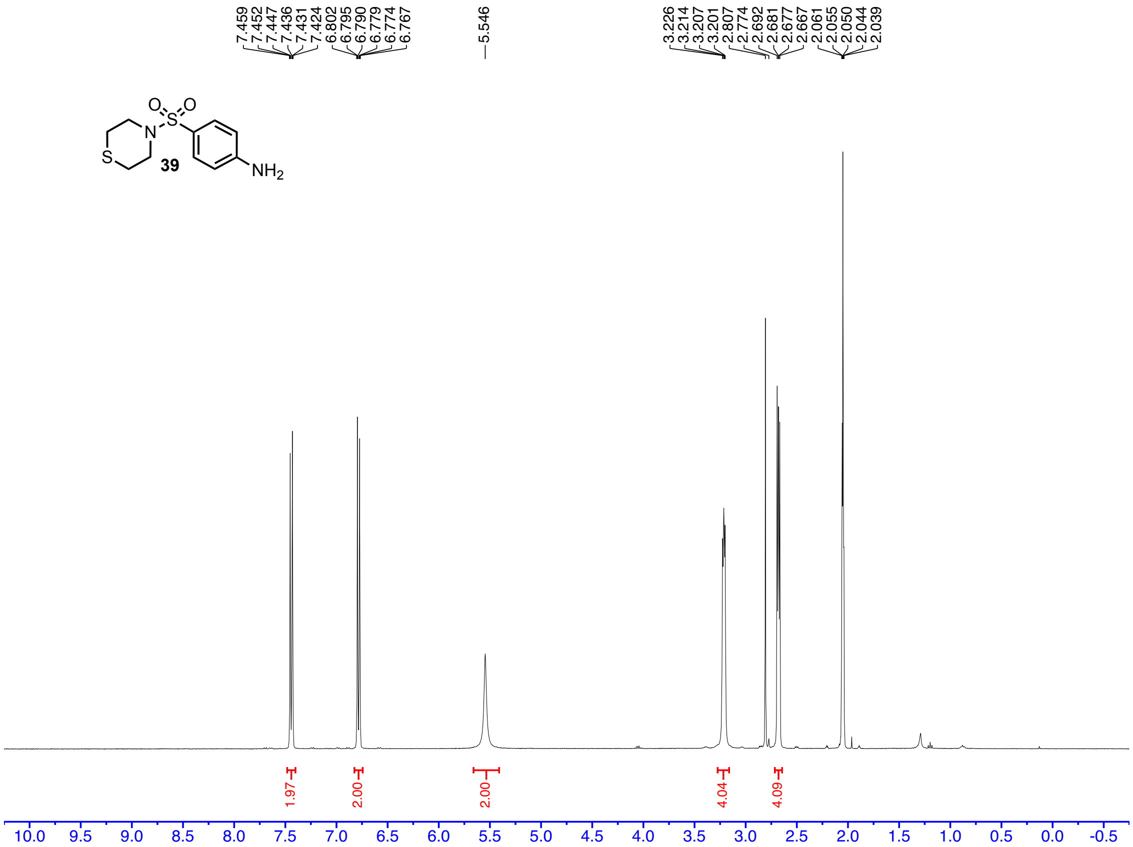

STM-5-39 (6-21-22) - 1H NMR - 400.13 MHz - Acetone

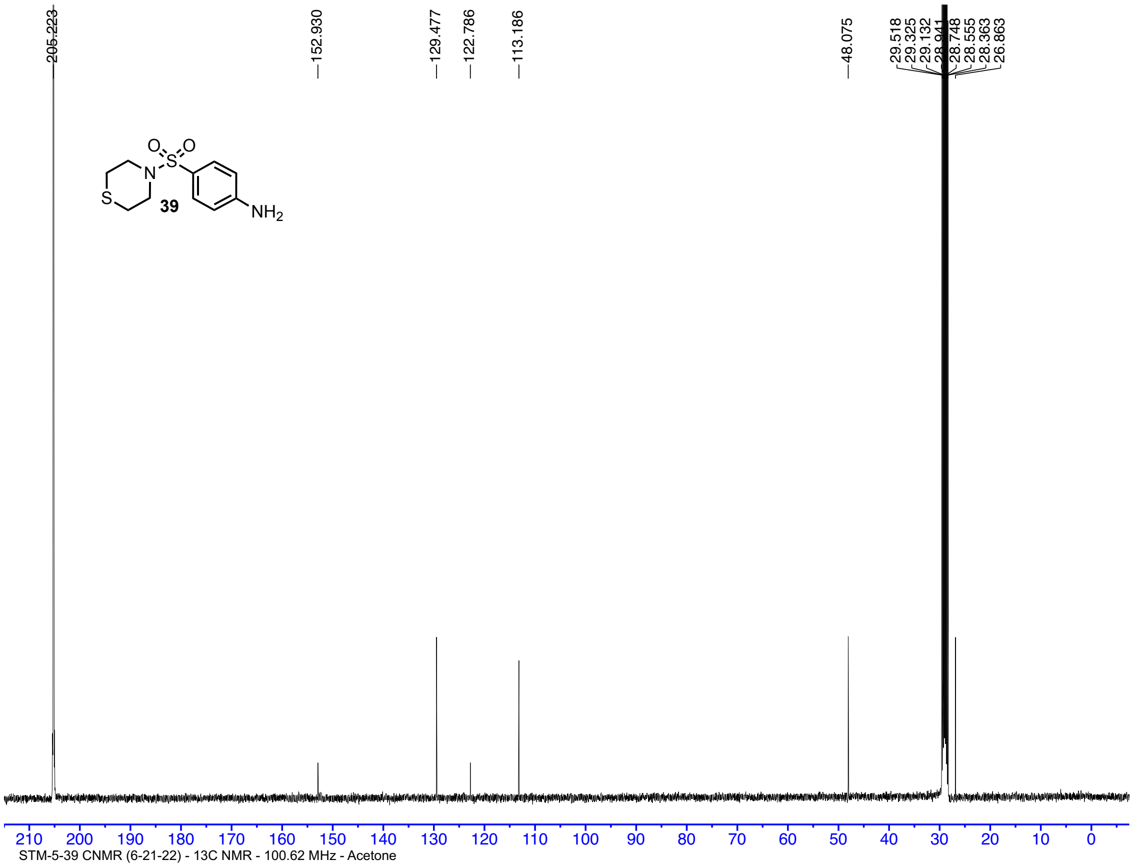

STM-5-39 CNMR (6-21-22) - 13C NMR - 100.62 MHz - Acetone

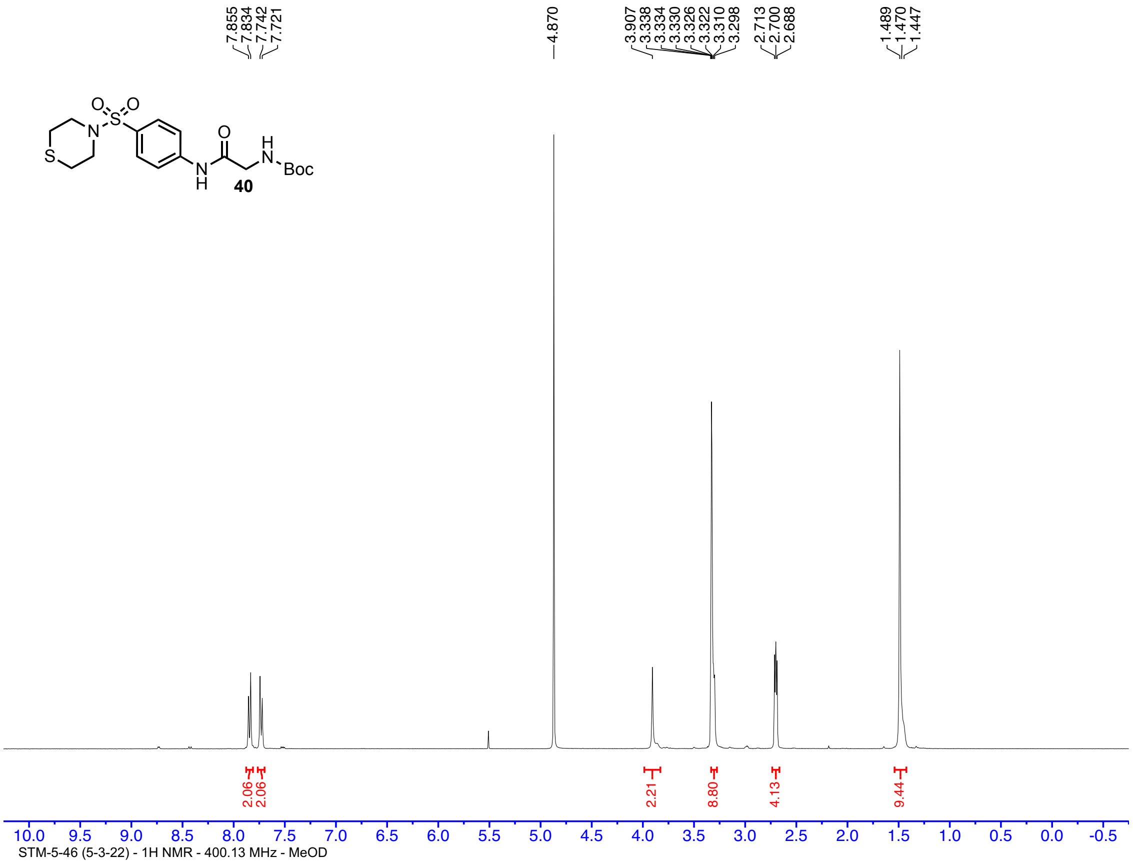
7.855
7.834
7.742
7.721
4.870
3.907
3.338
3.334
3.330
3.326
3.322
3.310
3.298
2.713
2.700
2.688
1.489
1.470
1.447
40
2.06
2.06
2.21
8.80
4.13
9.44
10.0 9.5 9.0 8.5 8.0 7.5 7.0 6.5 6.0 5.5 5.0 4.5 4.0 3.5 3.0 2.5 2.0 1.5 1.0 0.5 0.0 -0.5
STM-5-46 (5-3-22) - 1H NMR - 400.13 MHz - MeOD

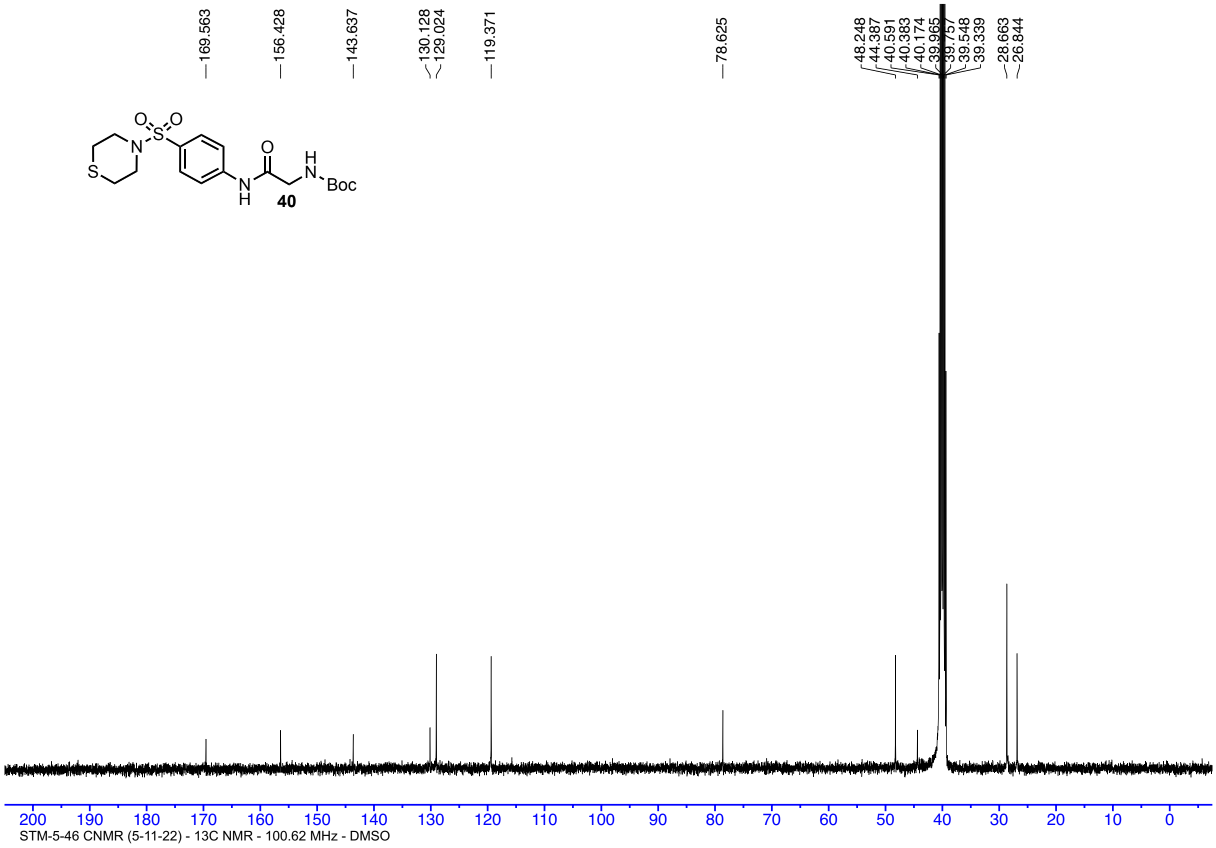
169.563
156.428
143.637
130.128
129.024
119.371
78.625
48.248
44.387
40.591
40.383
40.174
39.965
39.757
39.548
39.339
28.663
26.844
40
200 190 180 170 160 150 140 130 120 110 100 90 80 70 60 50 40 30 20 10 0
STM-5-46 CNMR (5-11-22) - 13C NMR - 100.62 MHz - DMSO

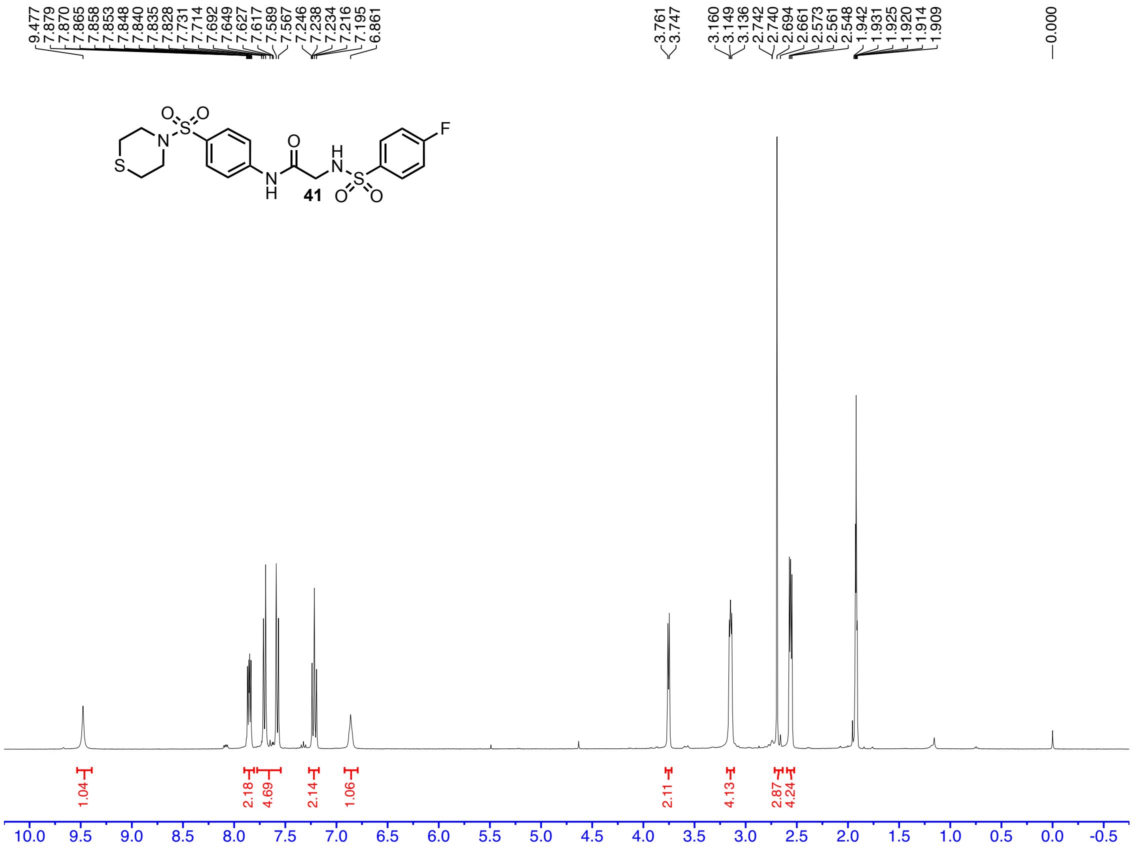

STM-5-52 (5-22-22) - 1H NMR - 400.13 MHz - Acetone

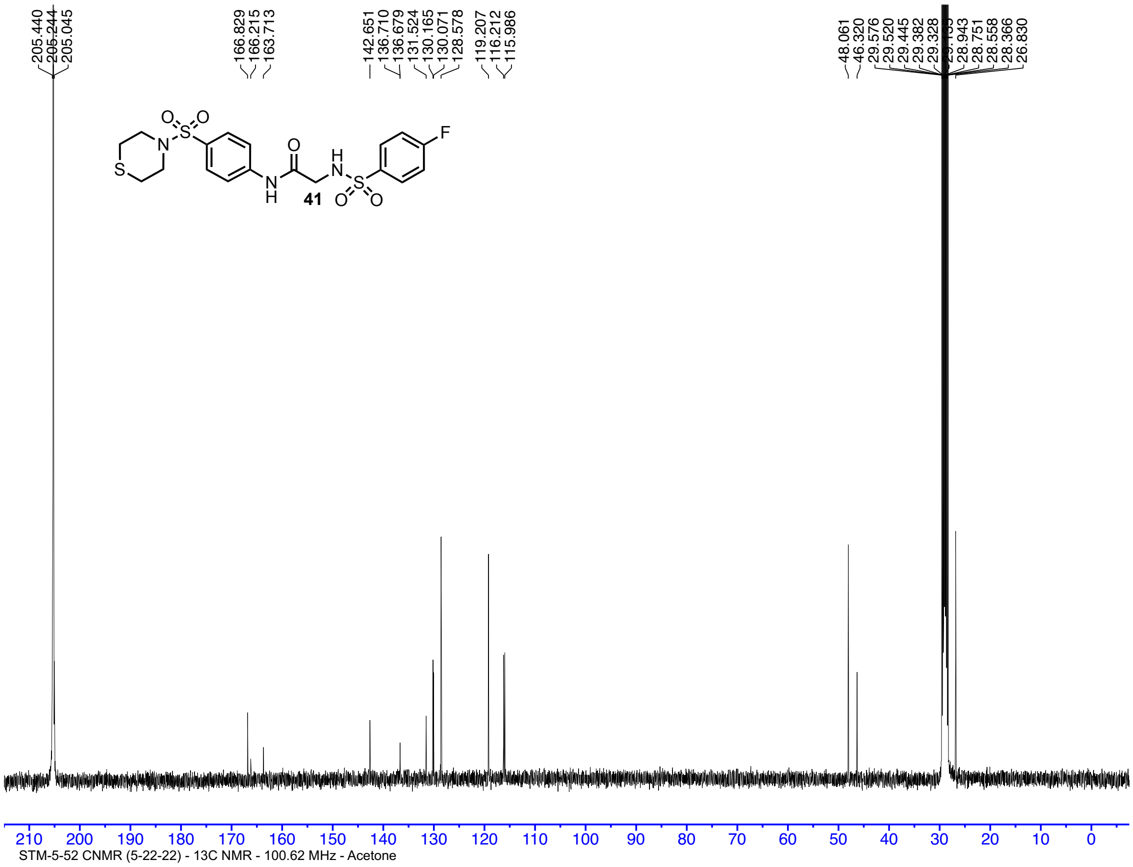

STM-5-52 CNMR (5-22-22) - 13C NMR - 100.62 MHz - Acetone

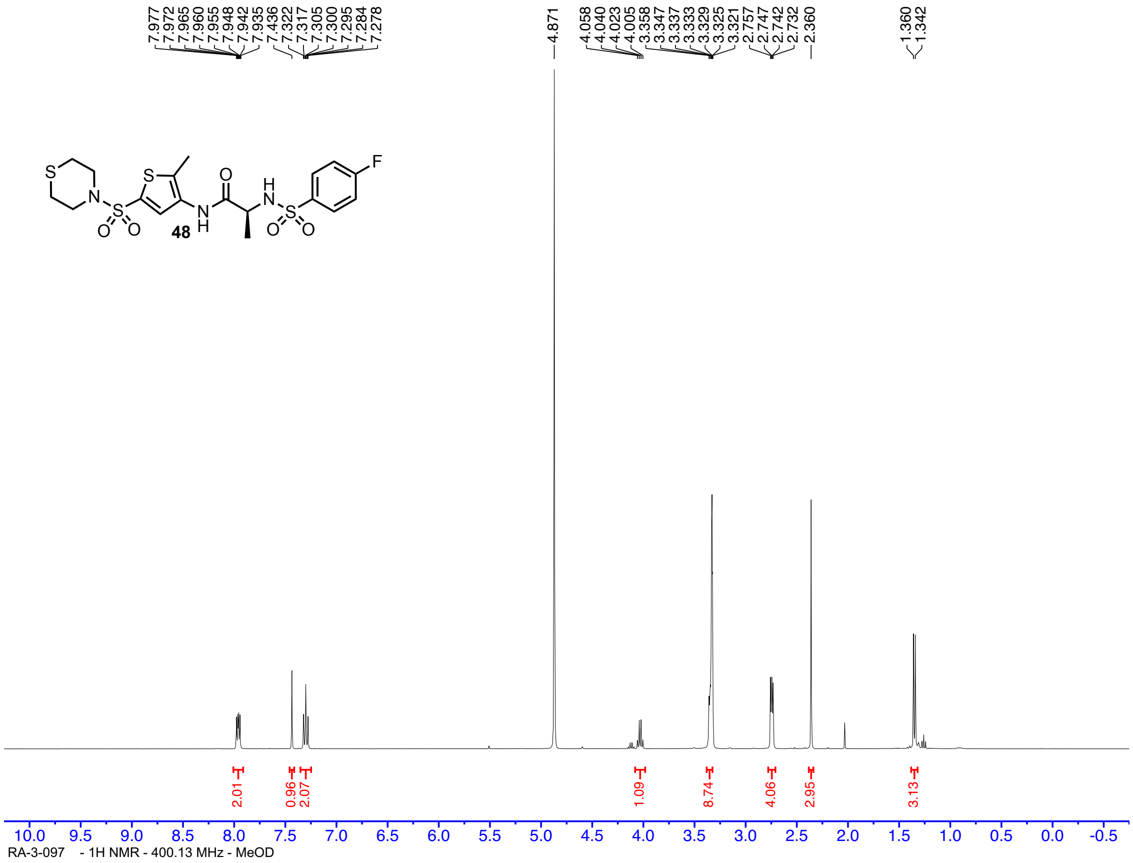
7.977
7.972
7.965
7.960
7.955
7.948
7.942
7.935
7.436
7.322
7.317
7.305
7.300
7.295
7.284
7.278
4.871
4.058
4.040
4.023
4.005
3.358
3.347
3.337
3.333
3.329
3.325
3.321
2.757
2.747
2.742
2.732
2.360
1.360
1.342
48
2.01
0.96
2.07
1.09
8.74
4.06
2.95
3.13
10.0 9.5 9.0 8.5 8.0 7.5 7.0 6.5 6.0 5.5 5.0 4.5 4.0 3.5 3.0 2.5 2.0 1.5 1.0 0.5 0.0 -0.5
RA-3-097 - 1H NMR - 400.13 MHz - MeOD

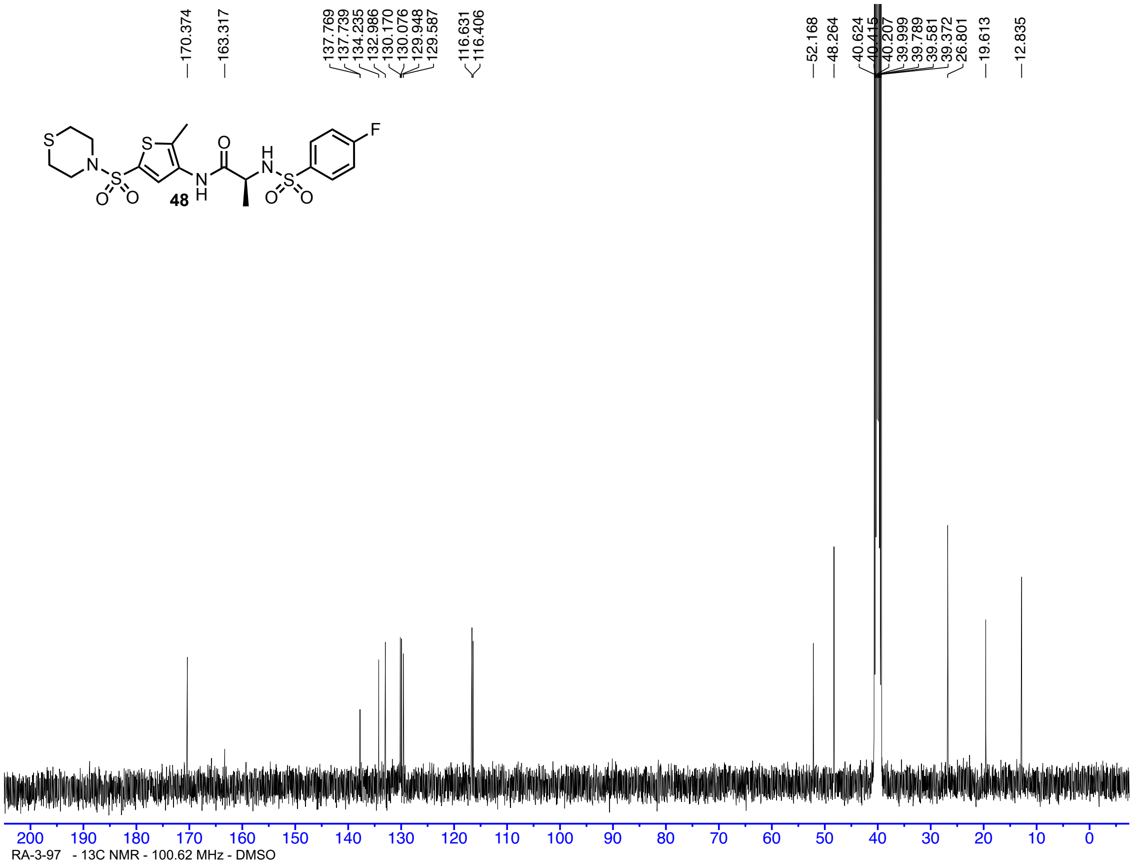
170.374
163.317
137.769
137.739
134.235
132.986
130.170
130.076
129.948
129.587
116.631
116.406
52.168
48.264
40.624
40.415
40.207
39.999
39.789
39.581
39.372
26.801
19.613
12.835
48
200 190 180 170 160 150 140 130 120 110 100 90 80 70 60 50 40 30 20 10 0
RA-3-97 - 13C NMR - 100.62 MHz - DMSO

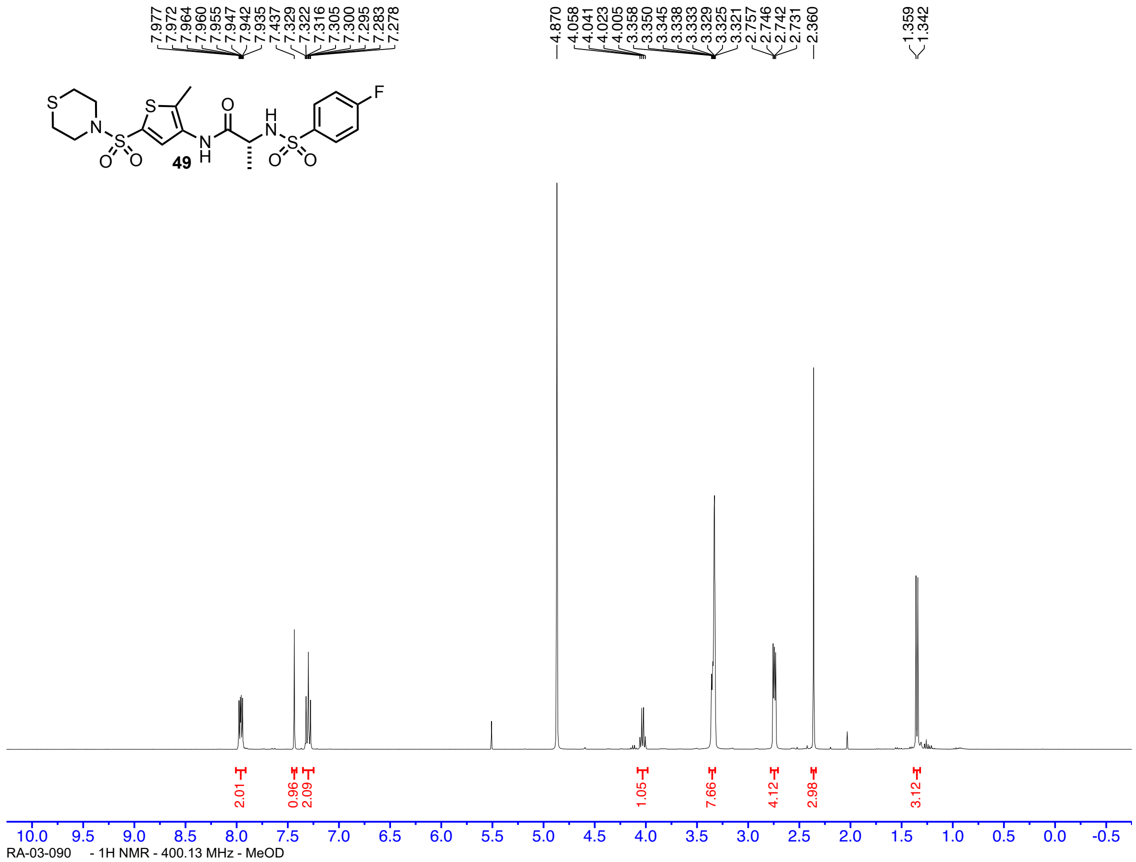

RA-03-090 - 1H NMR - 400.13 MHz - MeOD

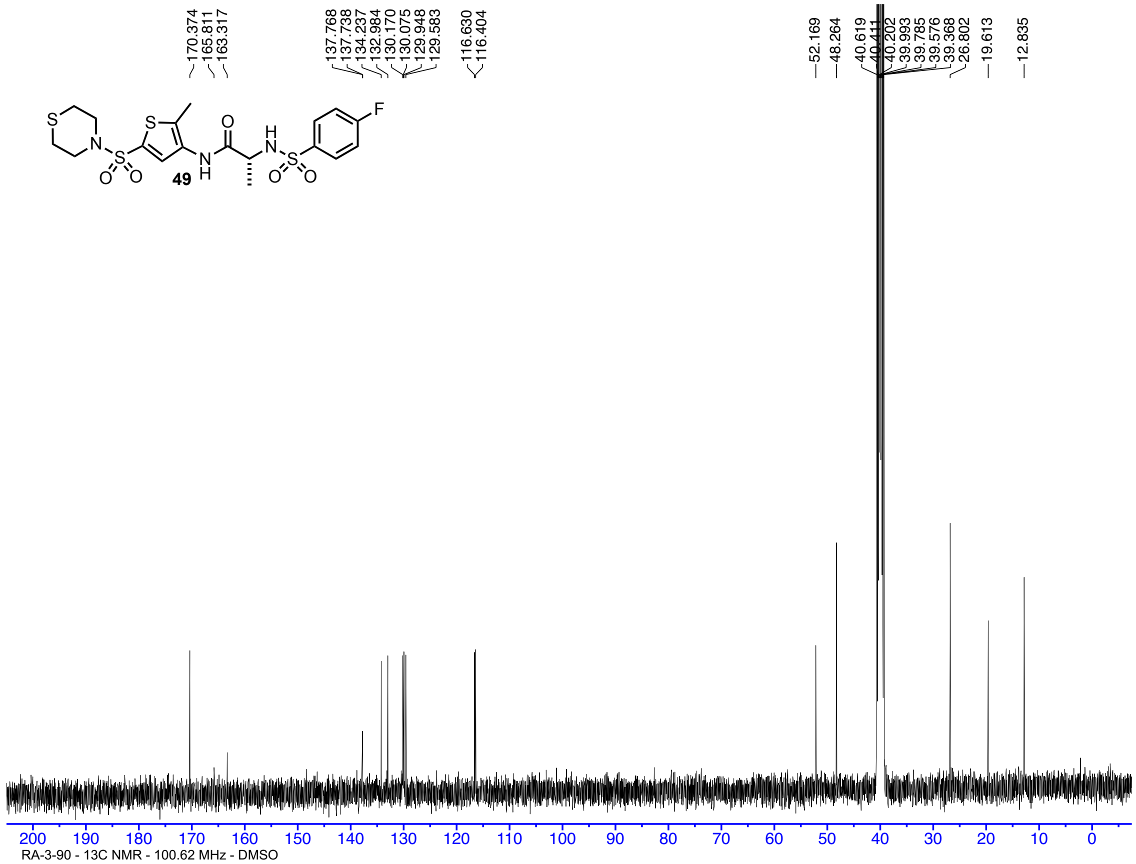

RA-3-90 - 13C NMR - 100.62 MHz - DMSO

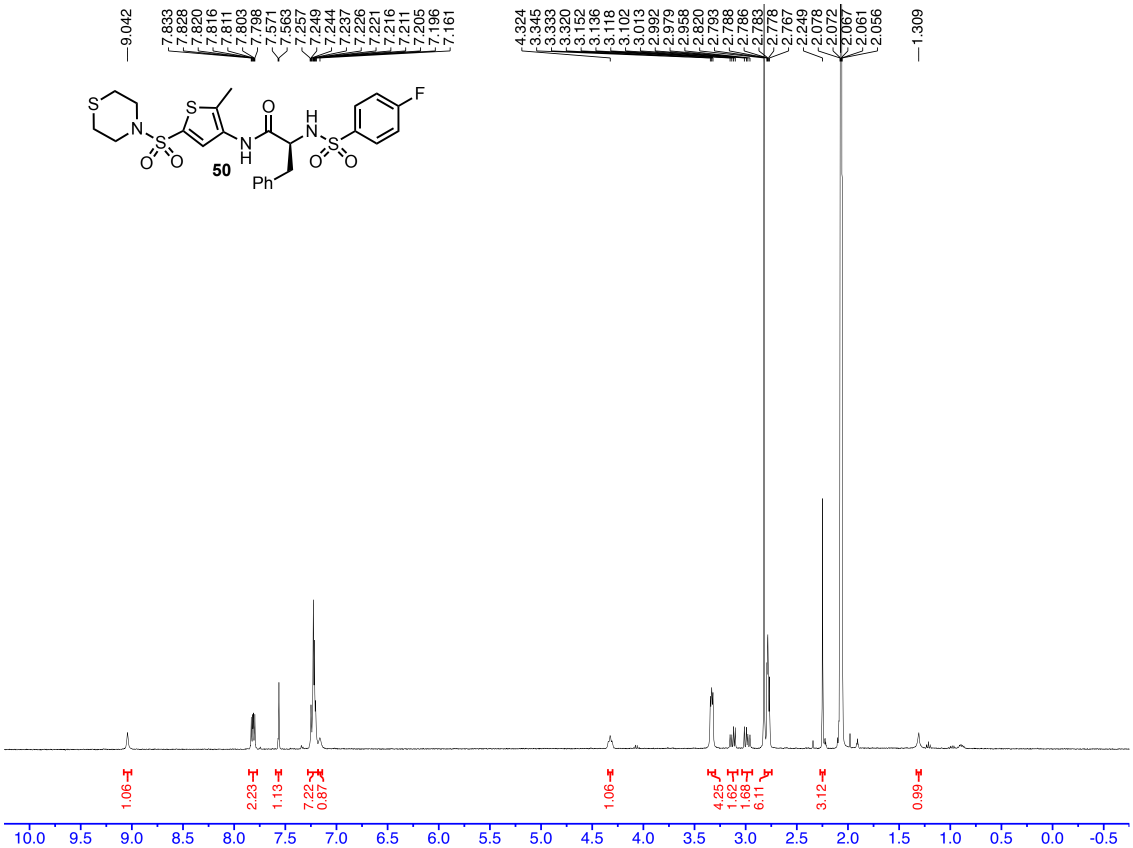

AMP-4-016 — pure - 1H NMR - 400.13 MHz - Acetone

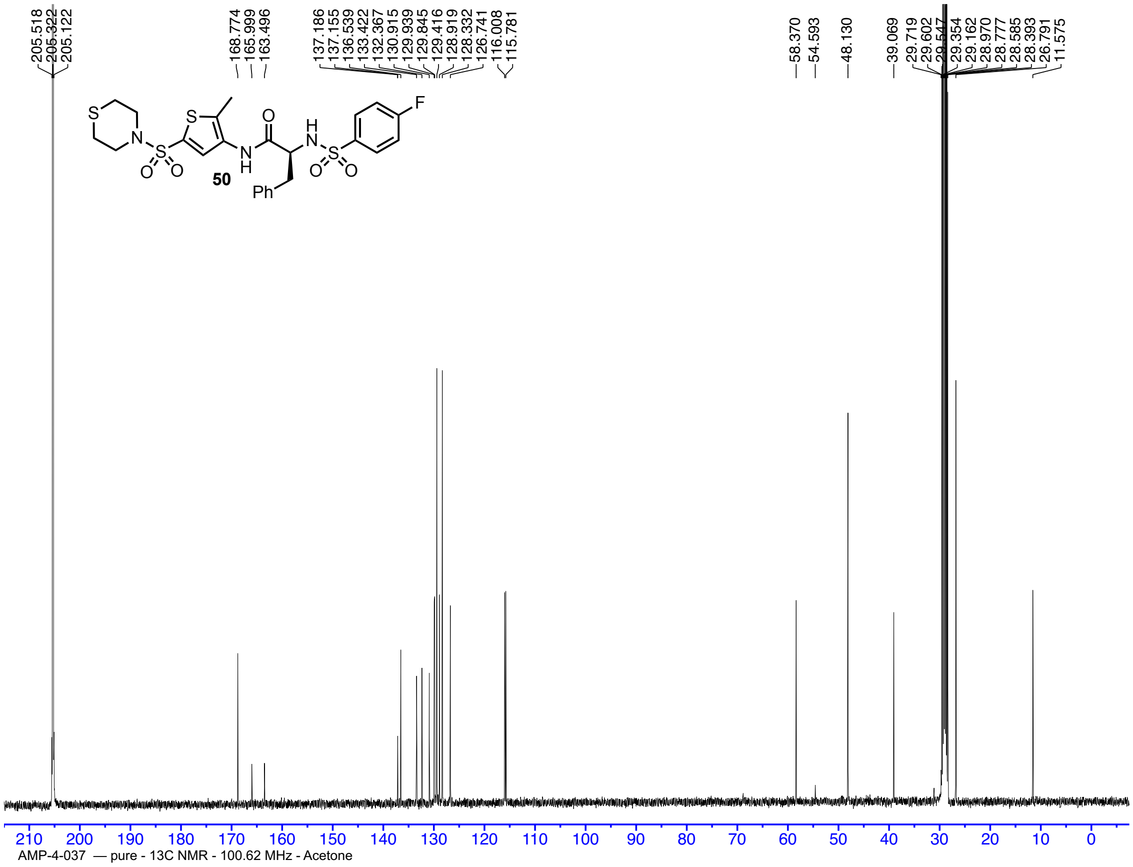

AMP-4-037 — pure - 13C NMR - 100.62 MHz - Acetone

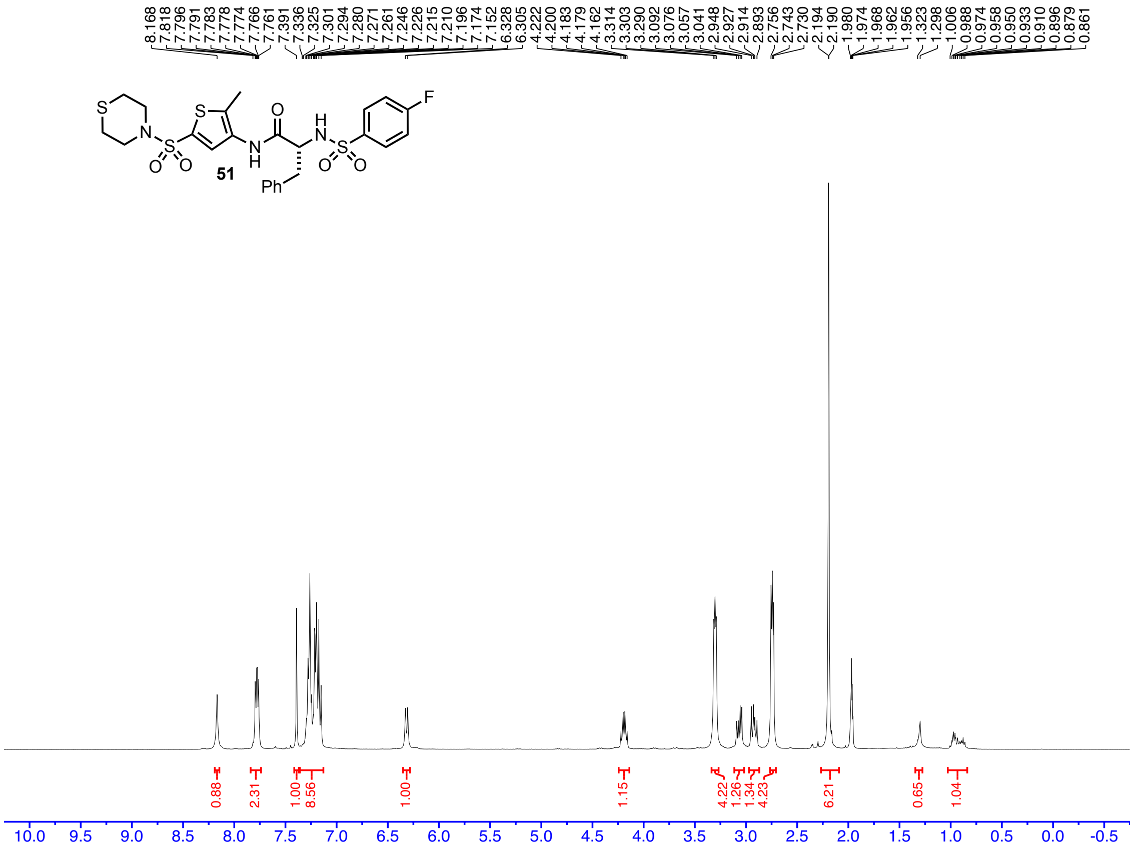

AMP-4-038 — dried - 1H NMR - 400.13 MHz - CD3CN

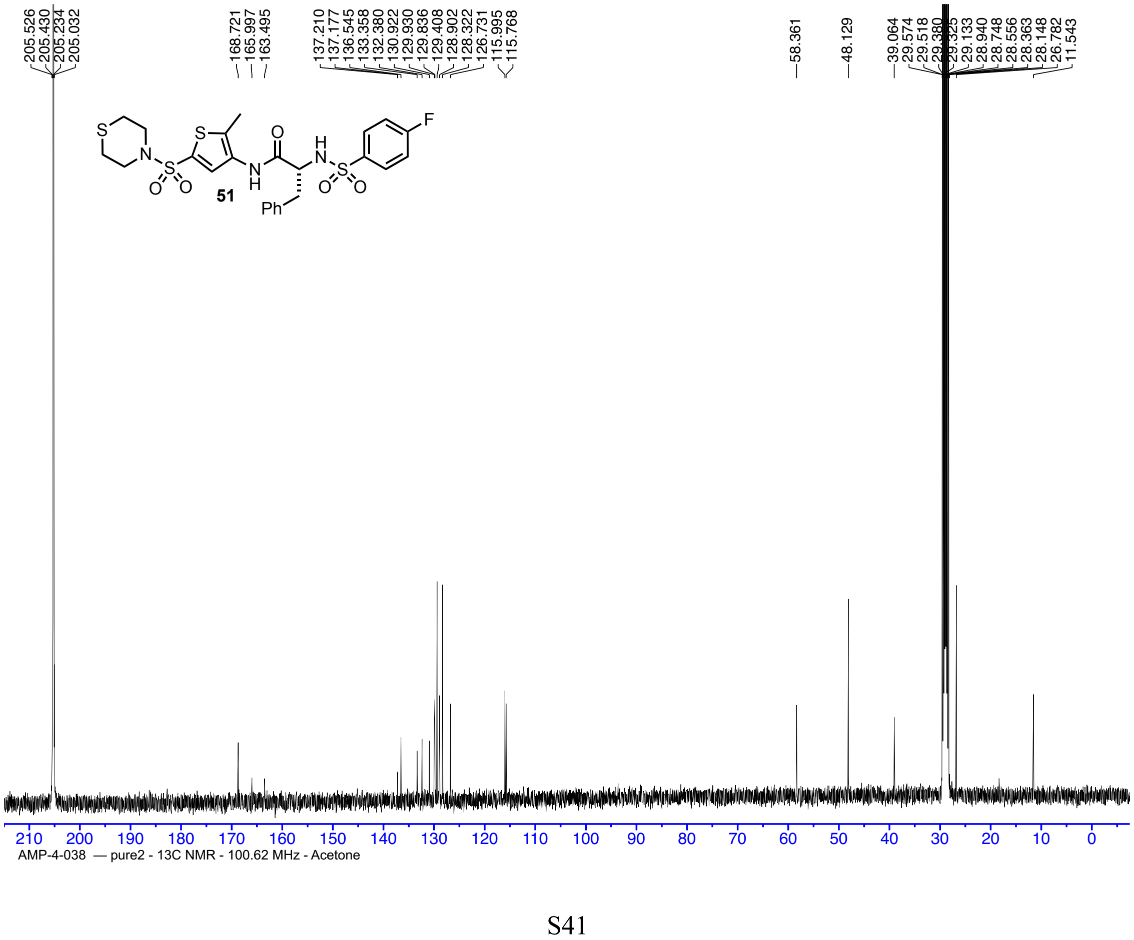

AMP-4-038 — pure2 - 13C NMR - 100.62 MHz - Acetone

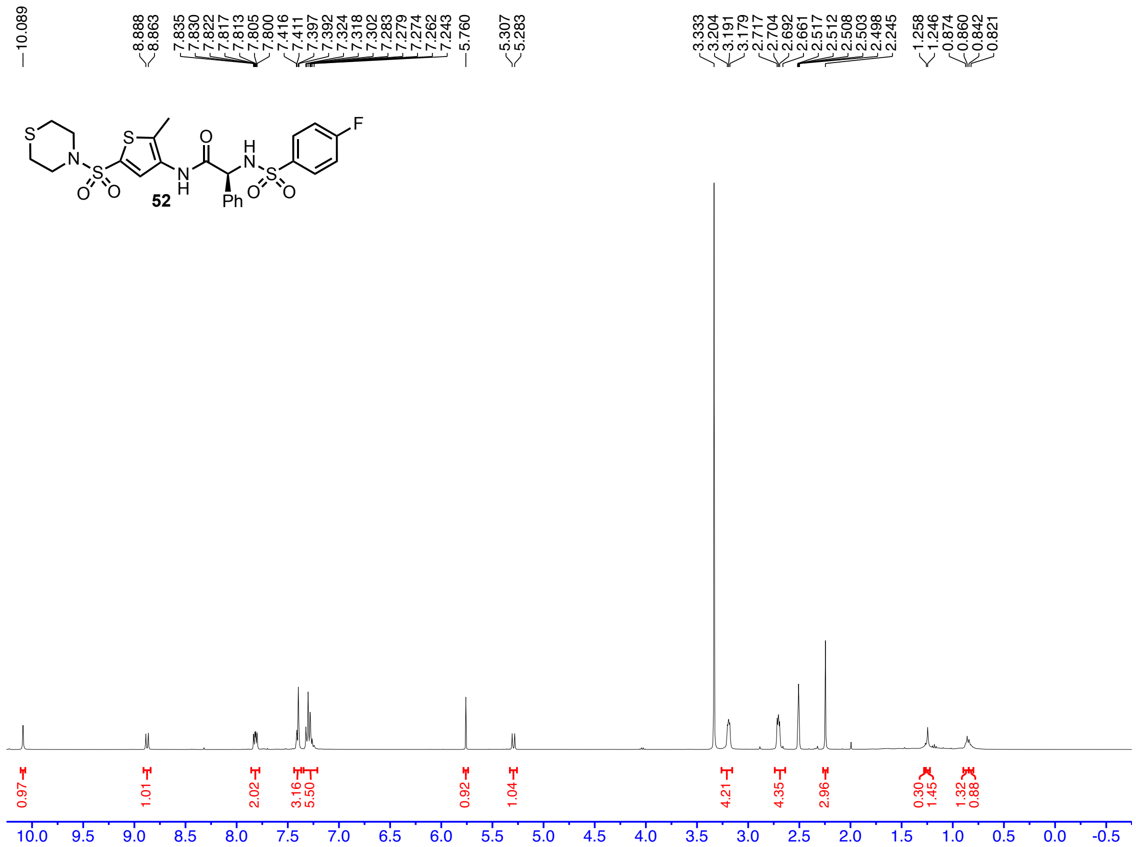

RA-03-121 - 1H NMR - 400.13 MHz - DMSO

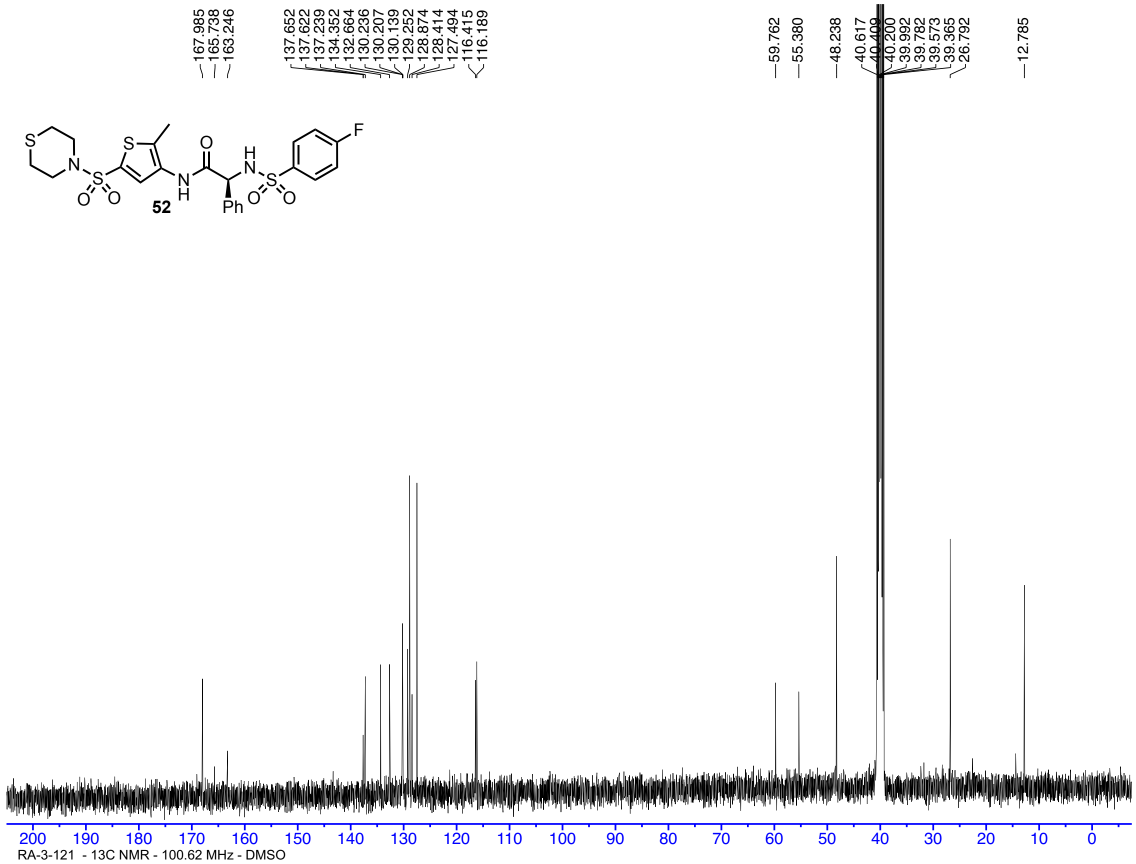

RA-3-121 - 13C NMR - 100.62 MHz - DMSO

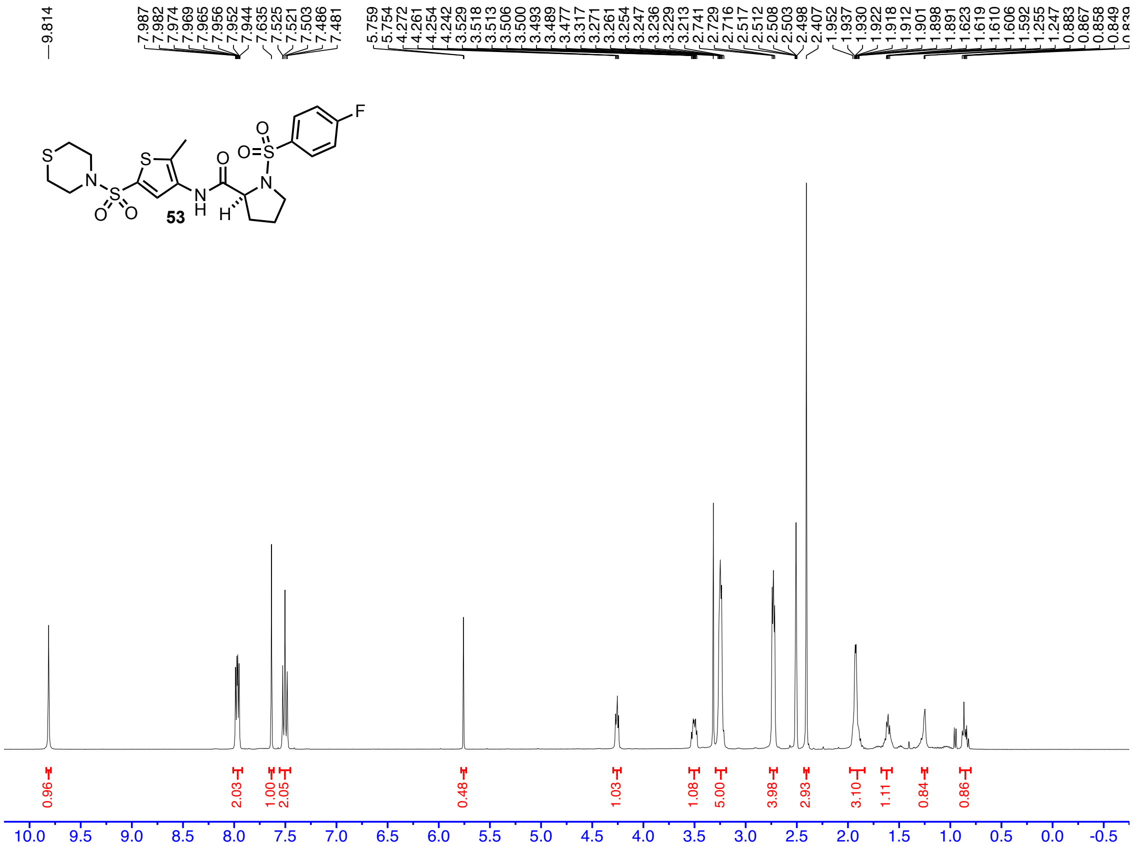

RA-3-106 - 1H NMR - 400.13 MHz - DMSO

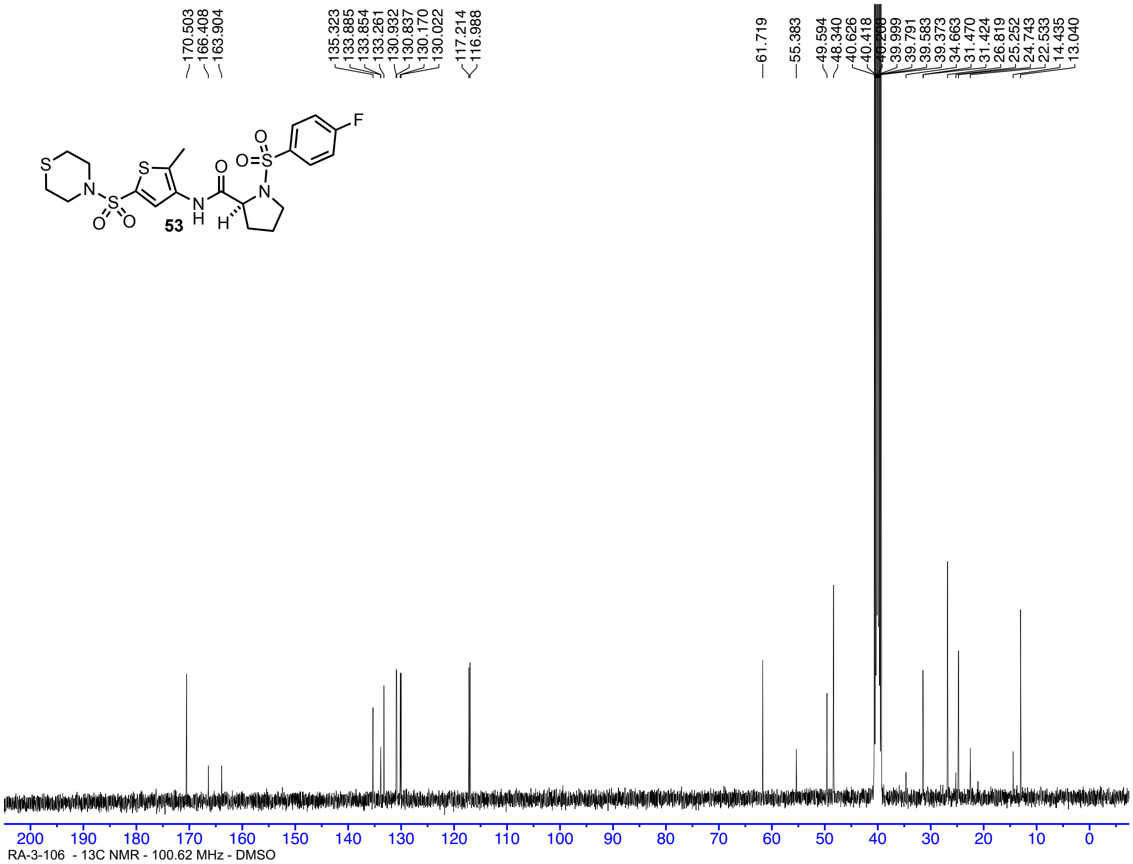

RA-3-106 - 13C NMR - 100.62 MHz - DMSO

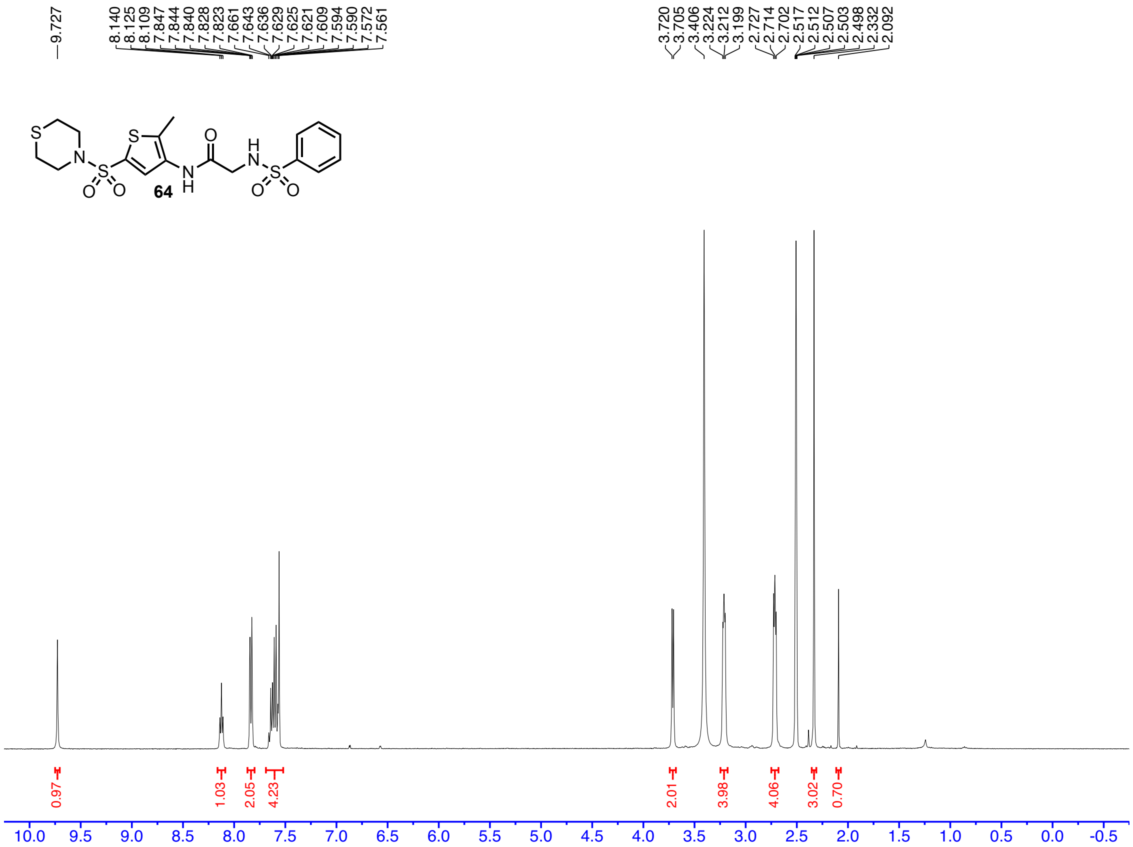

STM-4-97 (8-19-22) - 1H NMR - 400.13 MHz - DMSO

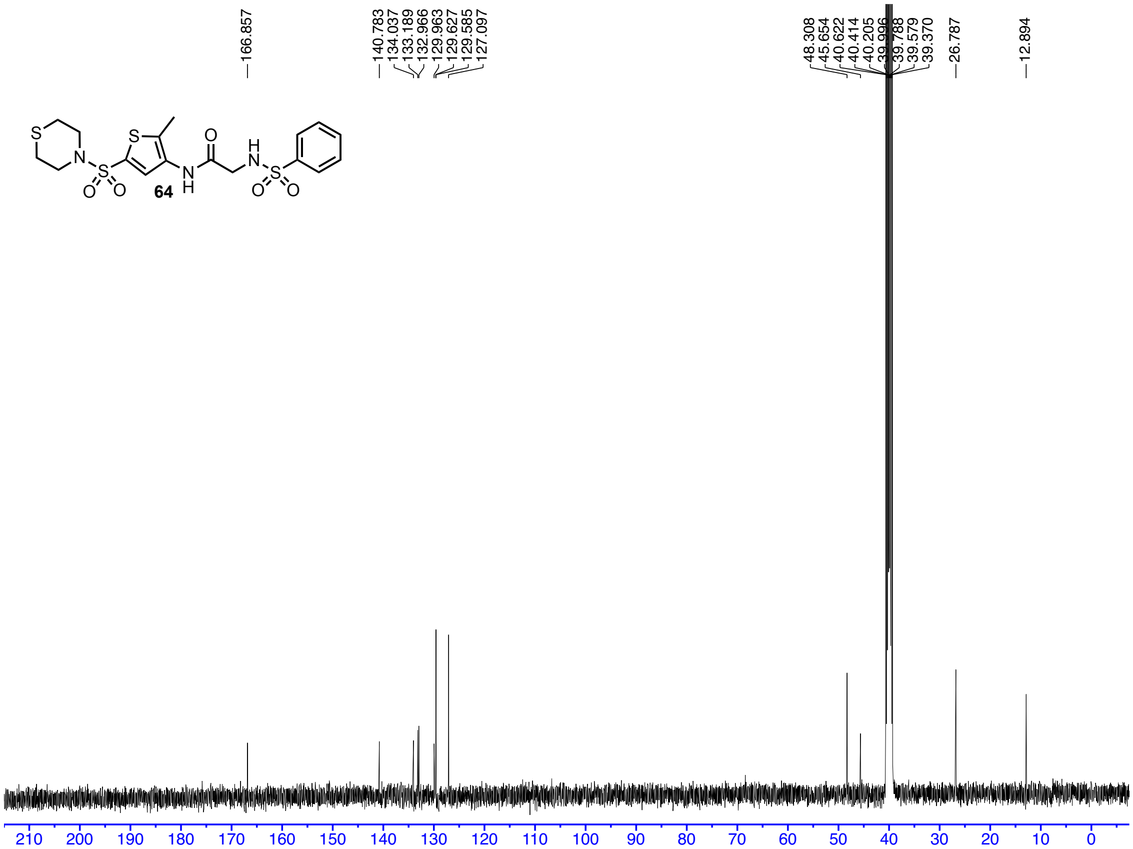

STM-4-97 CNMR (8-19-22) - 13C NMR - 100.62 MHz - DMSO

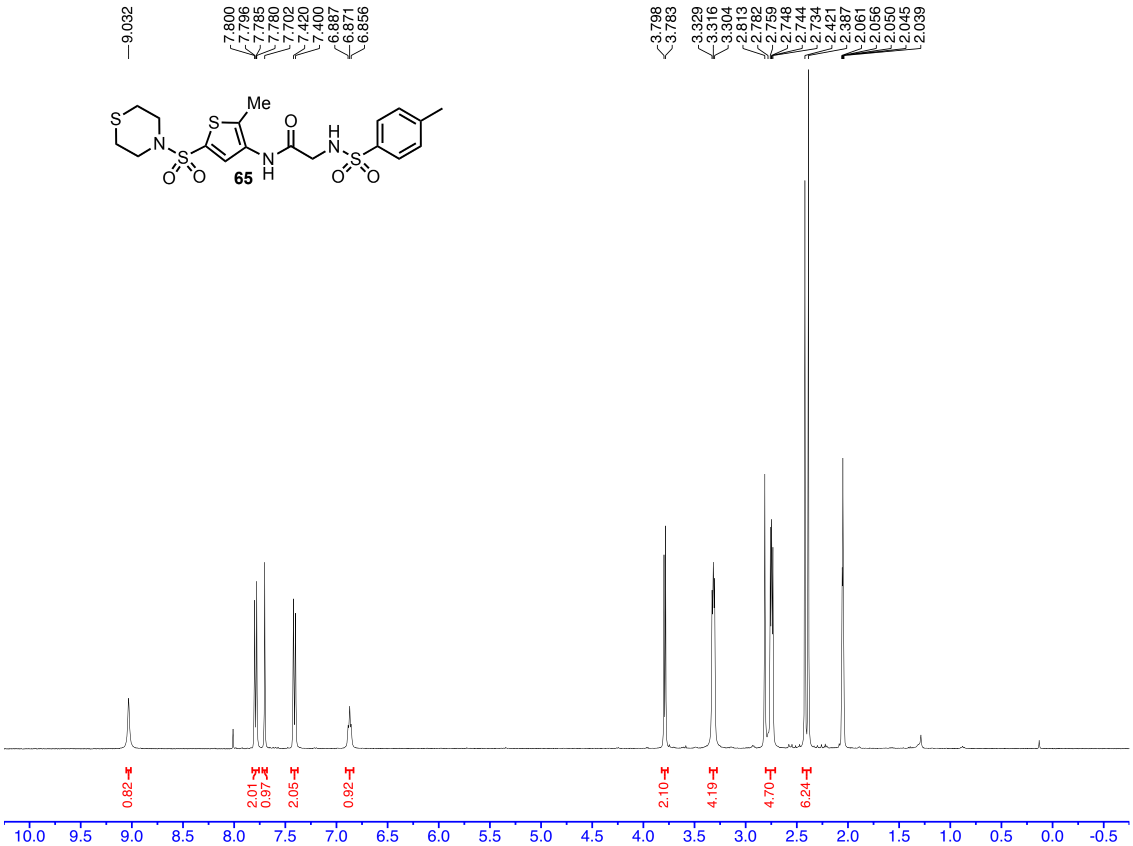

STM-4-75 (8-16-22) - 1H NMR - 400.13 MHz - Acetone

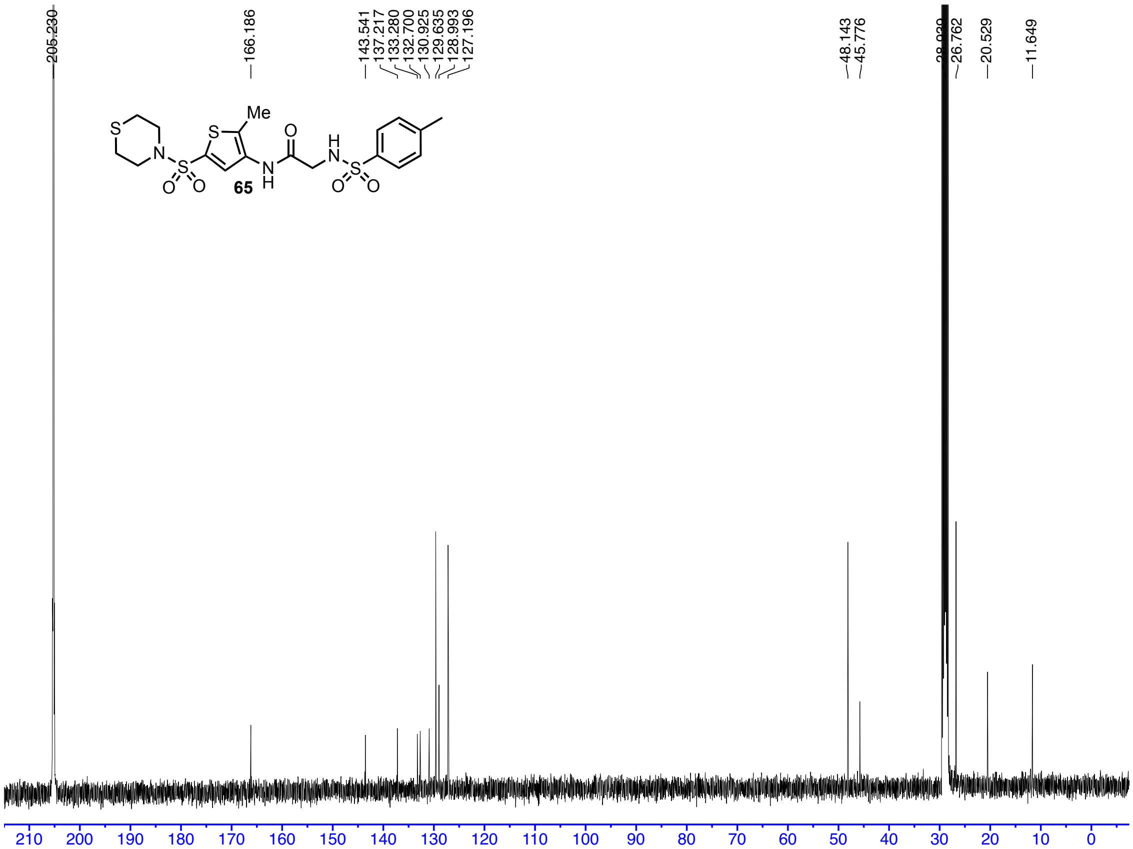

STM-4-75 CNMR (8-16-22) - 13C NMR - 100.62 MHz - Acetone

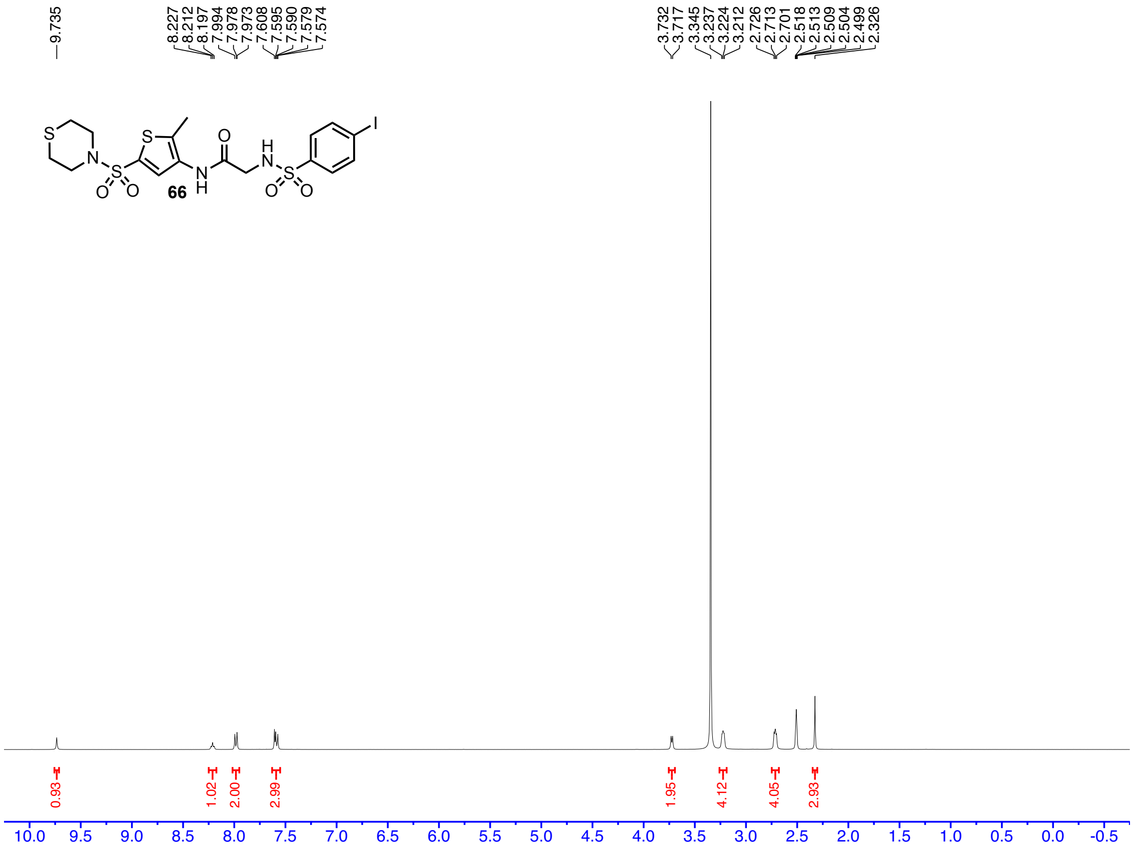

STM-4-095 — check - 1H NMR - 400.13 MHz - DMSO

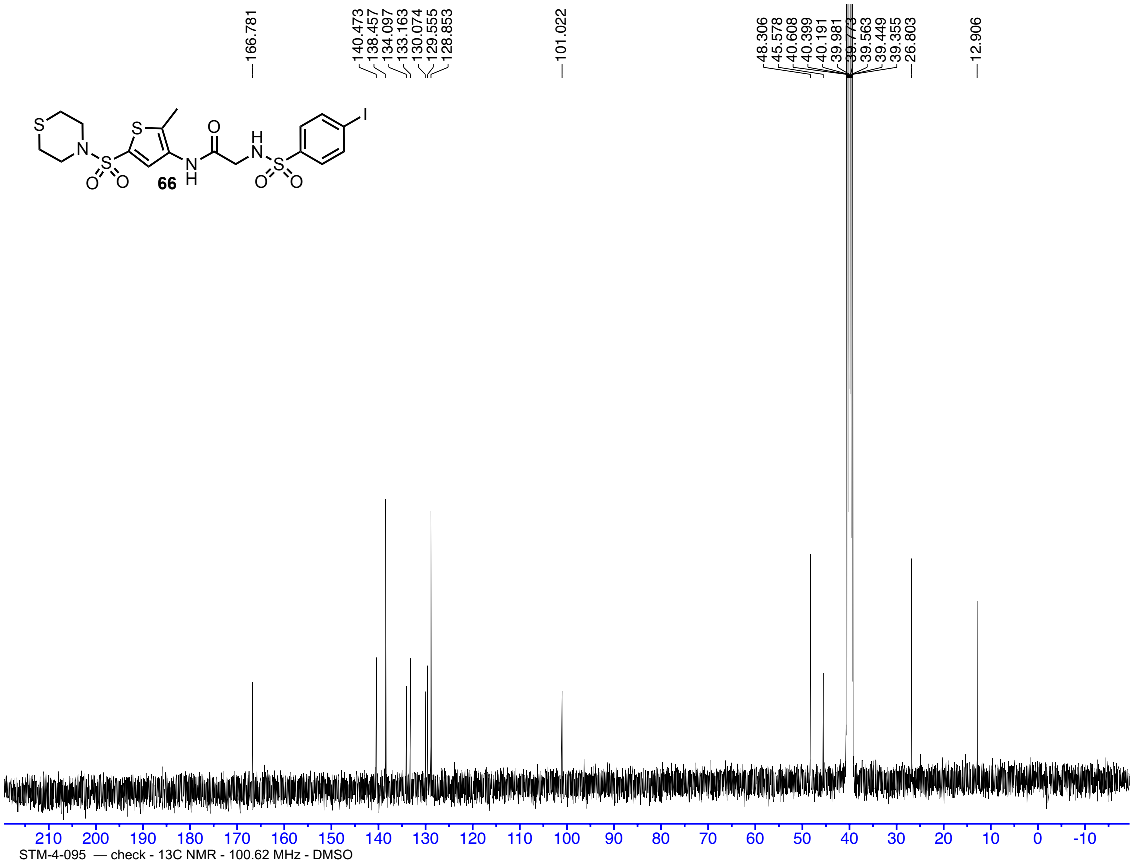

STM-4-095 — check - 13C NMR - 100.62 MHz - DMSO

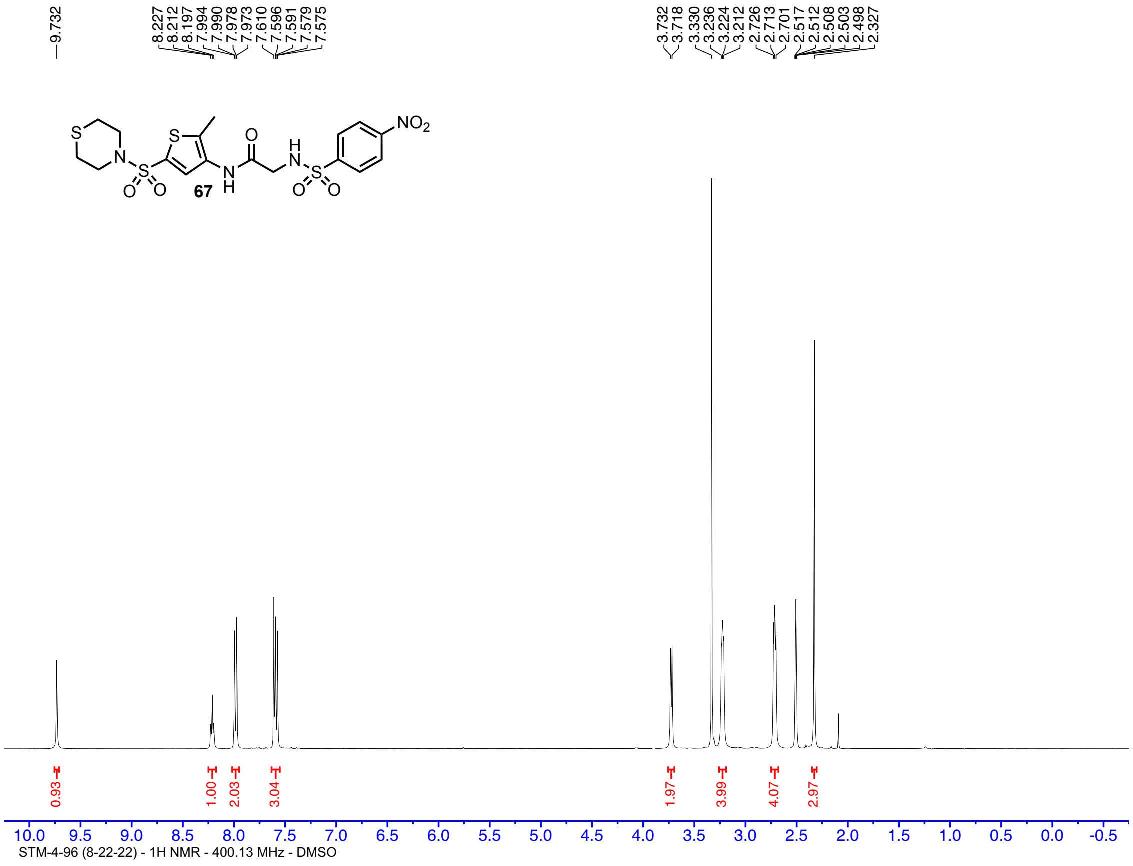

9.732
8.227
8.212
8.197
7.994
7.990
7.978
7.973
7.610
7.596
7.591
7.579
7.575
3.732
3.718
3.330
3.236
3.224
3.212
2.726
2.713
2.701
2.517
2.512
2.508
2.503
2.498
2.327
NO2
S
N
O
H
67
0.93
1.00
2.03
3.04
1.97
3.99
4.07
2.97
STM-4-96 (8-22-22) - 1H NMR - 400.13 MHz - DMSO

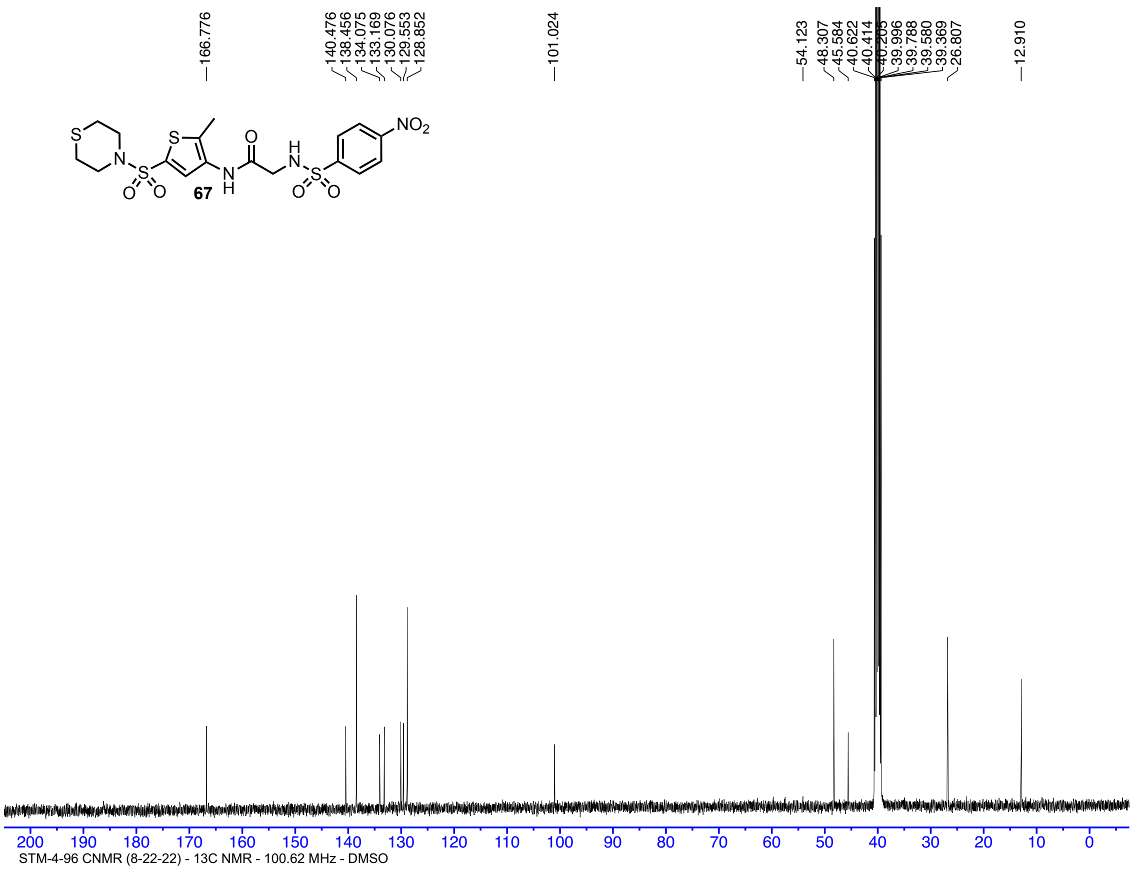

166.776
140.476
138.456
134.075
133.169
130.076
129.553
128.852
101.024
54.123
48.307
45.584
40.622
40.414
39.996
39.788
39.580
39.369
26.807
12.910
NO2
67
STM-4-96 CNMR (8-22-22) - 13C NMR - 100.62 MHz - DMSO

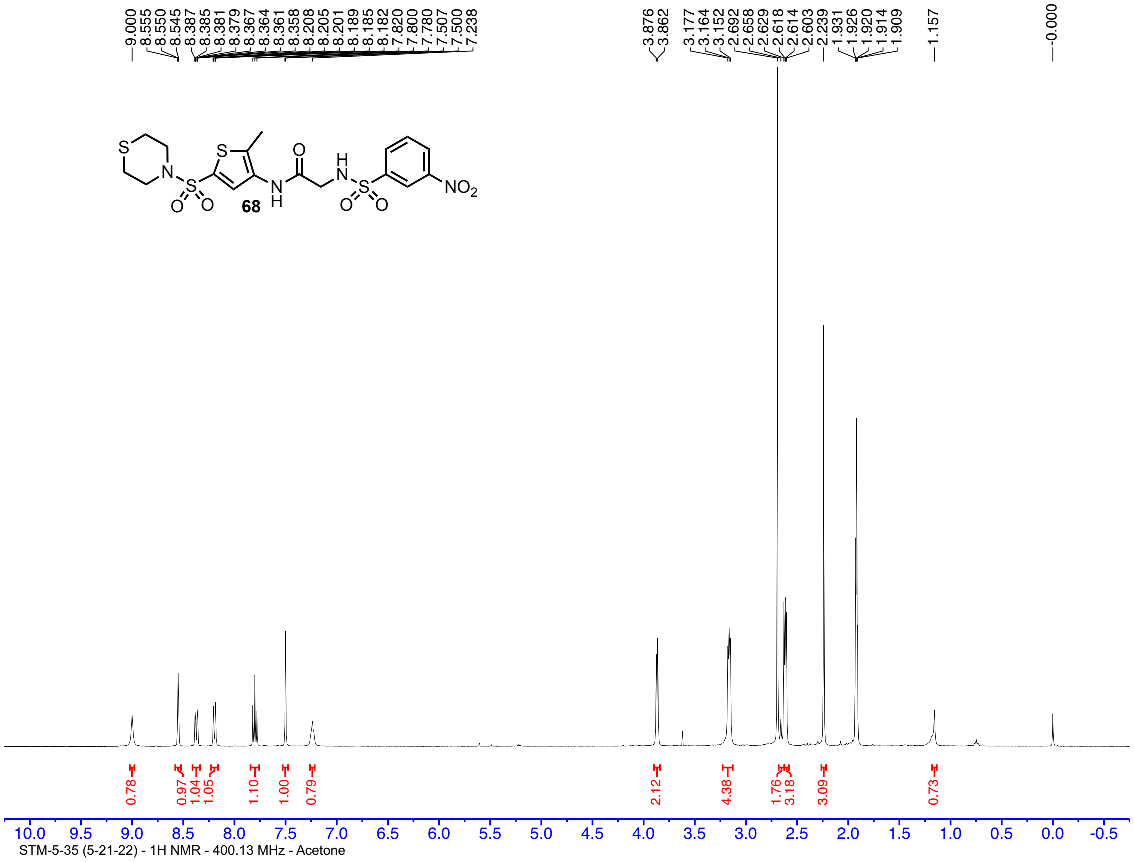

STM-5-35 (5-21-22) - 1H NMR - 400.13 MHz - Acetone

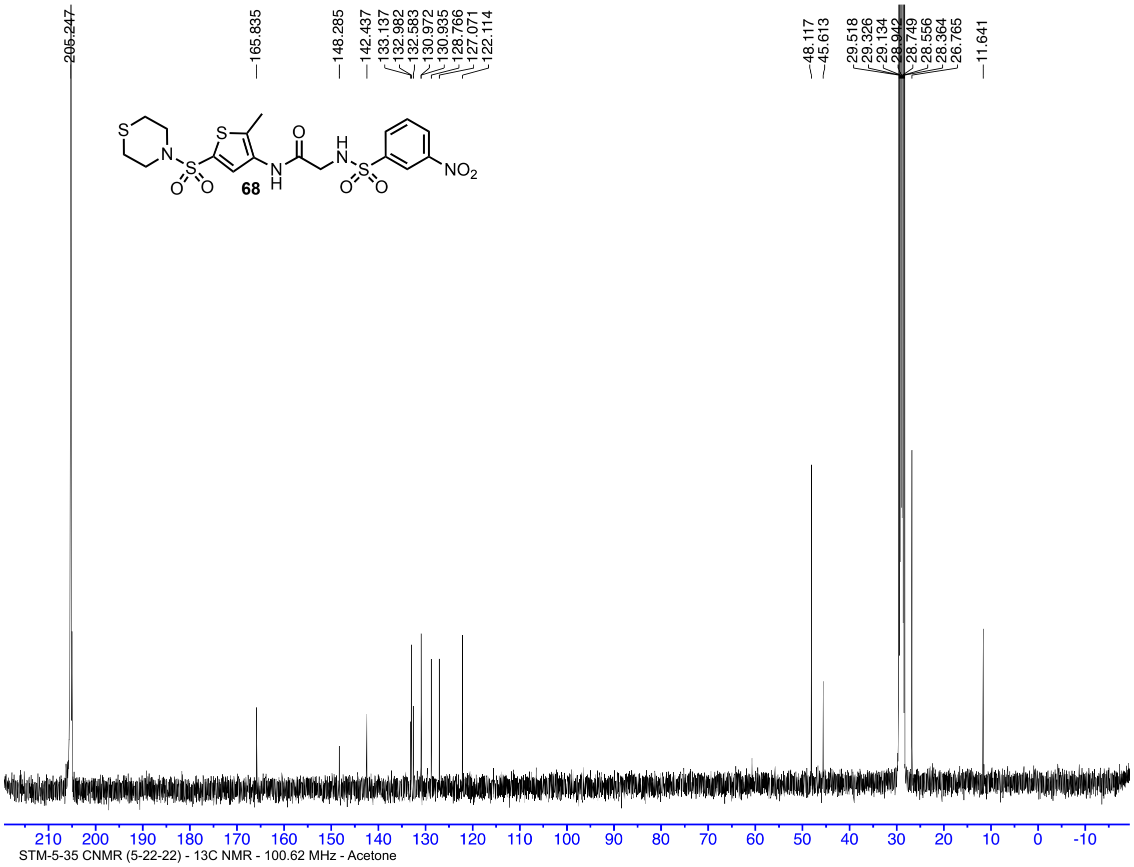

STM-5-35 CNMR (5-22-22) - 13C NMR - 100.62 MHz - Acetone

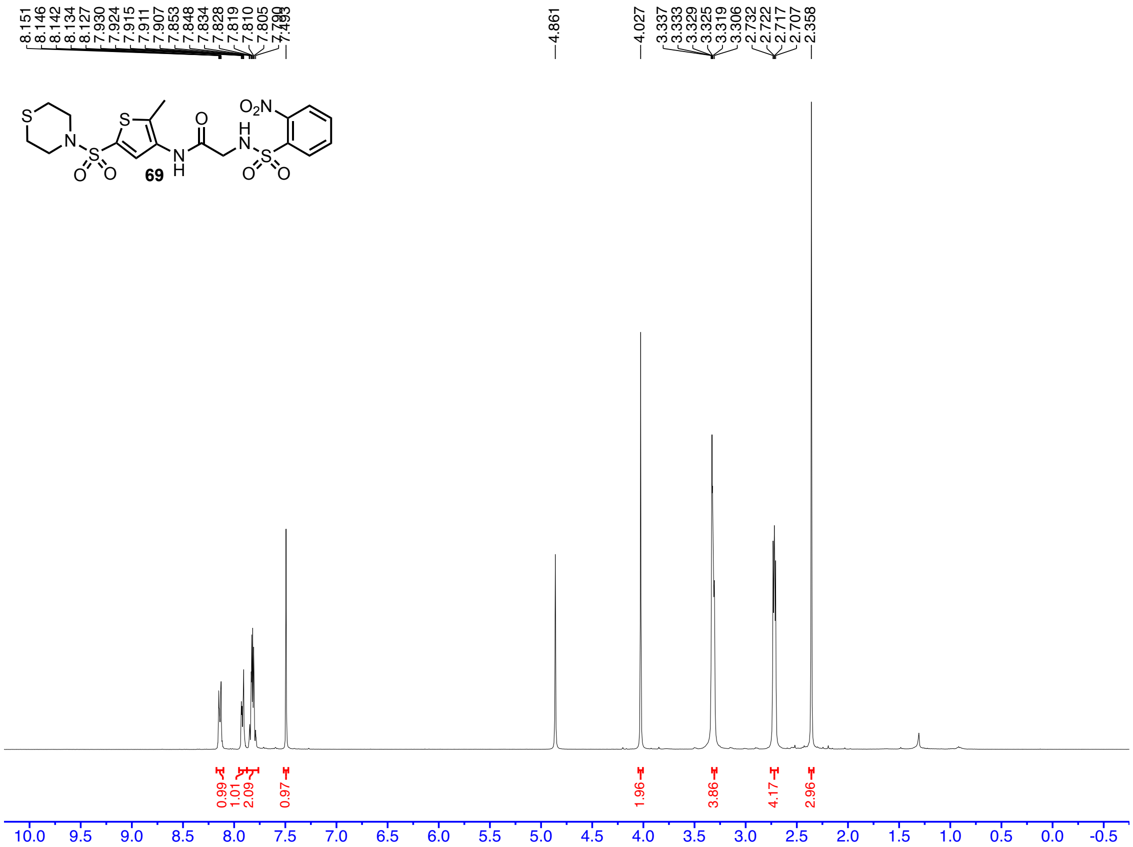

STM-5-34 (4-26-22) - 1H NMR - 400.13 MHz - MeOD

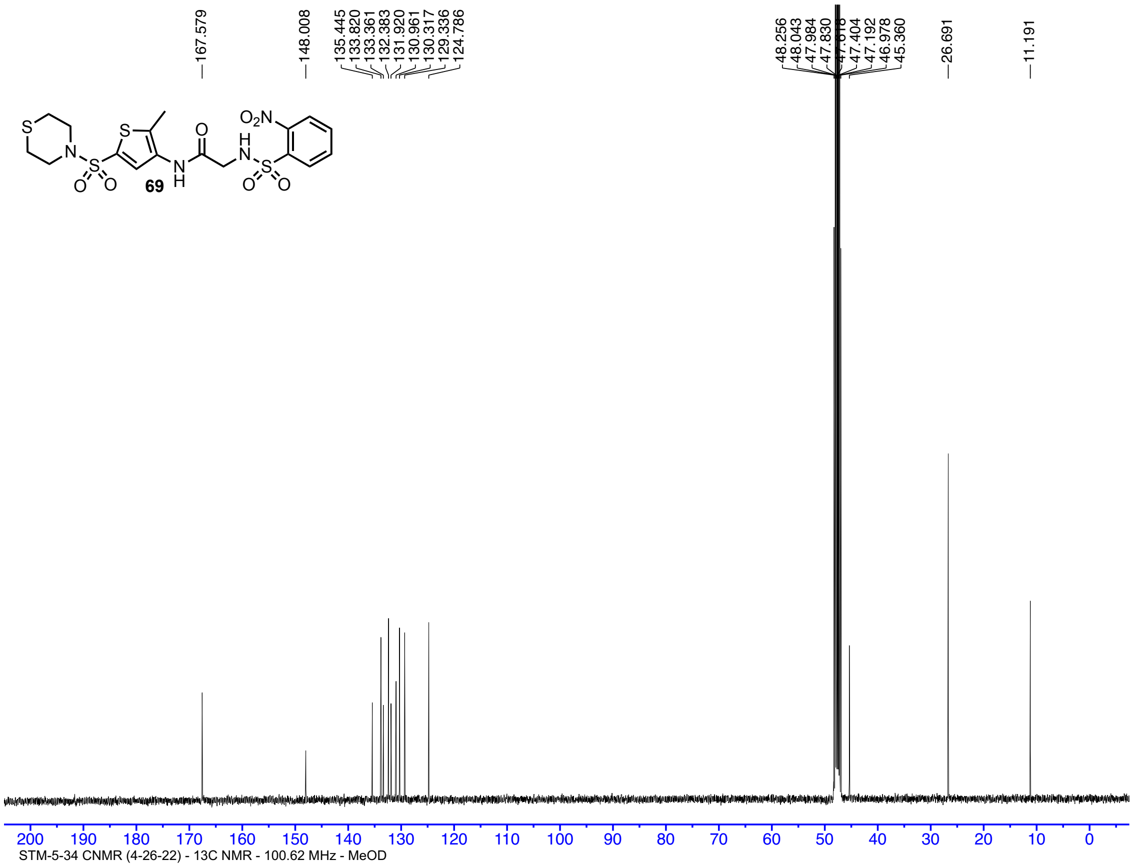

STM-5-34 CNMR (4-26-22) - 13C NMR - 100.62 MHz - MeOD

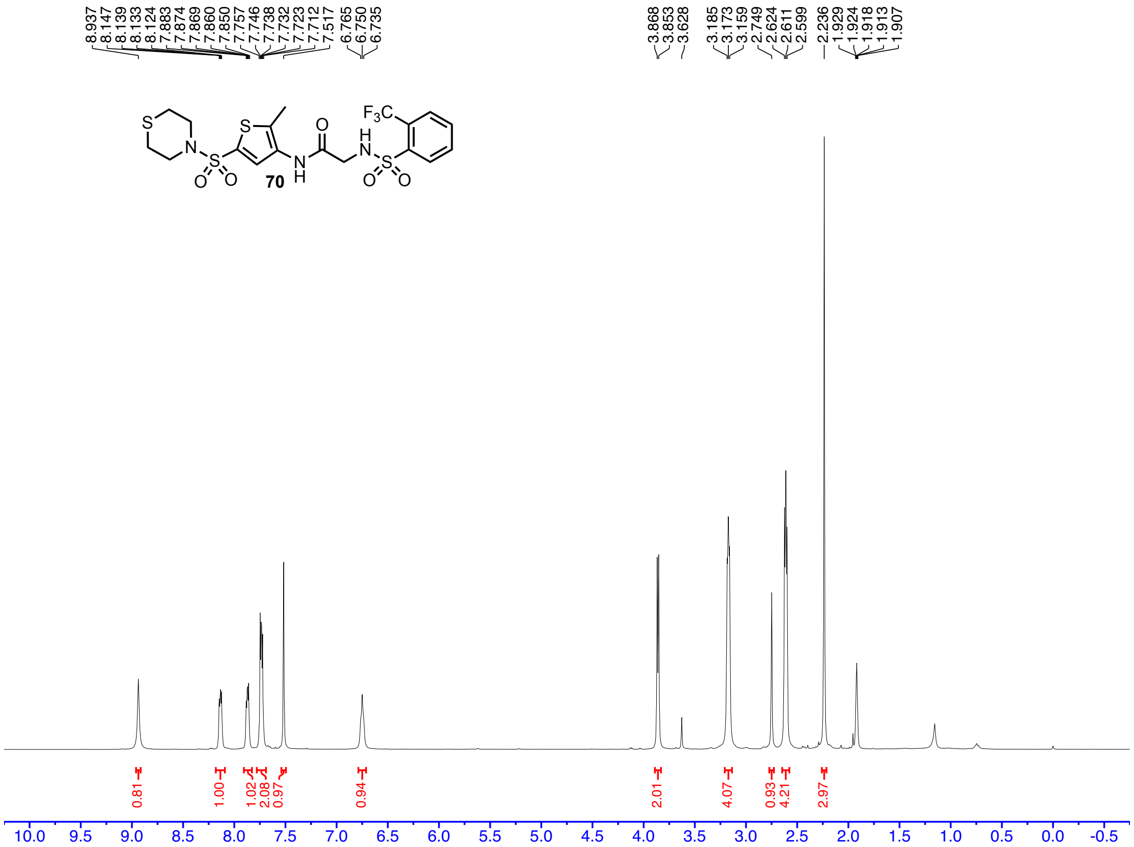

STM-5-98 (6-29-22) - 1H NMR - 400.13 MHz - Acetone

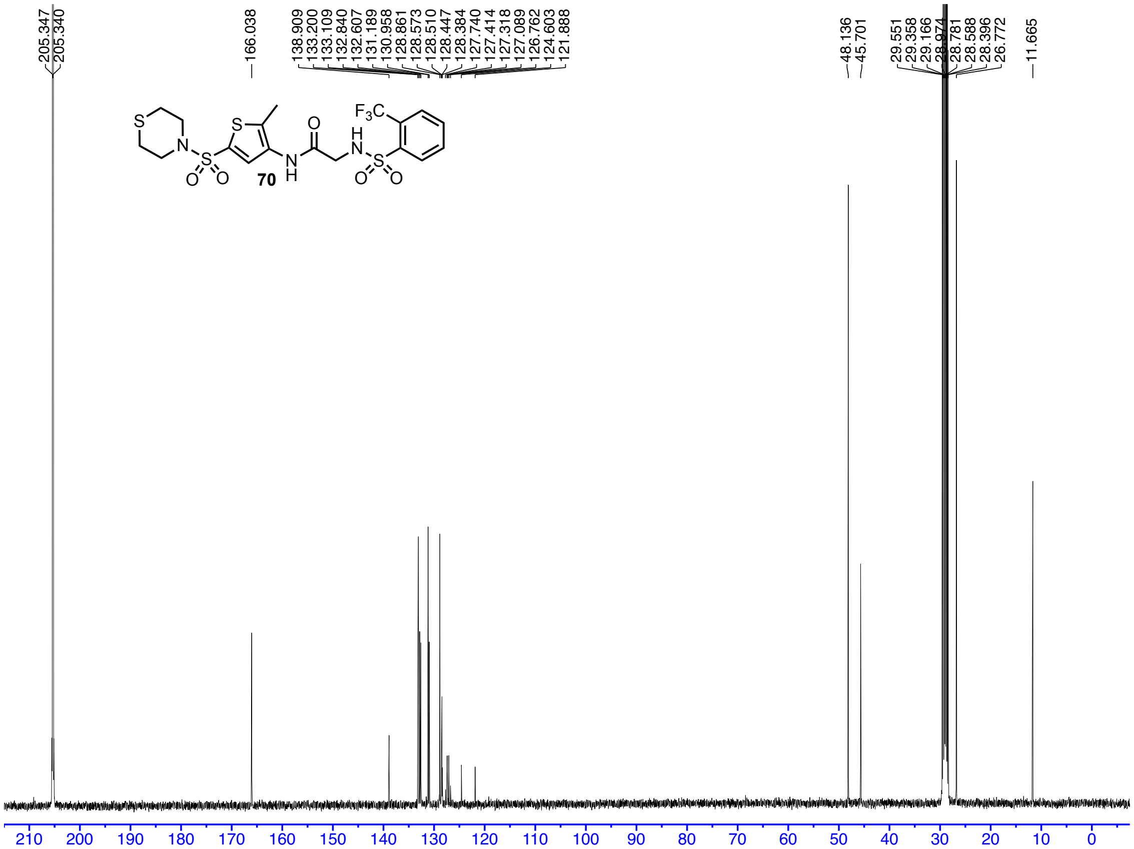

STM-5-98 CNMR (6-29-22) - 13C NMR - 100.62 MHz - Acetone

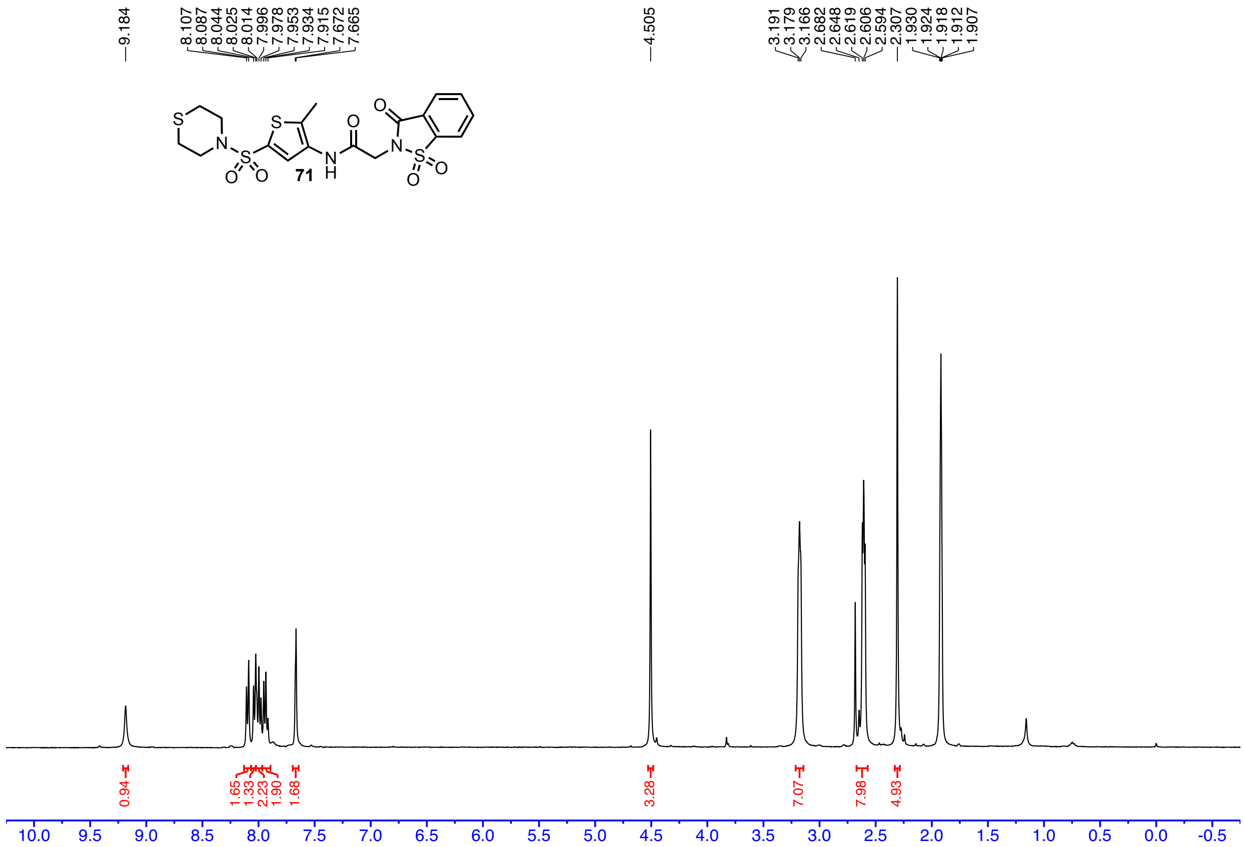
STM-5-97 (6-29-22) - 1H NMR - 400.13 MHz - Acetone

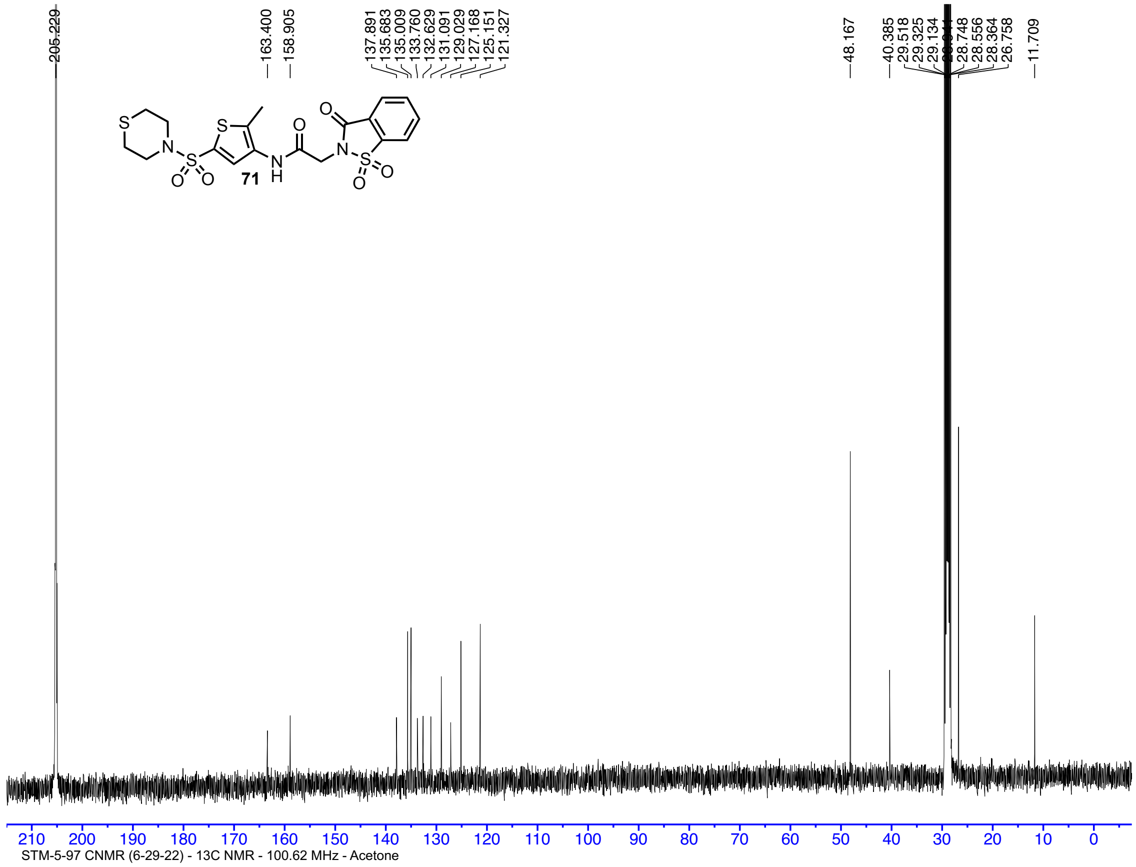
STM-5-97 CNMR (6-29-22) - 13C NMR - 100.62 MHz - Acetone

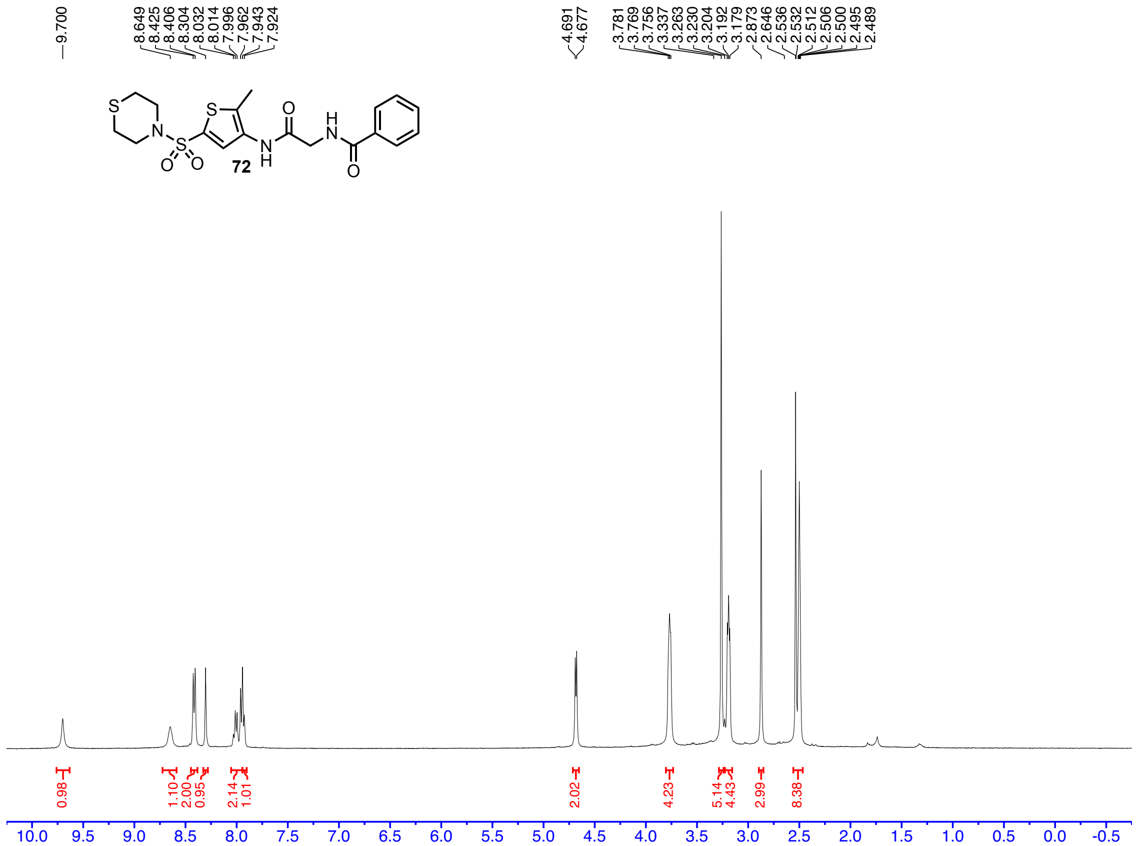

STM-4-91 (8-17-22) - 1H NMR - 400.13 MHz - Acetone

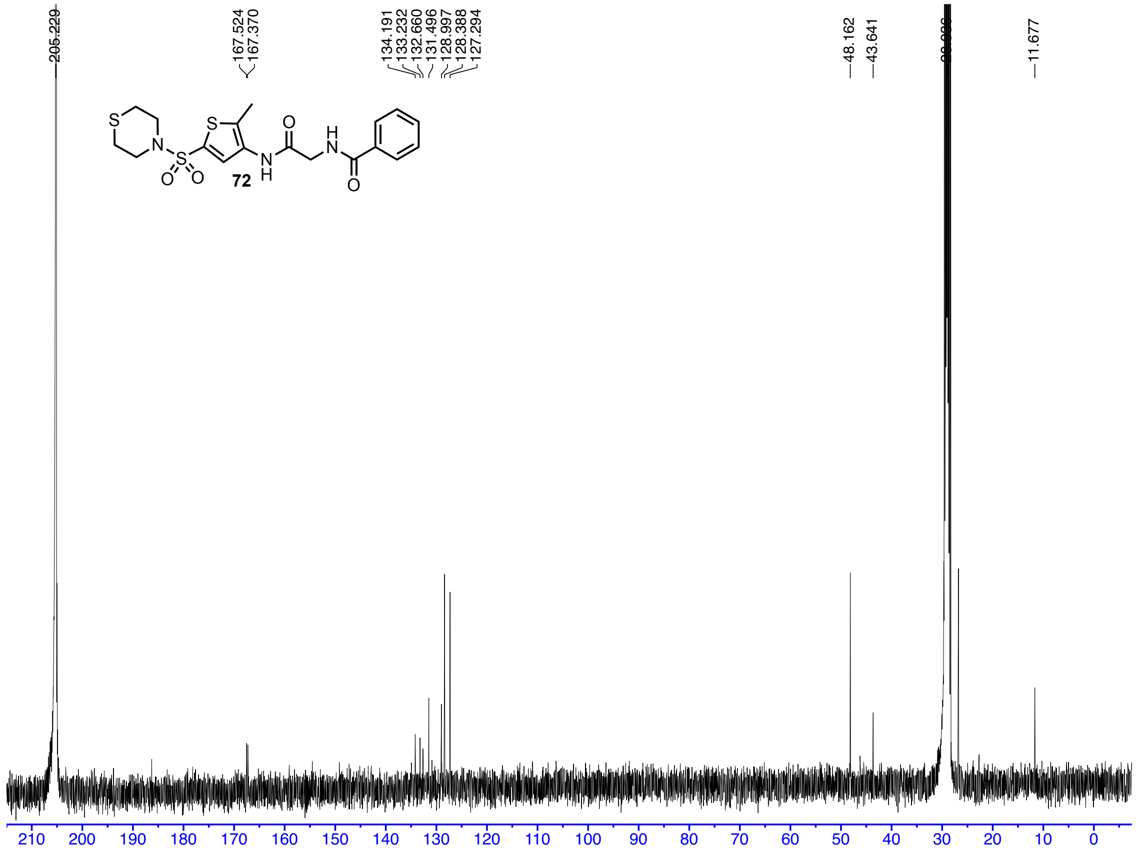

STM-4-91 CNMR (8-17-22) - 13C NMR - 100.62 MHz - Acetone

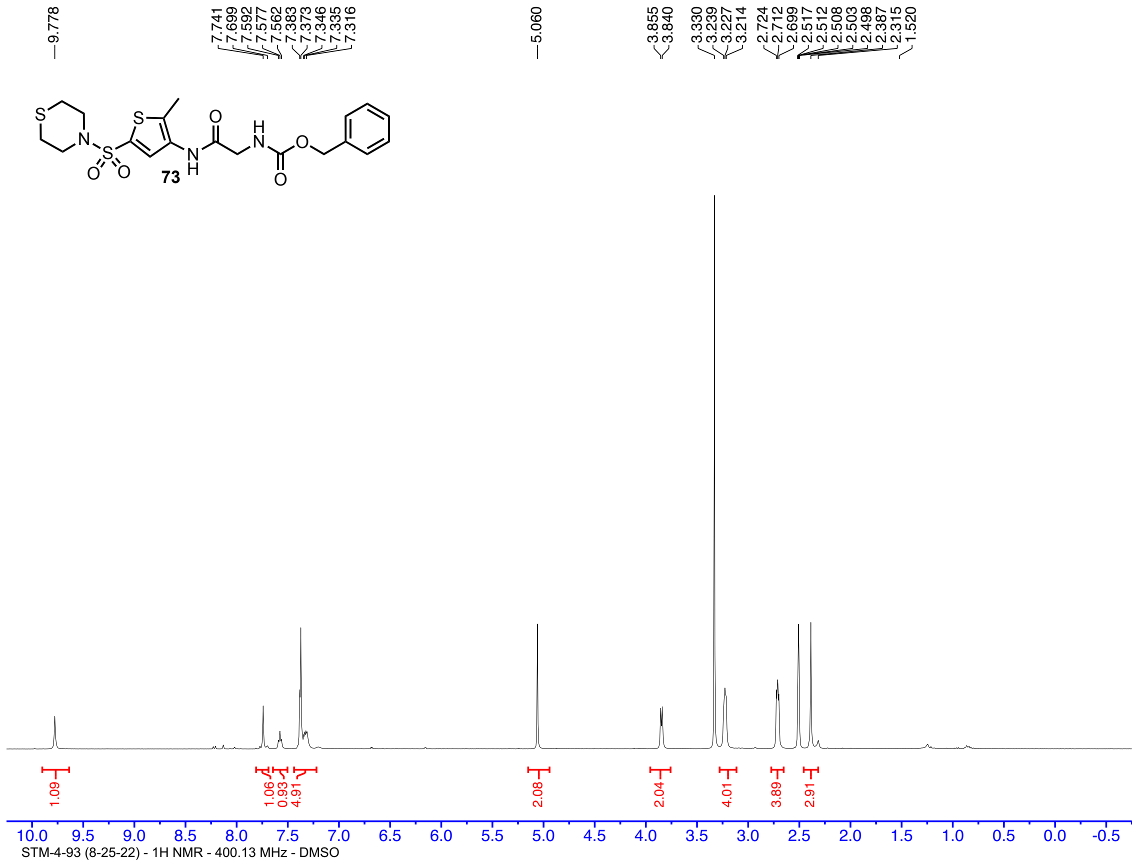
9.778
7.741 7.699 7.592 7.577 7.562 7.383 7.373 7.346 7.335 7.316
5.060
3.855 3.840
3.330 3.239 3.227 3.214
2.724 2.712 2.699 2.517 2.512 2.508 2.503 2.498 2.387 2.315 1.520
73
1.09
1.06 0.93 4.91
2.08
2.04
4.01
3.89
2.91
10.0 9.5 9.0 8.5 8.0 7.5 7.0 6.5 6.0 5.5 5.0 4.5 4.0 3.5 3.0 2.5 2.0 1.5 1.0 0.5 0.0 -0.5
STM-4-93 (8-25-22) - 1H NMR - 400.13 MHz - DMSO

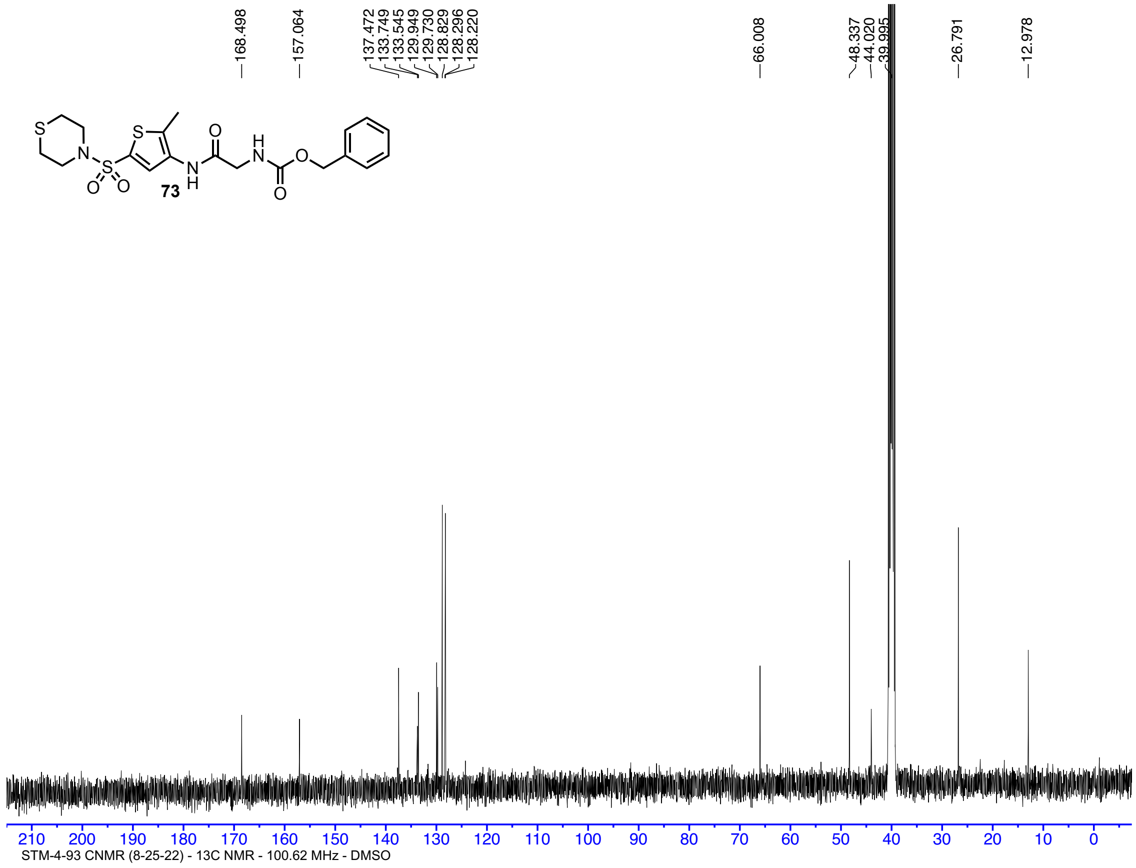
168.498
157.064
137.472 133.749 133.545 129.949 129.730 128.829 128.296 128.220
66.008
48.337 44.020 39.995
26.791
12.978
73
210 200 190 180 170 160 150 140 130 120 110 100 90 80 70 60 50 40 30 20 10 0
STM-4-93 CNMR (8-25-22) - 13C NMR - 100.62 MHz - DMSO
